# The JeffSTARS Advocacy and Community Partnership Elective: A Closer Look at Child Health Advocacy in Action

**DOI:** 10.15766/mep_2374-8265.10526

**Published:** 2016-12-31

**Authors:** Esther K. Chung, Stephanie Kahn, Marc Altshuler, J. Lindsey Lane, James Plumb

**Affiliations:** 1 Professor of Pediatrics, Sidney Kimmel Medical College at Thomas Jefferson University; 2 Nemours and Director, JeffSTARS; 3 First-year Medical Student, Sidney Kimmel Medical College at Thomas Jefferson University; 4 Past Educational Coordinator, JeffSTARS at Jefferson Pediatrics/Nemours duPont Philadelphia; 5 Associate Professor of Family and Community Medicine, Sidney Kimmel Medical College at Thomas Jefferson University; 6 Associate Program Director of the Family and Community Medicine Residency, Sidney Kimmel Medical College at Thomas Jefferson University; 7 Professor of Pediatrics, University of Colorado School of Medicine; 8 Vice Chair of Medical Education, University of Colorado School of Medicine; 9 Professor of Family and Community Medicine, Sidney Kimmel Medical College at Thomas Jefferson University; 10 Director of the Center on Urban Health, Thomas Jefferson University; 11 Associate Course Director, JeffSTARS

**Keywords:** Social Determinants of Health, Poverty, Editor's Choice, Advocacy, Health Policy, Child Health, Women's Health

## Abstract

**Introduction:**

Advocacy and service-learning increasingly are being incorporated into medical education and residency training. The Jefferson Service Training in Advocacy for Residents and Students (JeffSTARS) curriculum is an educational program for Thomas Jefferson University and Nemours trainees. The JeffSTARS Advocacy and Community Partnership Elective is one of two core components of the larger curriculum.

**Methods:**

The elective is a monthlong rotation that provides trainees in their senior year of medical school or residency training the opportunity to learn about health advocacy in depth. Trainees develop a basic understanding of social determinants of health, learn about health policy, participate in legislative office visits, and work directly with community agencies on a mutually agreeable project. The elective provides advocacy training to self-selected trainees from area medical schools and residency programs to develop a cadre of physicians empowered to advocate for child health.

**Results:**

JeffSTARS has advanced the field of child health advocacy locally by forging new partnerships and building a network of experts, agencies, and academic institutions. After this experience, trainees realize that their health expertise is very valuable to health advocacy and policy development. JeffSTARS is recognized nationally as one of a growing number of advocacy training programs for students and residents, with trainees presenting selected projects at national meetings.

**Discussion:**

Teaching advocacy has raised awareness about social determinants of health, community resources, and the medical home. One of the many benefits of the elective has been to strengthen the skills and expertise of trainees and faculty members alike.

## Educational Objectives

After participating in the advocacy elective, trainees will be able to:

1. Understand how social determinants and policies affect child health.
2. Partner with community organizations to advocate for community and child health.
3. Model practical and effective advocacy skills for lifelong advocacy, including, but not limited to, building relationships in the community, incorporating advocacy into clinical practice, and identifying policy solutions to child health problems.

## Introduction

Physicians play an important role by advocating for health in daily practice. More than 90% of surveyed physicians rated community participation and collective advocacy as important.^1^ Although well positioned to advocate for community health, physicians often lack necessary training and skills.^1–3^ Medical trainees encounter the effects of poverty, racism, and other social determinants of health, but training opportunities to discuss and consider how to address these issues are limited. Faculty members are often clinicians with little time allotted for advocacy, and role models who combine clinical care with community advocacy may be difficult for trainees to identify on their own. Health problems related to obesity, violence, tobacco smoke, lead poisoning, sexual health, literacy, substance abuse, mental health, and poverty require creative solutions that incorporate family, physician, and community perspectives. Advocacy and service-learning, core principles of medical professionalism,^4,5^ increasingly are being incorporated into medical education and residency training. Novel approaches to teaching advocacy range from videos and modules to 1-month required curricula.^6–11^ The Jefferson Service Training in Advocacy for Residents and Students (JeffSTARS) elective builds on these approaches and the work of others, including McIntosh, Block, Kapsak, and Pearson,^12^ who implemented a required fourth-year clerkship based on a successful elective in community health, and Earnest, Wong, and Federico,^13^ who defined and described the spectrum of physician advocacy.

Various opportunities are offered at our institution for trainees to be directly involved in working with underserved populations, but prior to the JeffSTARS curriculum, with its advocacy elective, there was no formalized advocacy training. In 2009, the Jefferson/Nemours pediatric residency training program partnered with the Sidney Kimmel Medical College (SKMC) of Thomas Jefferson University to develop and implement the JeffSTARS curriculum with support from an American Academy of Pediatrics (AAP) Community Access to Child Health Residency Training and Community Pediatrics Training Initiative grant^14^ and a 3-year advocacy education grant from the Center on Medicine as a Profession at Columbia University.^15^ Now in its eighth year, JeffSTARS is integrated into the overall curriculum for students and residents at SKMC and Nemours. Below, we describe the JeffSTARS advocacy elective in further detail.

## Methods

The advocacy elective is offered to fourth-year medical students and second- and third-year pediatric and family and community medicine residents. The elective has a child health focus; however, the curriculum can be tailored to trainees interested in pursuing careers in specialties other than pediatrics, including obstetrics and gynecology, internal medicine, family medicine, and emergency medicine. Trainees learn about the elective at orientation, and the course is listed in the medical school curriculum as an interdepartmental elective. Faculty members at all of the local training institutions are aware that the advocacy elective, unique to our institution, is offered throughout the academic year.

The educational approach of the advocacy elective was modeled after the physician (formerly Soros) advocacy fellowship at the Center on Medicine as a Profession at Columbia University,^16^ where physicians spent 50% of their time working side-by-side with a community organization. In the advocacy elective, medical students and residents work with community agency professionals to address child health problems and to learn about health advocacy from nonclinical professionals for 50% of the rotation. The remaining 50% of time is spent in clinical care (30%) and in didactic and reflective sessions (20%).

The 1-month JeffSTARS advocacy elective is offered five times throughout each academic year and is run by a course director and an educational coordinator. To provide a rich, learner-driven experience, a maximum of four trainees participate each month. From past experience, the elective is most successful when learners are highly motivated to do independent work. Therefore, for fourth-year medical students, the elective is offered frequently at the start of the academic year when they are focused on building their portfolio for residency applications. Furthermore, the elective is not offered during the winter months, when holidays and weather conditions limit the ability to interact with agencies and legislative offices. The elective trainees, along with peers rotating through the outpatient setting, are required to participate in the following required educational sessions at our institution: weekly grand rounds, morning reports, advocacy cafés, and monthly advocacy journal clubs. By participating in sessions required of all outpatient trainees, those taking part in the elective are not isolated and can share their advocacy experience and knowledge with other trainees.

Implementing this elective at a new institution requires advance planning, a faculty champion, a division home, and support from clinical clerkship directors, residency program directors, the dean of academic affairs, and the medical school course review committee. Throughout this report, references are made to various course materials that are further detailed in the summary course implementation checklist (Appendix A). Similarly, prior to the start of each elective rotation, some advance planning is required. Further details are given below in the *Community engagement* section and also in the attached elective checklist (Appendix B).

### Elective Schedule

Elective participant schedules are divided into clinical, didactic, reflective and sharing, and community engagement blocks of time (Appendix C). Trainees spend 30% (1.5 days/week) of their time in clinical work in the pediatric or other designated specialty clinic, 20% (1 day/week) in didactic and reflective work (i.e., seminars and group discussions), and 50% (2.5 days/week) in community engagement—working closely with a community partner on a mutually agreeable advocacy project.

#### Clinical

The advocacy elective is run out of the Department of Pediatrics, but it is open to trainees pursuing other disciplines. Medical students and residents perform their clinical care in pediatrics in conjunction with the existing outpatient rotation. The trainees work clinically under the supervision of a faculty member, typically the course director or the faculty liaison from the chosen specialty clinic. The clinical component of the rotation allows trainees to continue to develop their clinical skills and provides an opportunity for learners to use newly acquired advocacy skills when interacting with patients. For example, the trainees are expected to practice in-depth interviews with their patients to better understand the social determinants that impact health. The trainees are also encouraged, on the other hand, to think about how their clinical work informs their current and future advocacy agendas.

#### Didactic

The didactic portion of the advocacy elective consists of seminars unique to the elective, as well as other sessions required for all trainees participating in the outpatient clinical rotation. One-hour seminars focus on developing advocacy skills that are applied to physician careers. Physician and nonphysician advocates are identified as potential seminar speakers prior to the start of the rotation. These advocates include local lawyers, community organization leaders, public health professionals, physicians, and lobbyists. Two to 3 months prior to the elective, the educational coordinator contacts potential speakers via email with dates of the upcoming elective and a list of seminar topics and learning objectives (Appendix D). Speakers choose the topic that best fits their professional expertise. The seminars are conversational in nature, but the speakers are asked to meet the three objectives associated with their topic as outlined in Appendix D. Reading materials for each seminar are determined by the speaker and the course director, and printed copies make up the course syllabus that is given as a three-ring binder to each trainee at the start of the elective. This syllabus undergoes minor changes depending on the availability of speakers and current events. In addition, supplementary materials may be created to enhance skill building. For example, with the seminar, “In-Depth Interviews to Understand Social Factors Affecting Your Patient's Health,” trainees are asked to complete a social history exercise. A copy of the bibliography listing these reading materials and supplementary materials can be found in Appendix E. For seminar-specific slides, see Appendix J. To reinforce newly established relationships with health advocates, trainees sign provided thank-you notes for seminar speakers at the end of the rotation. Further didactic sessions required of the elective trainees and all others participating in the outpatient clinical rotation include the following: weekly grand rounds, morning report, and a monthly advocacy journal club.

#### Reflective and sharing

Trainees are assigned a faculty mentor and a community mentor prior to the start of the rotation. The faculty mentor has expertise in working with community partners and in advising students. During the rotation, the trainees meet weekly for 1 hour with their faculty mentor to discuss their learning needs and strategies for working with their community partner in a nonclinical setting. The faculty mentor works with each learner to develop three learner-driven project goals and three overall objectives for the elective. Trainees also meet twice weekly with a community mentor, identified at their community site, to ensure that project goals are met and to discuss agency events and meetings. At the end of the rotation, participants share their advocacy experience with peers by describing their project, noting lessons learned, and expanding on how their experience will affect their future medical practice via a 15- to 20-minute PowerPoint presentation at an advocacy café. In addition to the faculty and community mentor meetings, trainees have the opportunity to reflect and share what they have learned with each other and the course director at a clinical case conference (scheduled in Appendix C), where each trainee provides a case and the others generate solutions and suggestions to advocate for the patient and family. The monthly advocacy journal clubs and weekly advocacy cafés required of all outpatient trainees allow the advocacy elective trainees to share what they have learned at the seminars and community sites with others participating in the required clerkship and outpatient rotation.

#### Community engagement

Three months before the start of the rotation, the course director meets by phone or in person (preferred) with each trainee to identify health advocacy areas of interest and to discuss expectations for the elective. Based on a trainee's identified interests and the course objectives, the director connects the trainee with several community agencies (for a description of the variety of agencies, see Appendix F). The community agency may be one known to the course director or one known to the trainee if the agency meets the following criteria: (1) works to improve the overall health and welfare of children, (2) has experience in community engagement and/or legislative advocacy, (3) has an office where daily business is conducted, and (4) has an available mentor who is nonclinical (see Appendix G). The trainee then contacts each of several agencies to determine which one is the best fit. The trainee and a leader at the community agency discuss the trainee's interests, the agency's areas of focus, and potential advocacy projects. Each trainee must identify a community partner and mentor at least 1 month prior to the start of the rotation. During the rotation, trainees work side-by-side at a community agency for 2.5 days/week. At the community agency, they participate in meetings with community members and agency leaders, work on a project that helps the agency and focuses on health advocacy, and learn practical advocacy skills demonstrated by their mentor and other agency staff members. Trainees are encouraged to focus on producing a tangible product, such as a white paper or summary of a particular topic to be used at or by the agency, op-eds, or patient materials. Several examples of past projects resulting from this 1-month elective at our institution are provided as a resource (Appendix H; full list available upon request).

#### Legislative Advocacy Day

During the final week, trainees prepare fact sheets for Legislative Advocacy Day, when they travel to legislative offices with the course director to discuss health issues and bills with state and federal legislators from both houses and all political parties. The educational coordinator identifies each trainee's legislators based on home address and calls legislative offices to schedule four to five meetings for this day. Trainees meet with the course director 1 week before Legislative Advocacy Day to prepare for the meetings and to plan and develop fact sheets. Trainees finalize fact sheets to advocate for health issues and bills that relate to their project or their work with the community site partner. There are many examples of quality fact sheets available on the internet. To help our trainees, we provide specific examples of fact sheets on our letterhead so that they have concrete examples. In addition, a formatted fact sheet provides a template that trainees are required to use during our rotation (see Appendix I for sample fact sheets on our letterhead). The course director fact-checks, edits, and finalizes each fact sheet and provides written and verbal feedback to each trainee. On the morning of Legislative Advocacy Day, trainees rehearse their fact sheets and role-play with one another under the supervision and advisement of the course director. Approximately 30 minutes per trainee is allotted for this rehearsal. The trainees and course director drive to local state and federal offices when the legislators are not in session and to capital city offices when the legislators are in session. Approximately 30 minutes is spent at each legislative office, and typically, five visits are scheduled throughout the day. To reinforce newly established relationships with policy makers, trainees sign provided thank-you notes for the legislative offices.

### Learner Assessment and Evaluations

At the beginning and end of the elective, trainees complete an advocacy assessment (Appendices K & L) so that the course director can identify their learning needs and assess the knowledge obtained from the course. The educational coordinator sends these assessments via an online survey tool so that the course director can easily access the results online. The trainee's faculty and community site mentors complete evaluations of the trainee at the end of the rotation (see Appendix M). The educational coordinator emails these evaluation templates to the mentors at the beginning of the elective and sends a reminder at the end of the month to have completed templates returned electronically.

Participants in the elective receive an evaluation template for each seminar speaker at the beginning of the rotation, and they are expected to return these electronically at the end of the elective (Appendix N). These are emailed back to the educational coordinator, who compiles the evaluations and sends deidentified feedback to each seminar speaker. Additionally, elective participants complete an evaluation of their community partner, which is similarly collected by the educational coordinator and provided to the course director (Appendix O). Based on trainee feedback, modifications should be made to alter involvement with some agencies. The elective experience is under continued review and evaluation. Changes are made to improve the experience based on this feedback; therefore, the materials provided with this summary are modifiable and should be tailored to individuals, locations, resources, and time. In addition to leading and presenting at an advocacy café, each trainee completes an end-of-course written report to describe what he or she has learned and accomplished during the rotation and to provide general written feedback (Appendix P). As with all clinical rotations, trainees receive a written final evaluation using an institution-specific form (i.e., standard student or resident evaluation form; our institution-specific form is available upon request). These forms are given to the clinical supervisors when they are persons other than the course director. Students are scored on a 5-point, Likert-like scale (1 = *Failure*, 5 = *High Honors)* for the following institution evaluation domains and subdomains:

• Professional behavior:
  ○ Integrity, compassion, altruism, interpersonal relationships, initiative/reliability, commitment to learning.
• Cognitive skills:
  ○ Fund of knowledge, synthesis and application of knowledge.
• Clinical skills:
  ○ Data gathering, physical examination, communication skills, patient education, technical/procedural skills, use and interpretation of diagnostic tests, oral presentations, documentation.
• Overall evaluation.

For residents, the evaluation domains are scored on a 9-point, Likert-like scale (1 = *Novice*, 9 = *Master*) and consist of patient care, medical knowledge, practice-based learning and improvement, interpersonal and communication skills, professionalism, and systems-based practice.

The content of the overall evaluation is based on the following information: an evaluation of the trainees' clinical performance by their clinic supervisors, trainees' performance in preparation for and their presentation skills during Legislative Advocacy Day, comments from seminar speakers and observations of the course director, written evaluation forms from faculty and community mentors, trainees' final report, and their final presentation. Based on the time spent in the respective areas, the final grades for students are broken down by the following percentages: community mentor evaluation (50%), clinical supervisor evaluation as noted above (30%), final report (10%), preparation for and performance during Legislative Advocacy Day (5%), and preparation, content, and delivery of the final presentation (5%).

## Results

The advocacy elective has resulted in a number of sustainable and noteworthy outcomes, including the following: (1) establishment of a refugee clinic in pediatrics; (2) inclusion of Wilmington, Delaware, as an additional site for the advocacy elective and as a city listed on the advocacy resource website, Cap4kids (www.cap4kids.org); (3) student presentations at national meetings (e.g., the Pediatric Academic Societies Meeting; see Appendix Q); (4) expansion of the elective to include visiting medical students and residents from outside programs; (5) strengthening of relationships with community organizations; and (6) publication of trainees' op-eds in local newspapers.

The office clinical practice now has a weekly refugee clinic on Wednesday mornings in partnership with a local refugee settlement agency called Nationalities Service Center. This was established after a pediatric resident partnered with Nationalities Service Center during her advocacy elective. The resident learned about similar refugee clinics at the district health centers in Philadelphia, Pennsylvania, and at the Jefferson Family Medicine practice and felt that an important area of advocacy was to make sure that refugee children had access to a medical home. After the resident discussed this with the practice medical director, faculty members were recruited to set up the clinic, which now runs weekly.

We added Wilmington as an additional site for the advocacy elective based on the interest and availability of a faculty member and community agencies there. In the past 7 years, two residents have elected to spend their clinical and community time with experts in Wilmington. With a growing interest in promoting advocacy in Wilmington, one of our chief residents worked to have the city and its community resources added to the Cap4kids website.

While the two outcomes above are specific to our location, student presentations, offering the elective to visitors, and strengthening relationships with community organizations are possible independent of location. Students and residents completing the elective are given the opportunity to submit their work as an abstract to a local or national meeting. Seven out of 53 trainees (13%) have had their work accepted and presented. In addition, two have published op-eds in major newspapers. The advocacy elective has been a positive experience for the trainees and the community agencies, which willingly continue to work with our students and residents without any financial incentive. Through word of mouth, we have residents and students contacting us from outside programs. To date, we have had four visiting students and residents (8%), and another three are scheduled for the 2016–2017 academic year.

The elective final report serves as a way for the course director to understand how time was spent at the community sites and to determine if learning objectives were met. It is also an opportunity for trainees to write critically about the elective and to provide written feedback. Below are selected comments based on feedback about the elective in the final report and through evaluation forms.

• “[The elective] truly opened my eyes to the importance of speaking up for our patients and the power that physicians can have in advocating for patients at the state and/or federal level.”
• “I was very inspired by everyone I met from the PA AAP [Pennsylvania Chapter of the AAP]. I learned how you can take issues you are passionate about and advocate for them while still having a career as a practicing physician.”
• “The seminars were my favorite part of the elective…. [They] were really informative and demonstrated advocacy in real time. Each one of the speakers had a story or an expertise to share. Some of the experiences shared are so moving, others are empowering.”
• “Thank you for an amazing experience in advocacy that provided me with the skills and courage to lobby on Capitol Hill with ACOG [the American Congress of Obstetricians and Gynecologists]. I am gently encouraging my peers to quit whining and voice their concerns to the people who really need to hear it—our members of Congress!”

At the end of the rotation, each trainee meets one-on-one with the course director to receive and provide feedback about the overall course experience, scheduling, educational coordinator and course director communication, seminars, and community partners not covered in the written evaluations. Based on rich participant feedback and annual review and discussion among program leadership, most major modifications to the elective were made in the first 3 years of implementation. Trainees noted that no time was allocated for the advocacy assessments; therefore, time at the start and end of the rotation is designated for these assessments. Faculty and community mentors did not always meet on a regular basis with trainees. Now, prior to the start of each rotation, the course director communicates by email and/or phone to remind mentors about their responsibilities. Trainees who spent time telecommuting and independently working on their projects from home submitted final reports that lacked substance and reported lower overall satisfaction; therefore, trainees must be at the community sites during the designated times. Ensuring that the community mentor is aware of this requirement and providing him or her with a schedule is important. At this time, the advocacy assessments are used strictly to identify learner needs at the start of the rotation and individual improvements in knowledge. Universally, all learners have reported improved skills and knowledge across domains. Due to the limited number of trainees taking the advocacy elective and the lack of a comparison group, aggregate analyses specific to the elective have not yet been conducted using the assessments. The advocacy assessments were used for the larger JeffSTARS curriculum for the other major component, in which advocacy components were added to a required outpatient rotation, impacting a larger number of trainees. Results from this aggregate look at the assessments demonstrate the potential use of these tools, and data have been presented as posters and published as abstracts at the Pediatric Academics Societies Meetings in 2011 and 2012 (abstracts available at http://www.abstracts2view.com/pasall/). Modifications to the curriculum, community partners, preceptors, and seminar speakers are made on an ongoing basis in response to trainee, faculty, and community partner feedback.

## Discussion

The advocacy elective, as part of the larger JeffSTARS advocacy curriculum, is now in its eighth year. On a practical level, the elective helps trainees learn about social determinants of health and policies as they apply to child health, introduces them to community organizations, and offers them practical advocacy skills that they can use in their clinical practice. Oftentimes, Legislative Advocacy Day is the first opportunity that trainees have had to meet with their local and federal legislators. After this experience, trainees realize that their expertise as health professionals is very valuable to legislators and health policy in general.

There are five essential elements that have helped the advocacy elective succeed. First, the elective has had the full support of academic deans and the pediatric clerkship director at the medical school, the division chief of general pediatrics, and the residency program director. Administrative support from a part-time program coordinator, whose responsibilities are to plan and facilitate all elective activities, has been essential. Initially grant funded, this coordinator is now funded by the General Pediatrics Division. This financial commitment is a tangible demonstration of both ongoing philosophical support and the value placed on the elective and the overall advocacy program. In addition, the course director, partially supported by the residency program, enhances and expands relationships with other departments, community partners, and legislative offices. Second, the advocacy elective has dedicated faculty and community mentors from many local organizations. Third, our institution is a forward-thinking environment that provides a culture that welcomes change. Fourth, a learner-centered curriculum helps trainees make the cultural shift needed to understand the relevance of advocacy in the context of their work. Fifth, the elective would not be possible without eager trainees who go beyond the status quo and believe in advocating for their patients and communities.

Teaching advocacy has raised awareness about social determinants of health, community resources, and the medical home as defined by the AAP.^17^ The elective allows self-selected trainees to gain a more in-depth knowledge of skills needed for community advocacy. It allows trainees to work on an advocacy project with a nonphysician team. Following this experience and Legislative Advocacy Day, trainees often note that what initially seemed to be intimidating actually turned out to be doable. One of the many benefits of the elective has been to strengthen the skills and expertise not only of the trainees but also of faculty members, allowing individuals to become effective advocates through the following three-stage process: (1) developing credibility in the community through community service and networking, (2) understanding the legislative process by becoming involved in attempting to pass legislation, and (3) working proactively as an expert in child health policy recognized by appropriate policy makers.^18^ Despite the elective's successes, we recognize a number of ongoing challenges, including the effort needed to keep the curriculum up to date and interesting and the need to maintain community relationships through clear and frequent communication.

## Appendices

A. CM1. Course Implementation at New Institution Checklist.docx
B. CM2. Elective Checklist.docx
C. CM3. Sample Schedule.docx
D. CM4. Seminar Topic List With Learning Objectives.docx
E. CM5. Syllabus Bibliography.docx
F. CM6. List of Community Partners.docx
G. CM7. Orientation for New Community Partner.docx
H. CM8. Selected Past Projects.docx
I. CM9. Sample Fact Sheets for Legislative Visits.docx
J. Seminar Materials folder
K. ET1. Advocacy Elective Assessment 1.pdf
L. ET2. Advocacy Elective Assessment 2.pdf
M. ET3. Trainee Evaluation by Community or Faculty Mentor.docx
N. ET4. Trainee Evaluation of Seminar.docx
O. ET5. Trainee Evaluation of Community Partner.docx
P. ET6. Final Report Template.docx
Q. Selected Trainee Abstracts and Presented Results folder All appendices are peer reviewed as integral parts of the Original Publication.
