# Supplementary material for: The JeffSTARS Advocacy and Community Partnership Elective: A Closer Look at Child Health Advocacy in Action
Source: MedEdPORTAL. 2016 Dec 31;12:10526. doi: 10.15766/mep_2374-8265.10526 (PMC6365684; doi:10.15766/mep_2374-8265.10526)
Supplement: Supplementary file 1 — A. CM1. Course Implementation at New Institution Checklist.docx B. CM2. Elective Checklist.docx C. CM3. Sample Schedule.docx D. CM4. Seminar Topic List With Learning Objectives.docx E. CM5. Syllabus Bibliography.docx F. CM6. List of Community Partners.docx G. CM7. Orientation for New Community Partner.docx H. CM8. Selected Past Projects.docx I. CM9. Sample Fact Sheets for Legislative Visits.docx J. Seminar Materials folder K. ET1. Advocacy Elective Assessment 1.pdf L. ET2. Advocacy Elective Assessment 2.pdf M. ET3. Trainee Evaluation by Community or Faculty Mentor.docx N. ET4. Trainee Evaluation of Seminar.docx O. ET5. Trainee Evaluation of Community Partner.docx P. ET6. Final Report Template.docx Q. Selected Trainee Abstracts and Presented Results folder [file mep-12-10526-s001.zip › A._CM1._Course_Implementation_at_New_Institution_Checklist.docx]

CM1. Course Implementation at New Institution Checklist

1. Six to twelve months prior to implementation (timelines will vary by institution), identify a faculty champion to lead this effort.
2. Review the following steps, and set a timeline for each of the steps.
3. Meet with co-faculty, including the division chief, independently or as a group to discuss starting a child health advocacy elective within the division home (e.g., the Division of General Pediatrics).
4. Meet with faculty from other divisions (e.g., Family and Community Medicine, Obstetrics and Gynecology, Internal Medicine, Emergency Medicine), independently or as a group, to determine if there is interest. Identify faculty liaisons in each division/department.
5. Develop an outline and course description following specifications of the medical school and residency program and based on guidelines provided in the Educational Summary Report (ESR) and the course materials.
6. Work with the division chief, the clinical clerkship director in Pediatrics, and the residency program director(s) to determine a strategy for implementation. Consider who has the skills and availability to direct the course; determine if support is available for an educational coordinator.
7. Seek input from the Dean of Academic Affairs.
8. Present the course to the relevant Curriculum Committee(s) for approval.
9. Determine if the course will be listed under Pediatrics or as an Interdisciplinary option.
10. Once there is institutional commitment to the course, seek input from students and residents, particularly Chief Residents, to determine if institution-specific modifications are needed.
11. Reach out to community partners and leaders and local advocacy experts to determine their interest and availability to help with seminars and mentoring. Generate a list of names, organizations, and contact information.
12. Identify local and national institutions with similar courses by searching on the internet, communicating with colleagues, and/or attending regional and national health advocacy meetings. Seek their input by meeting with leaders via phone, e-mail, and/or in-person (the preferred mode when feasible).
13. Prior to the start of the academic year, identify dates corresponding to 4^th^-year calendar blocks when the course will be offered.
14. Disseminate the course description and dates to the pediatrics clerkship coordinator, the clerkship director, faculty liaisons in other departments, the residency program coordinator(s), and the residency program director(s).
15. Conduct the course, modifying and building upon the provided ESR and Appendices.
