# Supplementary material for: The JeffSTARS Advocacy and Community Partnership Elective: A Closer Look at Child Health Advocacy in Action
Source: MedEdPORTAL. 2016 Dec 31;12:10526. doi: 10.15766/mep_2374-8265.10526 (PMC6365684; doi:10.15766/mep_2374-8265.10526)
Supplement: Supplementary file 1 — A. CM1. Course Implementation at New Institution Checklist.docx B. CM2. Elective Checklist.docx C. CM3. Sample Schedule.docx D. CM4. Seminar Topic List With Learning Objectives.docx E. CM5. Syllabus Bibliography.docx F. CM6. List of Community Partners.docx G. CM7. Orientation for New Community Partner.docx H. CM8. Selected Past Projects.docx I. CM9. Sample Fact Sheets for Legislative Visits.docx J. Seminar Materials folder K. ET1. Advocacy Elective Assessment 1.pdf L. ET2. Advocacy Elective Assessment 2.pdf M. ET3. Trainee Evaluation by Community or Faculty Mentor.docx N. ET4. Trainee Evaluation of Seminar.docx O. ET5. Trainee Evaluation of Community Partner.docx P. ET6. Final Report Template.docx Q. Selected Trainee Abstracts and Presented Results folder [file mep-12-10526-s001.zip › B._CM2._Elective_Checklist.docx]

CM2. Elective Checklist

Unless otherwise specified, tasks are completed by educational coordinator.

Prior to the course:

1. Prior to the start of the academic year, student registers for elective via the Registrar’s Office and the pediatric clerkship coordinator. Residents register via their program coordinator, who then contacts the pediatric clerkship coordinator.
2. Three months prior to the elective, the educational coordinator should reach out to registered Advocacy Elective trainees to confirm that they are taking the elective and to set up a time to meet in the next month with the course director.
3. Send a complete list of previous elective community projects for the trainee to review prior to the meeting with the course director.
4. Trainee meets in-person or by phone with course director who reviews the course objectives and requirements and discusses the interests of the trainee and potential community partners.
5. Follow-up with the trainee by e-mail or phone to ensure that s/he has several community partners to contact to explore project ideas and to determine the best fit. Keep consistent communication with trainees during the one to two months preceding the elective.
6. One-month prior to the elective, determine the final community partner site and mentor for each trainee. This may involve correspondence by the trainee with the course director and/or communication between the course director and the community site contact (see Orientation for New Community Partner).
7. Contact faculty mentor(s) and/or clinical supervisors (who are often the same person) depending on the number of registered trainees and their respective clinical interests.
8. Ask trainee to send their availability over the month and any possible absences (make-up time is required at some institutions when more than two days are missed)
9. Using the Sample Schedule template, create schedules specific to the trainee(s), determining if and when the trainee will be in clinic or away.
   1. Schedule three clinical sessions per week.
   2. Schedule two full days and one half-day per week as community site time.
   3. Establish a day for Legislative Advocacy Day, preferentially during the final week, based on the course director’s availability.
   4. Set times for weekly mentor meetings, and include Grand Rounds, Morning Reports, Advocacy Cafes, and other required sessions.
10. Two months prior to the elective, create seminar schedule. Send possible time slots to list of seminar speakers. The first seminar should be the introduction session with the course director.
11. Schedule seminar meeting locations.
12. Copy materials for course syllabus. Include reading materials and PowerPoint handouts for each seminar, when available.
13. Schedule Survey Monkey message that Advocacy Assessment 1 is due by the first day of elective block.
14. Send final schedule to trainee and their designated community mentor.
15. Set up a meeting between the course director and the community mentor to review objectives of the course and community partner responsibilities, if not completed in step 6 above.
16. Send out introduction via e-mail, providing all of the evaluation and report templates. Include final elective schedule and seminar schedule. Include Advocacy Cafe schedule and ACMGE Guidelines.

During the course:

Week 1

1. Meet with trainees at the start of the rotation to collect cell phone numbers/contact information, hand out course syllabus, and review final schedules.
2. Send e-mail reminders to all seminar speakers for upcoming seminars.
3. Make sure trainees have proper ID/EMR log-in for clinical sessions.
4. Course director to review learning needs from completed Advocacy Assessment 1.

Week 2

1. Check in with trainees to see how things are with the community site, the rotation in general.
2. Remind trainees to complete seminar evaluations.
3. Collect residential addresses for the trainees to determine their elected legislators.
4. Send e-mail to trainees with guidelines on how to prepare for Legislative Advocacy Day. Include sample factsheets.

Week 3

1. Contact offices of local and federal legislators. Aim to schedule 4-5 half-hour long meetings with legislative offices. Try to include a variety of offices (i.e., local, federal, Republican, Democratic, house, senate).
2. Send a reminder to trainees that factsheets and copies of bills to be discussed are due.
3. Send a reminder to trainees about the Advocacy Café presentation. Trainees will need to submit reading materials for attendees, and they will need to prepare PowerPoint presentations.

Week 4

1. Send final Legislative Advocacy Day schedule to trainees.
2. Send, in advance, legislative offices factsheets and bills that trainees will discuss.
3. Send trainees list of assignments that are due by the end of the rotation.
   1. Final Report
   2. Seminar Evaluations
   3. PowerPoint Presentation
   4. Evaluation of Community Site
4. Schedule a Survey Monkey message that Advocacy Assessment 2 is due by the last day.
5. Trainees send “thank you” notes to the seminar speakers and the legislative office.
6. Trainee(s) meet with course director for a feedback session.
7. Reminders are sent to Community Mentor to complete evaluation of trainee.
8. Give each trainee a “Physician Advocate” certificate (optional).

Following the elective:

1. After all assignments and evaluations are reviewed, course director to complete the final course evaluation.
2. Send thank you notes and provide feedback to seminar speakers and community sites.
