# Supplementary material for: The JeffSTARS Advocacy and Community Partnership Elective: A Closer Look at Child Health Advocacy in Action
Source: MedEdPORTAL. 2016 Dec 31;12:10526. doi: 10.15766/mep_2374-8265.10526 (PMC6365684; doi:10.15766/mep_2374-8265.10526)
Supplement: Supplementary file 1 — A. CM1. Course Implementation at New Institution Checklist.docx B. CM2. Elective Checklist.docx C. CM3. Sample Schedule.docx D. CM4. Seminar Topic List With Learning Objectives.docx E. CM5. Syllabus Bibliography.docx F. CM6. List of Community Partners.docx G. CM7. Orientation for New Community Partner.docx H. CM8. Selected Past Projects.docx I. CM9. Sample Fact Sheets for Legislative Visits.docx J. Seminar Materials folder K. ET1. Advocacy Elective Assessment 1.pdf L. ET2. Advocacy Elective Assessment 2.pdf M. ET3. Trainee Evaluation by Community or Faculty Mentor.docx N. ET4. Trainee Evaluation of Seminar.docx O. ET5. Trainee Evaluation of Community Partner.docx P. ET6. Final Report Template.docx Q. Selected Trainee Abstracts and Presented Results folder [file mep-12-10526-s001.zip › C._CM3._Sample_Schedule.docx]

| **The JeffSTARS Curriculum – Advocacy Elective**  **CM3. Advocacy Elective Sample Schedule** | | | | | |
| --- | --- | --- | --- | --- | --- |
|  | **MON (Date)** | **TUES (Date)** | **WED (Date)** | **THURS (Date)** | **FRI (Date)** |
| Week 1 | 8am-9am Advocacy Assessment 1 | 9am-5pm  Community Site | 8am-9am  Grand Rounds | 8am-9 am  Morning Report | 8am-9am  Mentor Meeting |
|  | 9am-12pm  Patient Care |  | 9am-12pm  Patient Care | 9am-12pm  Patient Care | 9am-12pm  Patient Care |
|  | 12pm-1pm  Seminar #1 |  |  |  | 12:30pm-2pm  Advocacy Cafe |
|  | 1pm-5pm  Community Site |  |  |  |  |
|  |  |  | 1pm-2 pm Seminar #2 | 1pm-5 pm  Community Site | 2pm-5pm  Community Project Time |
|  |  |  | 2pm-3 pm  Seminar #3 |  |  |
|  |  |  | 3pm-4 pm  Clinical Case Conference |  |  |
|  | **MON (Date)** | **TUES (Date)** | **WED (Date)** | **THURS (Date)** | **FRI (Date)** |
| Week 2 | 9am-12pm  Patient Care | 8am-9am  Seminar #5 | 8am-9am  Grand Rounds | 8am-9am  Morning Report | 8am-9am  Mentor Meeting |
|  |  | 9am-5pm  Community Site | 9am-5pm  Community Site | 9am-12pm  Patient Care | 9am-12pm  Patient Care |
|  | 12pm-1pm  Seminar #4 |  |  |  | 12:30pm-2:00pm Advocacy Cafe |
|  | 1pm-5pm  Community Site |  |  | 12pm-1pm  Seminar #6 | 2pm-3pm  Seminar #7 |
|  |  |  |  | 1pm-5pm  Community Site | 3:15pm-5:00pm  Legislative Advocacy Day Prep |

Clinical Didactic Reflective and Sharing Community Engagement

Components of Required Outpatient Rotation

| **JeffSTARS Advocacy Elective** | | | | | |
| --- | --- | --- | --- | --- | --- |
|  | **MON (Date)** | **TUES (Date)** | **WED (Date)** | **THURS (Date)** | **FRI (Date)** |
| Week 3 | 9am-12pm  Patient Care | 9am-10am  Seminar #9 | 8am-5pm  Legislative Advocacy Day | 8am-9am  Morning Report | 8am-9am  Mentor Meeting |
|  |  | 10am-5pm  Community Site |  | 9am-12pm  Patient Care | 9am-12pm  Patient Care |
|  | 12pm-1pm  Seminar #8 |  |  |  |  |
|  | 1pm-5pm  Community Site |  |  |  | 12:30pm-2:00pm Advocacy Cafe |
|  |  |  |  | 1pm-2pm  Seminar #10 | 2pm-3pm  Seminar #13 |
|  |  |  |  | 2pm-3pm  Seminar #11 | 3pm-4pm  Seminar #14 |
|  |  |  |  | 3pm-4pm  Seminar #12 |  |
|  | **MON (Date)** | **TUES (Date)** | **WED (Date)** | **THURS (Date)** | **FRI (Date)** |
| **Week 4** | 9am-12pm  Patient Care | 8am-9am Advocacy Journal Club | 8am-9am  Grand Rounds | 8am-9am  Morning Report | 8am-9am  Mentor Meeting |
|  |  | 9am-5pm  Community Site | 9am-5pm  Community Site | 9am-12pm  Patient Care | 9am-12pm  Patient Care |
|  | 12pm-1pm  Seminar #15 |  |  |  | 12:30pm-2:00pm  Advocacy Café  Advocacy Elective Presentations |
|  | 1pm-4pm  Community Project Time |  |  | 12pm-5pm  Community Site |  |
|  |  |  |  |  | 2:00pm-4:00pm  Advocacy Assessment 2 & Wrap Up |

Clinical Didactic Reflective and Sharing Community Engagement

Components of Required Outpatient Rotation
