# Supplementary material for: The JeffSTARS Advocacy and Community Partnership Elective: A Closer Look at Child Health Advocacy in Action
Source: MedEdPORTAL. 2016 Dec 31;12:10526. doi: 10.15766/mep_2374-8265.10526 (PMC6365684; doi:10.15766/mep_2374-8265.10526)
Supplement: Supplementary file 1 — A. CM1. Course Implementation at New Institution Checklist.docx B. CM2. Elective Checklist.docx C. CM3. Sample Schedule.docx D. CM4. Seminar Topic List With Learning Objectives.docx E. CM5. Syllabus Bibliography.docx F. CM6. List of Community Partners.docx G. CM7. Orientation for New Community Partner.docx H. CM8. Selected Past Projects.docx I. CM9. Sample Fact Sheets for Legislative Visits.docx J. Seminar Materials folder K. ET1. Advocacy Elective Assessment 1.pdf L. ET2. Advocacy Elective Assessment 2.pdf M. ET3. Trainee Evaluation by Community or Faculty Mentor.docx N. ET4. Trainee Evaluation of Seminar.docx O. ET5. Trainee Evaluation of Community Partner.docx P. ET6. Final Report Template.docx Q. Selected Trainee Abstracts and Presented Results folder [file mep-12-10526-s001.zip › D._CM4._Seminar_Topic_List_With_Learning_Objectives.docx]

**The JeffSTARS Curriculum – Advocacy Elective**

**CM4. Seminar Topic List with Learning Objectives**

1. **Overview – Introduction to Health Advocacy; Working with Your Community Partner**
   1. State the reasons why physicians are well-positioned to advocate for health
   2. Describe the socio-ecological model and discuss its impact on health behaviors
   3. Discuss various stakeholders involved in maintaining health in the United States

*The Course Director/Physician with Advocacy Experience leads this seminar, introducing learners to advocacy concepts, stakeholders involved in advocacy, and strengths and weaknesses of physicians entering the world of advocacy.*

**For slide presentation, see Appendix J.*

1. **The Roadmap to Successful Advocacy: Essentials to Being An Effective Change Agent**
   1. Understand that change requires knowing stakeholders and building relationships with community partners
   2. Gain skills to be an effective change agent
   3. Know that strategy is an important aspect of health advocacy

*A Professor of Obstetrics and Gynecology/Community Leader in Advocacy/Physician or Lawyer with Advocacy Experience leads this session by providing an example of past advocacy work, the associated stakeholders and the importance of building relationships. The leader points out that unlike clinical care, advocacy often involves stakeholders with differing perspectives, timelines and agendas. Skills for being effective and developing strategies are discussed.*

*No slide presentation is used for this seminar.*

1. **In-depth Interviews to Understand Social Factors Affecting Your Patient’s Health**
   1. Gain skills to screen for social determinants of health
   2. Improve patient descriptions to minimize judgment while maximizing information to help improve health
   3. Understand the essential role social factors have on health

*The Course Director/Faculty Physician leads this seminar on the importance of social factors in medical care. The seminar starts with the Social History Exercise (see Appendix C) and ends with a discussion. In this seminar, we provide tips to obtain and document a useful and accurate social history, and we review common errors made by physicians.*

**For slide presentation, see Appendix J.*

1. **Voting with Children’s Health and Trainees’ Schedules in Mind/Incorporating Advocacy into Your Future**
   1. Understand how policy makers are elected into office and the importance of voting
   2. Describe examples of how advocacy can be incorporated into various stages of a physician’s career
   3. Gain skills to describe health problems in non-partisan ways

*The Executive Director of Pennsylvania Chapter AAP/Pediatrician Experienced in Advocacy leads this seminar as a small group discussion focused on individual and organizational advocacy.  Using case examples of Pennsylvania pediatricians, the seminar leader illustrates the six opportunities for advocacy work through the chapter (legislation, regulation, education, media, administration and coalitions). The seminar leader encourages medical students and residents to get engaged in ways that work with their tight schedules and, more importantly, discusses the credibility, value and opportunity available by incorporating advocacy throughout their professional lives. No slide presentation is used for this seminar.*

1. **Community Engagement, Organizing and Relationship Building**
   1. Identify potential allies and opponents in an advocacy activity
   2. Appreciate the importance of being well informed but also having appropriate humility and tolerance when approaching allies or potential opponents
   3. Understand the need for patience

*Former Chair of the Department of Pediatrics/Physician/Lawyer/Community Leader/with Experience in Advocacy leads this advocacy session on mobilizing community advocacy resources, utilizing the case of a small state getting legislation passed to assure children with certain inborn errors of metabolism have access to specialized diet. In the session, we review the efforts made to provide special diet and outline the steps taken (over 15 years) to get legislation passed and signed.  Specifically we discuss a proposed (and now established) law requiring insurance companies to cover the specialized formula. We stress the need for patience, for polite persistence, for establishing collaboration and networks, and for utilizing various individuals (families of affected children) and agencies (hospitals, disease support groups, professional organizations etc.) in doing advocacy at the state level.*

*No slide presentation is used for this seminar.*

1. **Maternal Child Health from a Global Perspective**
   1. Recognize America’s commitment to global health
   2. Describe the importance of women’s health to individuals, families, and communities
   3. Describe the determinants of women’s and children’s health and how they vary in different settings
   4. Discuss the burden of disease for women worldwide, with a focus on women in low- and middle-income countries

*A Professor of Family and Community and Global Medicine/Physician with Global Health Experience leads this seminar as a small group discussion. The seminar leader discusses America’s infrastructure supporting Global Health, and the multiple factors affecting maternal child health. The leader uses examples from professional experiences working in Uganda and Rwanda, and provides a forum to explore global health work. The leader involves visiting medical students from Rwanda in the discussion, and their country’s approach to maternal/child health.*

**For slide presentation, see Appendix J.*

1. **How Media Can Impact Health Advocacy**
   1. Describe various media modalities involved in the dissemination of health information
   2. Identify ways in which the media can positively and negatively influence health behaviors and beliefs
   3. Gain skills to work with the media as a physician advocate

*The Course Director/Faculty Physician with Media Experience leads this seminar on the influence of the media on health. The seminar starts with a brief discussion about the learners’ notions about reporters and the media, and important recent examples of how the media has impacted health. In this seminar, we discuss professional experience with the media including mistakes along the way, and we discuss skills necessary for working with the verbal and written media.*

**For slide presentation, see Appendix J.*

1. **Advocacy Communication/Crafting the Message for Effective Advocacy**
   1. Gain skills in knowing how to craft and effective advocacy message
   2. Understand how physicians communicate with patients
   3. Learn how to effectively communicate with community agencies to promote health

*An Assistant Professor in the College of Population Health/Lawyer with Expertise in Advocacy and Health Policy and is actively engaged with the American Public Health Association's policy and advocacy initiatives leads this advocacy session. In this seminar different scenarios and exemplary advocacy materials (e.g., fact sheets, infographics, op-ed pieces and letters to the editor) are discussed. In the session, we review a systematic approach for conceptualizing, developing, implementing and evaluating an advocacy initiative. Specifically, we review the US legislative structure and processes to guide the selection of timely issues and appropriate policy makers. We discuss the need for thorough and objective preparation, professional and attentive presentation, and honest evaluation, re-assessment and follow-up.*

*No slide presentation is used for this seminar.*

1. **Working with Decision Makers/ Understanding Advocacy Strategy**
   1. Understand the US legal structure and procedures to guide governmental advocacy initiatives
   2. Conduct background ('due diligence') research about identified policy maker(s), (e.g., determine political affiliation, committee appointments, priority issues, past voting records to inform policy positions, options and framing the issue(s))
   3. Assess the political, economic and social landscapes when considering advocacy strategies

*A Community Leader/Physician or Lawyer with Policy Making Experience leads this seminar. During this session, the seminar leader provides a case example of his/her experience with working with policy makers to advocate for a health-related change. Learners discuss the influence of politics, economics, and society on policy development and change.*

*No slide presentation is used for this seminar.*

1. **Working in Partnerships/Multidisciplinary Collaboration and Stakeholders**
   1. Understand the various agendas and demands among stakeholders
   2. Gain skills to identify common ground with various community agencies
   3. Learn to delegate advocacy activities building on the strengths of various stakeholders and community agencies

*A Professor of Family and Community Medicine-Non-Physician/Public Health Expert/Community Leader in Advocacy leads this seminar as a small group discussion. She discusses her past experiences in working with community based organizations and coalitions in addressing health and social issues that impact communities.  The discussion includes identifying stakeholders, how to actively engage stakeholders so that desired shared outcomes can be developed through visioning and decision making processes, and how to evaluate efforts and share results in ways that help to sustain partner engagement overtime so that desired results are achieved. Students are asked to share their experiences in collaborative activities including what worked, what didn’t and how they would modify their approach.*

*No slide presentation is used for this seminar.*

1. **Identifying the Problems: Discrimination and Structural and Institutional Barriers to Health**
   1. Review importance of special education accommodations for students with psychiatric conditions.
   2. Review legal mandates of IDEA for providing a “free and appropriate education” for all public school students with established special education needs.
   3. Using the School District of Philadelphia as a local example, describe and discuss structural and institutional barriers that lead to discrimination towards disadvantaged students and their families in obtaining appropriate special education accommodations.
   4. Discuss and describe some advocacy approaches that physicians can take to address institutional barriers to special education accommodations for students.

*A Faculty Director of Child and Adolescent Psychiatry/Experienced Mental Health Provider leads this seminar to help learners understand the role of discrimination and structural and institutional barriers to health. *For slide presentation, see Appendix J.*

1. **Advocacy in the Office Setting**
   1. Understand the role of advocacy in the outpatient setting
   2. Describe the social determinants of health and provide examples of resources in the community to help families in need.
   3. Describe how early life experiences affect short- and long-term health

*An Associate Professor of Pediatrics from Drexel University/Primary Care Pediatrician with Advocacy Experience leads this seminar. In this seminar, screening for and addressing social determinants of health are covered. There is special emphasis on early learning, the harmful effects of stress and adverse childhood experiences (ACEs), and poverty.*

**For slide presentation, see Appendix J.*

1. **Refugee and Immigrant Health – Issues and Challenges**
   1. Define and discuss determinants of migrant health
   2. Provide a case example of research-based advocacy in refugee and immigrant populations in [location/city]
   3. Discuss how to become a physician advocate in the process of political asylum for unaccompanied minors coming into the United States from other countries

*A Faculty Member with Clinical Experience in Caring for Refugees and Immigrants leads this seminar. Distinctions are made between refugees and immigrants, and US policies are discussed.*

**For slide presentation, see Appendix J.*

1. **Framing a Health Advocacy Campaign Around Current Issues**
   1. Describe several examples of active health issues where advocacy efforts are needed
   2. Understand the essential elements of a health advocacy campaign
   3. Gain skills to stay current and to impact policy related issues

*A Clinical Professor of Medicine at the University of Pennsylvania and a former adjunct at the School of Public Health at Drexel University/Community Health Leader/Public Health Leader with Advocacy Experience leads this seminar.*

*No slide presentation is used for this seminar.*

1. **Research & Advocacy**
   1. Describe how medical research can be used to advocate for health issues
   2. Provide examples of research studies in medicine that have influenced health care and health policy
   3. List areas where medical research is needed to advocate for health

*A Physician or Non-Physician Researcher with Expertise in Social Determinants of Health, Poverty, or Topic where Advocacy has a Role may lead this seminar. In this seminar, the leader explains how research is one way in which physicians can be health advocates*

*No slide presentation is used for this seminar; however, it could be helpful for individual researchers to bring slides that demonstrate the scope and impact of their research.*
