# Supplementary material for: The JeffSTARS Advocacy and Community Partnership Elective: A Closer Look at Child Health Advocacy in Action
Source: MedEdPORTAL. 2016 Dec 31;12:10526. doi: 10.15766/mep_2374-8265.10526 (PMC6365684; doi:10.15766/mep_2374-8265.10526)
Supplement: Supplementary file 1 — A. CM1. Course Implementation at New Institution Checklist.docx B. CM2. Elective Checklist.docx C. CM3. Sample Schedule.docx D. CM4. Seminar Topic List With Learning Objectives.docx E. CM5. Syllabus Bibliography.docx F. CM6. List of Community Partners.docx G. CM7. Orientation for New Community Partner.docx H. CM8. Selected Past Projects.docx I. CM9. Sample Fact Sheets for Legislative Visits.docx J. Seminar Materials folder K. ET1. Advocacy Elective Assessment 1.pdf L. ET2. Advocacy Elective Assessment 2.pdf M. ET3. Trainee Evaluation by Community or Faculty Mentor.docx N. ET4. Trainee Evaluation of Seminar.docx O. ET5. Trainee Evaluation of Community Partner.docx P. ET6. Final Report Template.docx Q. Selected Trainee Abstracts and Presented Results folder [file mep-12-10526-s001.zip › E._CM5._Syllabus_Bibliography.docx]

**CM5. Syllabus Materials (Articles Organized by Seminar Title)**

**1. Overview – Introduction to Health Advocacy; Working with Your Community Partner**

Berman S. Training Pediatricians to Become Child Advocates. *Pediatrics.* 1998; 102 (3): 632-635.

Stone JR. Saving and ignoring lives: physicians' obligations to address root social influences on health--moral justifications and educational implications. *Cambridge Quarterly of Healthcare Ethics: The International Journal of Healthcare Ethics Committees.* 2010;19(4):497-509.

**2. The Roadmap to Successful Advocacy: Essentials to Being An Effective Change Agent**

Berwick DM. A primer on leading the improvement of systems. *BMJ (Clinical research ed.).* 1996;312(7031):619-622.

Conner RL. The Advocacy Project Discussion Paper Series No. 09-1. Resistance: A Primer for Advocates and Change Agents. <http://poseidon01.ssrn.com/delivery.php?ID=335074111074012070110100123094101073109017024072035030106078024111120083009067093107037033035060058099112006011109026103112099062066043049061126115118006092106023029066031065121125090100085114067106098020083022021106123116072065064001110007027125009&EXT=pdf>. Accessed June 14, 2016.

Gruen RL, Pearson, SD, Brenna TA. Physician-Citizens Public Roles and Professional Obligations. *JAMA* 2004; 94-98.

Satcher D, Kaczorowski J, Topa D. The expanding role of the pediatrician in improving child health in the 21st century. *Pediatrics.* 2005;115(4 Suppl):1124-1128.

**3. In-depth Interviews to Understand Social Factors Affecting Your Patient’s Health**

Braveman P, Egerter S, Barclay C. What shapes health-related behaviors? The role of social factors. Robert Wood Johnson Foundation. <http://www.rwjf.org/en/library/research/2011/03/what-shapes-health-related-behaviors---.html> Accessed June 14, 2016.

Chung EK, Siegel B, Garg A, et al. Screening for Social Determinants of Health among Children and Families Living in Poverty: A Guide for Clinicians. *Curr Probl PediatrAdolesc Health Care.* 2016;46:135-153.

Williams DR, Costa MV, Odunlami AO, Mohammed SA. Moving upstream: how interventions that address the social determinants of health can improve health and reduce disparities. *Journal of Public Health Management and Practice : JPHMP*. 2008;14 Suppl:S8-17.

See end of this document for Supplemental Material – Social History Exercise.

**4. Voting with Children’s Health and Trainees’ Schedules in Mind/Incorporating Advocacy into Your Future**

AAP Advocacy Guide. Chapter 4: Core Advocacy Skills. <http://www.mdaap.org/AdvocacyGuide.pdf> Accessed June 14, 2016.

AAP Advocacy Guide. Chapter 5: We’re Not Alone – Broadening Participation. <http://www.mdaap.org/AdvocacyGuide.pdf> Accessed June 14, 2016.

**5. Community Engagement, Organizing and Relationship Building**

Israel BA, Coombe CM, Cheezum RR, et al. Community-Based Participatory Research: A Capacity-Building Approach for Policy Advocacy Aimed at Eliminating Health Disparities. *American Journal of Public Health.* 2010; 100(11): 2094-2102.

Ochoa ER, Nash C. Community Engagement and its Impact on Child Health Disparities: Building Blocks, Examples, and Resources. *Pediatrics.* 2009; 124(3): S237-S45.

**6. Maternal Child Health from a Global Perspective**

Bhutta ZA, Chopra M, Axelson H, et al. Countdown to 2015 decade report (2000-10): taking stock of maternal, newborn, and child survival. *Lancet.* 2010; 375: 2032-2044.

Roseman MJ, Reichenbach L. International conference on population and development at 15 years: Achieving sexual and reproductive health and rights for all? *American Journal of Public Health.* 2010; 100(3): 403-406.

Shiffman J. Issue attention in global health: the case of newborn survival. *Lancet.* 2010; 375: 2045-2049.

Unsafe abortions: eight maternal deaths every hour. *Lancet.* 2009; 374: 1301.

**7. How Media Can Impact Health Advocacy**

Planning for Media Advocacy. The Praxis Project. <http://www.unnaturalcauses.org/assets/uploads/file/UC_MediaAdvocacy.pdf> Accessed July 7, 2016

**8. Advocacy Communication/Crafting the Message for Effective Advocacy**

**American Public Health Association.** APHA Legislative Advocacy Handbook: A Guide for Effective Public Health Advocacy. <http://www.kpha.us/resources/Documents/2015_Documents/APHA%20Legislative%20Advocacy%20Handbook1.pdf>**. Accessed April 3, 2016.**

**9. Working with Decision Makers/ Understanding Advocacy Strategy**

Hall R. Fifteen lessons in practical politics. *Journal of Pediatric Health Care.* 2000; 14(1): 38-40.

Hall R. Six painful lessons of politics*. Journal of Pediatric Health Care*. 2001; 15(6): 319-321.

**10. Working in Partnerships/Multidisciplinary Collaboration and Stakeholders**

Torres GW, Margolin FS. The collaboration primer: proven strategies, considerations, and tools to get you started. <http://www.hret.org/upload/resources/collaboration-primer.pdf> Accessed July 7, 2016

**11. Identifying the Problems: Discrimination and Structural and Institutional Barriers to Health**

Knitzer J, Cooper J. Beyond integration: challenges for children’s mental health. *Health Affairs.* 2006; 25(3): 670-679.

Luebbert JF, Malone RP, Riser L. Disability law and the administration of psychotropic medication in the school setting. *Psychiatric Services.* 2000; 51(11): 1369-1370.

**12. Advocacy in the Office Setting**

Taylor DR, Maniar P. The Children’s Advocacy Project of Philadelphia’s Cap4Kids Survey: An Innovative Tool for Pediatrician--Community-Based Organization Collaboration. *Clinical Pediatrics.* 2007; 46(6): 512-517.

**13. Refugee and Immigrant Health – Issues and Challenges**

Adams KM, Gardiner LD, Assefi N. Healthcare challenges from the developing world: post-immigration refugee medicine. *BMJ.* 2004; 328: 1548-1552.

Flanagin J. On the Border, Who Gets to Be a Refugee? *The New York Times.* 2014.

Guterres A, Spiegel P. The State of the World’s Refugees: Adapting Health Responses to Urban Environments. *JAMA.* 2012; 308(7): 673-674.

Kett M. Displaced populations and long term humanitarian assistance. *BMJ.* 2005; 331: 98-100.

Muller D. Haiti. *JAMA.* 2011; 305(5): 447-448.

Nazario S. The Children of Drug Wars. *The New York Times.* 2014.

**14. Framing a Health Advocacy Campaign around Current Issues**

Chapman S. Reflections on a 38- year career in public health advocacy: 10 pieces of advice to early career researchers and advocates. *Public Health Res Pract*. 2015;25(2):e2521514.

See end of this document for Supplemental Material.

**15. Research & Advocacy**

Christoffel KK. Public Health Advocacy: Process and Product. *American Journal of Public Health*. 2000; 90(5): 722-726.

SUPPLEMENTAL MATERIAL

**In-depth Interviews to Understand Social Factors Affecting Your Patient’s Health**

Social History Exercise:

Another Look at the Pediatric History: How to Be a Better Advocate

[Faculty Name]

[Institution Name]

Case: JC is a 6 yo African American boy with ADHD, speech delay and behavior problems who lives in foster care after neglect by his 21 yo mother who was also noncompliant. The foster mother tells you that he may be reunited with his biological mother soon. This patient is new to your practice.

1. Circle words that could lead to negative assumptions about this patient and his family, and write down substitutions that might be less presumptive or judgmental.

1. List questions that might help you better understand this family’s situation and allow you to better serve this patient.
2. If this were your foster child, list some expectations that you might have of the doctor at this visit. How do these compare to the expectations you have as a doctor?

SUPPLEMENTAL MATERIAL

**Framing a Health Advocacy Campaign around Current Issues**

**Assignment prior to seminar:**

From any news source you choose - preferably written (as opposed to radio or TV, unless we can access the feed) - pick one news issue from this past week that you think has a health/public health aspect to it, and that is a potential advocacy campaign. Feel free to have as broad a definition of health as you wish, as long as you can defend it. Please bring the article (real newsprint if you wish, a printout is fine, or your laptop is fine, as long as you have the text). A clinical situation that you think is in need of systemic advocacy is an acceptable alternative.

Consider:

1) What is the health issue?

2) What needs changing?

3) What would be your goal?

4) Who can give it to you?

5) What tactics would you use to change the position of the answer to #4?

6) Who might be your allies?

Read the two articles:

1. The article by Chapman – see Syllabus
2. The *NY Times* article available at this link: <http://nyti.ms/1dVuN96>

**Additional reading that you might find of interest:**

Almost anything on the website of the Berkeley Media Studies Group, bmsg.org – they have a great “handbook”. See:

<http://bmsg.org/sites/default/files/bmsg_handbook_working_upstream.pdf>

Dorfman L. Studying the news on public health: how content analysis supports media advocacy. *American journal of health behavior.* 2003;27 Suppl 3:S217-226.

Dorfman L, Krasnow ID. Public health and media advocacy. *Annual Review of Public Health.* 2014;35:293-306.

Christoffel KK. Public health advocacy: process and product. *American Journal of Public Health.* 2000;90(5):722-726. (see Syllabus under “Research and Advocacy”)

Frieden TR. A framework for public health action: the health impact pyramid. *American Journal of Public Health.* 2010;100(4):590-595.
