# Supplementary material for: The JeffSTARS Advocacy and Community Partnership Elective: A Closer Look at Child Health Advocacy in Action
Source: MedEdPORTAL. 2016 Dec 31;12:10526. doi: 10.15766/mep_2374-8265.10526 (PMC6365684; doi:10.15766/mep_2374-8265.10526)
Supplement: Supplementary file 1 — A. CM1. Course Implementation at New Institution Checklist.docx B. CM2. Elective Checklist.docx C. CM3. Sample Schedule.docx D. CM4. Seminar Topic List With Learning Objectives.docx E. CM5. Syllabus Bibliography.docx F. CM6. List of Community Partners.docx G. CM7. Orientation for New Community Partner.docx H. CM8. Selected Past Projects.docx I. CM9. Sample Fact Sheets for Legislative Visits.docx J. Seminar Materials folder K. ET1. Advocacy Elective Assessment 1.pdf L. ET2. Advocacy Elective Assessment 2.pdf M. ET3. Trainee Evaluation by Community or Faculty Mentor.docx N. ET4. Trainee Evaluation of Seminar.docx O. ET5. Trainee Evaluation of Community Partner.docx P. ET6. Final Report Template.docx Q. Selected Trainee Abstracts and Presented Results folder [file mep-12-10526-s001.zip › F._CM6._List_of_Community_Partners.docx]

**The JeffSTARS Curriculum – Advocacy Elective**

**CM6. Suggested List of Community Partners for Trainee Community Experience and Project**

1. State chapter of the American Academy of Pediatrics or other professional organization
2. Advocacy organization aimed at access to prenatal care and early childhood
3. Environmental advocacy group
4. Community legal agency addressing legal matters related to health insurance
5. Community legal agency addressing public programs for early learning and public education for older children
6. Community legal agency addressing women’s reproductive rights
7. Community organization that provides clinical care and support services at home for children and families
8. Nonprofit organization with goal to improve maternal and child health and wellbeing
9. Local treatment center that provides woman-centered treatment services for pregnant/postpartum women
10. Local Special Supplemental Nutrition Program for Women, Infants and Children (WIC) program
11. Local Chapter of Physicians for Social Responsibility or other physician advocacy group
12. Community organization dedicated to ensuring access to healthy food and reducing food insecurity
13. Local or state government health department
14. Local or state government human services or welfare department
15. Advocacy organization aimed at addressing issues that affect children with special healthcare needs
16. Community immigrant and refugee service organization
17. Community agency addressing lesbian, gay, bisexual, transgender, and queer/questioning (LGBTQ) health issues
