# Supplementary material for: The JeffSTARS Advocacy and Community Partnership Elective: A Closer Look at Child Health Advocacy in Action
Source: MedEdPORTAL. 2016 Dec 31;12:10526. doi: 10.15766/mep_2374-8265.10526 (PMC6365684; doi:10.15766/mep_2374-8265.10526)
Supplement: Supplementary file 1 — A. CM1. Course Implementation at New Institution Checklist.docx B. CM2. Elective Checklist.docx C. CM3. Sample Schedule.docx D. CM4. Seminar Topic List With Learning Objectives.docx E. CM5. Syllabus Bibliography.docx F. CM6. List of Community Partners.docx G. CM7. Orientation for New Community Partner.docx H. CM8. Selected Past Projects.docx I. CM9. Sample Fact Sheets for Legislative Visits.docx J. Seminar Materials folder K. ET1. Advocacy Elective Assessment 1.pdf L. ET2. Advocacy Elective Assessment 2.pdf M. ET3. Trainee Evaluation by Community or Faculty Mentor.docx N. ET4. Trainee Evaluation of Seminar.docx O. ET5. Trainee Evaluation of Community Partner.docx P. ET6. Final Report Template.docx Q. Selected Trainee Abstracts and Presented Results folder [file mep-12-10526-s001.zip › G._CM7._Orientation_for_New_Community_Partner.docx]

CM7. Orientation for New Community Partner and/or Community Mentor

Step 1. Course Director, other faculty or trainee identifies a potential Community Partner for the JeffSTARS Advocacy Elective, based on the following criteria:

- 1. The organization is an established community organization (i.e., non-profit, public health, governmental) with at least 3 years experience working in health advocacy with local families/clients and other organizations to improve the overall health and welfare of children and families.
  2. The organization has experience in community engagement and/or legislative advocacy.
  3. The organization has a physical office, where business is conducted and where a trainee can spend 2.5 days/week x 4 weeks learning about the daily activities of the organization.
  4. There is a designated non-clinical, community site mentor, who has a supervisory role in the organization and is available in-person to mentor and guide the trainee throughout the timeframe of the elective.

Step 2. The Educational Coordinator e-mails the Executive Director or other leader at the potential Community Partner with a brief introduction about the JeffSTARS Advocacy Elective, attaching a list of past projects and providing potential dates and times to schedule a meeting with the Course Director.

Step 3. Course Director meets by phone or in-person with the Community Mentor to discuss the course objectives, provide a rough overview of the schedule, discuss the requirement for a mutually agreeable project that can realistically be addressed in a 4-week period, and answer any questions that the site mentor may have.

(For established partners, Step 3 is may occur via e-mail as a reminder and to see if there are any questions or concerns.)
