# Supplementary material for: The JeffSTARS Advocacy and Community Partnership Elective: A Closer Look at Child Health Advocacy in Action
Source: MedEdPORTAL. 2016 Dec 31;12:10526. doi: 10.15766/mep_2374-8265.10526 (PMC6365684; doi:10.15766/mep_2374-8265.10526)
Supplement: Supplementary file 1 — A. CM1. Course Implementation at New Institution Checklist.docx B. CM2. Elective Checklist.docx C. CM3. Sample Schedule.docx D. CM4. Seminar Topic List With Learning Objectives.docx E. CM5. Syllabus Bibliography.docx F. CM6. List of Community Partners.docx G. CM7. Orientation for New Community Partner.docx H. CM8. Selected Past Projects.docx I. CM9. Sample Fact Sheets for Legislative Visits.docx J. Seminar Materials folder K. ET1. Advocacy Elective Assessment 1.pdf L. ET2. Advocacy Elective Assessment 2.pdf M. ET3. Trainee Evaluation by Community or Faculty Mentor.docx N. ET4. Trainee Evaluation of Seminar.docx O. ET5. Trainee Evaluation of Community Partner.docx P. ET6. Final Report Template.docx Q. Selected Trainee Abstracts and Presented Results folder [file mep-12-10526-s001.zip › H._CM8._Selected_Past_Projects.docx]

**The JeffSTARS Curriculum – Advocacy Elective**

**CM8. Selected Past Projects**

| **Advocacy Elective Selected Projects** | | |
| --- | --- | --- |
| **Community Partner** | **Description of Organization** | **Project Overview** |
| **Nationalities Service Center (NSC)**  [**http://www.nationalitiesservice.org/**](http://www.nationalitiesservice.org/) | A non-profit organization that provides social, educational, and legal services to immigrants and refugees in the Greater Philadelphia area. Since NSC’s founding in 1921, their mission has been to help immigrants and refugees participate fully in American society. | Pediatric third-year resident worked with NSC to help refugee families transition to America. She reviewed access to health care for these families, and found that care is limited to the city District Health Centers and a hospital-based Family and Community Medicine clinic. She helped to start the weekly Refugee Clinic at Jefferson Pediatrics/Nemours – Philadelphia. |
| **The Food Trust** [**http://thefoodtrust.org**](http://thefoodtrust.org) | **A non-profit organization whose mission is to ensure that everyone has access to affordable, nutritious food and information to make healthy decisions**. Working with neighborhoods, schools, grocers, farmers, and policymakers, they developed a comprehensive approach to improved food access that combines nutrition education and greater availability of affordable, healthy food. | Family and Community Medicine third-year resident helped to transform corner stores in North Philadelphia to include more fresh produce and healthier options to reduce obesity. |
|  |  |  |
| **Women’s Law Project** [**http://www.womenslawproject.org**](http://www.womenslawproject.org/NewPages/wlpAbout_Us.html) | **A non-profit organization whose mission** is to create a more just and equitable society by advancing the rights and status of all women throughout their lives. To this end, they engage in high-impact litigation, advocacy, and education. | Fourth-year medical student worked with legal advocates to educate policy makers on issues related to reproductive rights. |
