# Supplementary material for: The JeffSTARS Advocacy and Community Partnership Elective: A Closer Look at Child Health Advocacy in Action
Source: MedEdPORTAL. 2016 Dec 31;12:10526. doi: 10.15766/mep_2374-8265.10526 (PMC6365684; doi:10.15766/mep_2374-8265.10526)
Supplement: Supplementary file 1 — A. CM1. Course Implementation at New Institution Checklist.docx B. CM2. Elective Checklist.docx C. CM3. Sample Schedule.docx D. CM4. Seminar Topic List With Learning Objectives.docx E. CM5. Syllabus Bibliography.docx F. CM6. List of Community Partners.docx G. CM7. Orientation for New Community Partner.docx H. CM8. Selected Past Projects.docx I. CM9. Sample Fact Sheets for Legislative Visits.docx J. Seminar Materials folder K. ET1. Advocacy Elective Assessment 1.pdf L. ET2. Advocacy Elective Assessment 2.pdf M. ET3. Trainee Evaluation by Community or Faculty Mentor.docx N. ET4. Trainee Evaluation of Seminar.docx O. ET5. Trainee Evaluation of Community Partner.docx P. ET6. Final Report Template.docx Q. Selected Trainee Abstracts and Presented Results folder [file mep-12-10526-s001.zip › I._CM9._Sample_Fact_Sheets_for_Legislative_Visits.docx]

| 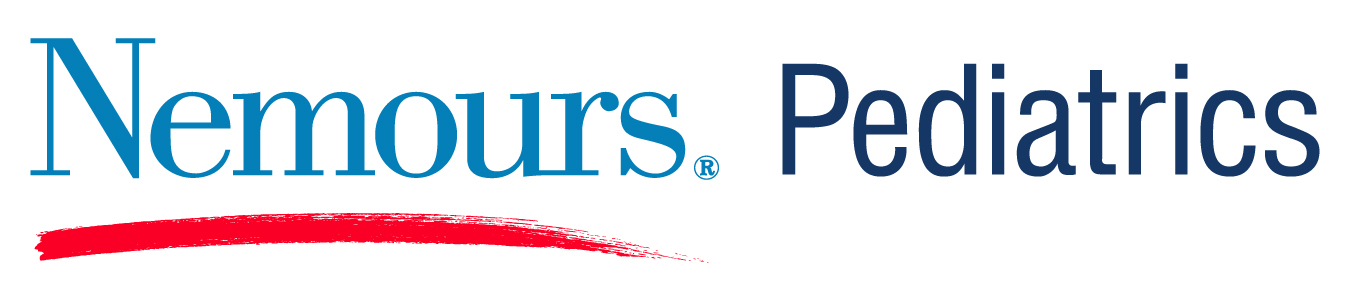 | **833 Chestnut Street** |
| --- | --- |
|  | Suite 300 |
|  | Philadelphia, PA 19107 |
|  |  |

*H.R. 5876 of the 112^th^ Congress proposed an* a*mendment to promote educational stability and success for children in foster care through changes to Part A of Title 1 of the Elementary and Secondary Education Act (ESEA).* *The amendment places obligations on school districts that complement those already placed on child welfare agencies in the 2008 Fostering Connections to Success and Increasing Adoptions Act.*

**Background on education of children and youth in foster care**

- More than 400,000 U.S. children and youth were in foster care at the end of 2009.
- Studies show that 75% of children in foster care are performing below grade level, 35% are in special education and as few as 11% attend college. Nationally, nearly half of youth who age out of foster care will not complete high school by age 18.
- Specific barriers facing youth in care include high rates of school mobility; delays in school enrollment; inappropriate school placements; lack of remedial support; failure to transfer full course credits; and difficulties accessing special education services.
- In Pennsylvania, 35% of foster youth experience three or more moves within a 12-23 month period.
- Estimated that students lose 4-6 months of academic progress with each school move.
- While the *Fostering Connections to Success and Increasing Adoptions Act* mandated state child welfare agencies to improve educational stability for children within the foster care system, it failed to place responsibility on school districts.
  - This places foster care children at increased risk for poor educational outcomes due to ineffective interagency collaboration.

**The ESEA Reauthorization Bill promotes educational stability for children in foster care by requiring school districts and child welfare agencies to collaborate to ensure that:**

- Children, when it is in their best interest, remain in their original school when they enter foster care, move from one foster care placement to another, or leave care during a school year.
- When it is not in a child’s best interest to remain in the same school, the child will be immediately enrolled in a new school and the child’s school records will be maintained and made available in a timely fashion and immediately transferred to the new school.
- A child in foster care who must change schools is assisted in transferring and recovering credits so that he or she can remain on track to receive a secondary school diploma.

**The Bill would also mandate the following provisions:**

- The state educational agency and state child welfare agency must develop an agreement to ensure transportation for children to and from their school of origin when it is in their best interest to remain there.
- The agreement must include a description of how foster care maintenance payments can be used for this purpose and how transportation will be maintained for children who leave foster care before the end of the school year.
- A description by the state and local educational agencies about how they plan to meet the obligations above.

**How you can help**

- Help to support the achievement of positive educational outcomes for children in foster care.
- Introduce and support bills similar to HB5876 of the 112^th^ Congress, that place obligations on school districts, therefore better facilitating interagency collaboration and mutual responsibility for the education of this vulnerable population.

| 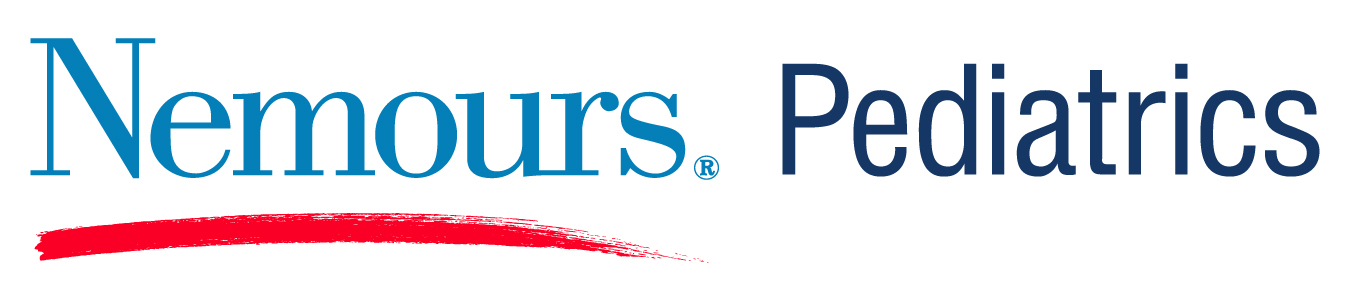 | **833 Chestnut Street** |
| --- | --- |
|  | Suite 300 |
|  | Philadelphia, PA 19107 |
|  |  |

**Neonatal Abstinence Syndrome (NAS)**

**•** Neonatal abstinence syndrome (NAS) is a term used to describe a group of symptoms in a newborn undergoing withdrawal from maternal drug use.

• Symptoms of NAS begin within days of birth and can last anywhere from 6-8 months. These symptoms include irritability, tremors, increased muscle tone, poor feeding, fevers, and seizures.

•In the last decade, there has been a parallel in two national trends: a rise in the prevalence of prescription opioid abuse and a rise in the incidence of NAS.

• In the US, maternal opioid use from 2000-2009 increased from 1.2 to 5.6 per 1,000 births while the diagnosis of NAS in newborns increased from 1.2 to 3.4 per 1,000 births.

• There is great variability with regard to how hospitals in PA are screening for, diagnosing, and managing NAS which results in unnecessary morbidity and a lack of cost effective care.

**Consequences of Neonatal Abstinence Syndrome**

• In the newborn, poor intrauterine growth, prematurity, seizures, and birth defects have all been associated with opioid exposure.

• NAS has been associated with long term developmental effects including an increased risk for cognitive difficulties, learning disorders, and behavioral disorders.

• For every newborn with NAS born in PA, the average cost for newborn care is $53,400 compared to the average cost of $9,000 for a healthy newborn.

• State Medicaid budgets are feeling the impact of the rise in NAS rates as Medicaid covers the medical expenses for approximately 78% percent of newborns with NAS.

**Current Legislation**

**•** More than 20 states have enacted legislation specific to addressing substance dependence during the perinatal period including adding prenatal drug screening to their Medicaid programs and requiring hospitals to report NAS newborns to the state health department in order to centralize data collection, track the epidemic, and identify means for intervention.

• HB 725: an act requiring that all Child Protective Service Workers undergo training in alcohol, drug abuse, and addiction, warning signs of alcohol and drug dependency in families, and how to make appropriate referrals for assessment and treatment.

**What can you do to help**

• Support or initiate legislation that increases resources for drug dependent pregnant women and their children including public awareness campaigns, medication assisted drug treatment programs and early childhood intervention services.

• Support HB 725 and other legislation to help education and train personnel who are in regular contact with vulnerable populations
