# Supplementary material for: The JeffSTARS Advocacy and Community Partnership Elective: A Closer Look at Child Health Advocacy in Action
Source: MedEdPORTAL. 2016 Dec 31;12:10526. doi: 10.15766/mep_2374-8265.10526 (PMC6365684; doi:10.15766/mep_2374-8265.10526)
Supplement: Supplementary file 1 — A. CM1. Course Implementation at New Institution Checklist.docx B. CM2. Elective Checklist.docx C. CM3. Sample Schedule.docx D. CM4. Seminar Topic List With Learning Objectives.docx E. CM5. Syllabus Bibliography.docx F. CM6. List of Community Partners.docx G. CM7. Orientation for New Community Partner.docx H. CM8. Selected Past Projects.docx I. CM9. Sample Fact Sheets for Legislative Visits.docx J. Seminar Materials folder K. ET1. Advocacy Elective Assessment 1.pdf L. ET2. Advocacy Elective Assessment 2.pdf M. ET3. Trainee Evaluation by Community or Faculty Mentor.docx N. ET4. Trainee Evaluation of Seminar.docx O. ET5. Trainee Evaluation of Community Partner.docx P. ET6. Final Report Template.docx Q. Selected Trainee Abstracts and Presented Results folder [file mep-12-10526-s001.zip › J._Seminar_Materials_folder/1._Introduction_to_Advocacy.pptx]

## Slide 1
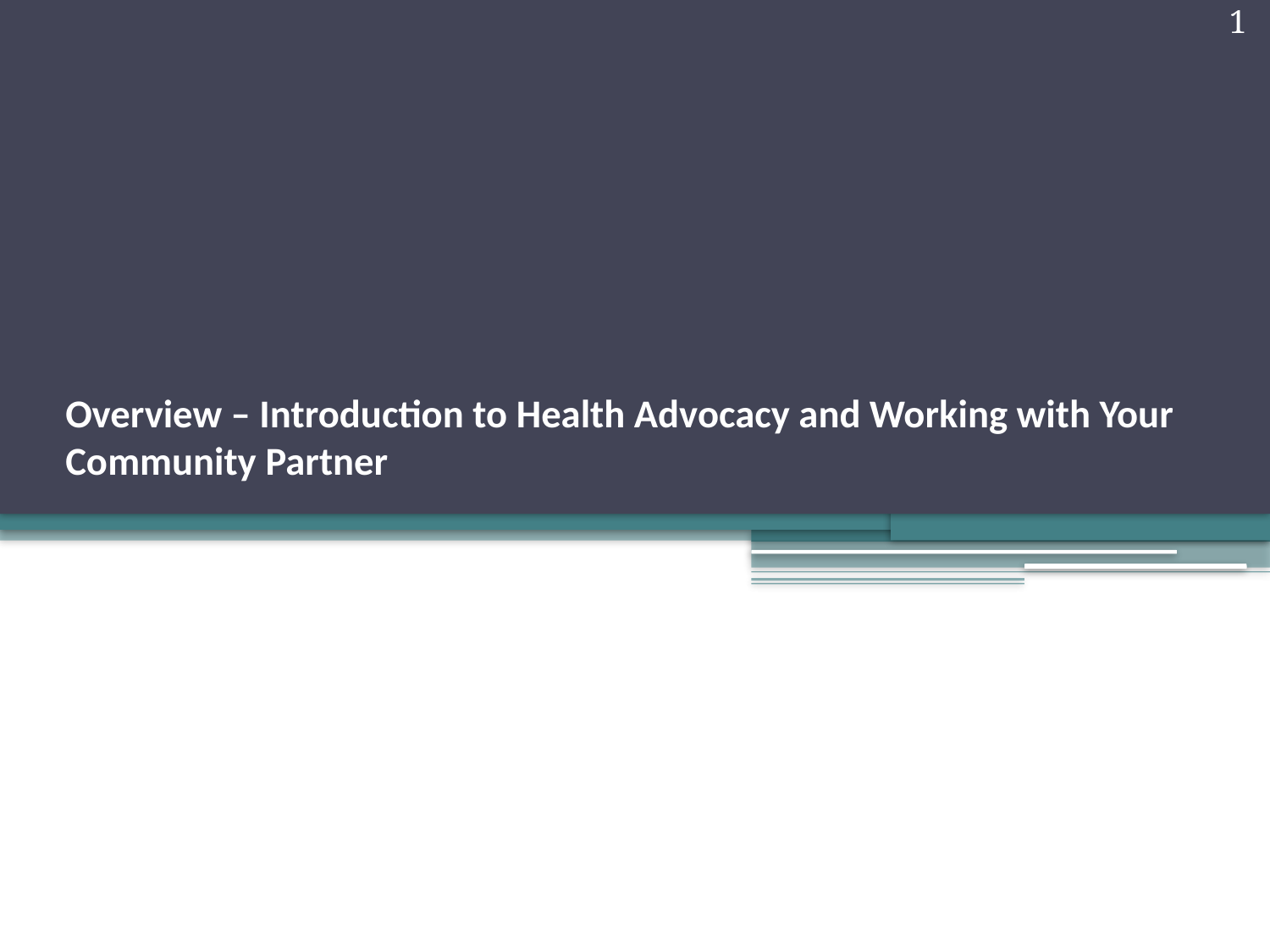

1
# Overview – Introduction to Health Advocacy and Working with Your Community Partner

## Slide 2
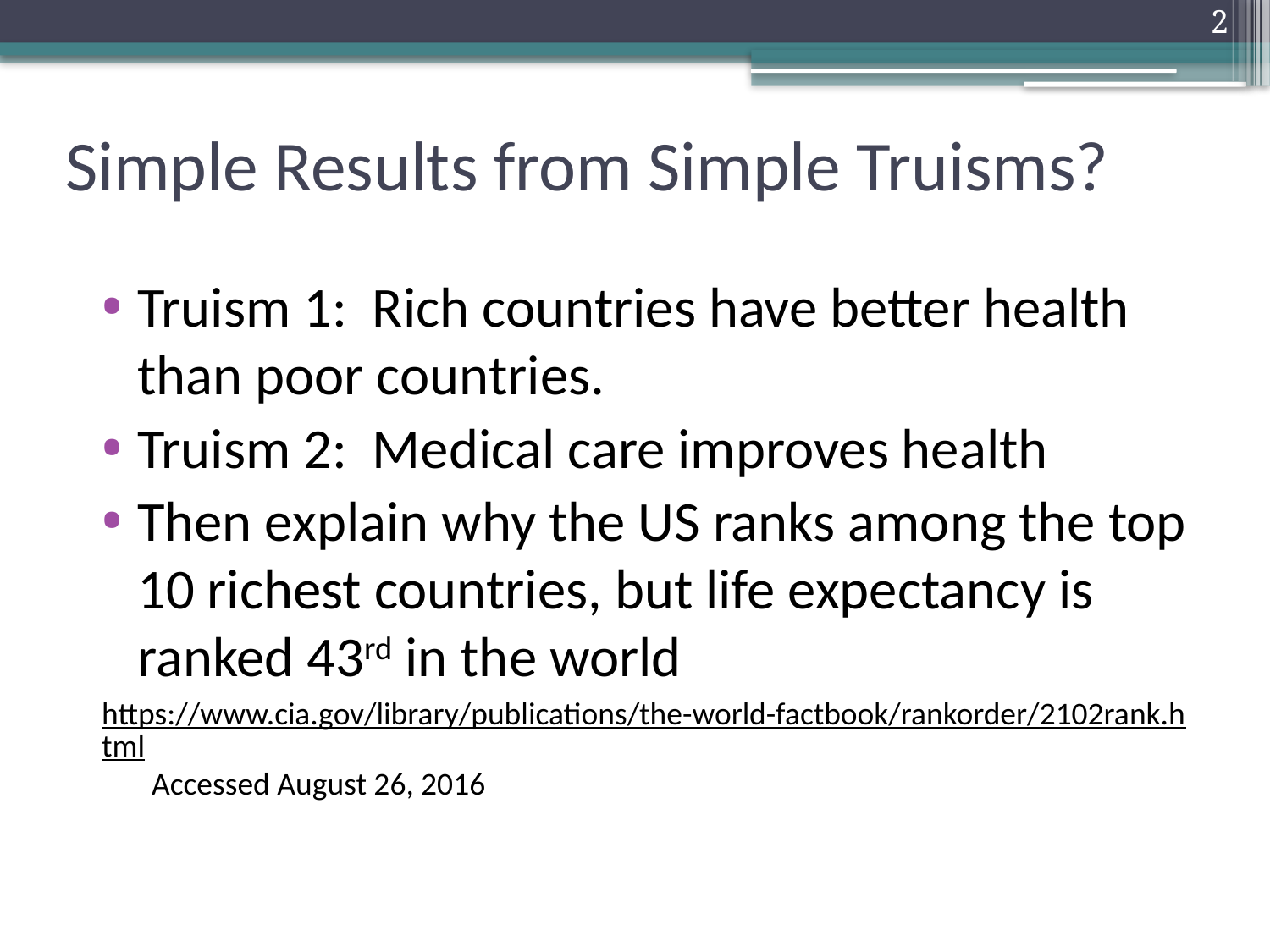

2
# Simple Results from Simple Truisms?
Truism 1: Rich countries have better health than poor countries.
Truism 2: Medical care improves health
Then explain why the US ranks among the top 10 richest countries, but life expectancy is ranked 43rd in the world
https://www.cia.gov/library/publications/the-world-factbook/rankorder/2102rank.html Accessed August 26, 2016

## Slide 3
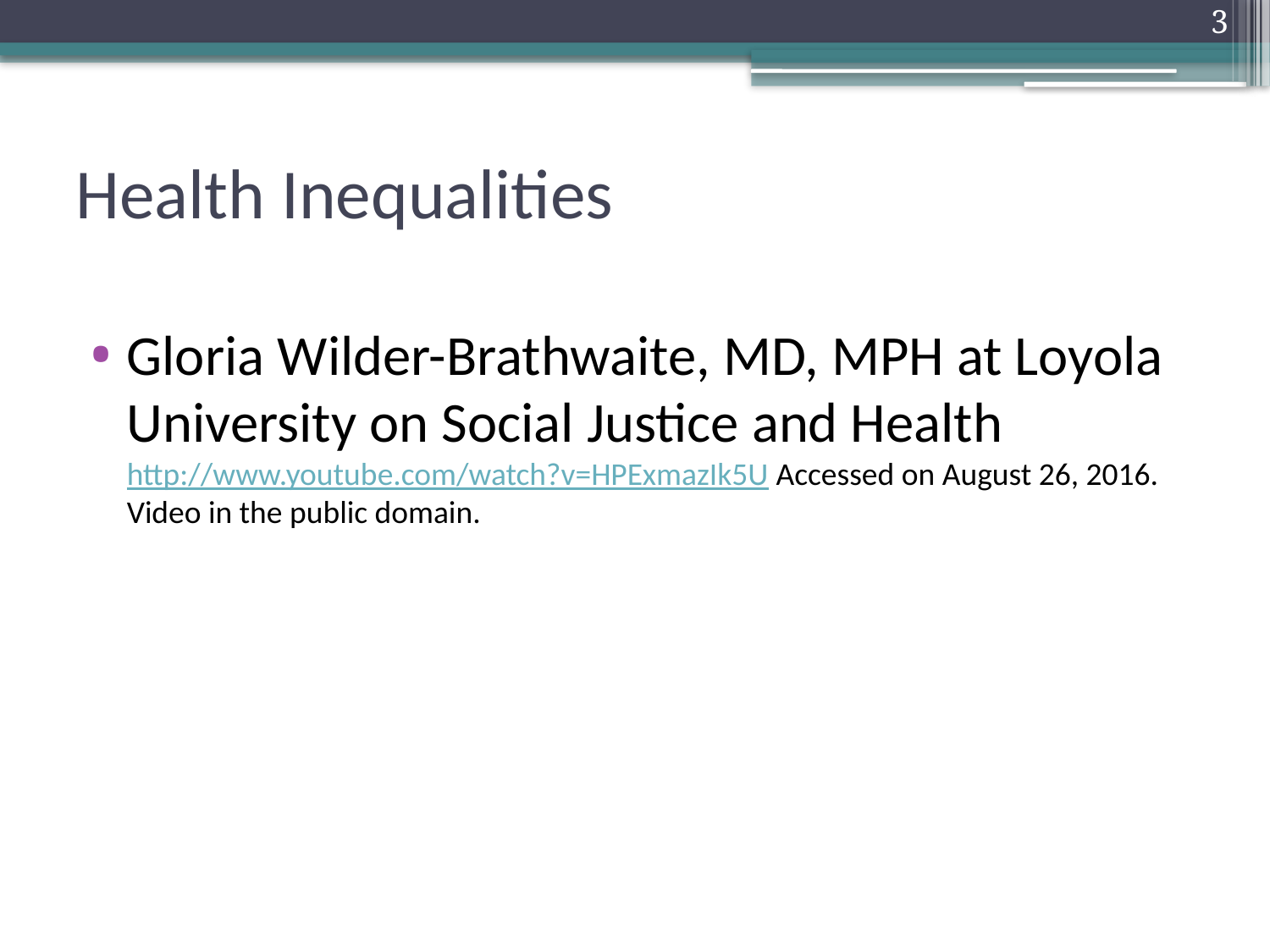

3
# Health Inequalities
Gloria Wilder-Brathwaite, MD, MPH at Loyola University on Social Justice and Health http://www.youtube.com/watch?v=HPExmazIk5U Accessed on August 26, 2016. Video in the public domain.

## Slide 4
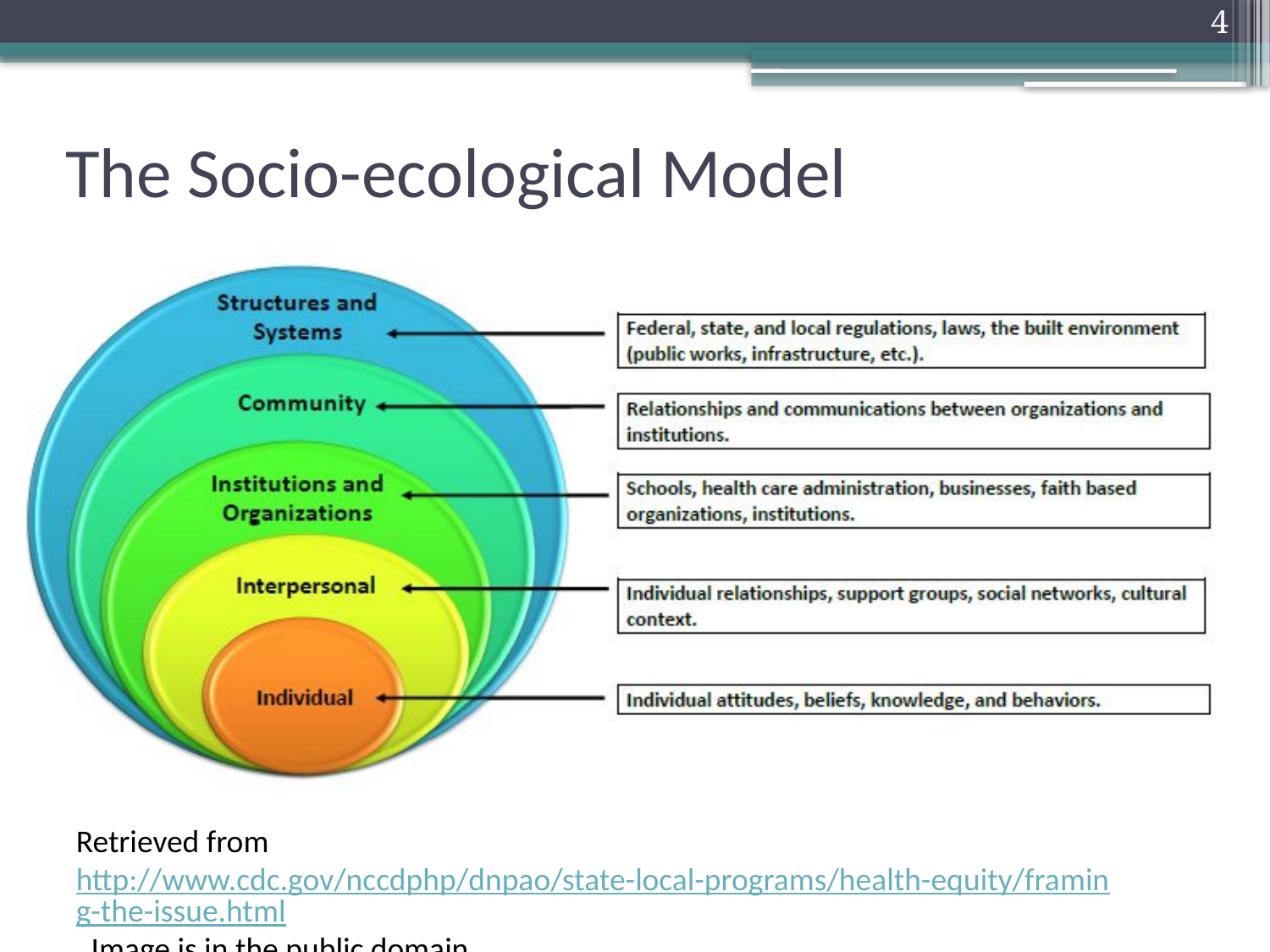

4
# The Socio-ecological Model
Retrieved from http://www.cdc.gov/nccdphp/dnpao/state-local-programs/health-equity/framing-the-issue.html. Image is in the public domain

## Slide 5
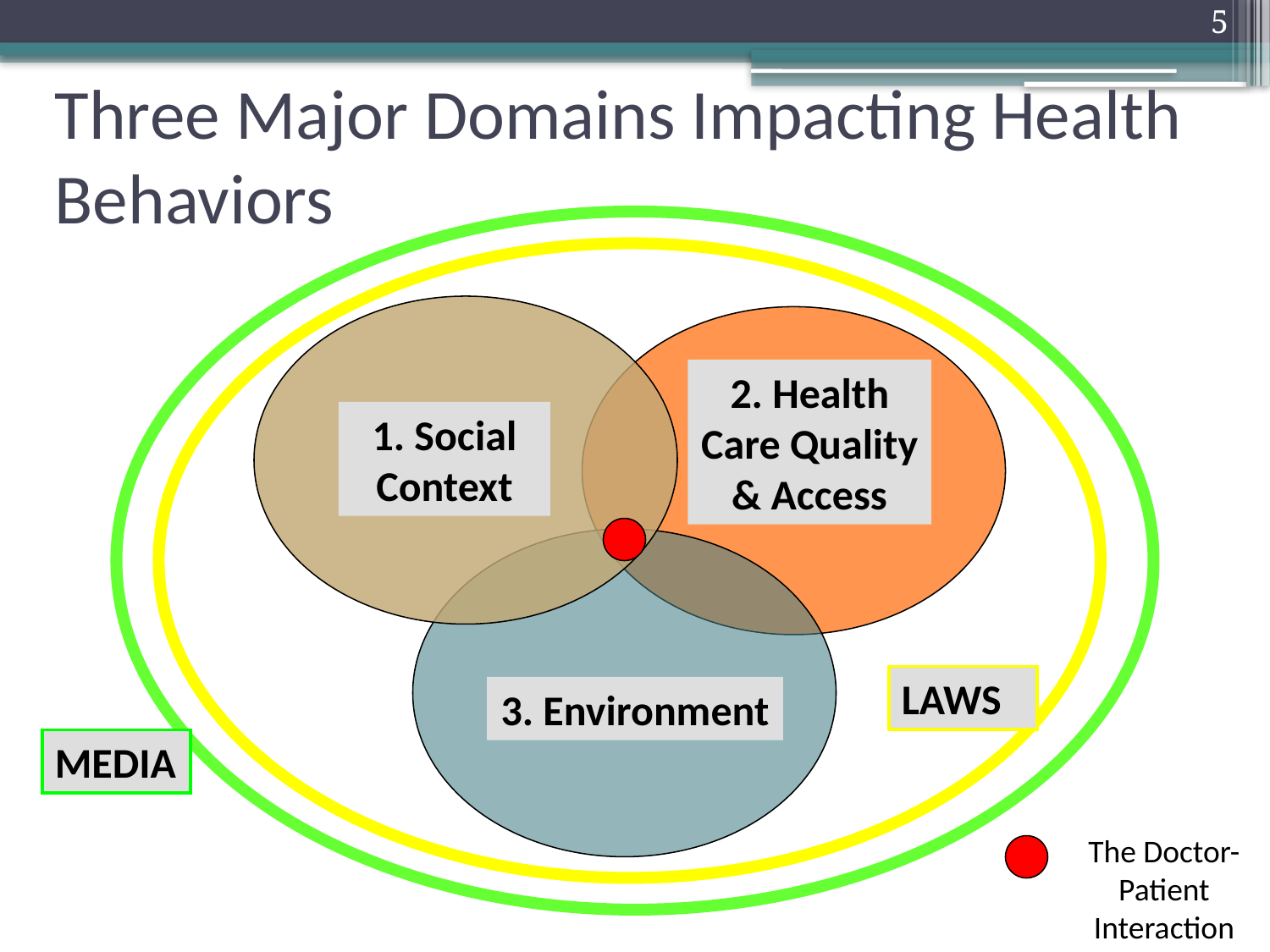

5
# Three Major Domains Impacting Health Behaviors
2. Health Care Quality & Access
1. Social Context
LAWS
3. Environment
MEDIA
The Doctor-Patient Interaction

## Slide 6
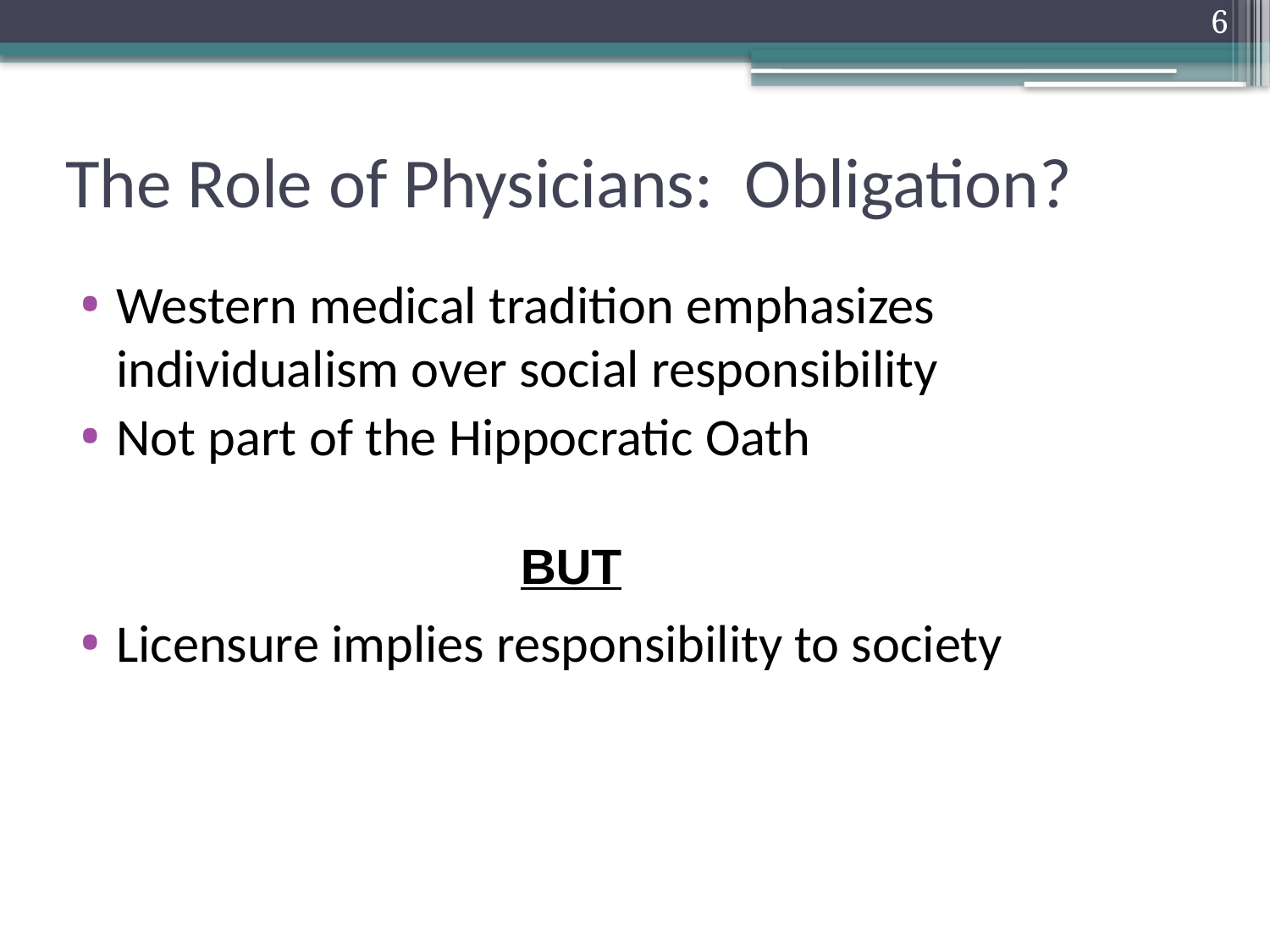

6
# The Role of Physicians: Obligation?
Western medical tradition emphasizes individualism over social responsibility
Not part of the Hippocratic Oath
Licensure implies responsibility to society
BUT

## Slide 7
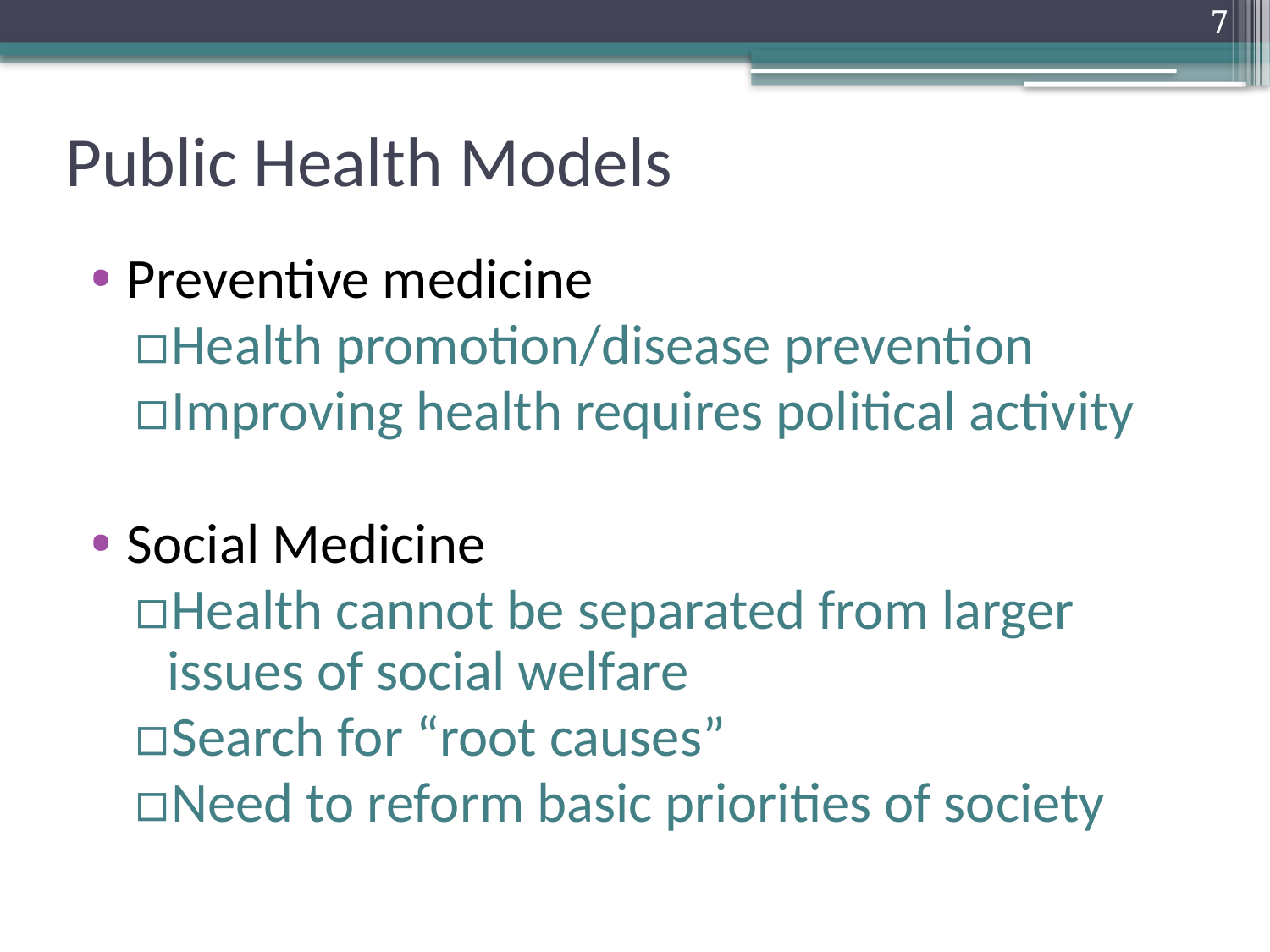

7
# Public Health Models
Preventive medicine
Health promotion/disease prevention
Improving health requires political activity
Social Medicine
Health cannot be separated from larger issues of social welfare
Search for “root causes”
Need to reform basic priorities of society

## Slide 8
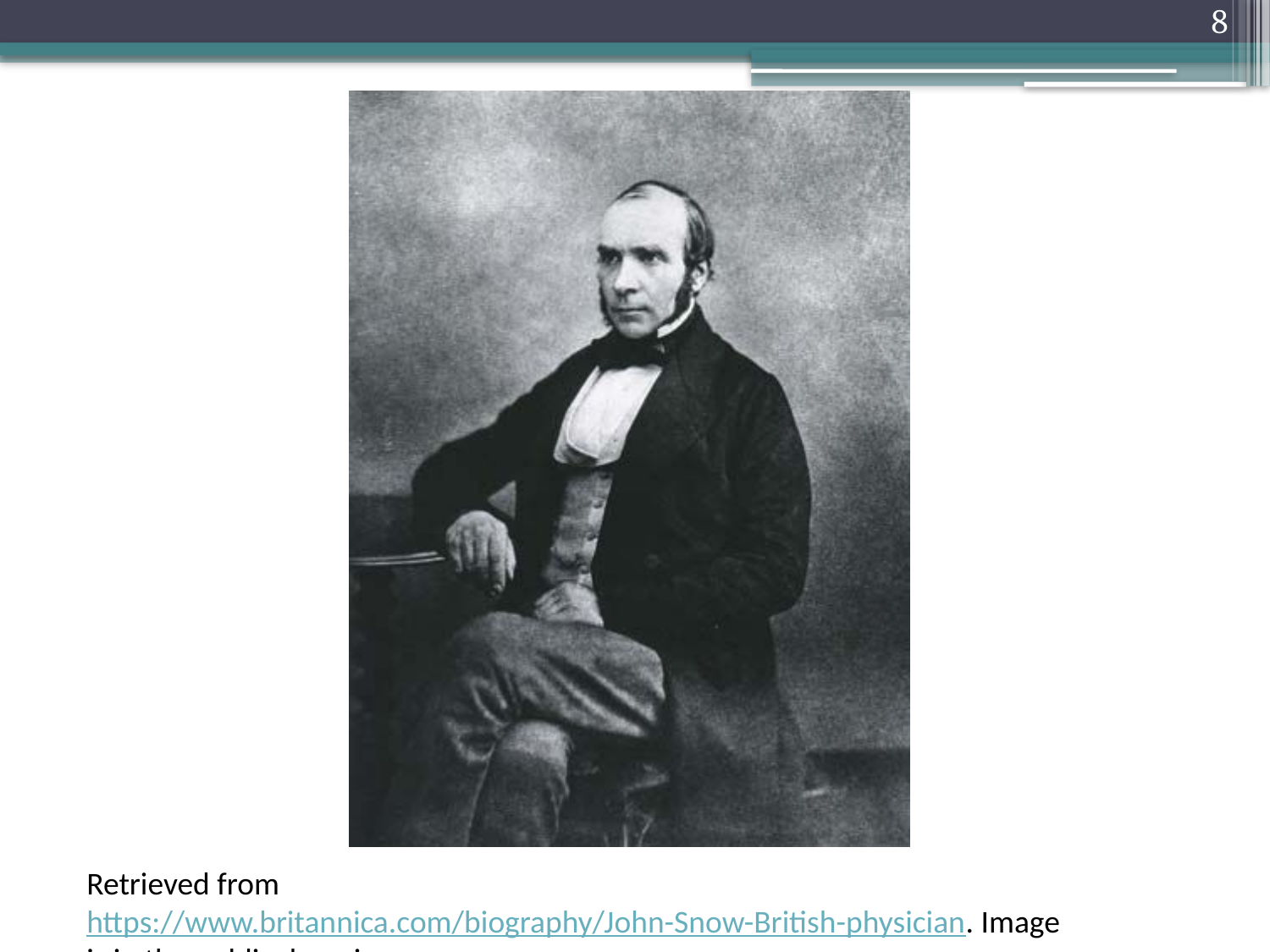

8
Retrieved from https://www.britannica.com/biography/John-Snow-British-physician. Image is in the public domain

## Slide 9
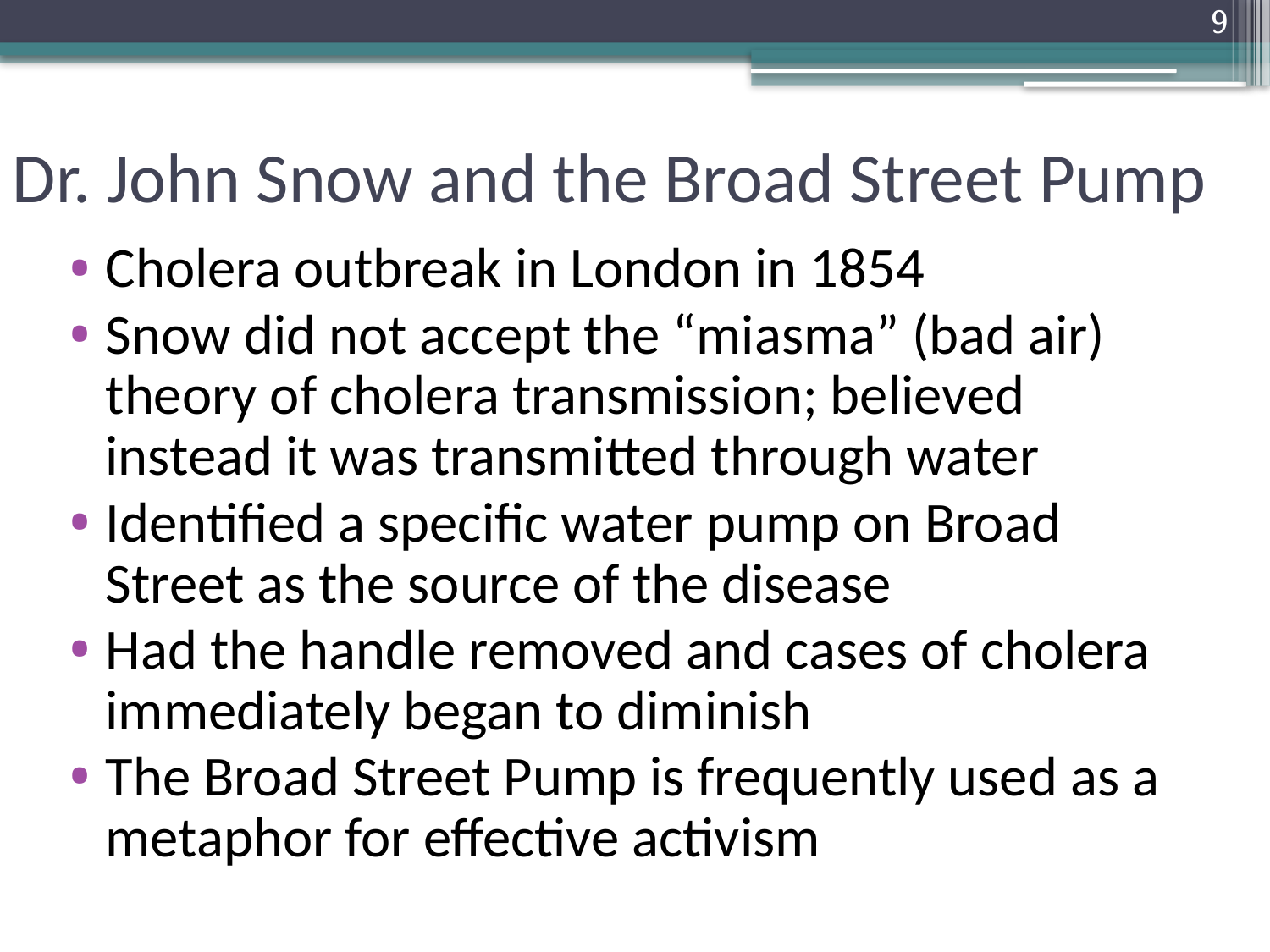

9
# Dr. John Snow and the Broad Street Pump
Cholera outbreak in London in 1854
Snow did not accept the “miasma” (bad air) theory of cholera transmission; believed instead it was transmitted through water
Identified a specific water pump on Broad Street as the source of the disease
Had the handle removed and cases of cholera immediately began to diminish
The Broad Street Pump is frequently used as a metaphor for effective activism

## Slide 10
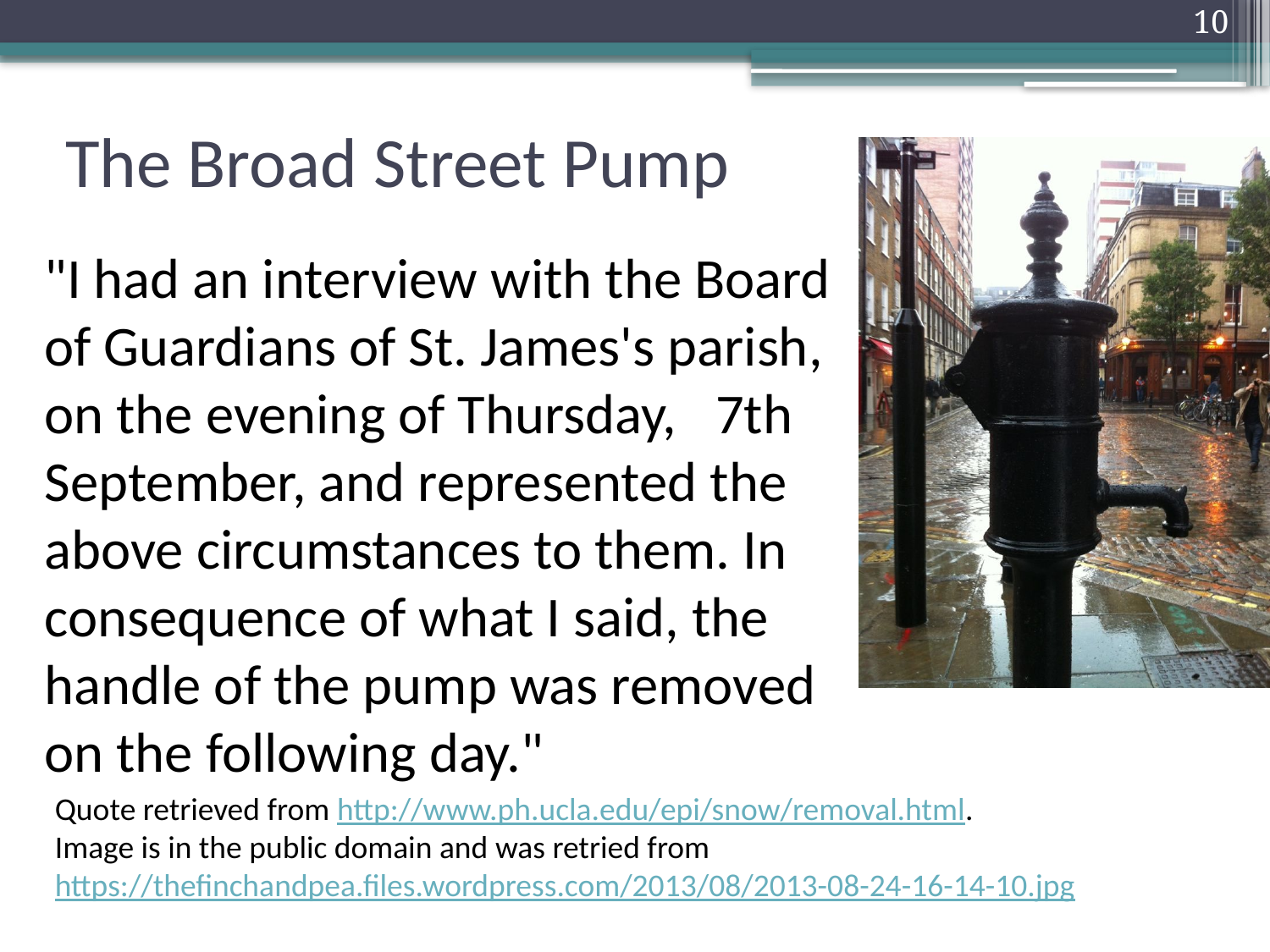

10
# The Broad Street Pump
"I had an interview with the Board of Guardians of St. James's parish, on the evening of Thursday,  7th September, and represented the above circumstances to them. In consequence of what I said, the handle of the pump was removed on the following day."
Quote retrieved from http://www.ph.ucla.edu/epi/snow/removal.html.
Image is in the public domain and was retried from https://thefinchandpea.files.wordpress.com/2013/08/2013-08-24-16-14-10.jpg

## Slide 11
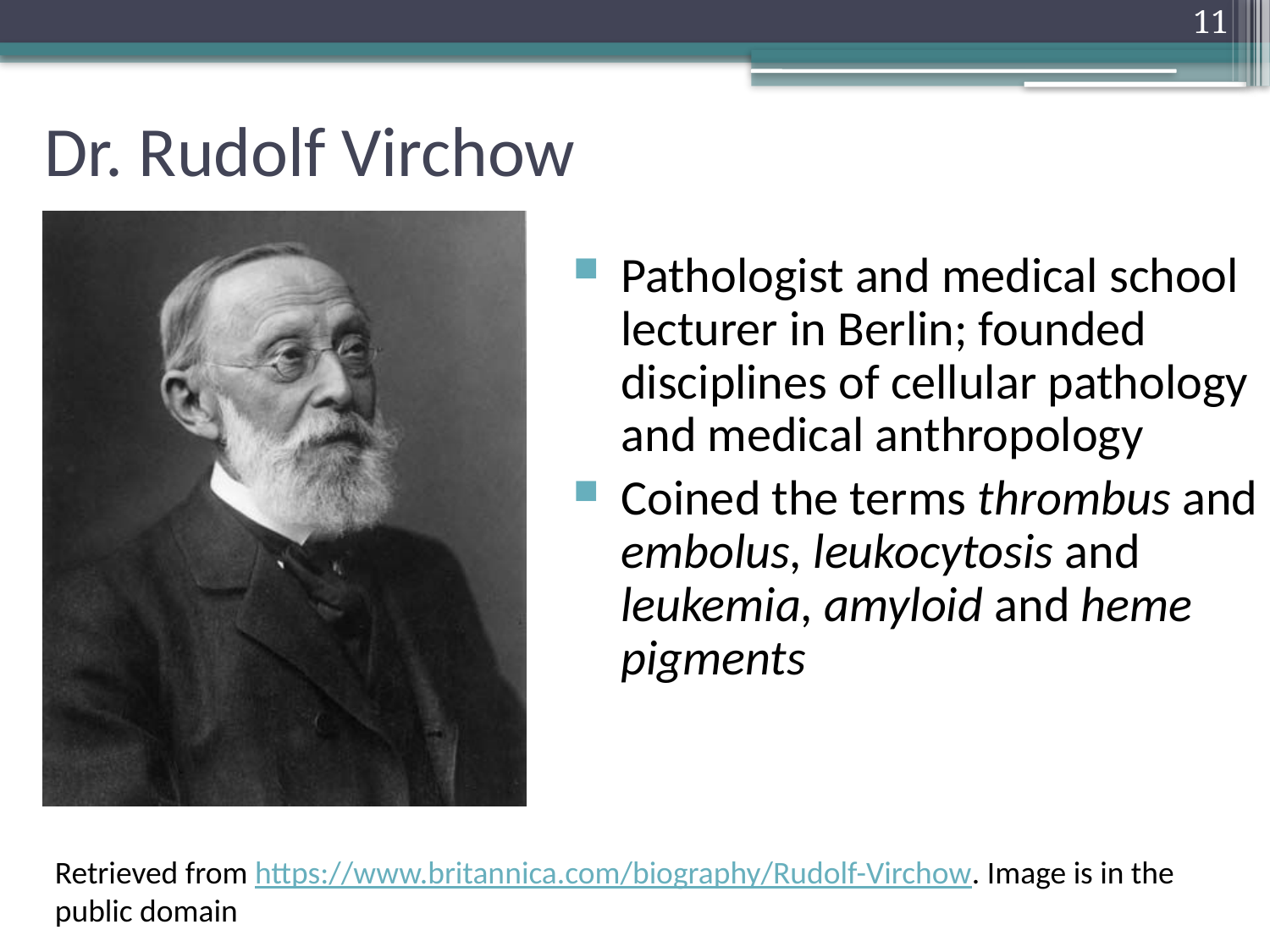

11
# Dr. Rudolf Virchow
Pathologist and medical school lecturer in Berlin; founded disciplines of cellular pathology and medical anthropology
Coined the terms thrombus and embolus, leukocytosis and leukemia, amyloid and heme pigments
Retrieved from https://www.britannica.com/biography/Rudolf-Virchow. Image is in the public domain

## Slide 12
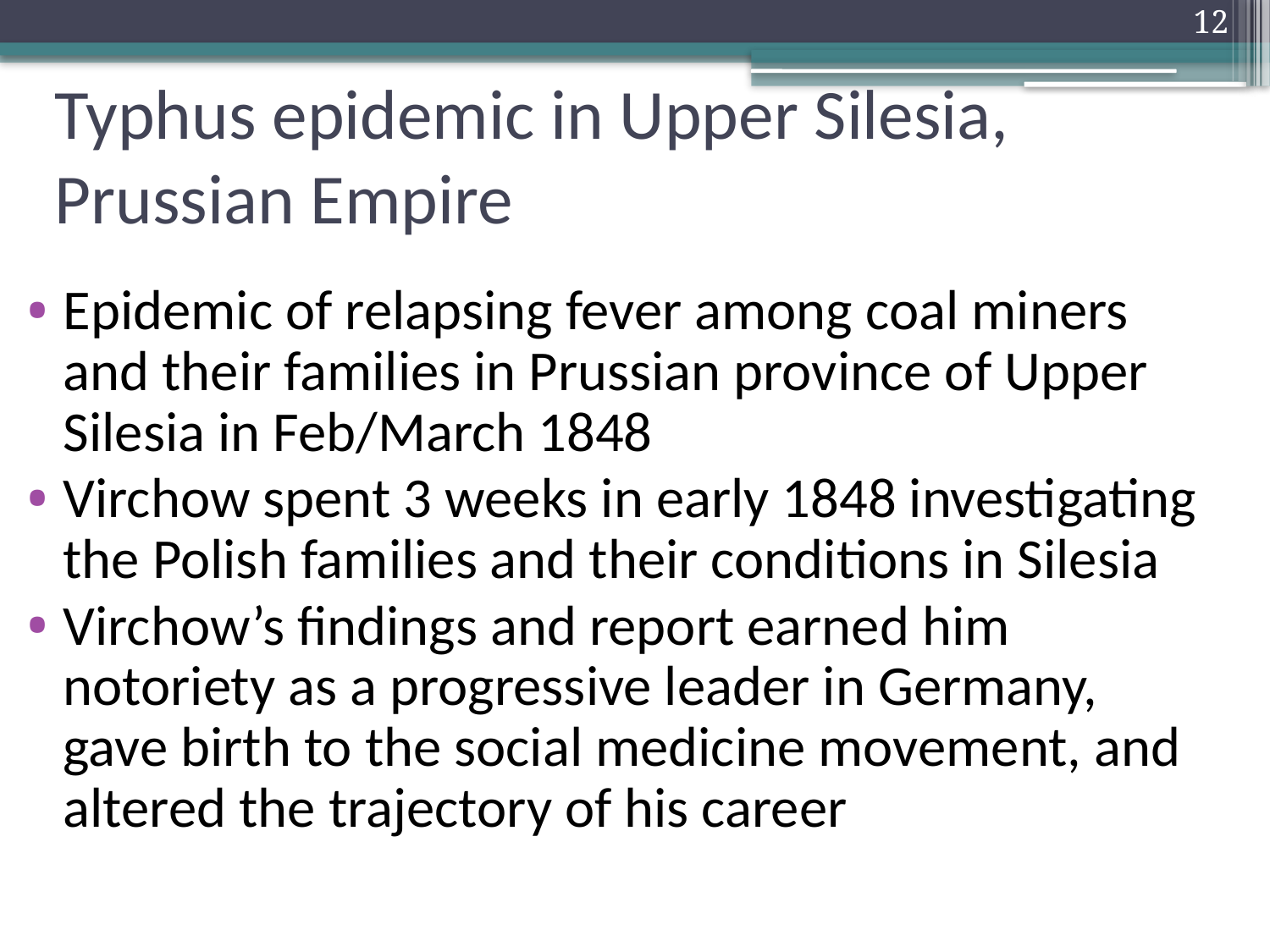

12
# Typhus epidemic in Upper Silesia, Prussian Empire
Epidemic of relapsing fever among coal miners and their families in Prussian province of Upper Silesia in Feb/March 1848
Virchow spent 3 weeks in early 1848 investigating the Polish families and their conditions in Silesia
Virchow’s findings and report earned him notoriety as a progressive leader in Germany, gave birth to the social medicine movement, and altered the trajectory of his career

## Slide 13
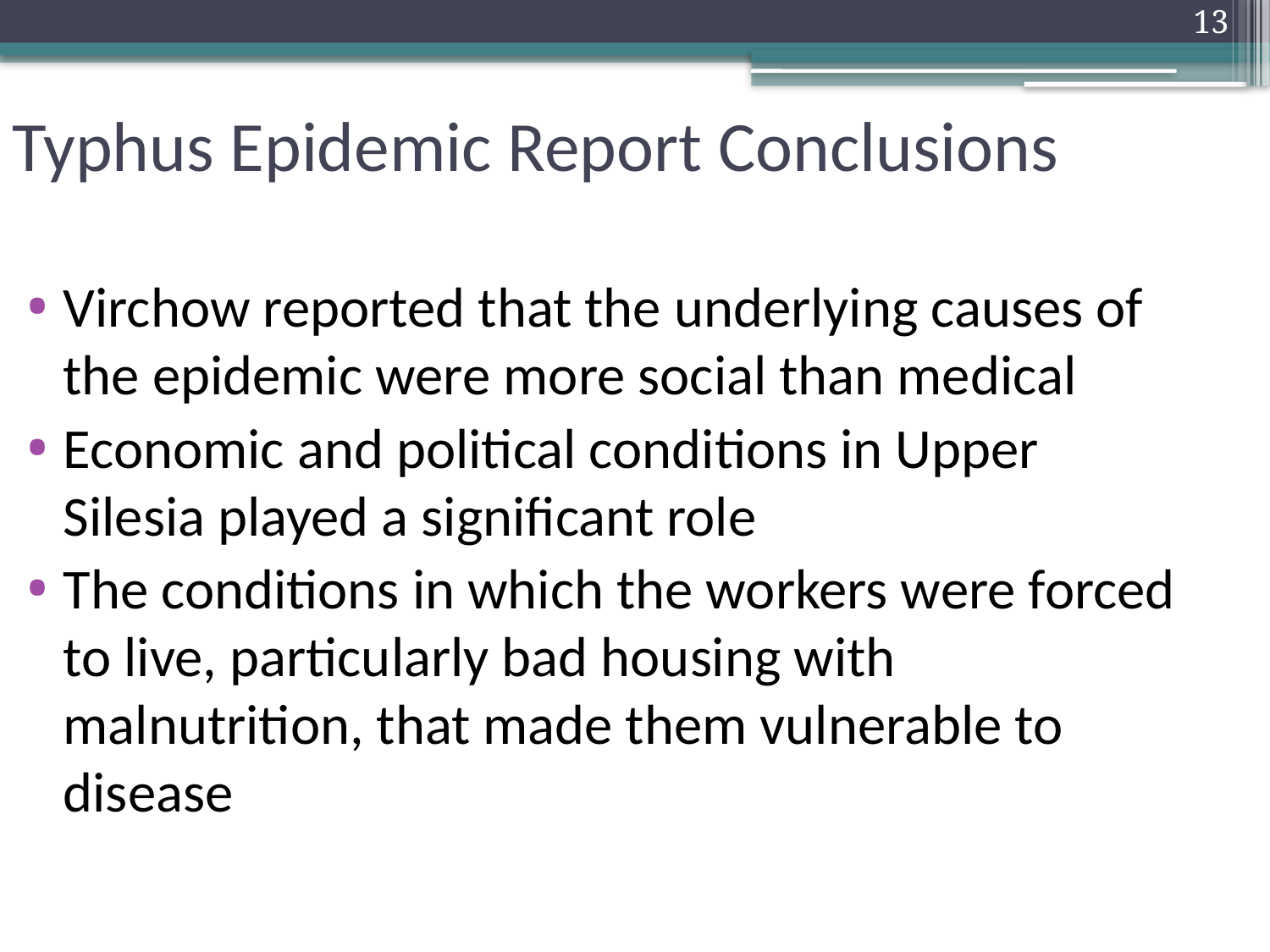

13
# Typhus Epidemic Report Conclusions
Virchow reported that the underlying causes of the epidemic were more social than medical
Economic and political conditions in Upper Silesia played a significant role
The conditions in which the workers were forced to live, particularly bad housing with malnutrition, that made them vulnerable to disease

## Slide 14
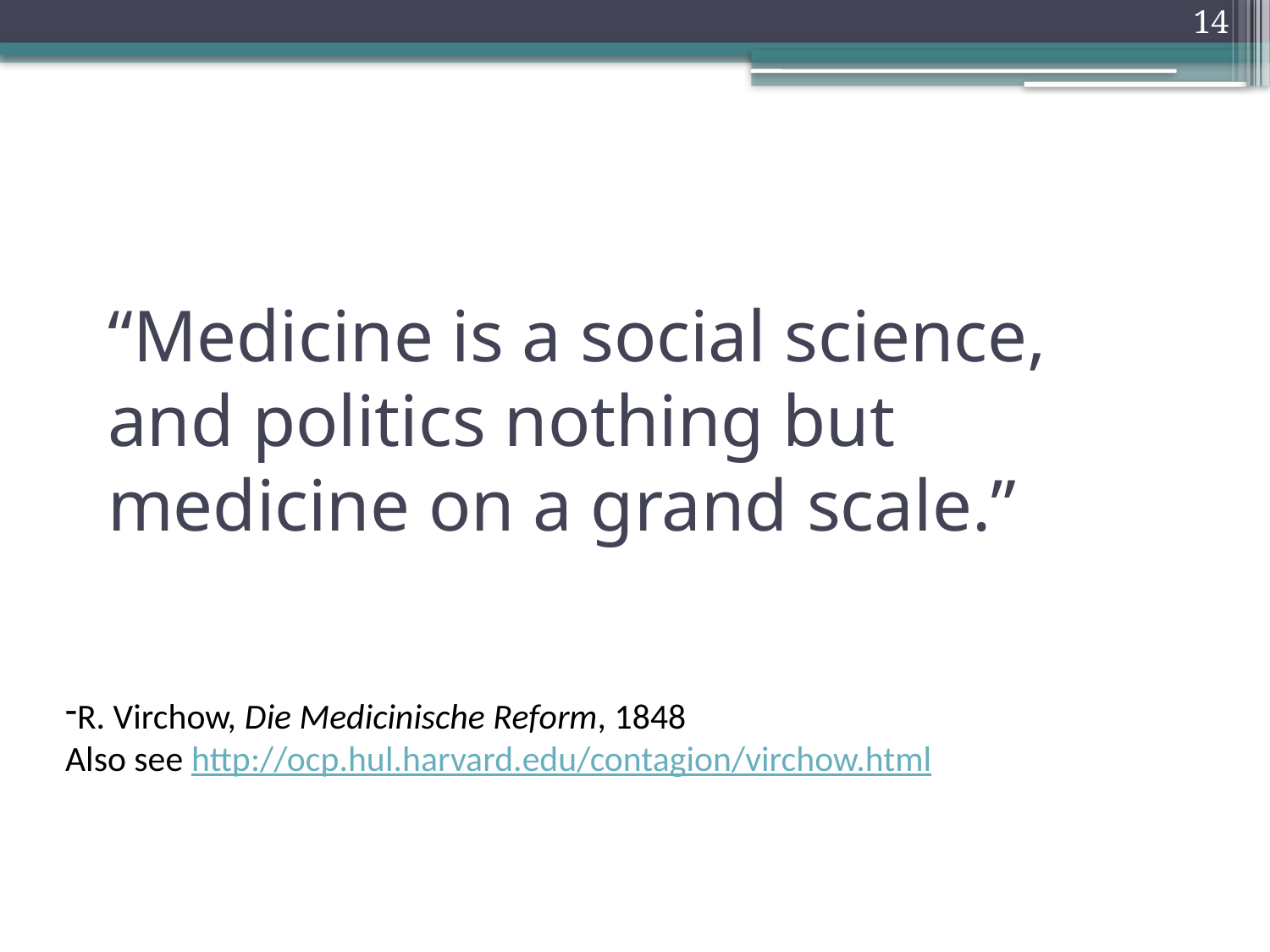

14
# “Medicine is a social science, and politics nothing but medicine on a grand scale.”
R. Virchow, Die Medicinische Reform, 1848
Also see http://ocp.hul.harvard.edu/contagion/virchow.html

## Slide 15
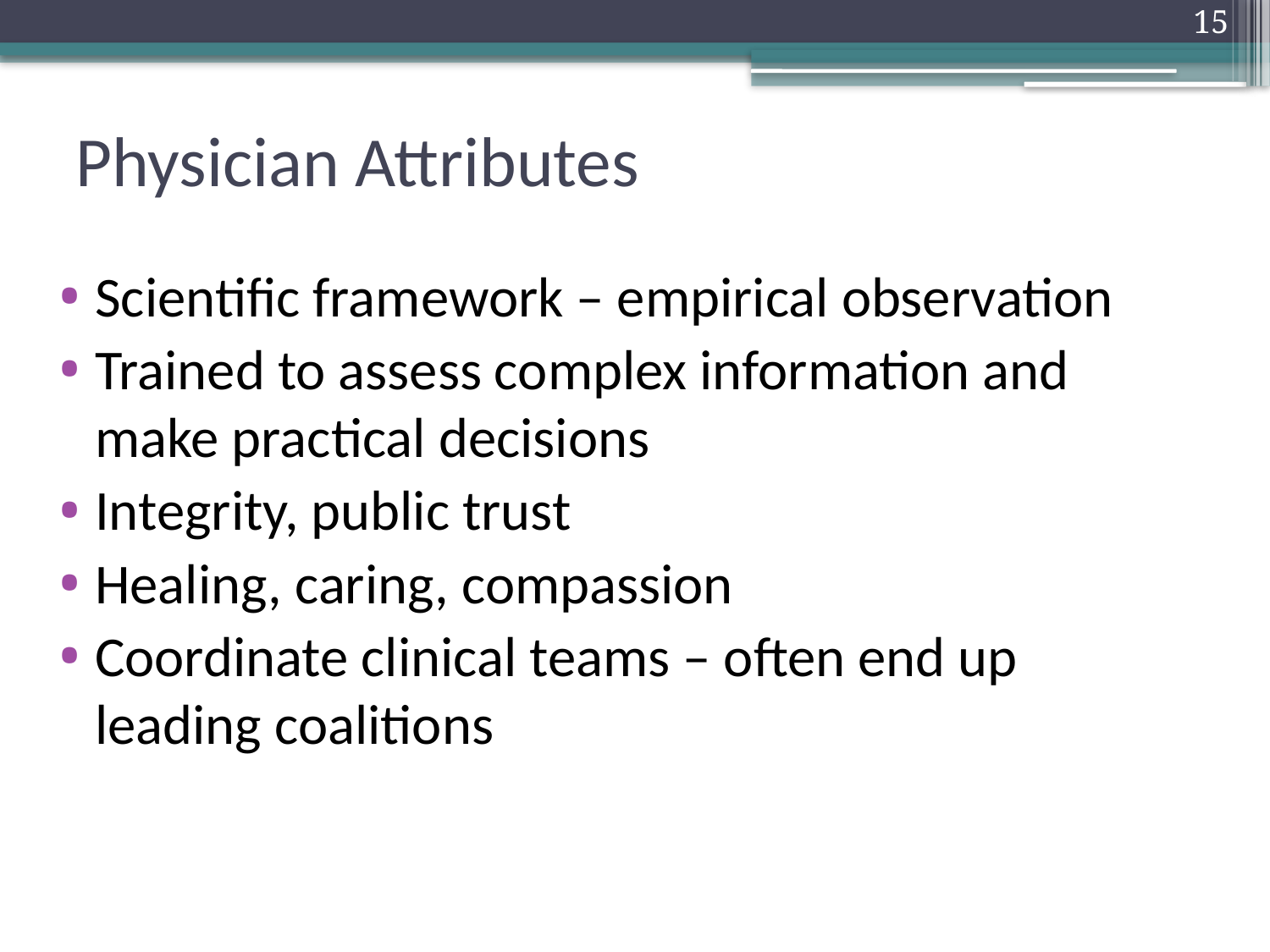

15
# Physician Attributes
Scientific framework – empirical observation
Trained to assess complex information and make practical decisions
Integrity, public trust
Healing, caring, compassion
Coordinate clinical teams – often end up leading coalitions

## Slide 16
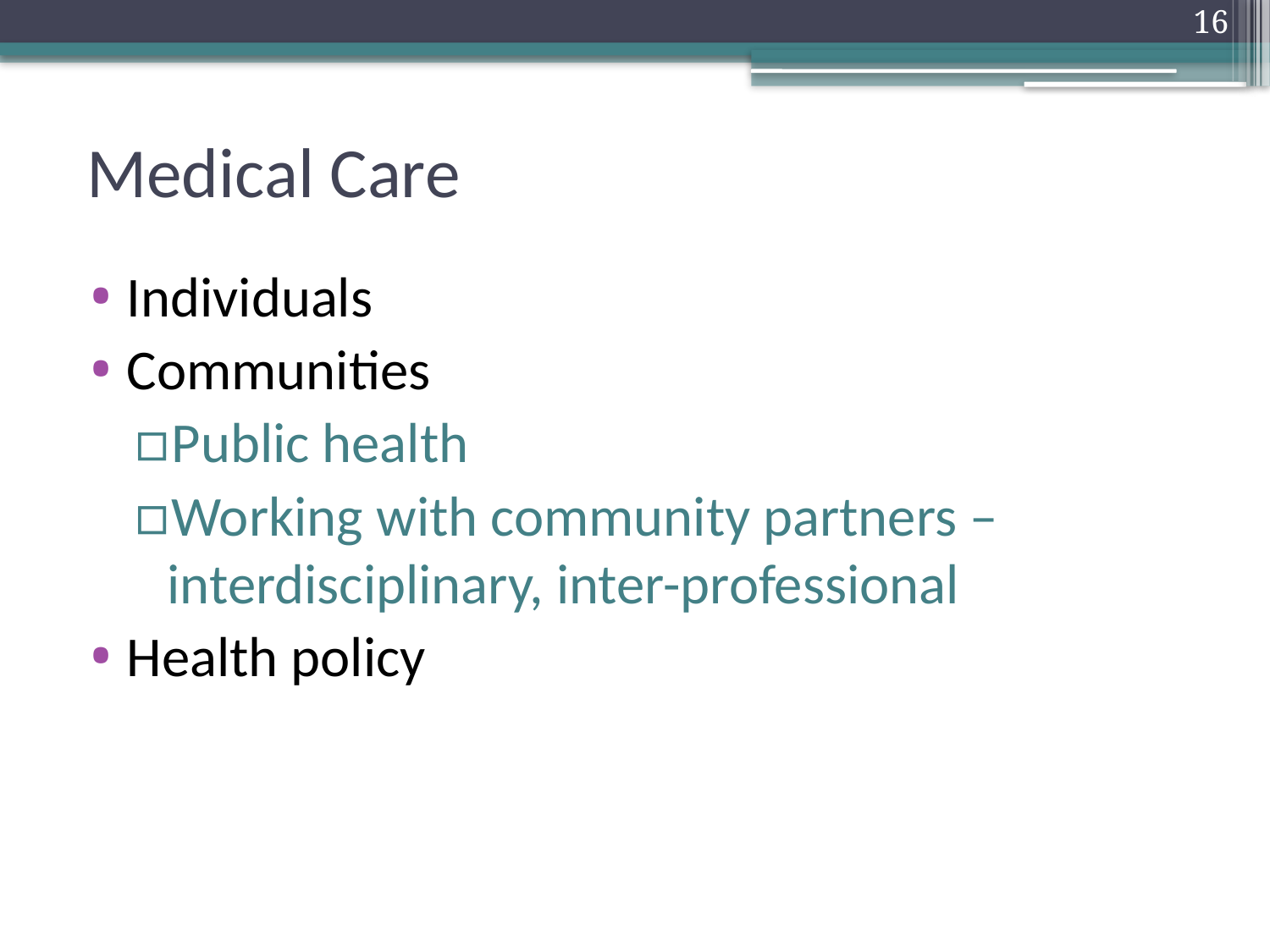

16
# Medical Care
Individuals
Communities
Public health
Working with community partners – interdisciplinary, inter-professional
Health policy

## Slide 17
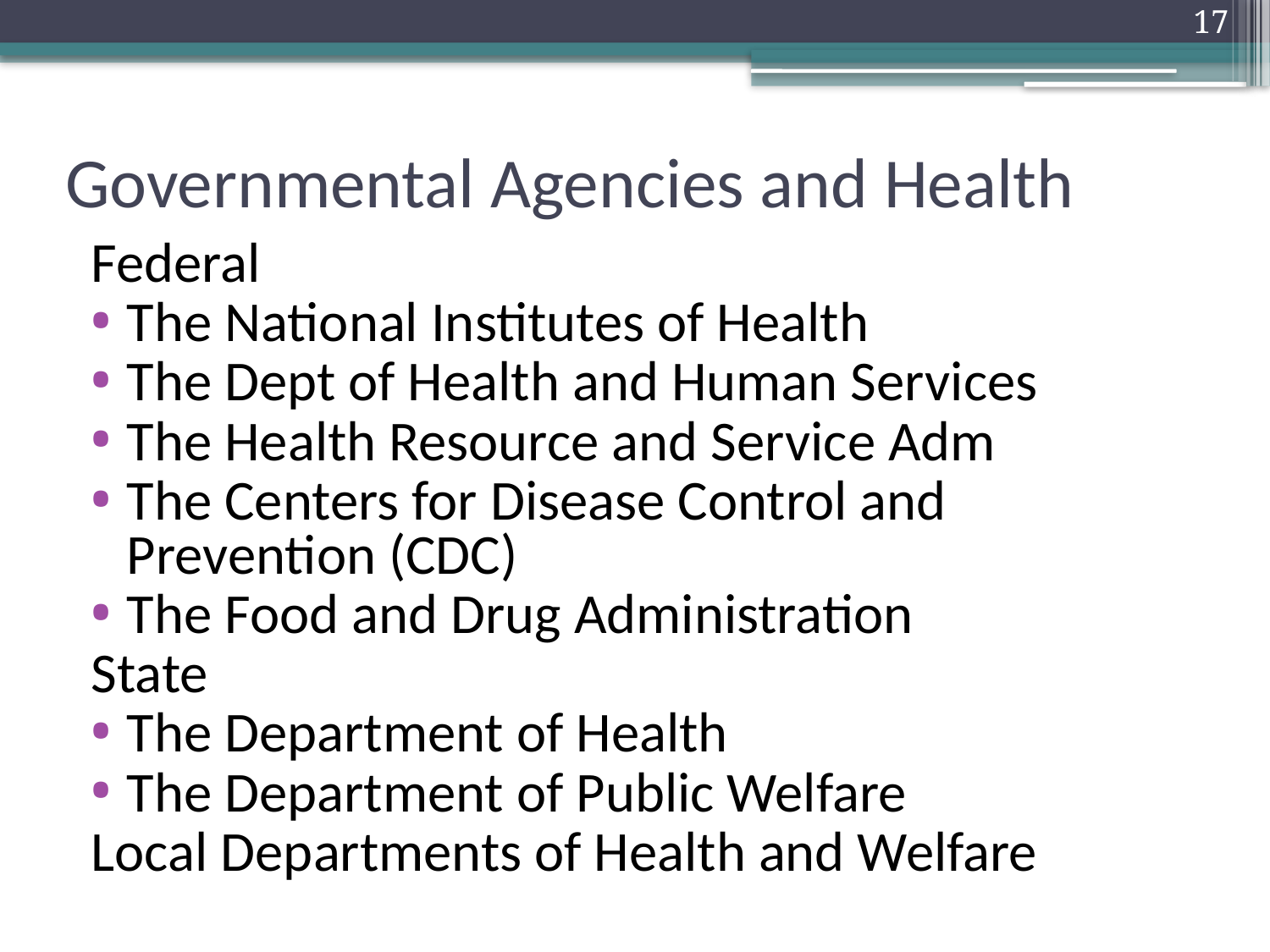

17
# Governmental Agencies and Health
Federal
The National Institutes of Health
The Dept of Health and Human Services
The Health Resource and Service Adm
The Centers for Disease Control and Prevention (CDC)
The Food and Drug Administration
State
The Department of Health
The Department of Public Welfare
Local Departments of Health and Welfare

## Slide 18
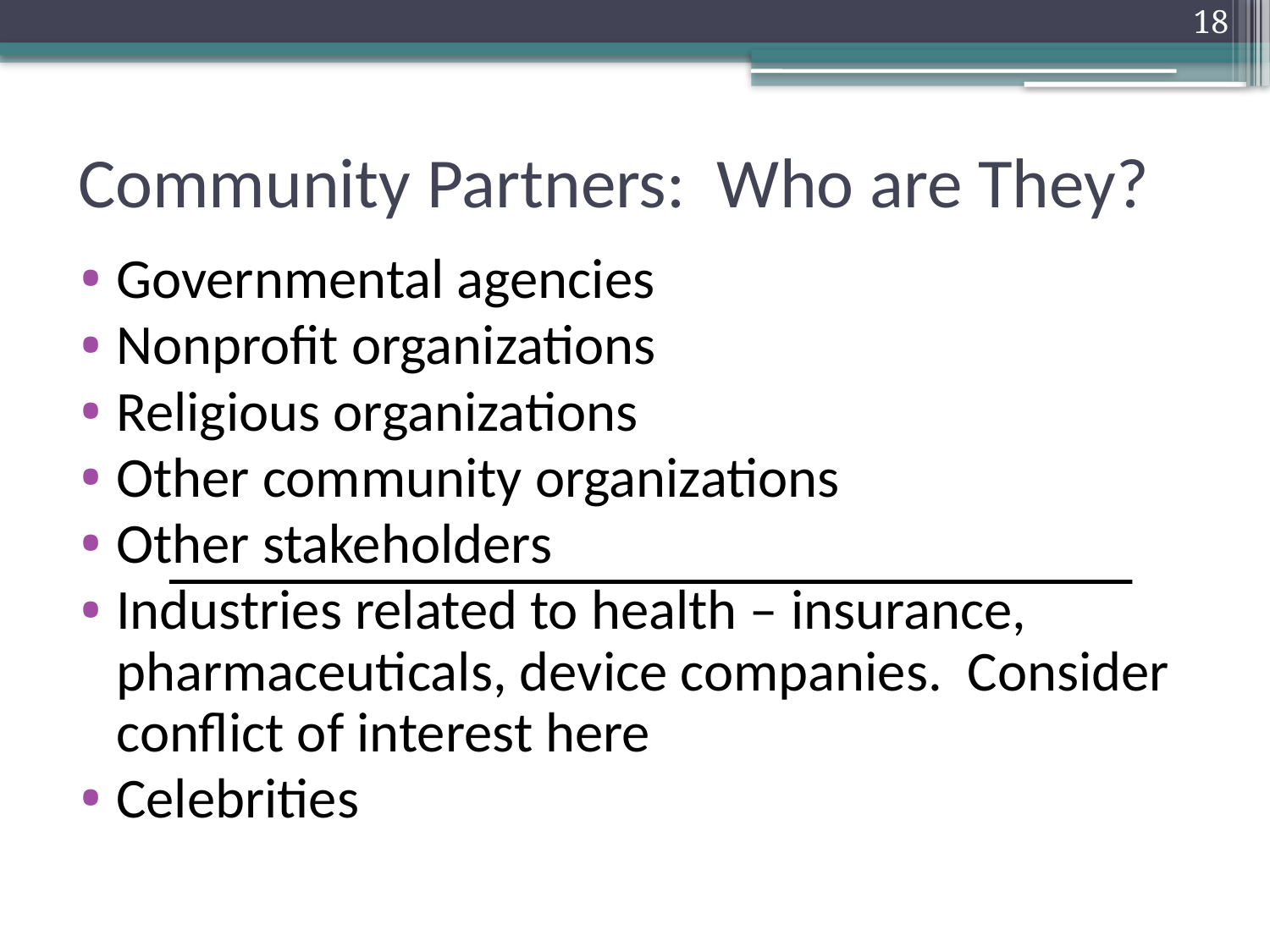

18
# Community Partners: Who are They?
Governmental agencies
Nonprofit organizations
Religious organizations
Other community organizations
Other stakeholders
Industries related to health – insurance, pharmaceuticals, device companies. Consider conflict of interest here
Celebrities

## Slide 19
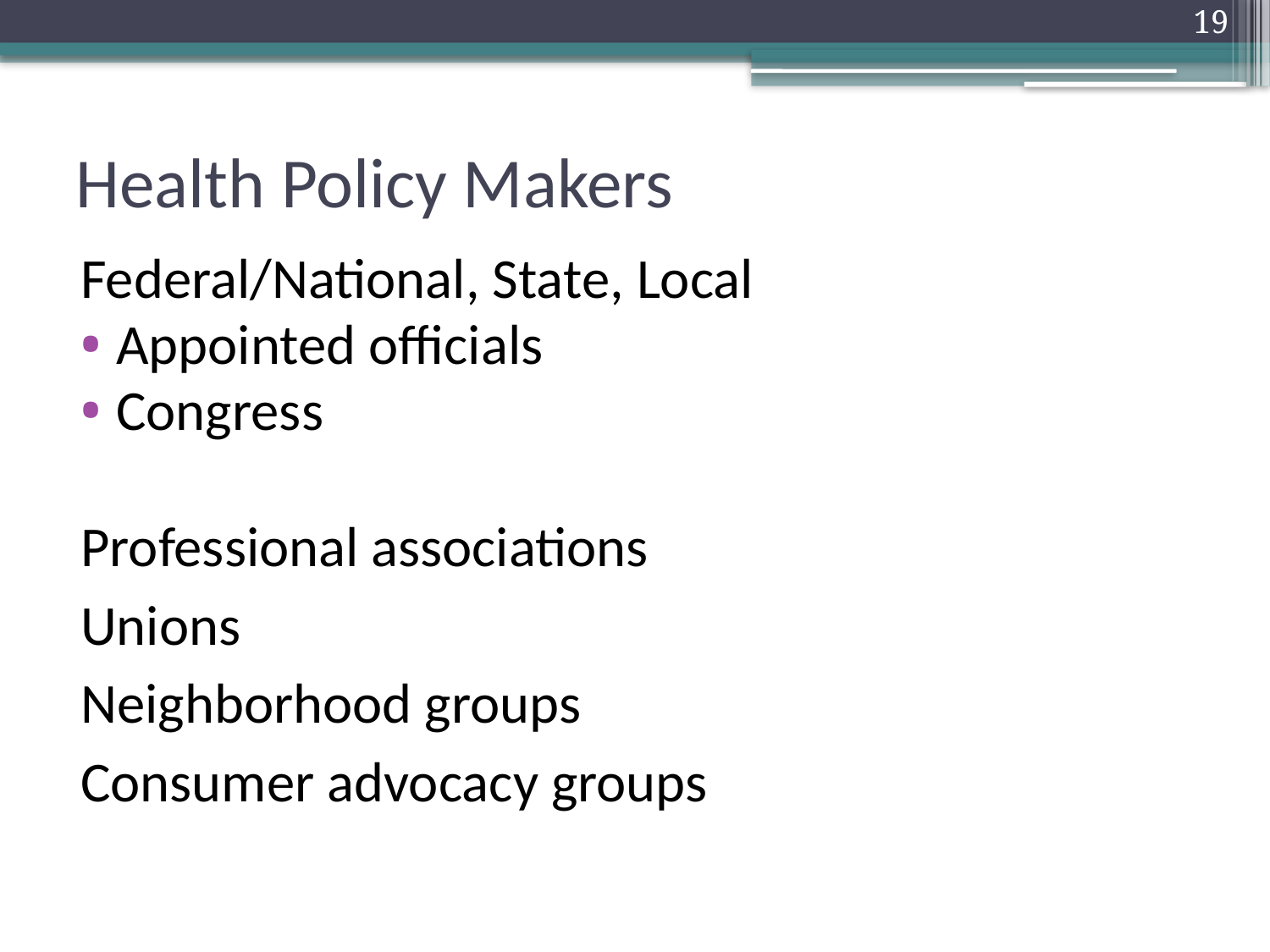

19
# Health Policy Makers
Federal/National, State, Local
Appointed officials
Congress
Professional associations
Unions
Neighborhood groups
Consumer advocacy groups

## Slide 20
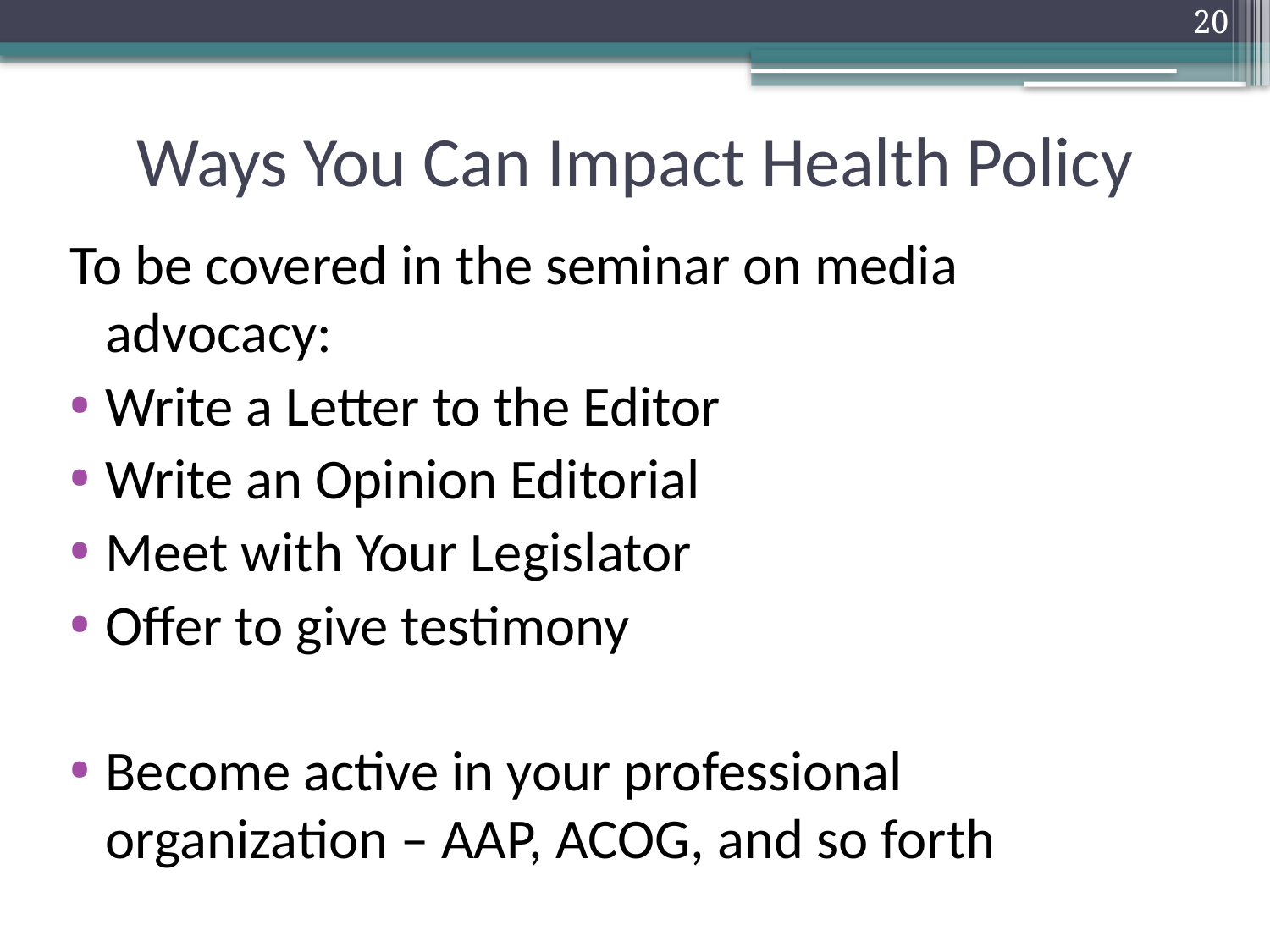

20
# Ways You Can Impact Health Policy
To be covered in the seminar on media advocacy:
Write a Letter to the Editor
Write an Opinion Editorial
Meet with Your Legislator
Offer to give testimony
Become active in your professional organization – AAP, ACOG, and so forth

## Slide 21
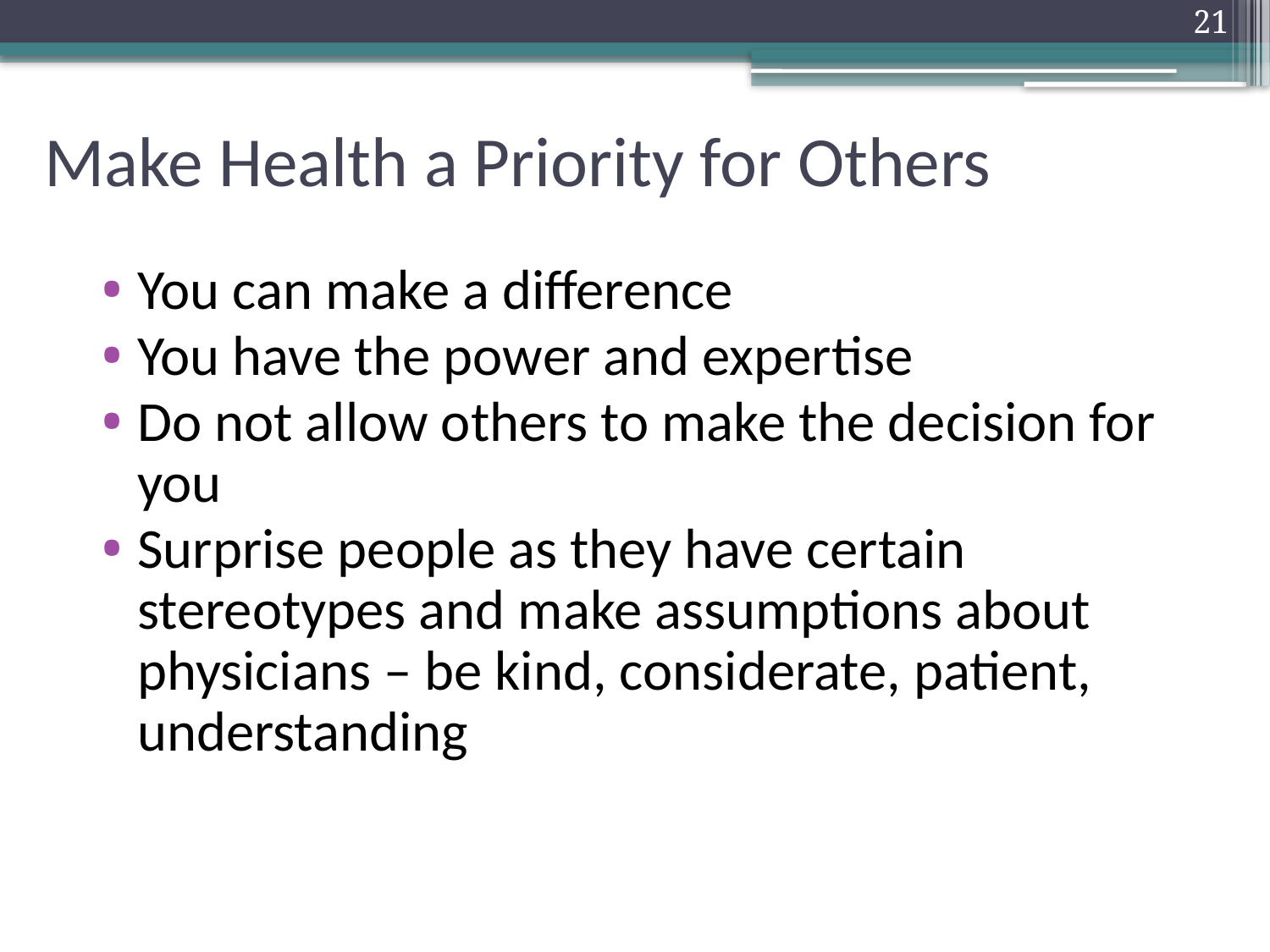

21
# Make Health a Priority for Others
You can make a difference
You have the power and expertise
Do not allow others to make the decision for you
Surprise people as they have certain stereotypes and make assumptions about physicians – be kind, considerate, patient, understanding

## Slide 22
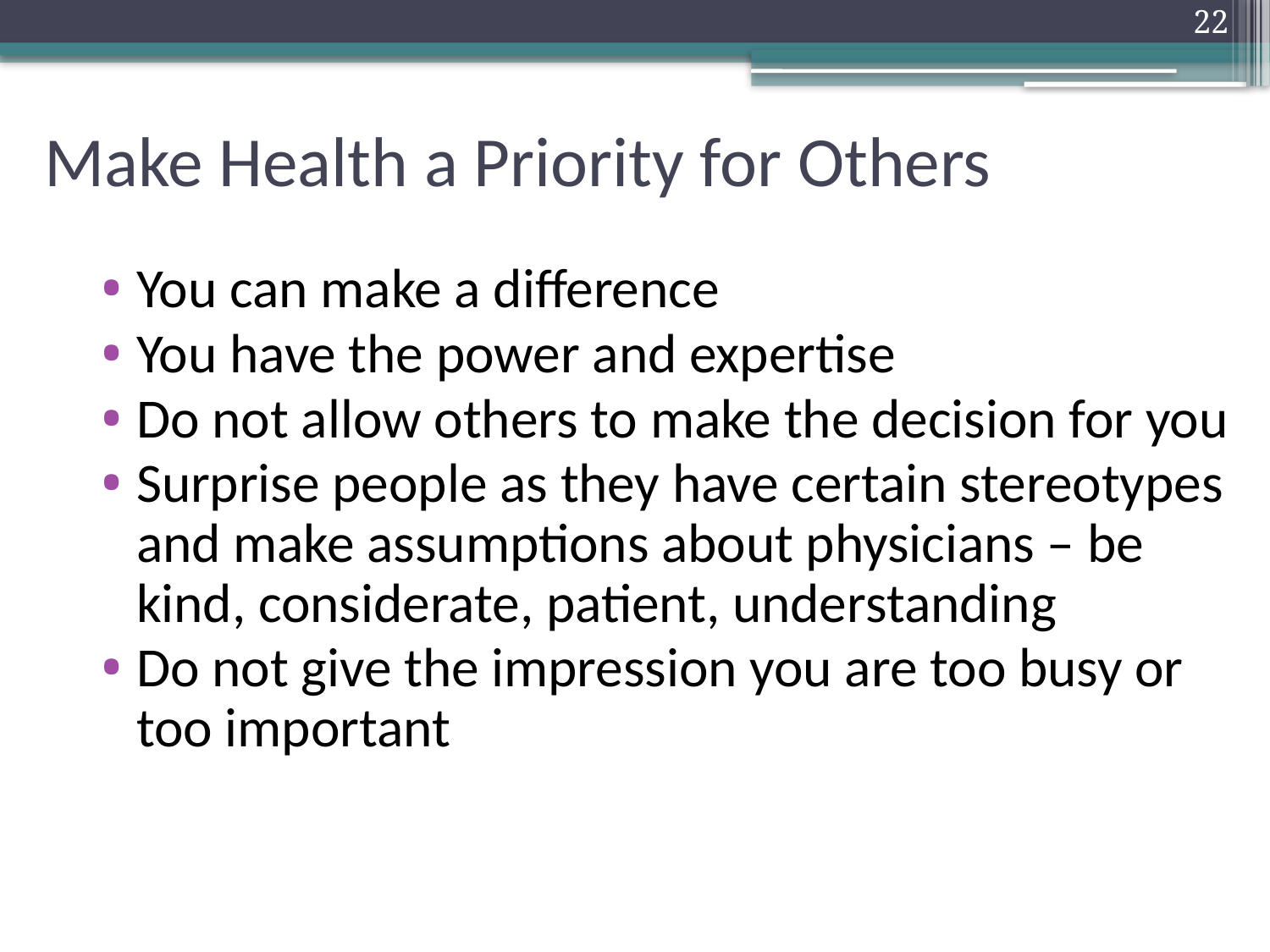

22
# Make Health a Priority for Others
You can make a difference
You have the power and expertise
Do not allow others to make the decision for you
Surprise people as they have certain stereotypes and make assumptions about physicians – be kind, considerate, patient, understanding
Do not give the impression you are too busy or too important

## Slide 23
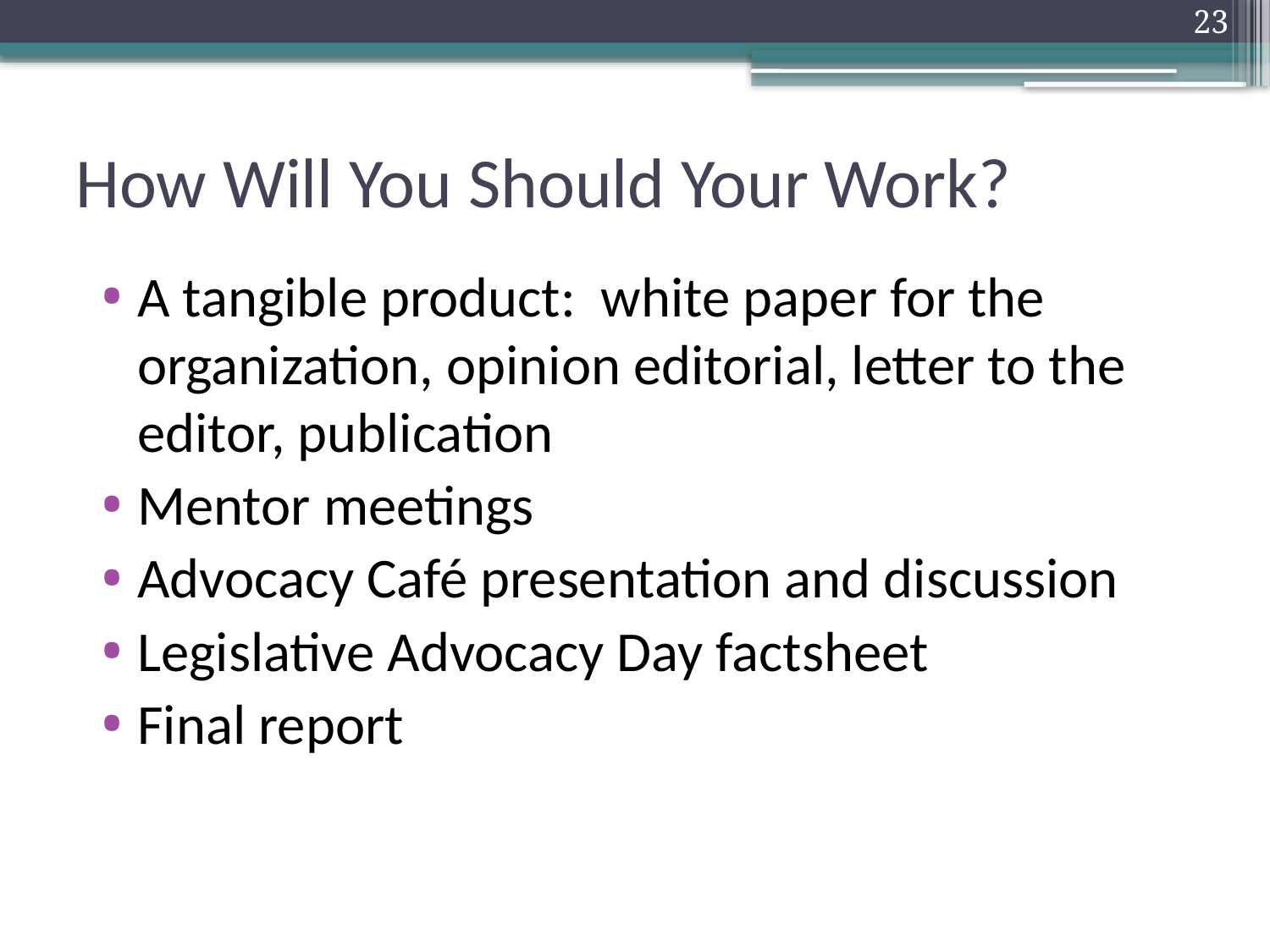

23
# How Will You Should Your Work?
A tangible product: white paper for the organization, opinion editorial, letter to the editor, publication
Mentor meetings
Advocacy Café presentation and discussion
Legislative Advocacy Day factsheet
Final report

## Slide 24
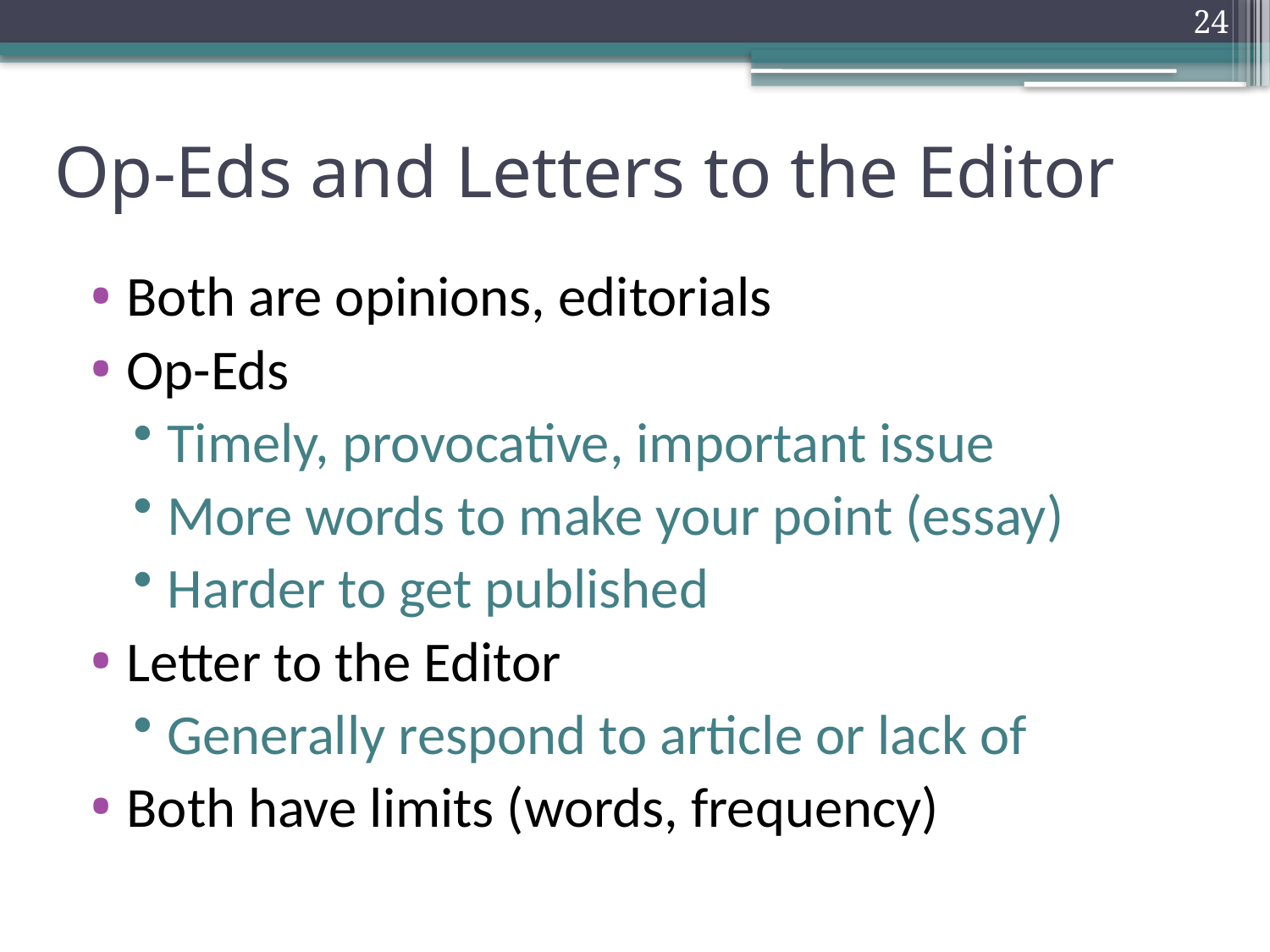

24
Op-Eds and Letters to the Editor
Both are opinions, editorials
Op-Eds
Timely, provocative, important issue
More words to make your point (essay)
Harder to get published
Letter to the Editor
Generally respond to article or lack of
Both have limits (words, frequency)

## Slide 25
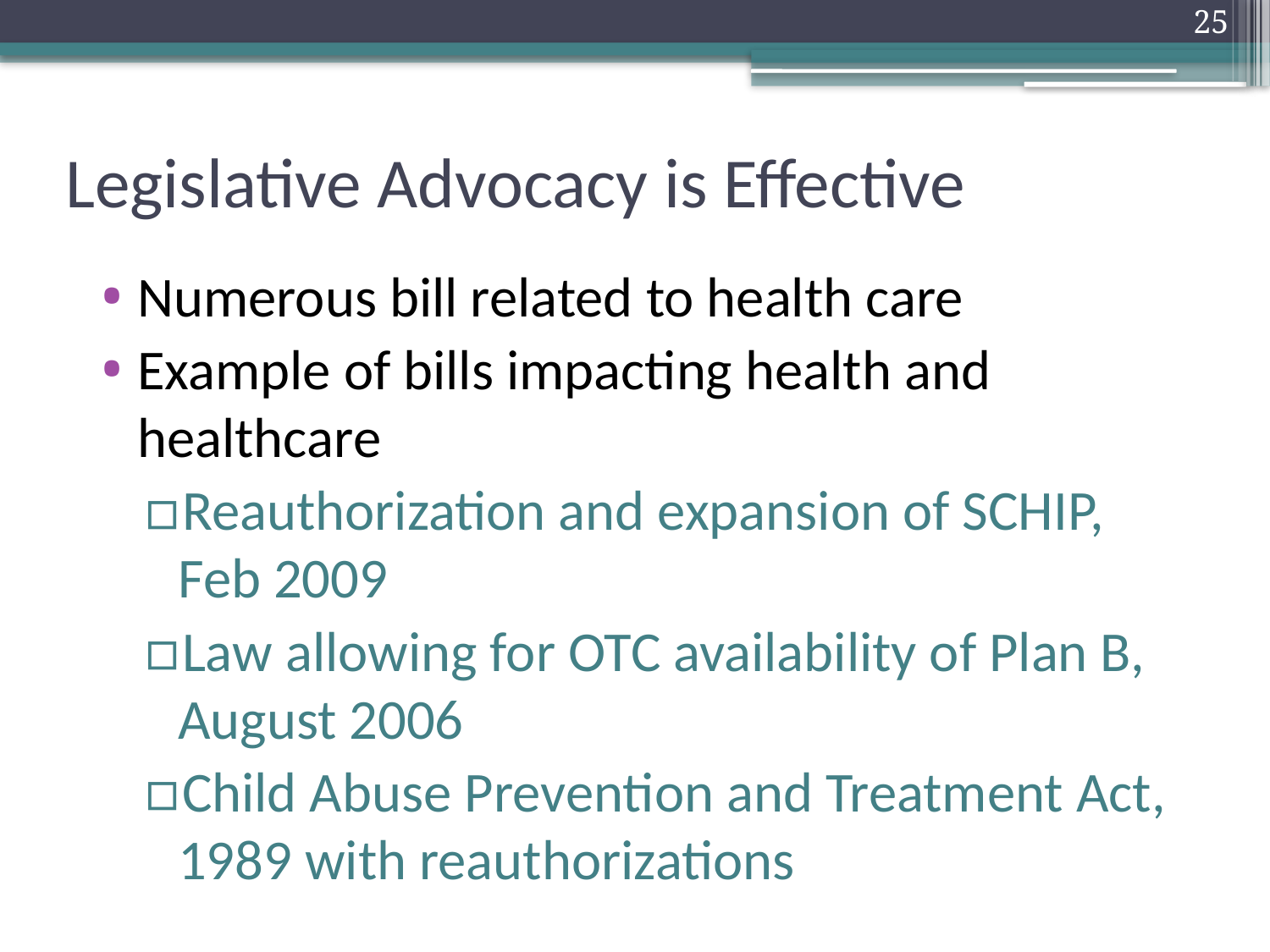

25
# Legislative Advocacy is Effective
Numerous bill related to health care
Example of bills impacting health and healthcare
Reauthorization and expansion of SCHIP, Feb 2009
Law allowing for OTC availability of Plan B, August 2006
Child Abuse Prevention and Treatment Act, 1989 with reauthorizations

## Slide 26
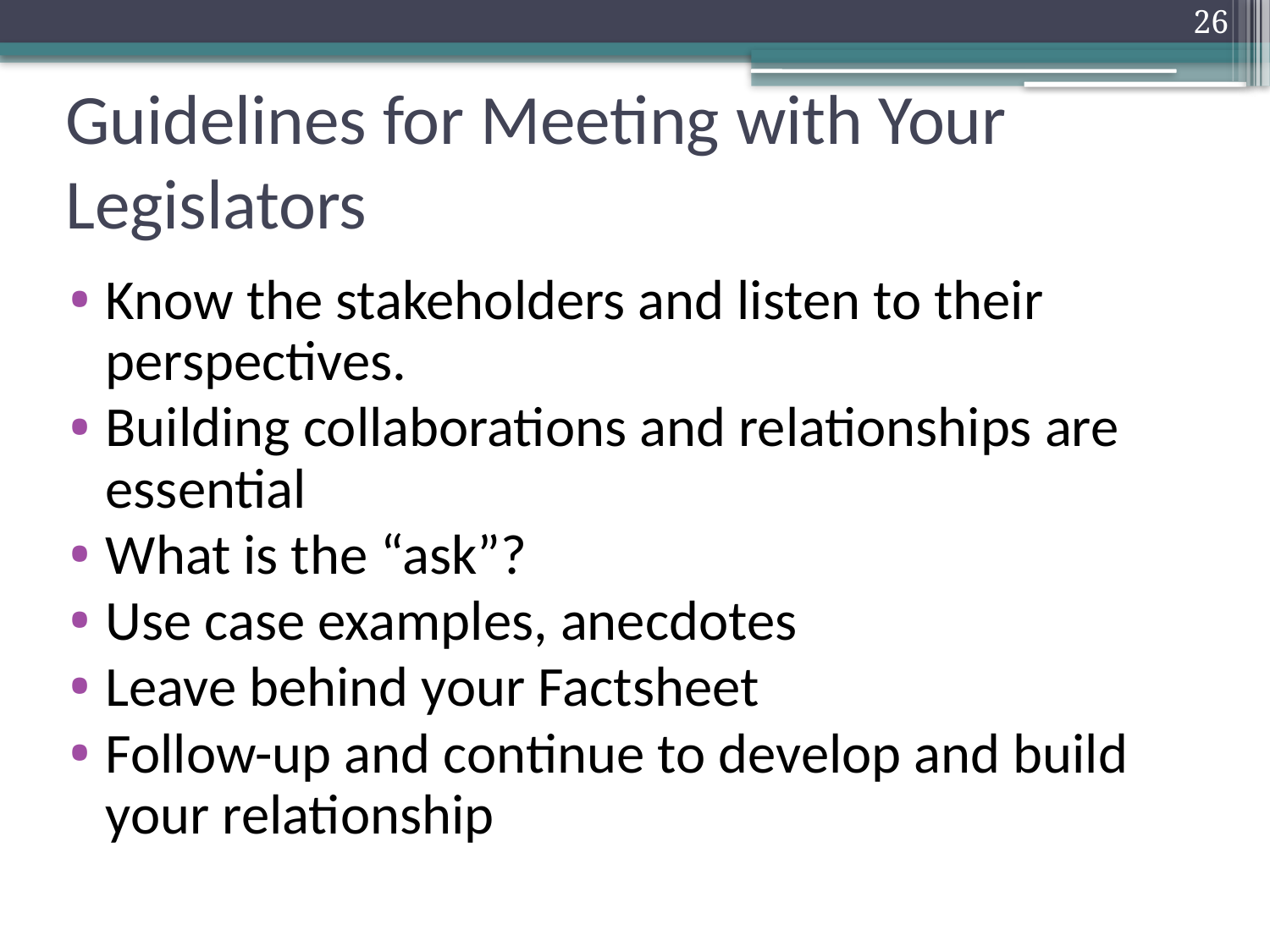

26
# Guidelines for Meeting with Your Legislators
Know the stakeholders and listen to their perspectives.
Building collaborations and relationships are essential
What is the “ask”?
Use case examples, anecdotes
Leave behind your Factsheet
Follow-up and continue to develop and build your relationship

## Slide 27
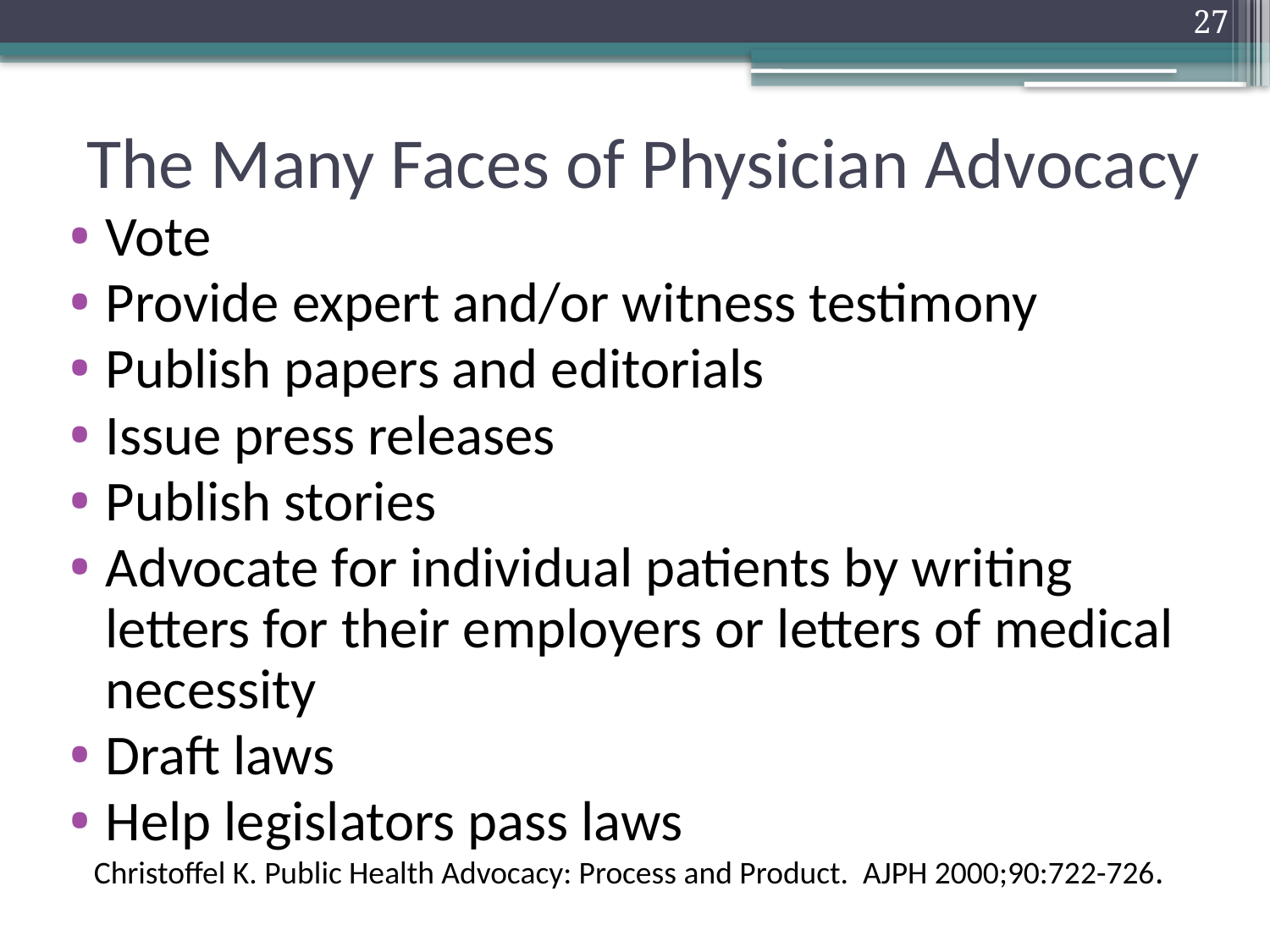

27
# The Many Faces of Physician Advocacy
Vote
Provide expert and/or witness testimony
Publish papers and editorials
Issue press releases
Publish stories
Advocate for individual patients by writing letters for their employers or letters of medical necessity
Draft laws
Help legislators pass laws
 Christoffel K. Public Health Advocacy: Process and Product. AJPH 2000;90:722-726.

## Slide 28
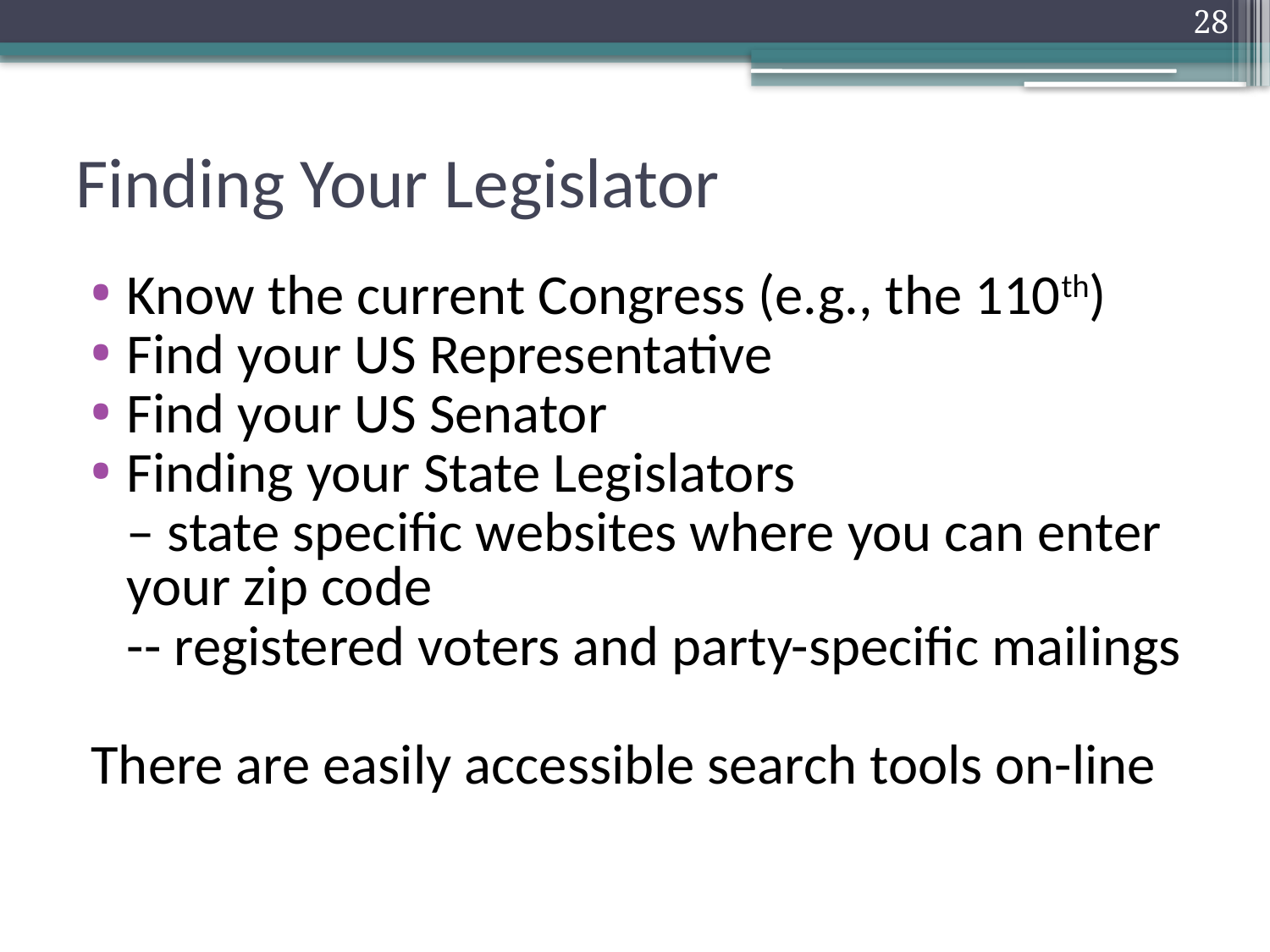

28
# Finding Your Legislator
Know the current Congress (e.g., the 110th)
Find your US Representative
Find your US Senator
Finding your State Legislators
	– state specific websites where you can enter your zip code
	-- registered voters and party-specific mailings
There are easily accessible search tools on-line

## Slide 29
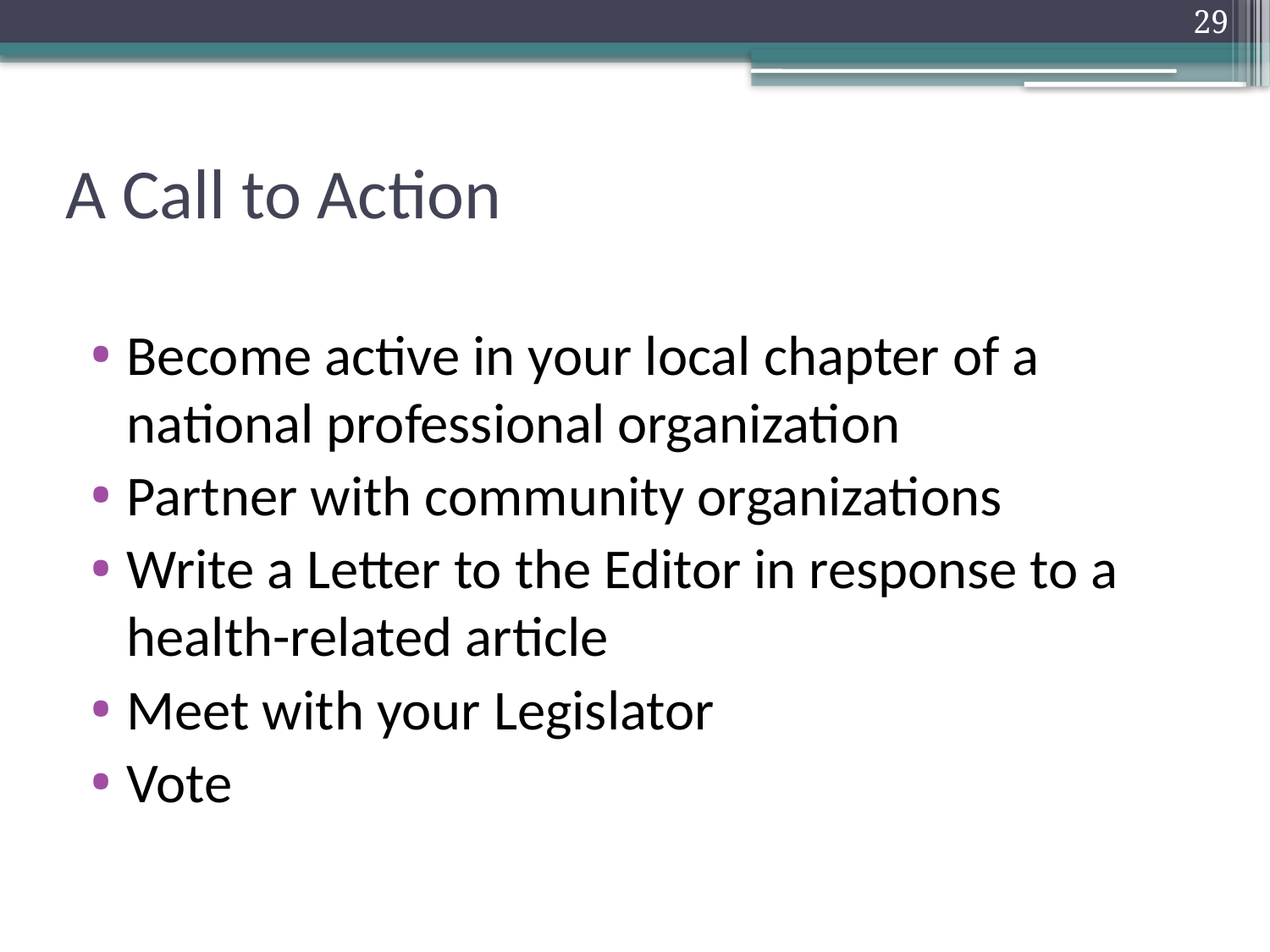

29
# A Call to Action
Become active in your local chapter of a national professional organization
Partner with community organizations
Write a Letter to the Editor in response to a health-related article
Meet with your Legislator
Vote

## Slide 30
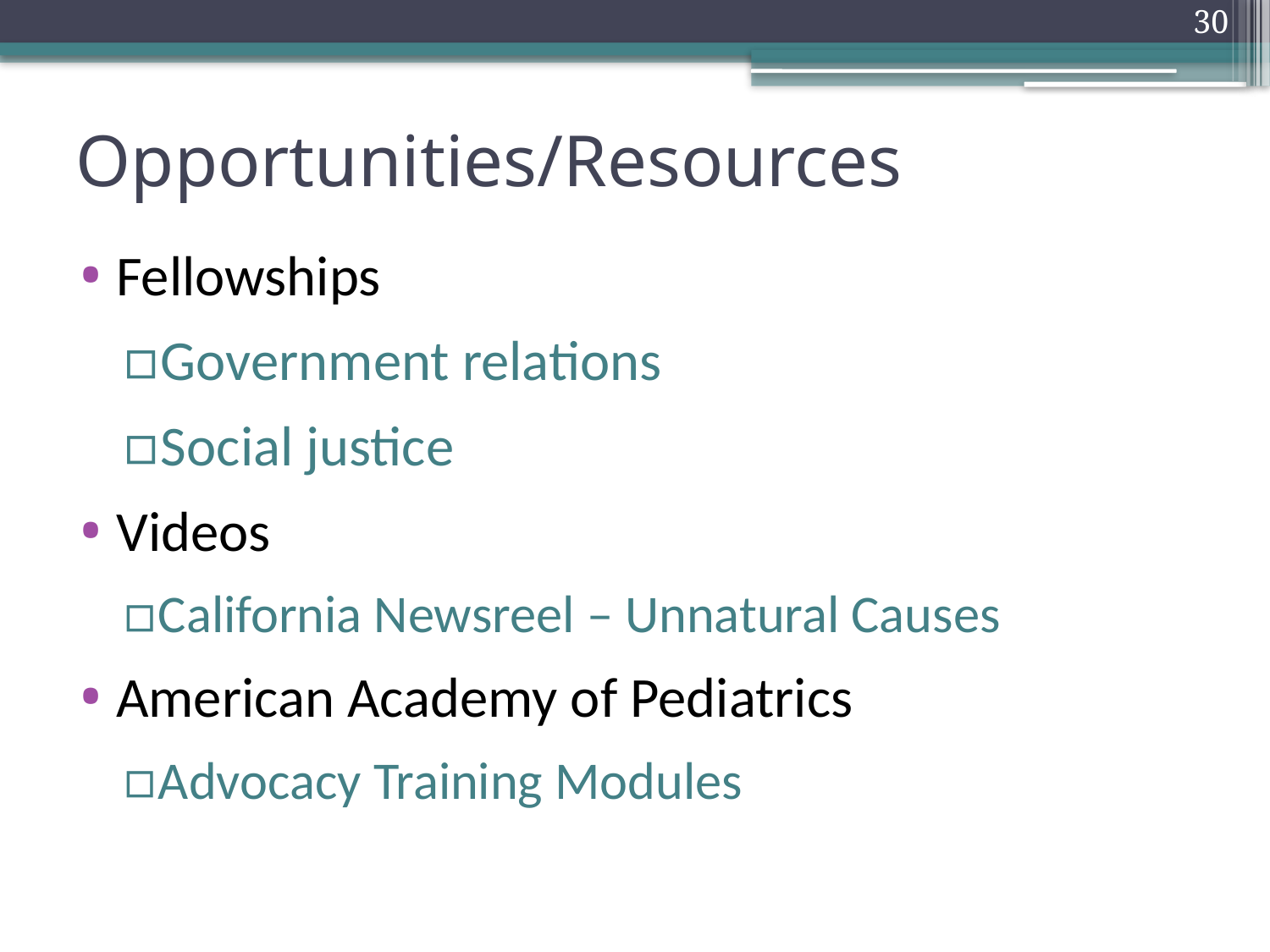

30
# Opportunities/Resources
Fellowships
Government relations
Social justice
Videos
California Newsreel – Unnatural Causes
American Academy of Pediatrics
Advocacy Training Modules
