# Supplementary material for: The JeffSTARS Advocacy and Community Partnership Elective: A Closer Look at Child Health Advocacy in Action
Source: MedEdPORTAL. 2016 Dec 31;12:10526. doi: 10.15766/mep_2374-8265.10526 (PMC6365684; doi:10.15766/mep_2374-8265.10526)
Supplement: Supplementary file 1 — A. CM1. Course Implementation at New Institution Checklist.docx B. CM2. Elective Checklist.docx C. CM3. Sample Schedule.docx D. CM4. Seminar Topic List With Learning Objectives.docx E. CM5. Syllabus Bibliography.docx F. CM6. List of Community Partners.docx G. CM7. Orientation for New Community Partner.docx H. CM8. Selected Past Projects.docx I. CM9. Sample Fact Sheets for Legislative Visits.docx J. Seminar Materials folder K. ET1. Advocacy Elective Assessment 1.pdf L. ET2. Advocacy Elective Assessment 2.pdf M. ET3. Trainee Evaluation by Community or Faculty Mentor.docx N. ET4. Trainee Evaluation of Seminar.docx O. ET5. Trainee Evaluation of Community Partner.docx P. ET6. Final Report Template.docx Q. Selected Trainee Abstracts and Presented Results folder [file mep-12-10526-s001.zip › J._Seminar_Materials_folder/11._Institutional_Barriers.pptx]

## Slide 1
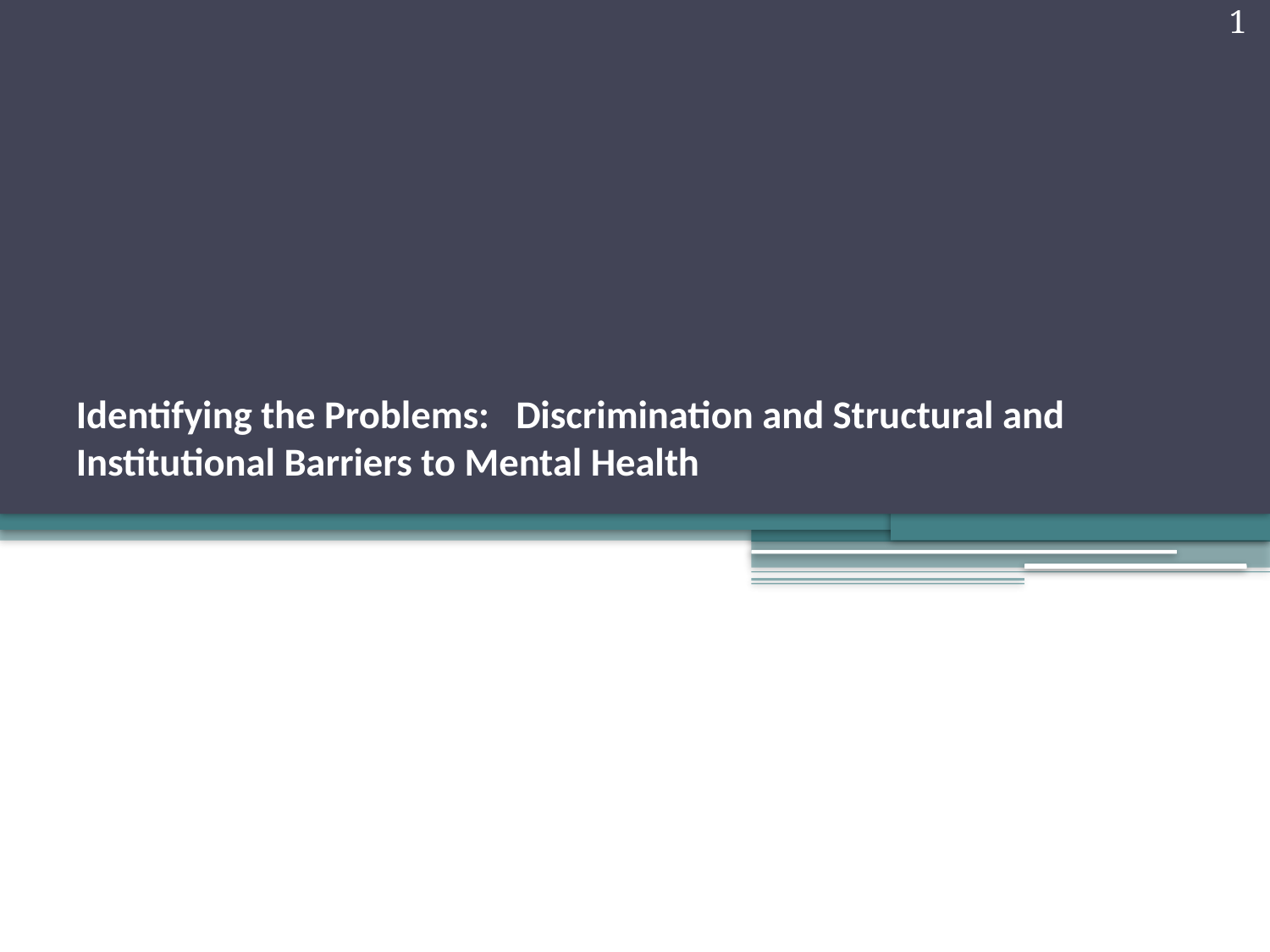

1
# Identifying the Problems: Discrimination and Structural and Institutional Barriers to Mental Health

## Slide 2
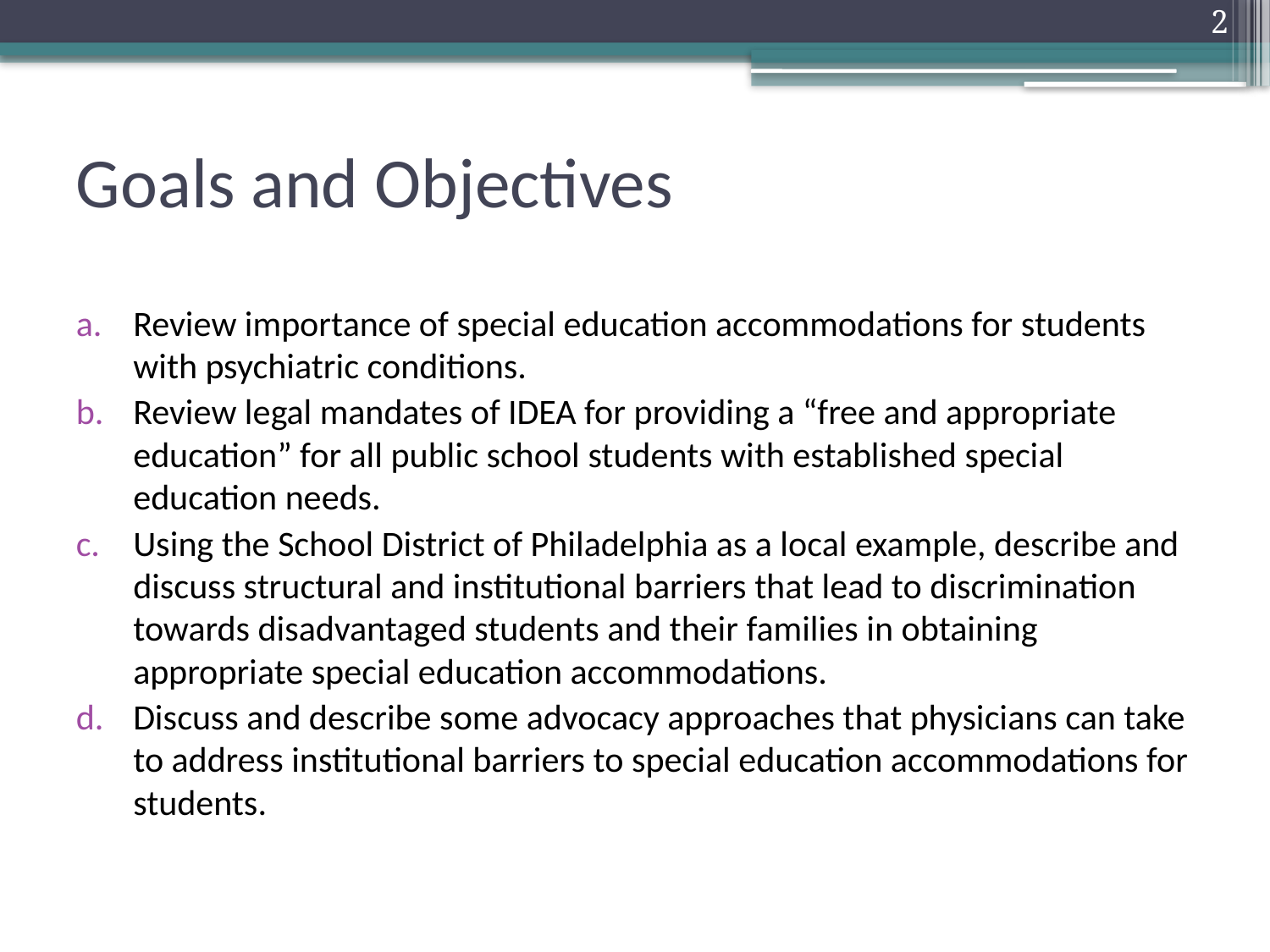

2
Goals and Objectives
Review importance of special education accommodations for students with psychiatric conditions.
Review legal mandates of IDEA for providing a “free and appropriate education” for all public school students with established special education needs.
Using the School District of Philadelphia as a local example, describe and discuss structural and institutional barriers that lead to discrimination towards disadvantaged students and their families in obtaining appropriate special education accommodations.
Discuss and describe some advocacy approaches that physicians can take to address institutional barriers to special education accommodations for students.

## Slide 3
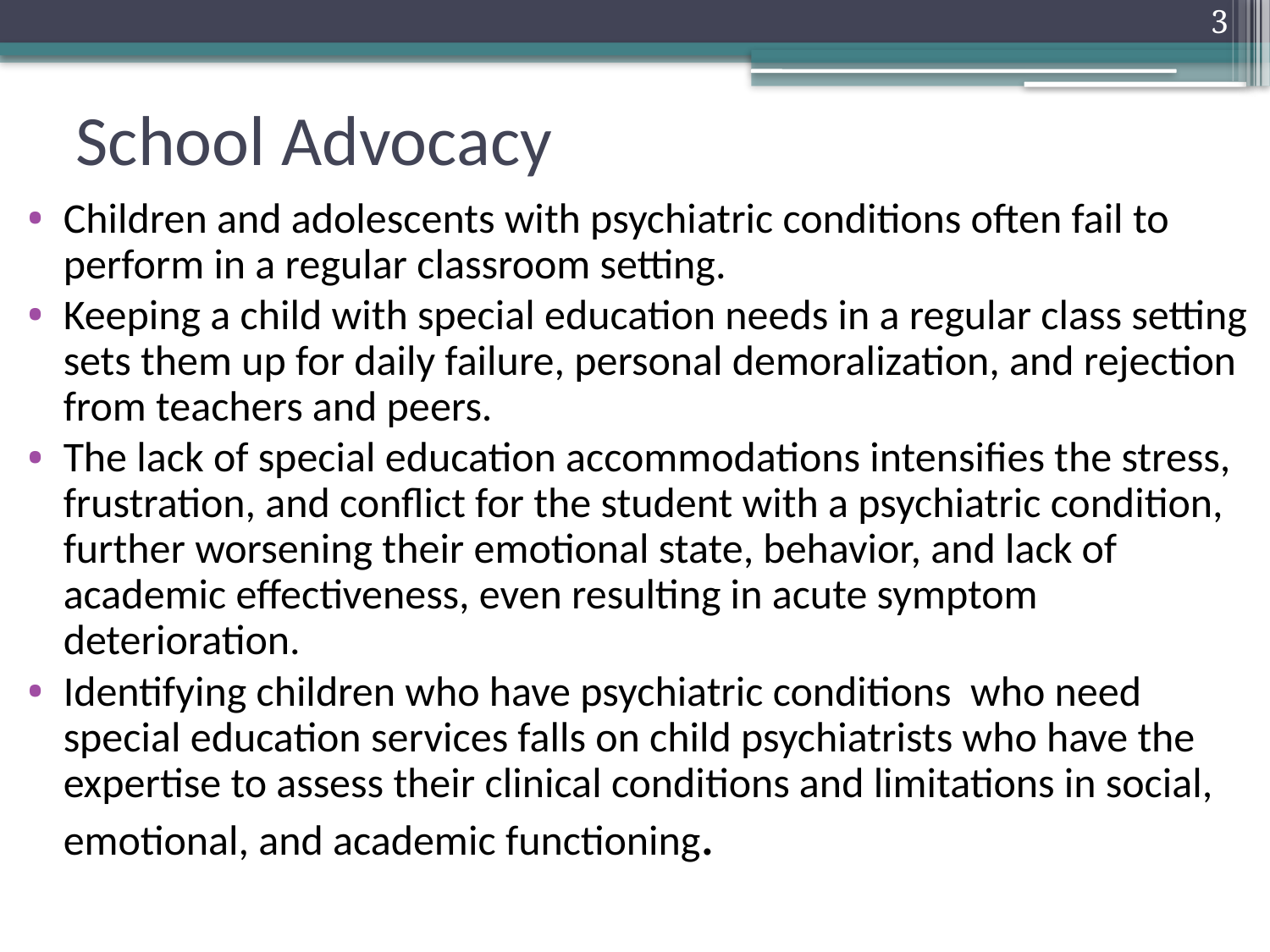

3
School Advocacy
Children and adolescents with psychiatric conditions often fail to perform in a regular classroom setting.
Keeping a child with special education needs in a regular class setting sets them up for daily failure, personal demoralization, and rejection from teachers and peers.
The lack of special education accommodations intensifies the stress, frustration, and conflict for the student with a psychiatric condition, further worsening their emotional state, behavior, and lack of academic effectiveness, even resulting in acute symptom deterioration.
Identifying children who have psychiatric conditions who need special education services falls on child psychiatrists who have the expertise to assess their clinical conditions and limitations in social, emotional, and academic functioning.

## Slide 4
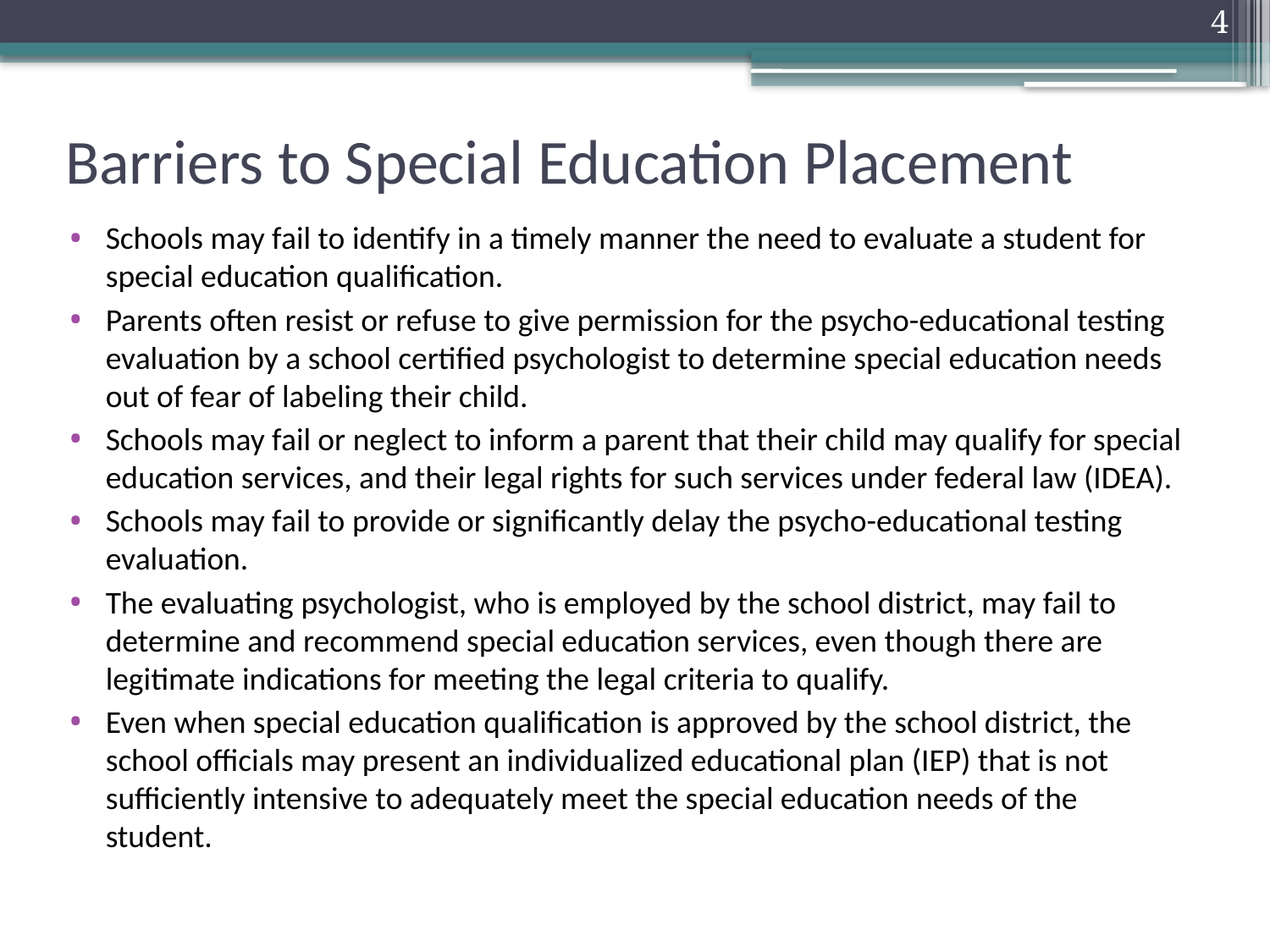

4
Barriers to Special Education Placement
Schools may fail to identify in a timely manner the need to evaluate a student for special education qualification.
Parents often resist or refuse to give permission for the psycho-educational testing evaluation by a school certified psychologist to determine special education needs out of fear of labeling their child.
Schools may fail or neglect to inform a parent that their child may qualify for special education services, and their legal rights for such services under federal law (IDEA).
Schools may fail to provide or significantly delay the psycho-educational testing evaluation.
The evaluating psychologist, who is employed by the school district, may fail to determine and recommend special education services, even though there are legitimate indications for meeting the legal criteria to qualify.
Even when special education qualification is approved by the school district, the school officials may present an individualized educational plan (IEP) that is not sufficiently intensive to adequately meet the special education needs of the student.

## Slide 5
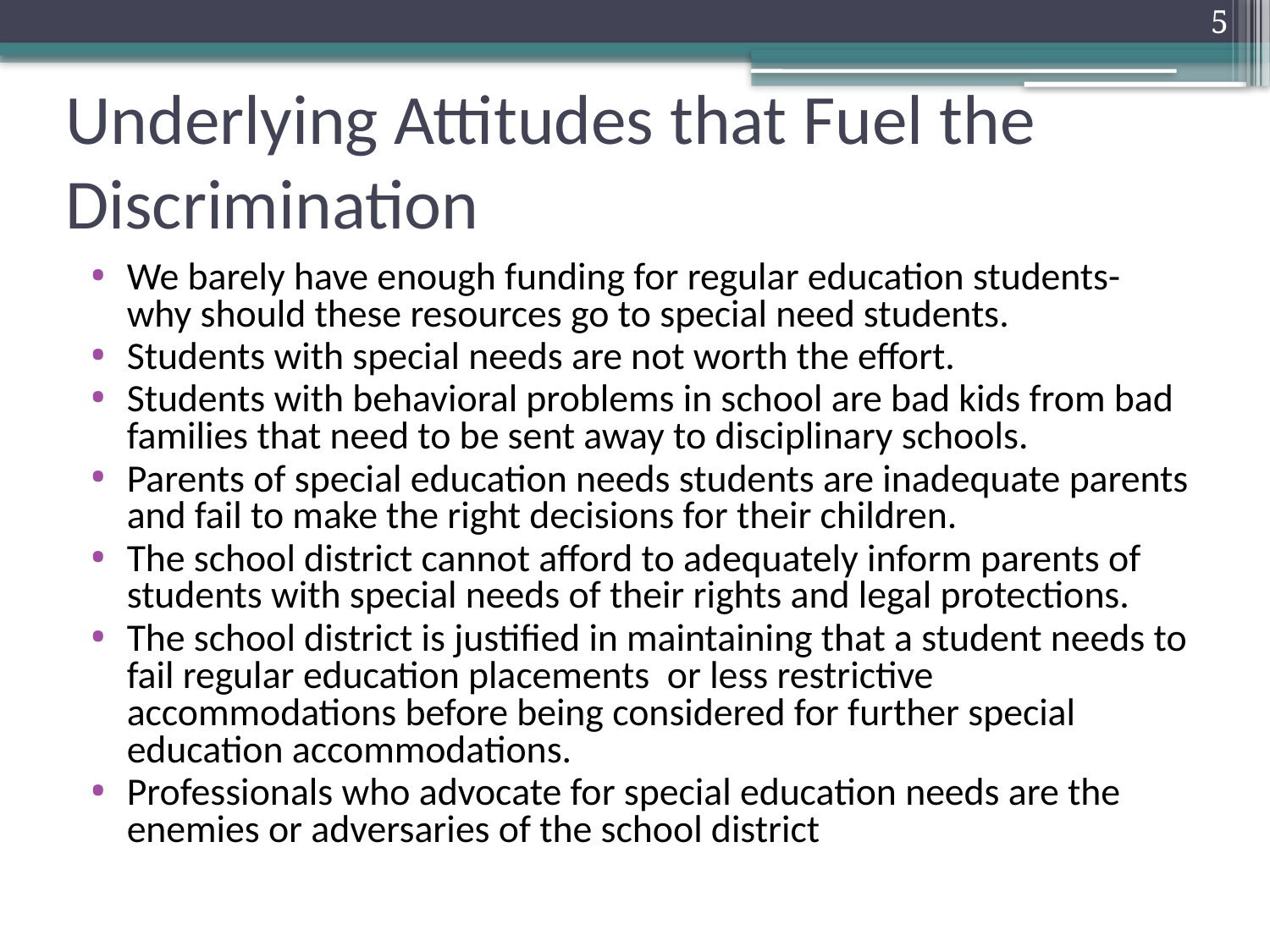

5
Underlying Attitudes that Fuel the Discrimination
We barely have enough funding for regular education students- why should these resources go to special need students.
Students with special needs are not worth the effort.
Students with behavioral problems in school are bad kids from bad families that need to be sent away to disciplinary schools.
Parents of special education needs students are inadequate parents and fail to make the right decisions for their children.
The school district cannot afford to adequately inform parents of students with special needs of their rights and legal protections.
The school district is justified in maintaining that a student needs to fail regular education placements or less restrictive accommodations before being considered for further special education accommodations.
Professionals who advocate for special education needs are the enemies or adversaries of the school district

## Slide 6
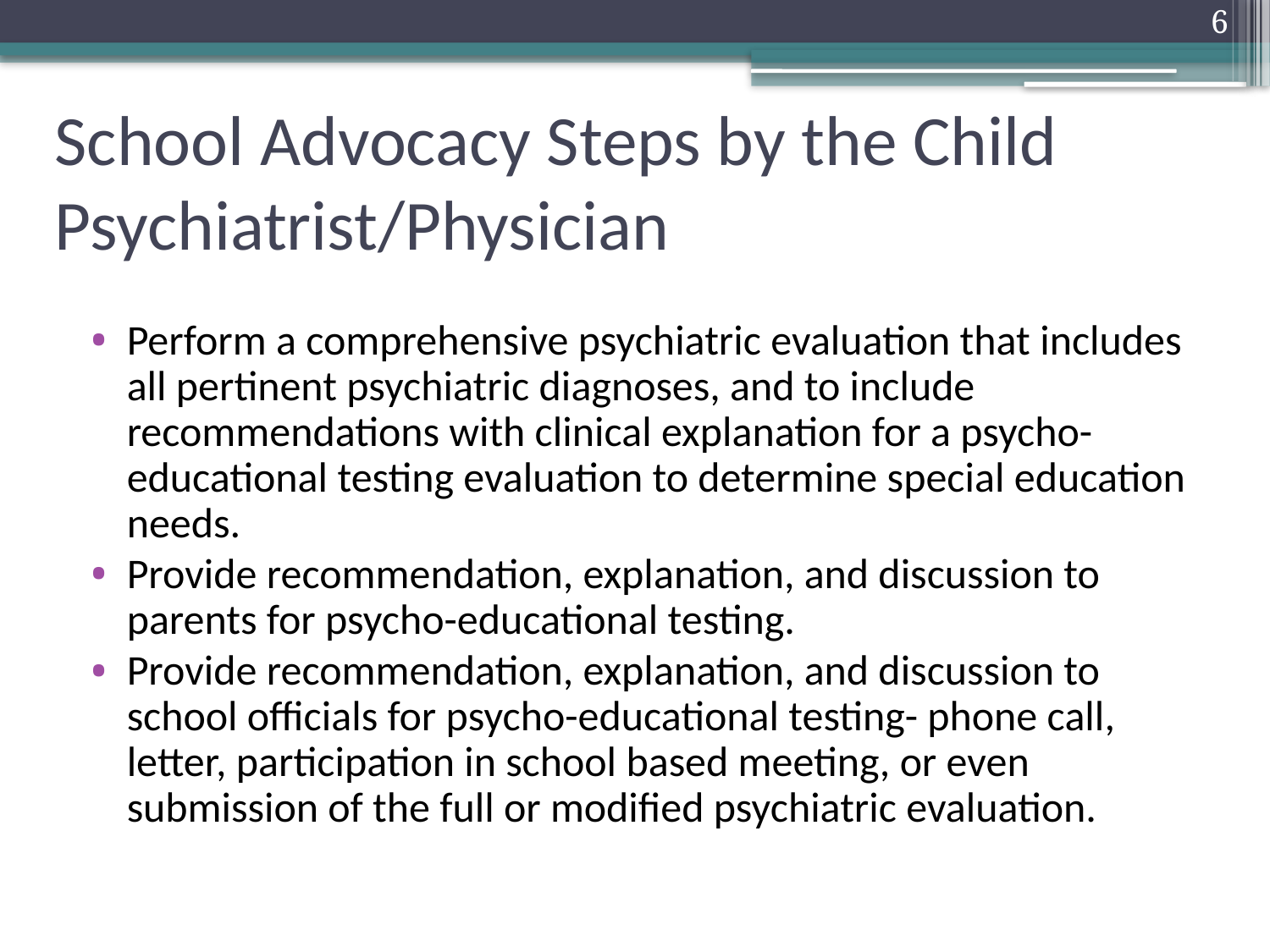

Perform a comprehensive psychiatric evaluation that includes all pertinent psychiatric diagnoses, and to include recommendations with clinical explanation for a psycho-educational testing evaluation to determine special education needs.
Provide recommendation, explanation, and discussion to parents for psycho-educational testing.
Provide recommendation, explanation, and discussion to school officials for psycho-educational testing- phone call, letter, participation in school based meeting, or even submission of the full or modified psychiatric evaluation.
6
School Advocacy Steps by the Child Psychiatrist/Physician

## Slide 7
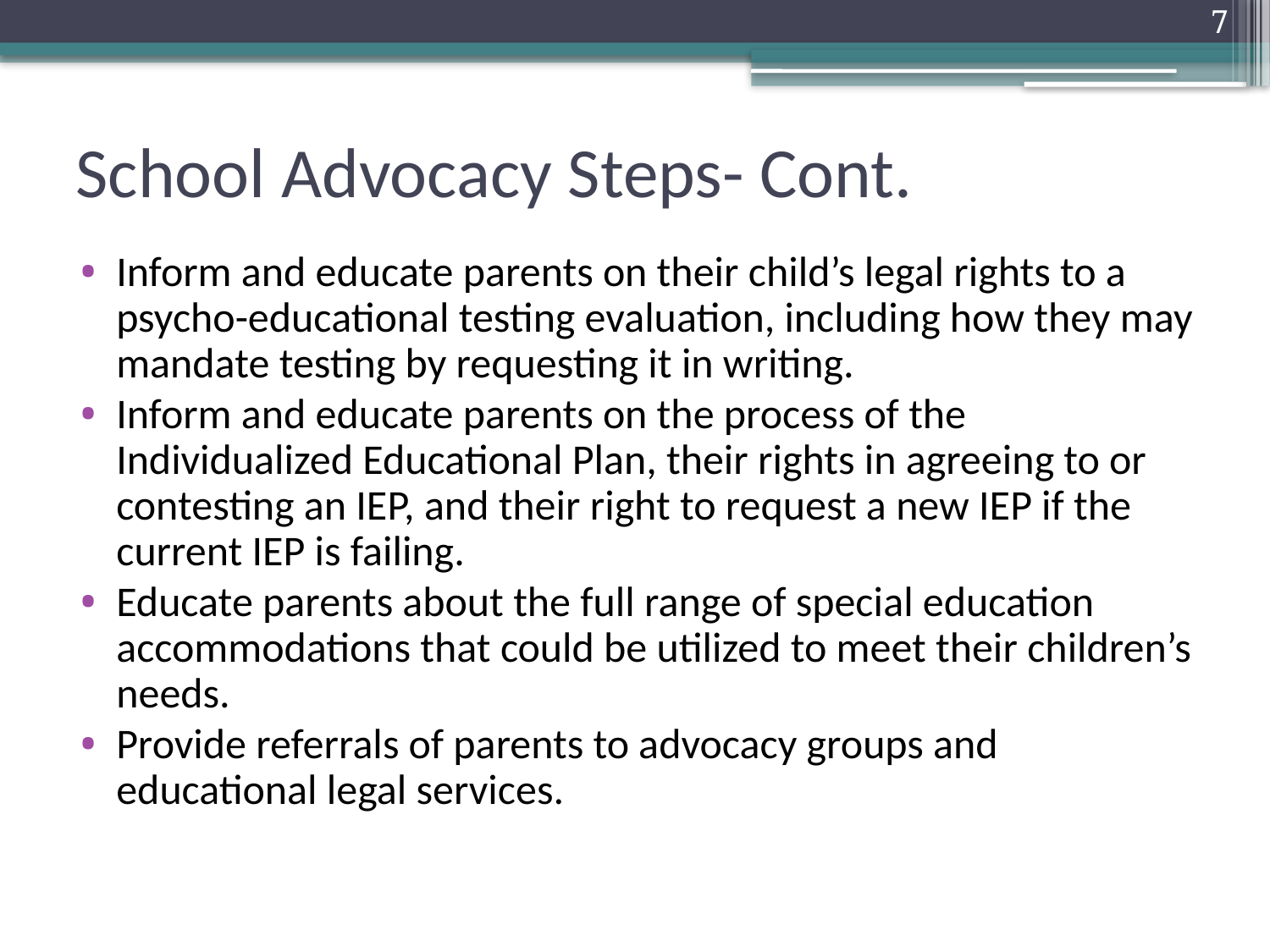

7
School Advocacy Steps- Cont.
Inform and educate parents on their child’s legal rights to a psycho-educational testing evaluation, including how they may mandate testing by requesting it in writing.
Inform and educate parents on the process of the Individualized Educational Plan, their rights in agreeing to or contesting an IEP, and their right to request a new IEP if the current IEP is failing.
Educate parents about the full range of special education accommodations that could be utilized to meet their children’s needs.
Provide referrals of parents to advocacy groups and educational legal services.

## Slide 8
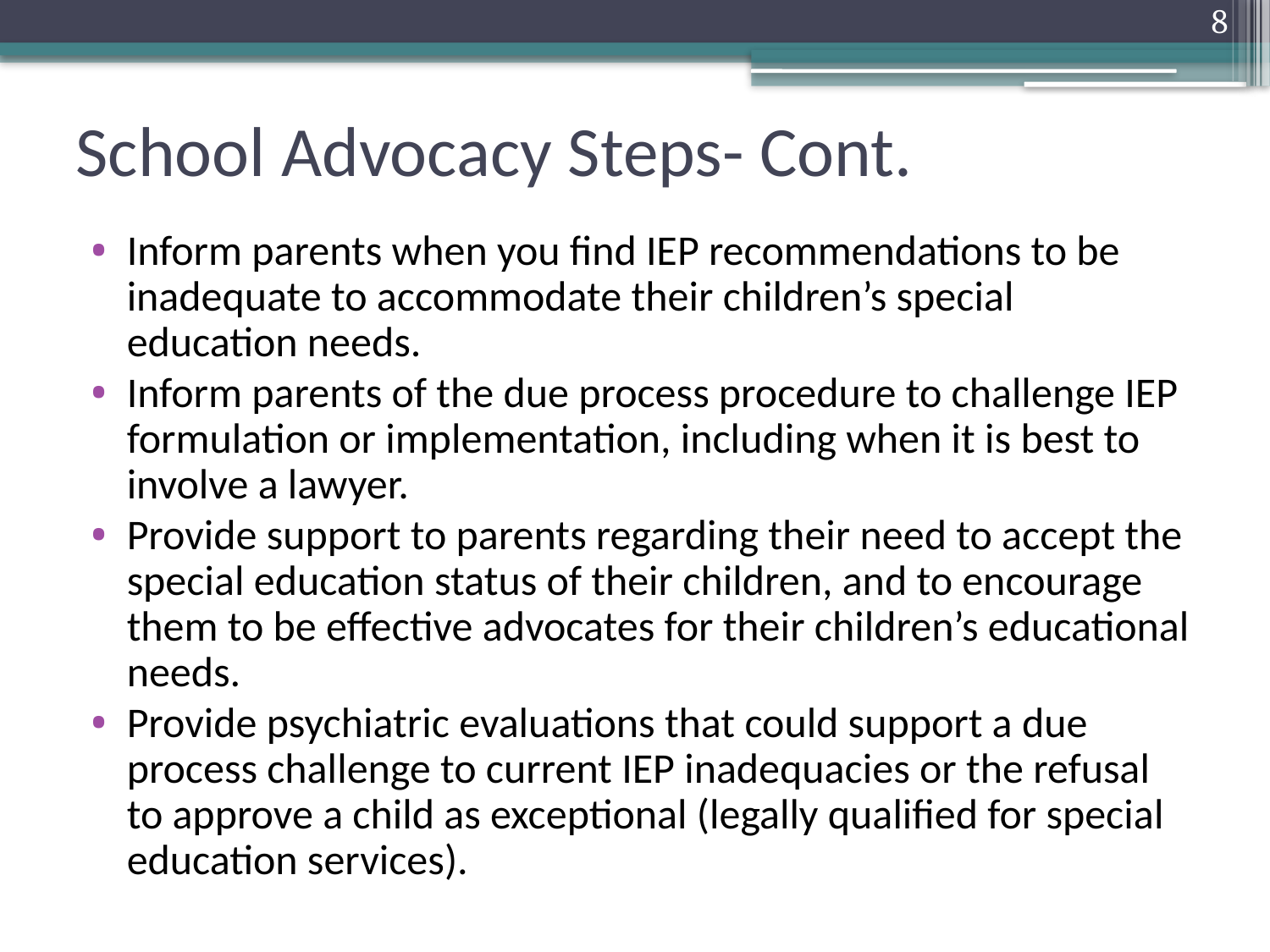

8
School Advocacy Steps- Cont.
Inform parents when you find IEP recommendations to be inadequate to accommodate their children’s special education needs.
Inform parents of the due process procedure to challenge IEP formulation or implementation, including when it is best to involve a lawyer.
Provide support to parents regarding their need to accept the special education status of their children, and to encourage them to be effective advocates for their children’s educational needs.
Provide psychiatric evaluations that could support a due process challenge to current IEP inadequacies or the refusal to approve a child as exceptional (legally qualified for special education services).

## Slide 9
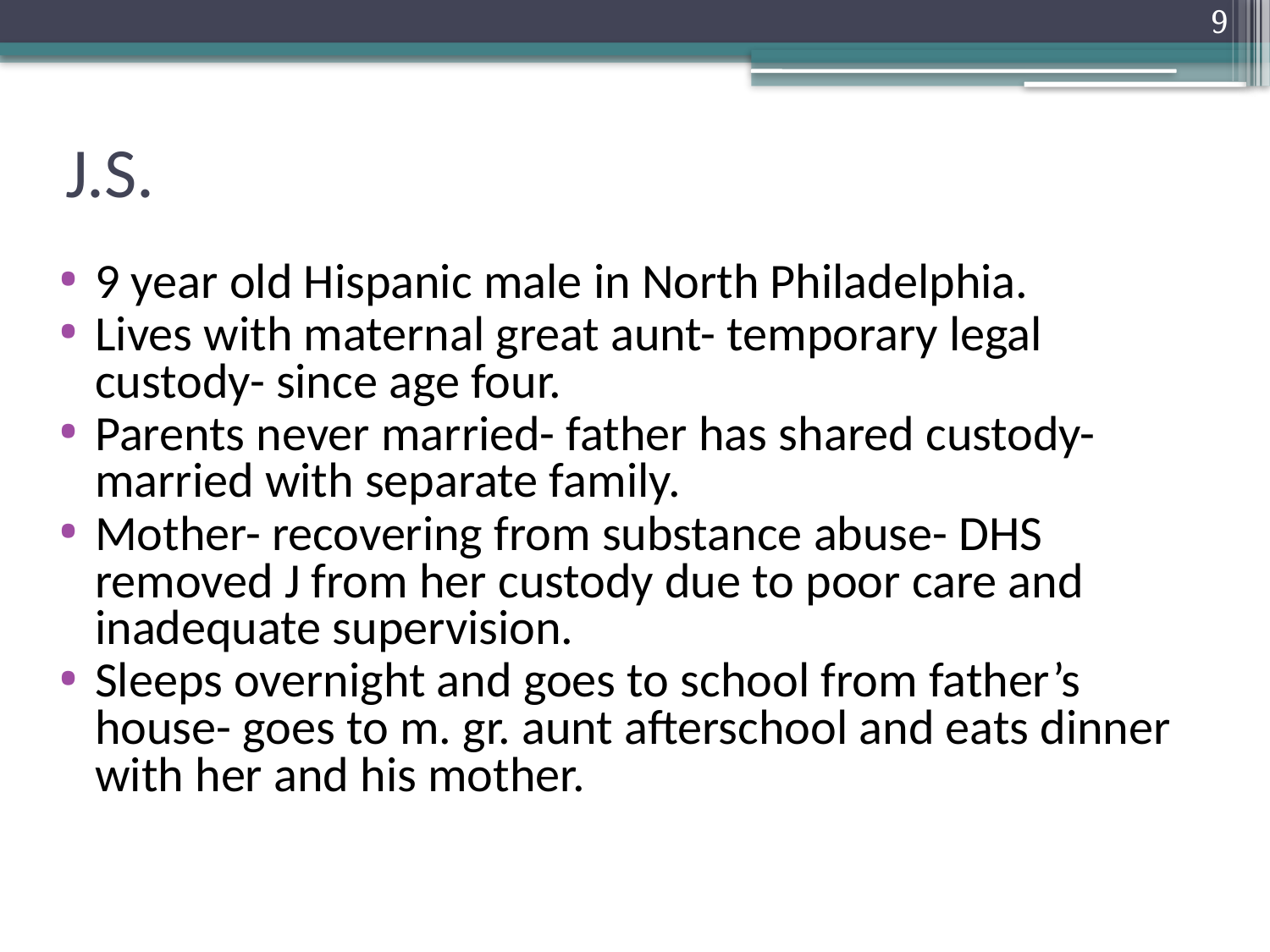

9
J.S.
9 year old Hispanic male in North Philadelphia.
Lives with maternal great aunt- temporary legal custody- since age four.
Parents never married- father has shared custody- married with separate family.
Mother- recovering from substance abuse- DHS removed J from her custody due to poor care and inadequate supervision.
Sleeps overnight and goes to school from father’s house- goes to m. gr. aunt afterschool and eats dinner with her and his mother.

## Slide 10
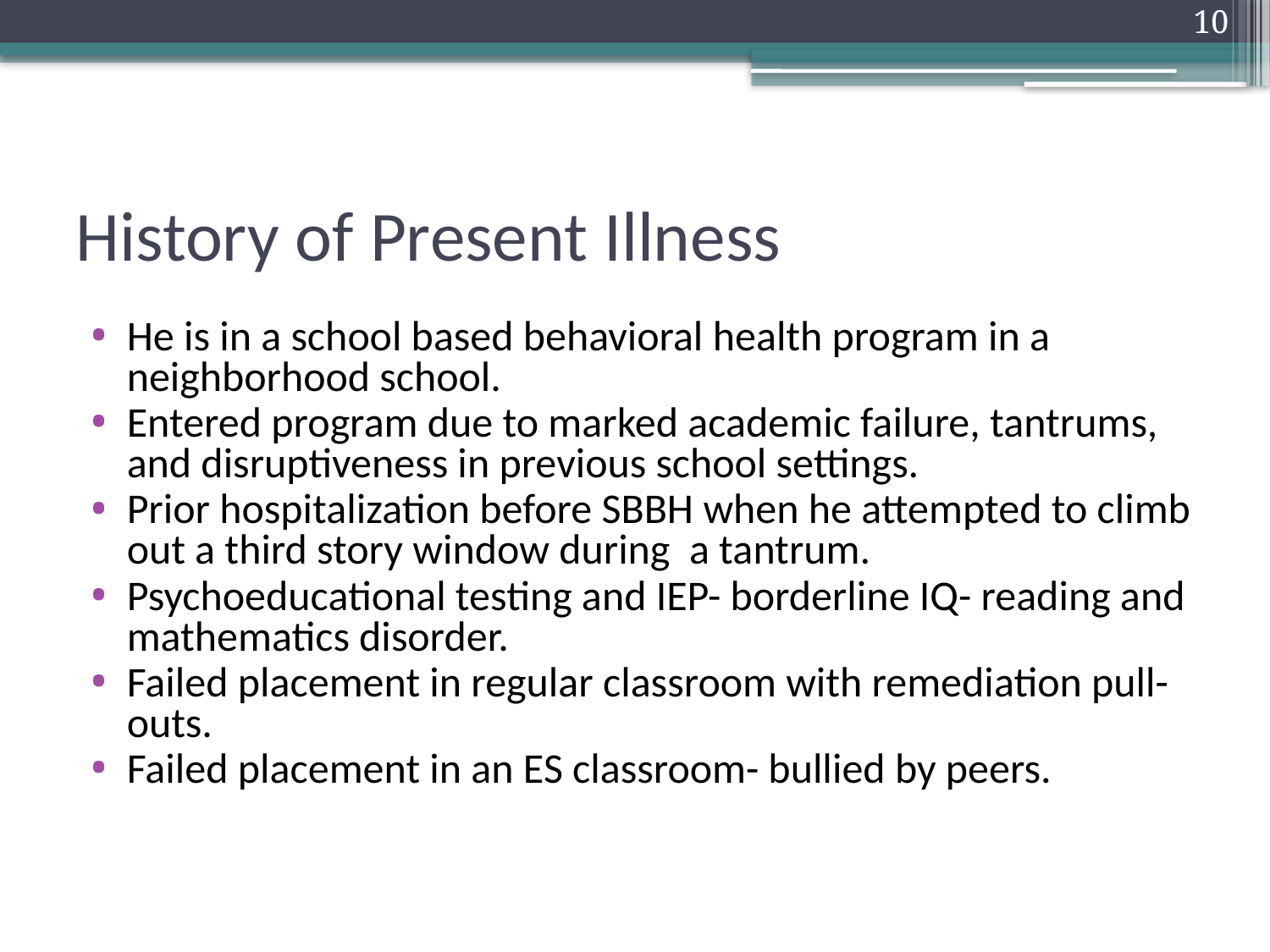

History of Present Illness
He is in a school based behavioral health program in a neighborhood school.
Entered program due to marked academic failure, tantrums, and disruptiveness in previous school settings.
Prior hospitalization before SBBH when he attempted to climb out a third story window during a tantrum.
Psychoeducational testing and IEP- borderline IQ- reading and mathematics disorder.
Failed placement in regular classroom with remediation pull-outs.
Failed placement in an ES classroom- bullied by peers.
10

## Slide 11
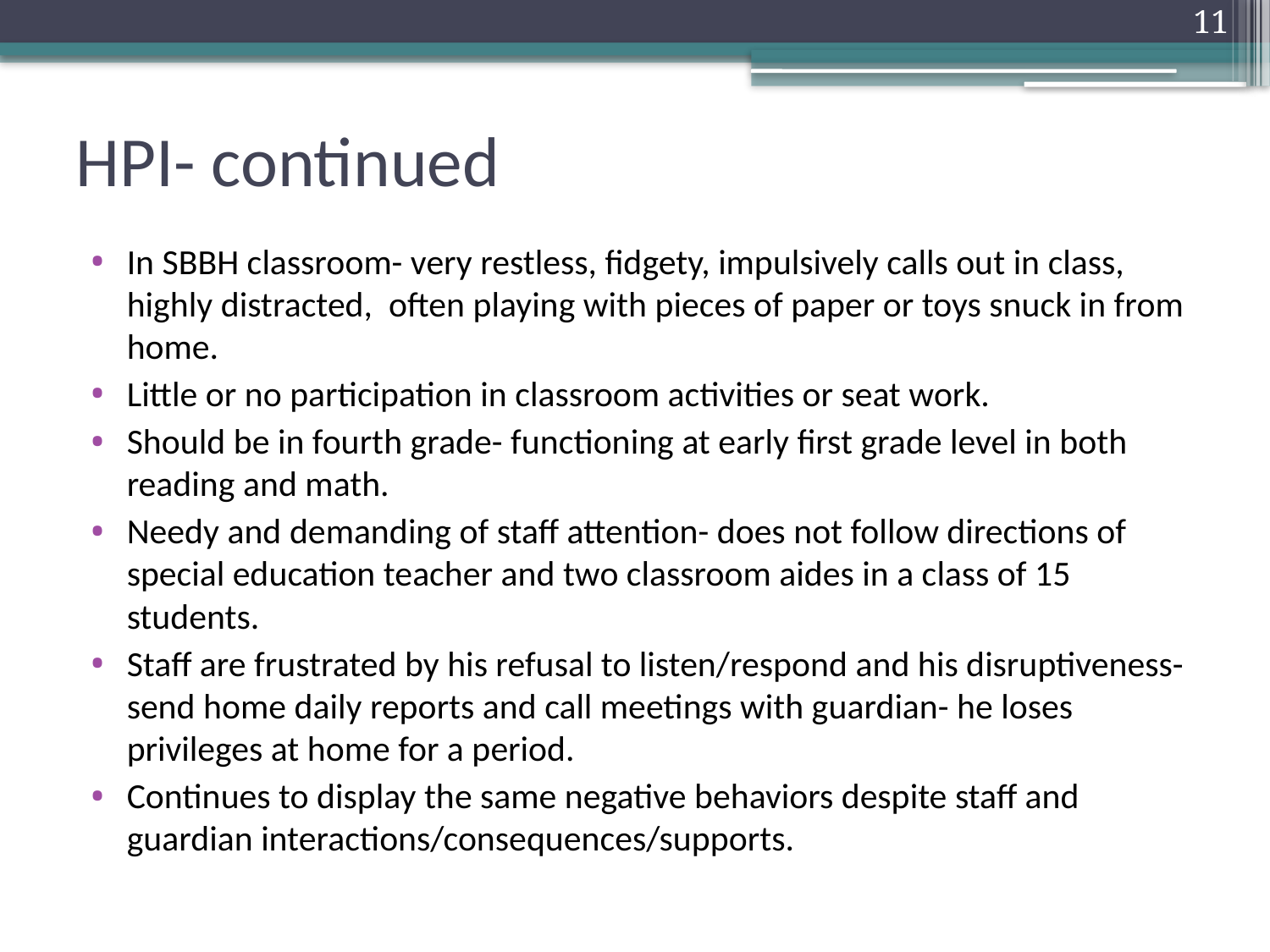

11
HPI- continued
In SBBH classroom- very restless, fidgety, impulsively calls out in class, highly distracted, often playing with pieces of paper or toys snuck in from home.
Little or no participation in classroom activities or seat work.
Should be in fourth grade- functioning at early first grade level in both reading and math.
Needy and demanding of staff attention- does not follow directions of special education teacher and two classroom aides in a class of 15 students.
Staff are frustrated by his refusal to listen/respond and his disruptiveness- send home daily reports and call meetings with guardian- he loses privileges at home for a period.
Continues to display the same negative behaviors despite staff and guardian interactions/consequences/supports.

## Slide 12
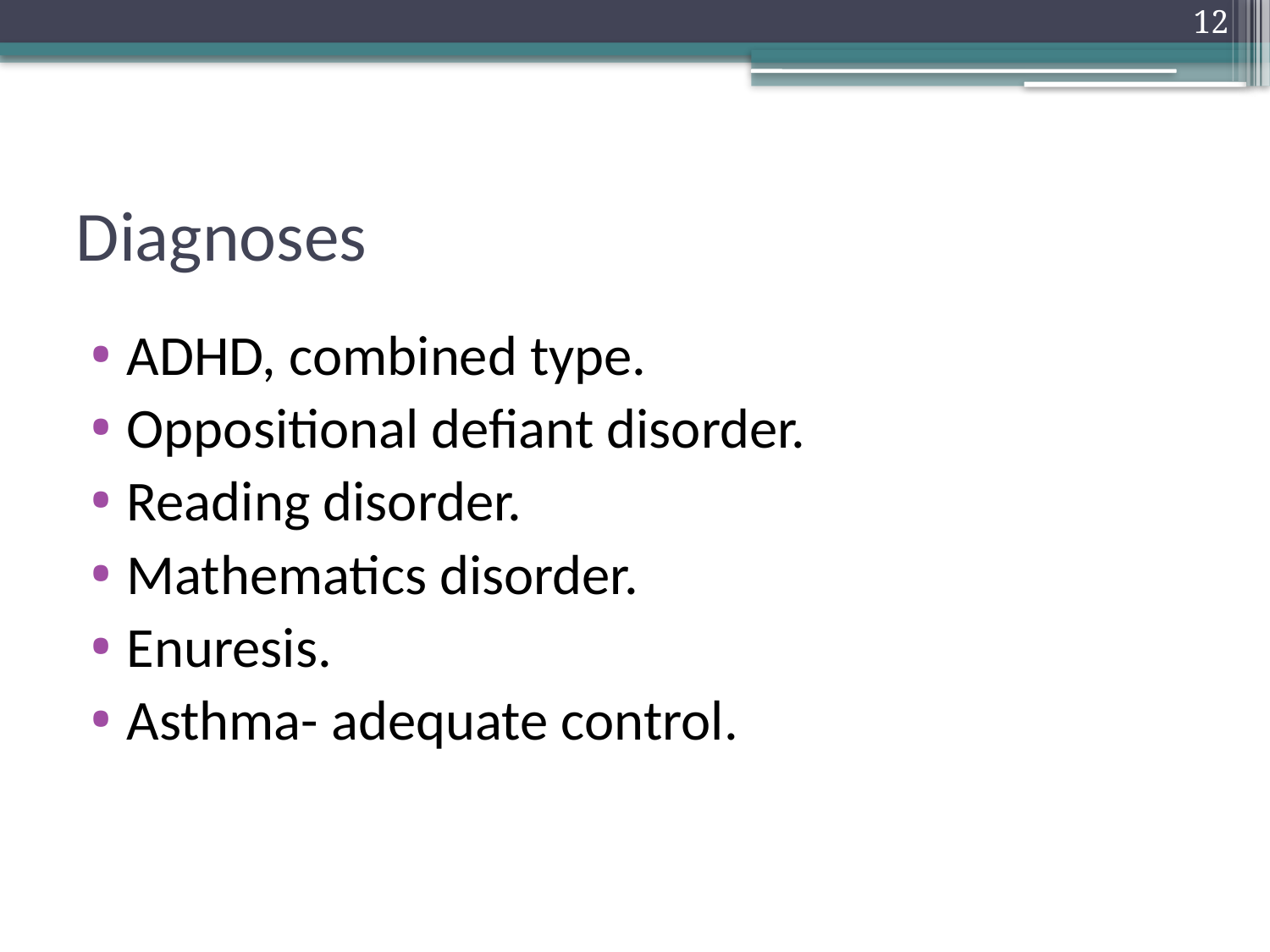

Diagnoses
ADHD, combined type.
Oppositional defiant disorder.
Reading disorder.
Mathematics disorder.
Enuresis.
Asthma- adequate control.
12

## Slide 13
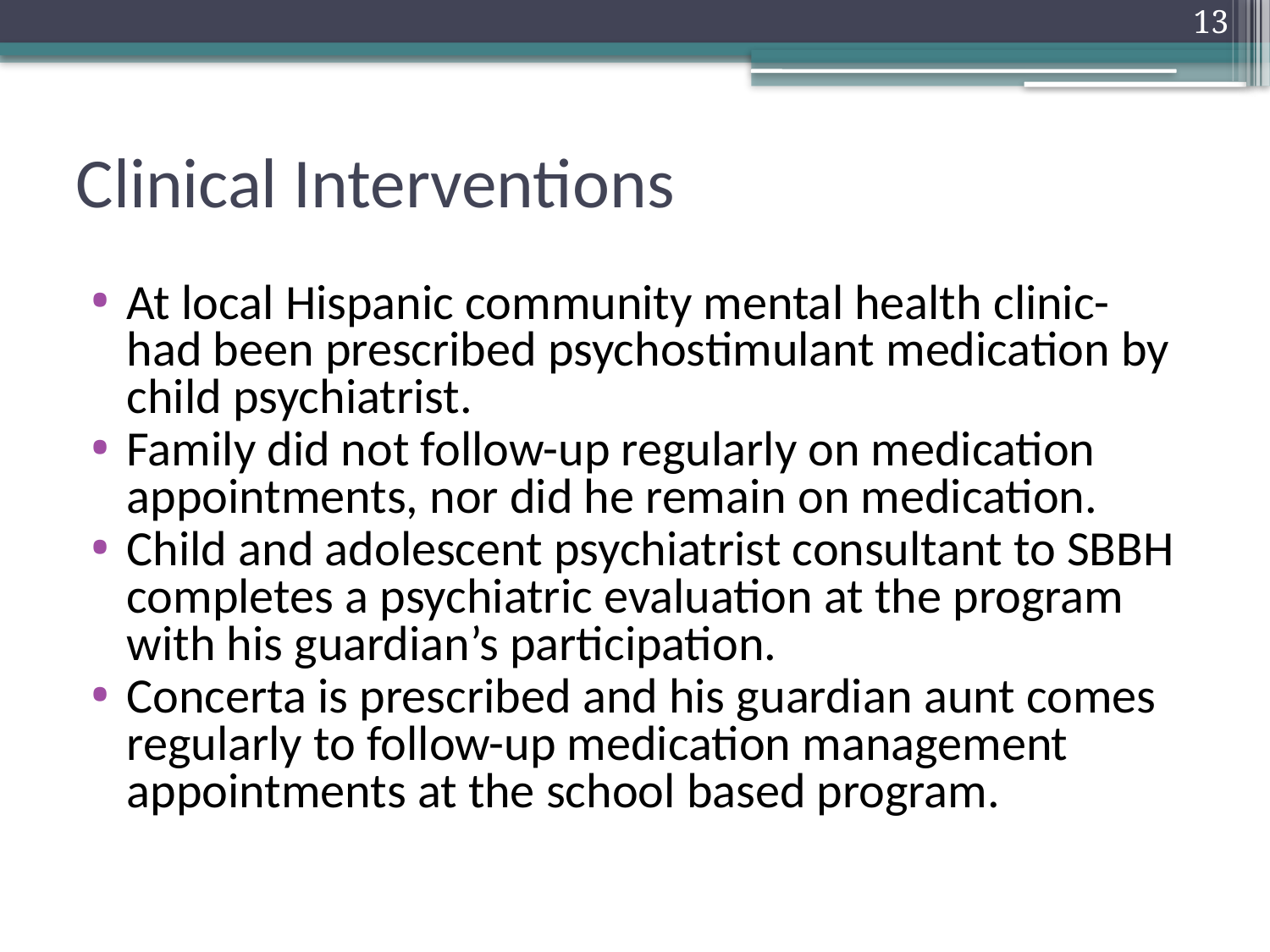

13
Clinical Interventions
At local Hispanic community mental health clinic- had been prescribed psychostimulant medication by child psychiatrist.
Family did not follow-up regularly on medication appointments, nor did he remain on medication.
Child and adolescent psychiatrist consultant to SBBH completes a psychiatric evaluation at the program with his guardian’s participation.
Concerta is prescribed and his guardian aunt comes regularly to follow-up medication management appointments at the school based program.

## Slide 14
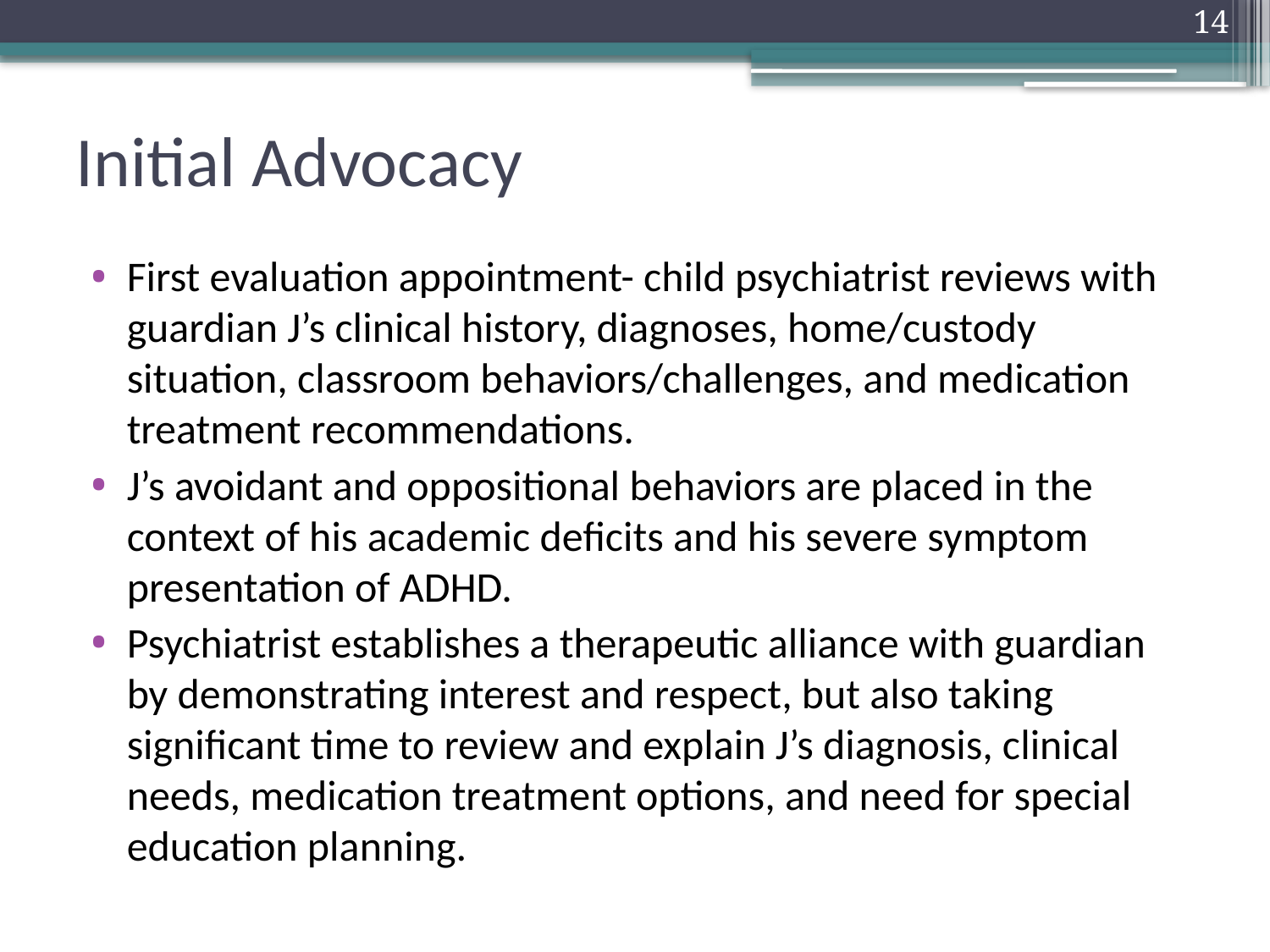

14
Initial Advocacy
First evaluation appointment- child psychiatrist reviews with guardian J’s clinical history, diagnoses, home/custody situation, classroom behaviors/challenges, and medication treatment recommendations.
J’s avoidant and oppositional behaviors are placed in the context of his academic deficits and his severe symptom presentation of ADHD.
Psychiatrist establishes a therapeutic alliance with guardian by demonstrating interest and respect, but also taking significant time to review and explain J’s diagnosis, clinical needs, medication treatment options, and need for special education planning.

## Slide 15
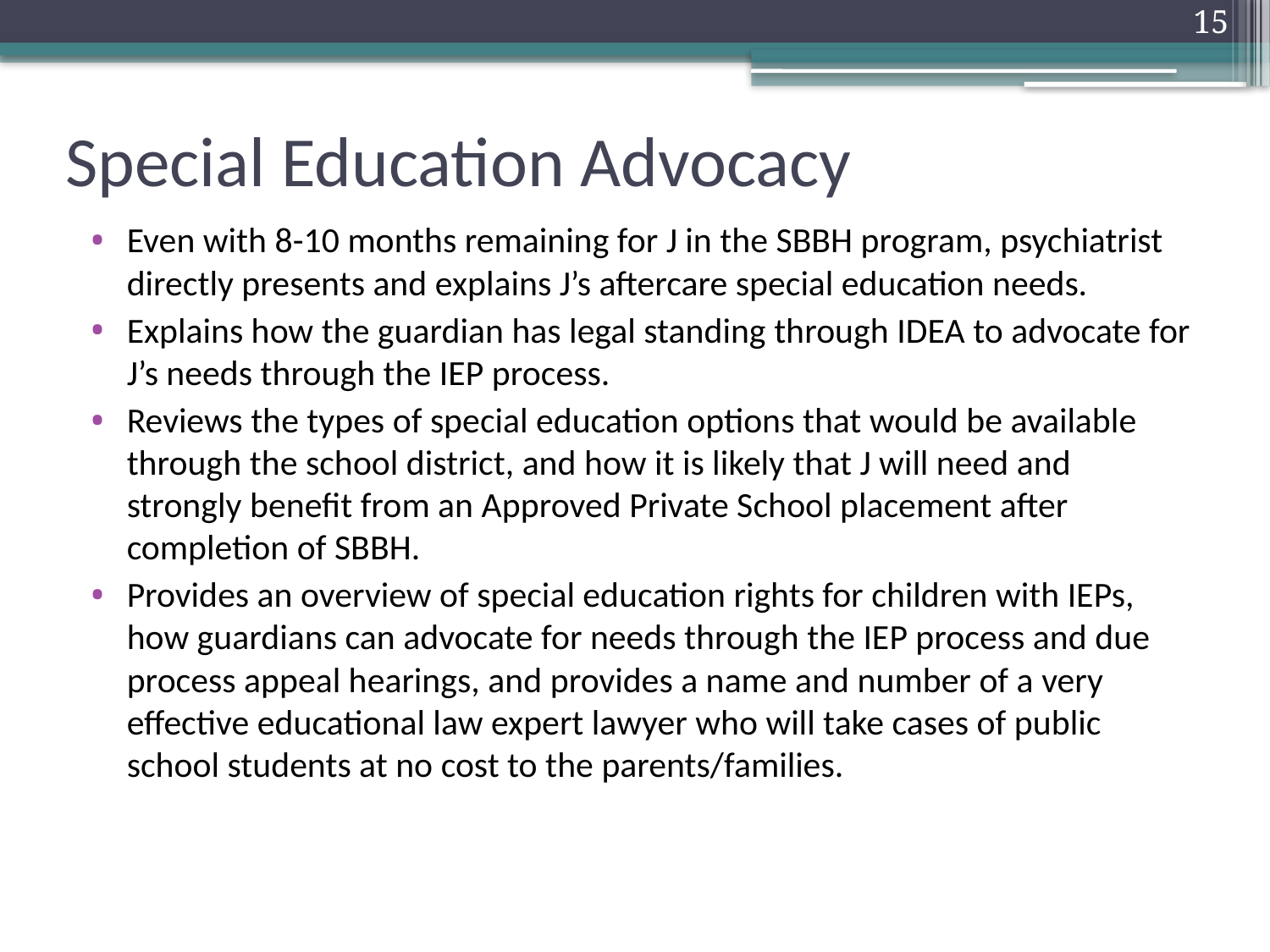

15
Special Education Advocacy
Even with 8-10 months remaining for J in the SBBH program, psychiatrist directly presents and explains J’s aftercare special education needs.
Explains how the guardian has legal standing through IDEA to advocate for J’s needs through the IEP process.
Reviews the types of special education options that would be available through the school district, and how it is likely that J will need and strongly benefit from an Approved Private School placement after completion of SBBH.
Provides an overview of special education rights for children with IEPs, how guardians can advocate for needs through the IEP process and due process appeal hearings, and provides a name and number of a very effective educational law expert lawyer who will take cases of public school students at no cost to the parents/families.

## Slide 16
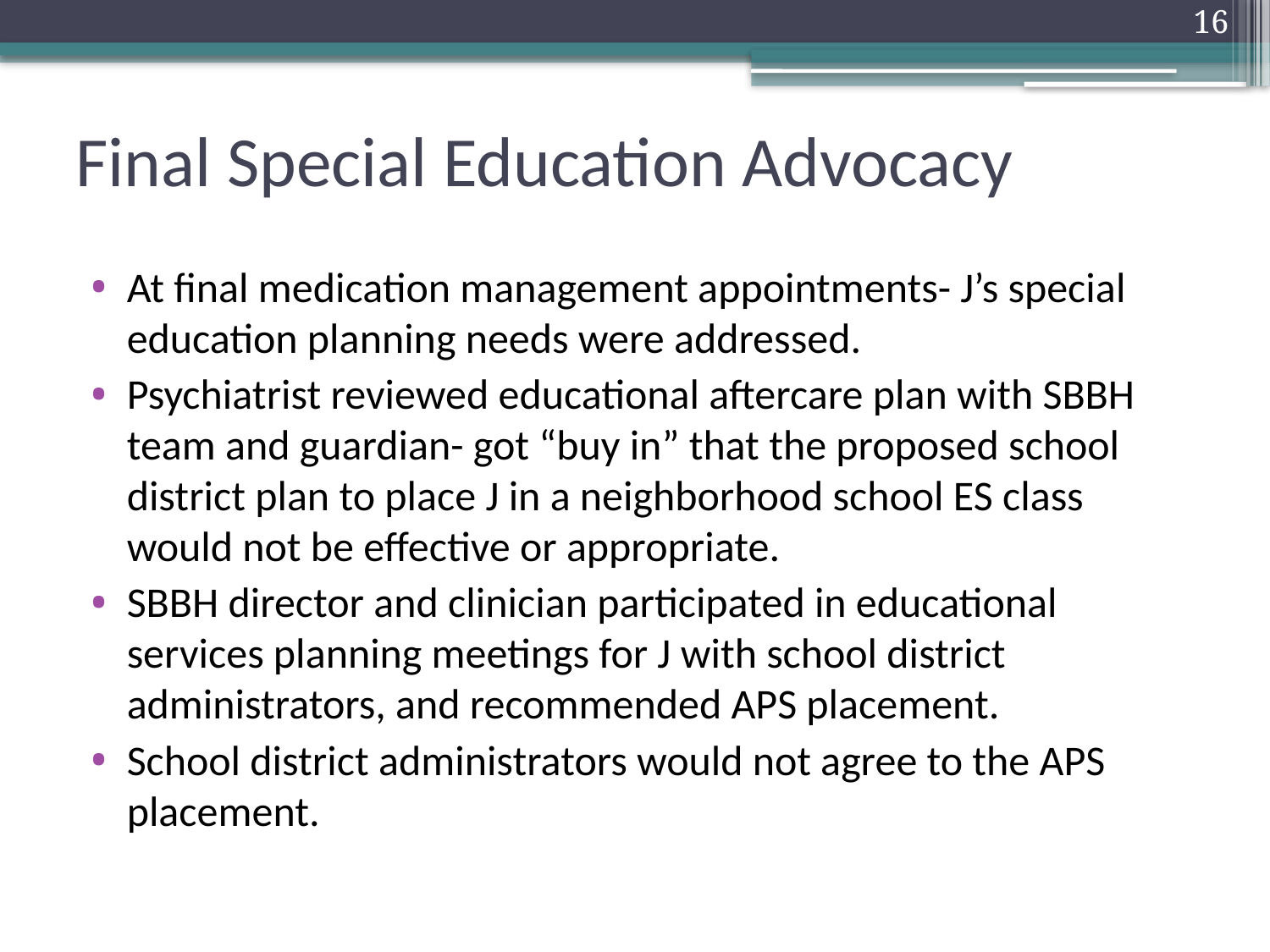

16
Final Special Education Advocacy
At final medication management appointments- J’s special education planning needs were addressed.
Psychiatrist reviewed educational aftercare plan with SBBH team and guardian- got “buy in” that the proposed school district plan to place J in a neighborhood school ES class would not be effective or appropriate.
SBBH director and clinician participated in educational services planning meetings for J with school district administrators, and recommended APS placement.
School district administrators would not agree to the APS placement.

## Slide 17
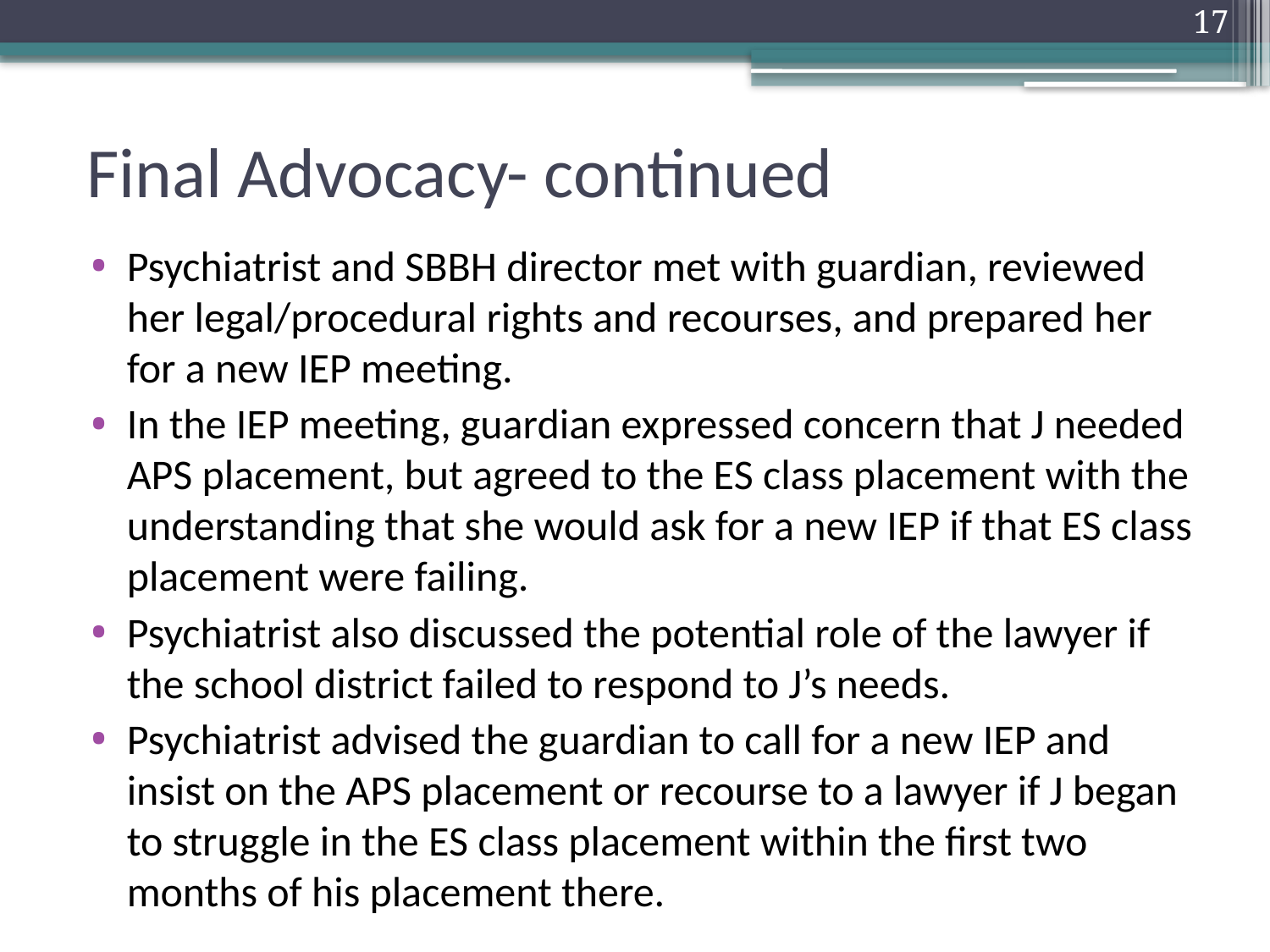

17
Final Advocacy- continued
Psychiatrist and SBBH director met with guardian, reviewed her legal/procedural rights and recourses, and prepared her for a new IEP meeting.
In the IEP meeting, guardian expressed concern that J needed APS placement, but agreed to the ES class placement with the understanding that she would ask for a new IEP if that ES class placement were failing.
Psychiatrist also discussed the potential role of the lawyer if the school district failed to respond to J’s needs.
Psychiatrist advised the guardian to call for a new IEP and insist on the APS placement or recourse to a lawyer if J began to struggle in the ES class placement within the first two months of his placement there.
