# Supplementary material for: The JeffSTARS Advocacy and Community Partnership Elective: A Closer Look at Child Health Advocacy in Action
Source: MedEdPORTAL. 2016 Dec 31;12:10526. doi: 10.15766/mep_2374-8265.10526 (PMC6365684; doi:10.15766/mep_2374-8265.10526)
Supplement: Supplementary file 1 — A. CM1. Course Implementation at New Institution Checklist.docx B. CM2. Elective Checklist.docx C. CM3. Sample Schedule.docx D. CM4. Seminar Topic List With Learning Objectives.docx E. CM5. Syllabus Bibliography.docx F. CM6. List of Community Partners.docx G. CM7. Orientation for New Community Partner.docx H. CM8. Selected Past Projects.docx I. CM9. Sample Fact Sheets for Legislative Visits.docx J. Seminar Materials folder K. ET1. Advocacy Elective Assessment 1.pdf L. ET2. Advocacy Elective Assessment 2.pdf M. ET3. Trainee Evaluation by Community or Faculty Mentor.docx N. ET4. Trainee Evaluation of Seminar.docx O. ET5. Trainee Evaluation of Community Partner.docx P. ET6. Final Report Template.docx Q. Selected Trainee Abstracts and Presented Results folder [file mep-12-10526-s001.zip › J._Seminar_Materials_folder/12._Advocacy_in_the_Office_Setting.pptx]

## Slide 1
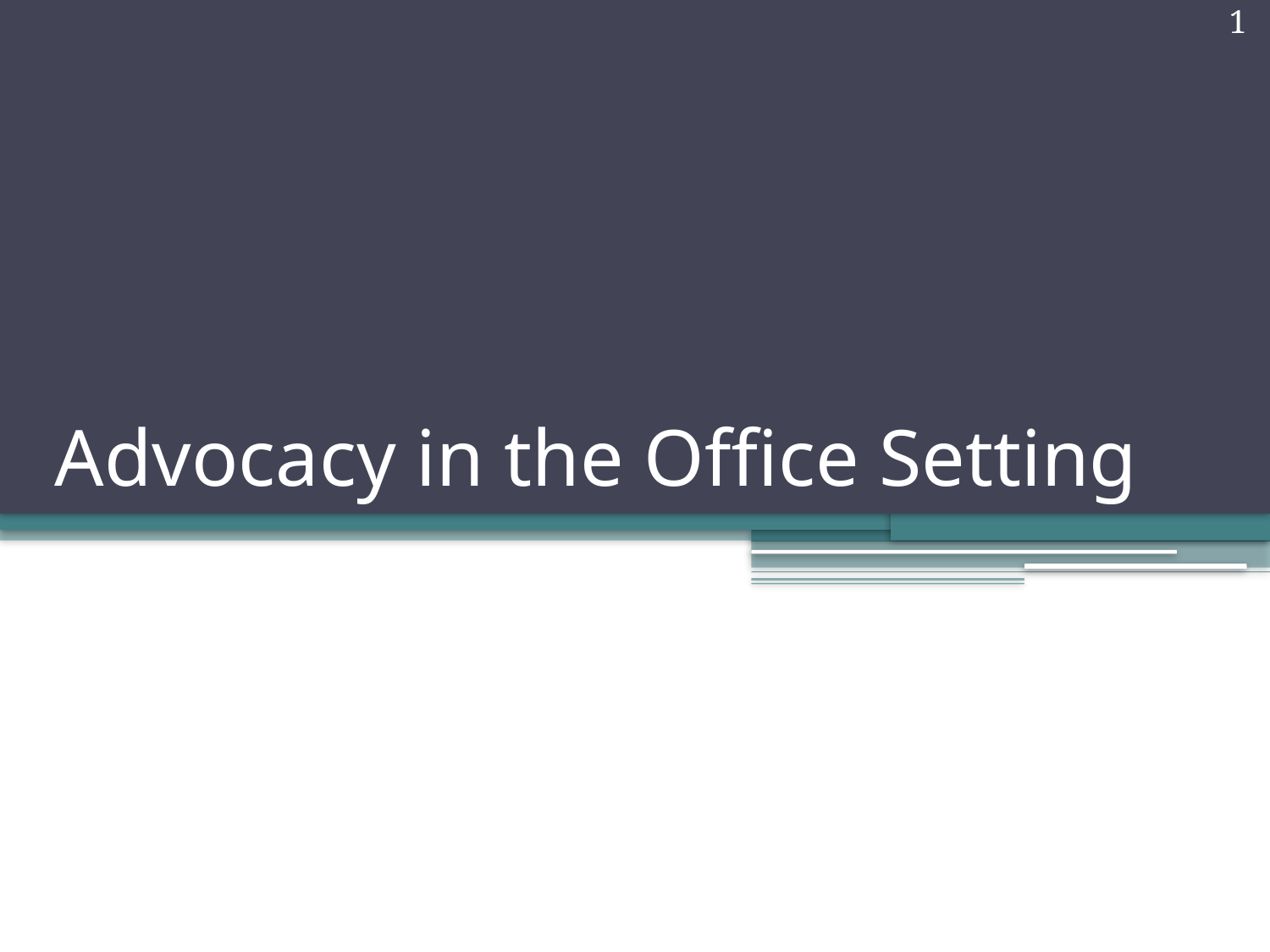

1
# Advocacy in the Office Setting

## Slide 2
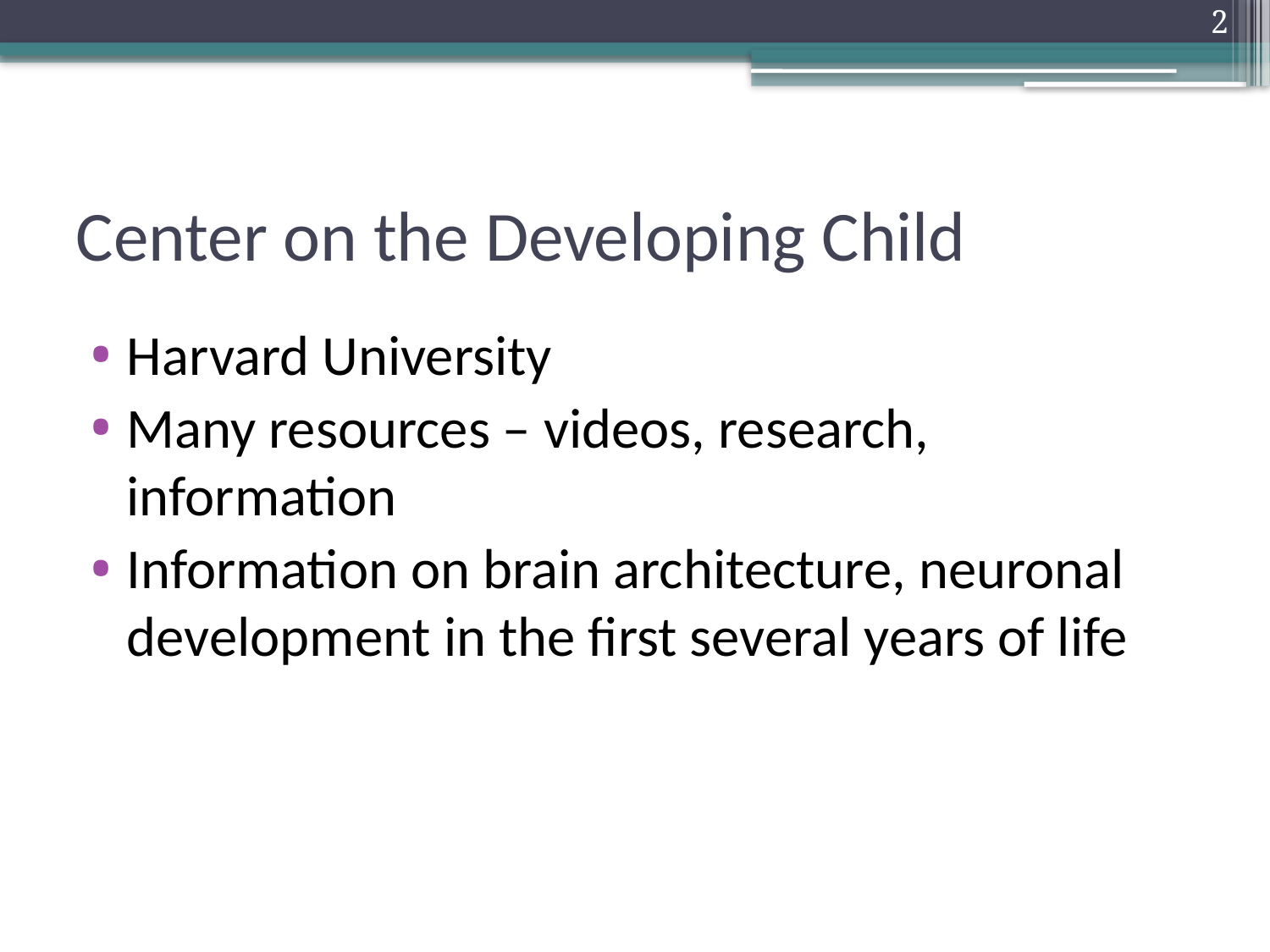

2
# Center on the Developing Child
Harvard University
Many resources – videos, research, information
Information on brain architecture, neuronal development in the first several years of life

## Slide 3
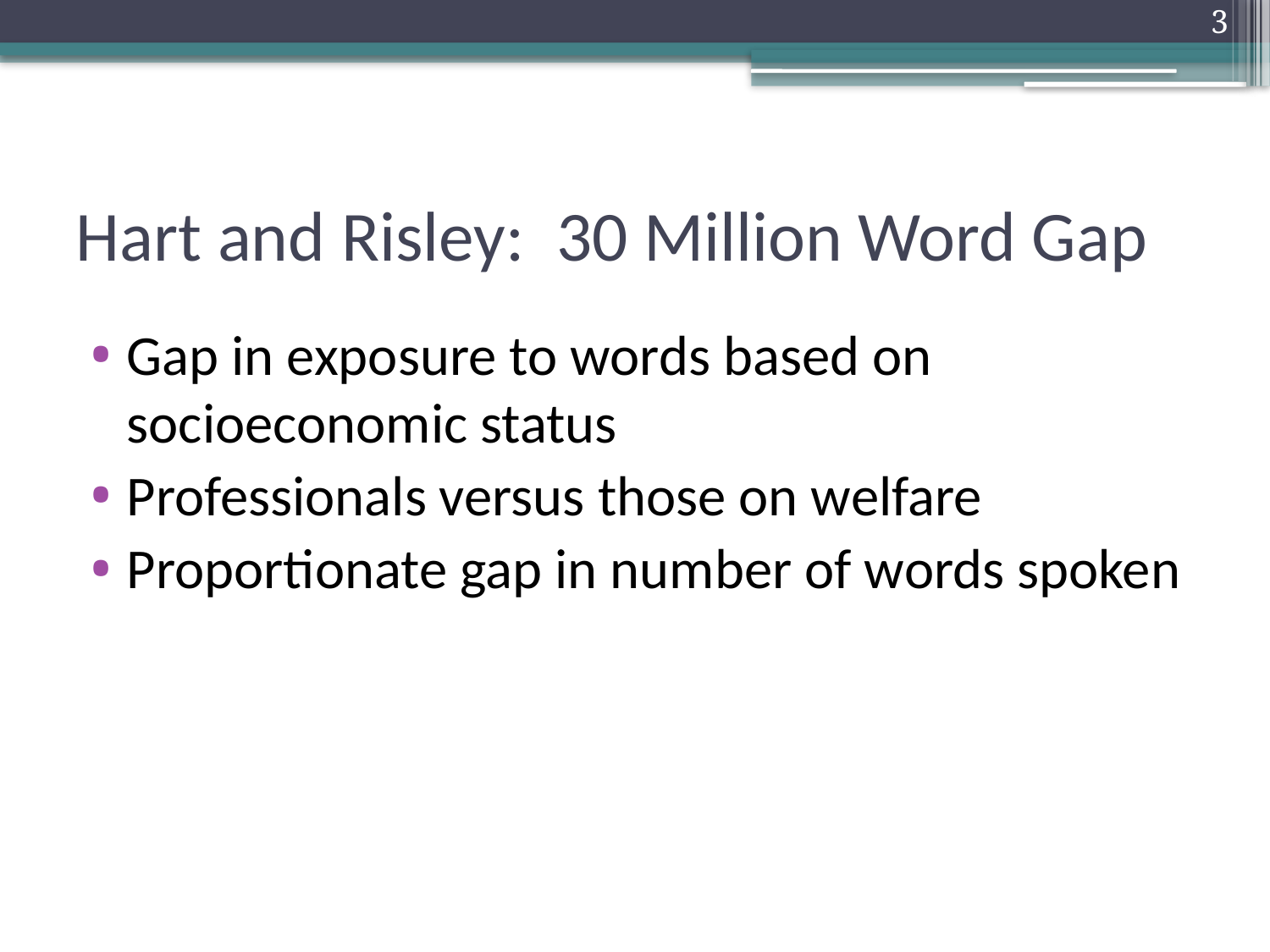

3
# Hart and Risley: 30 Million Word Gap
Gap in exposure to words based on socioeconomic status
Professionals versus those on welfare
Proportionate gap in number of words spoken

## Slide 4
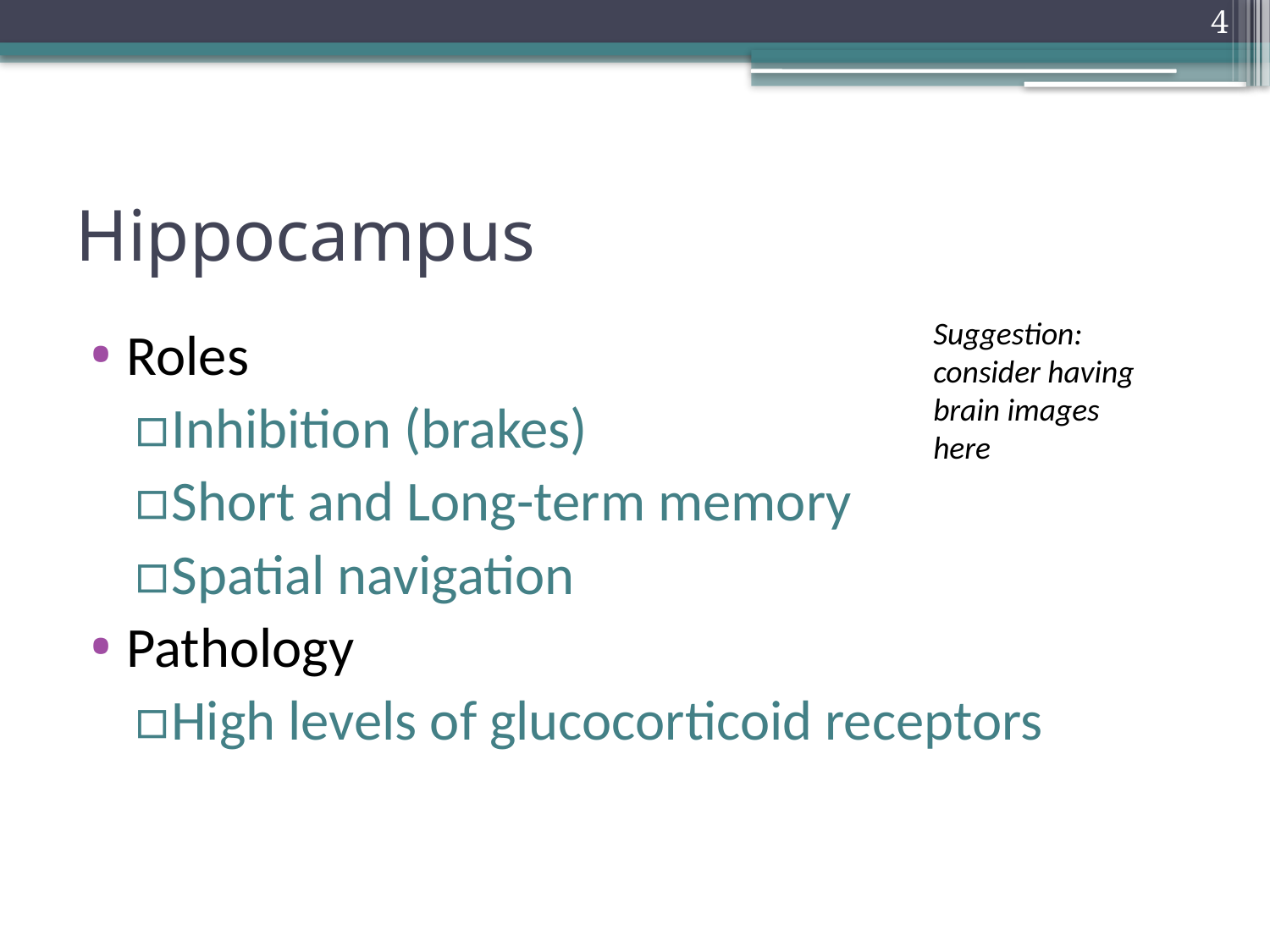

4
# Hippocampus
Suggestion: consider having brain images here
Roles
Inhibition (brakes)
Short and Long-term memory
Spatial navigation
Pathology
High levels of glucocorticoid receptors

## Slide 5
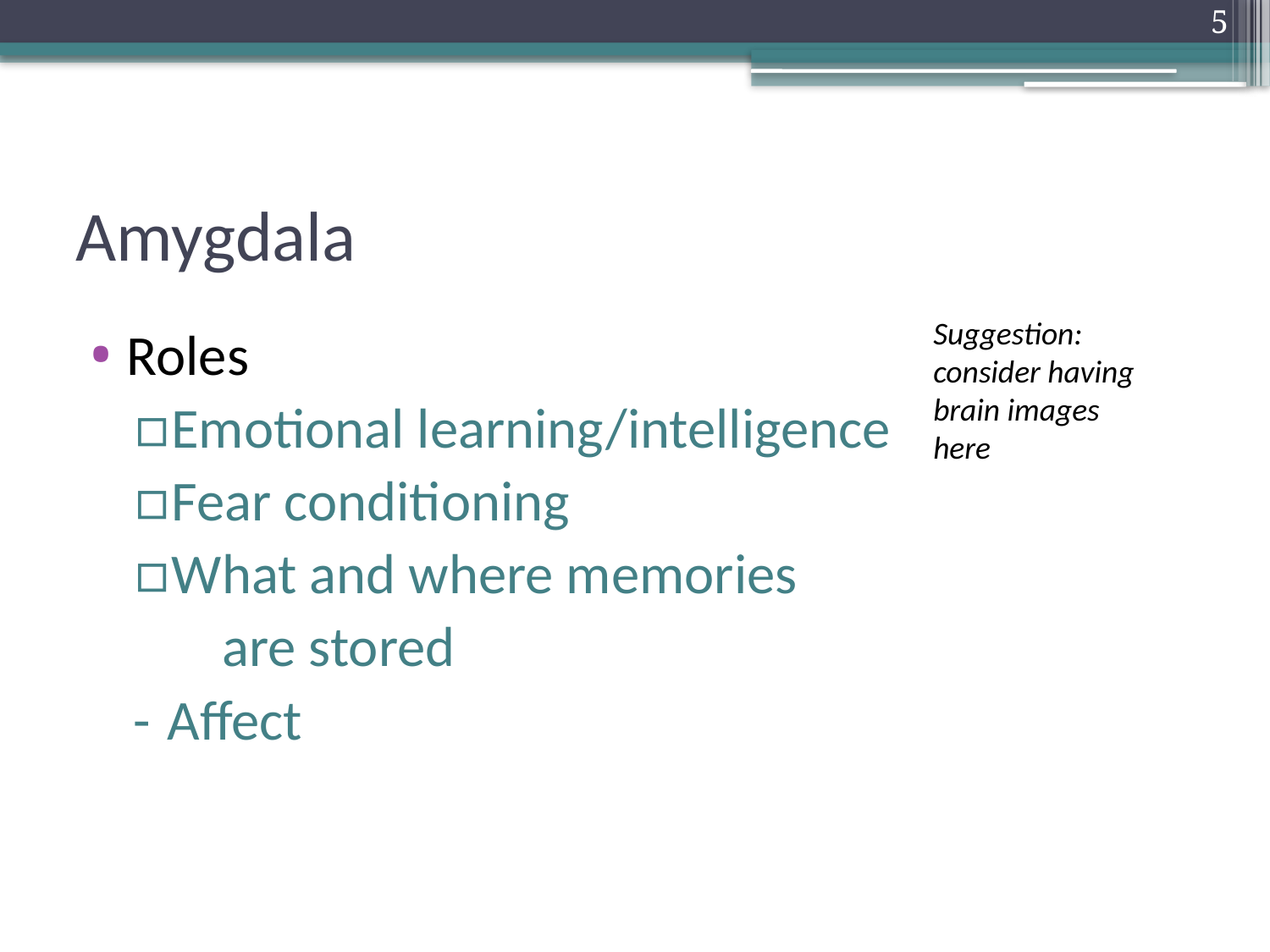

5
# Amygdala
Suggestion: consider having brain images here
Roles
Emotional learning/intelligence
Fear conditioning
What and where memories
 are stored
-	Affect

## Slide 6
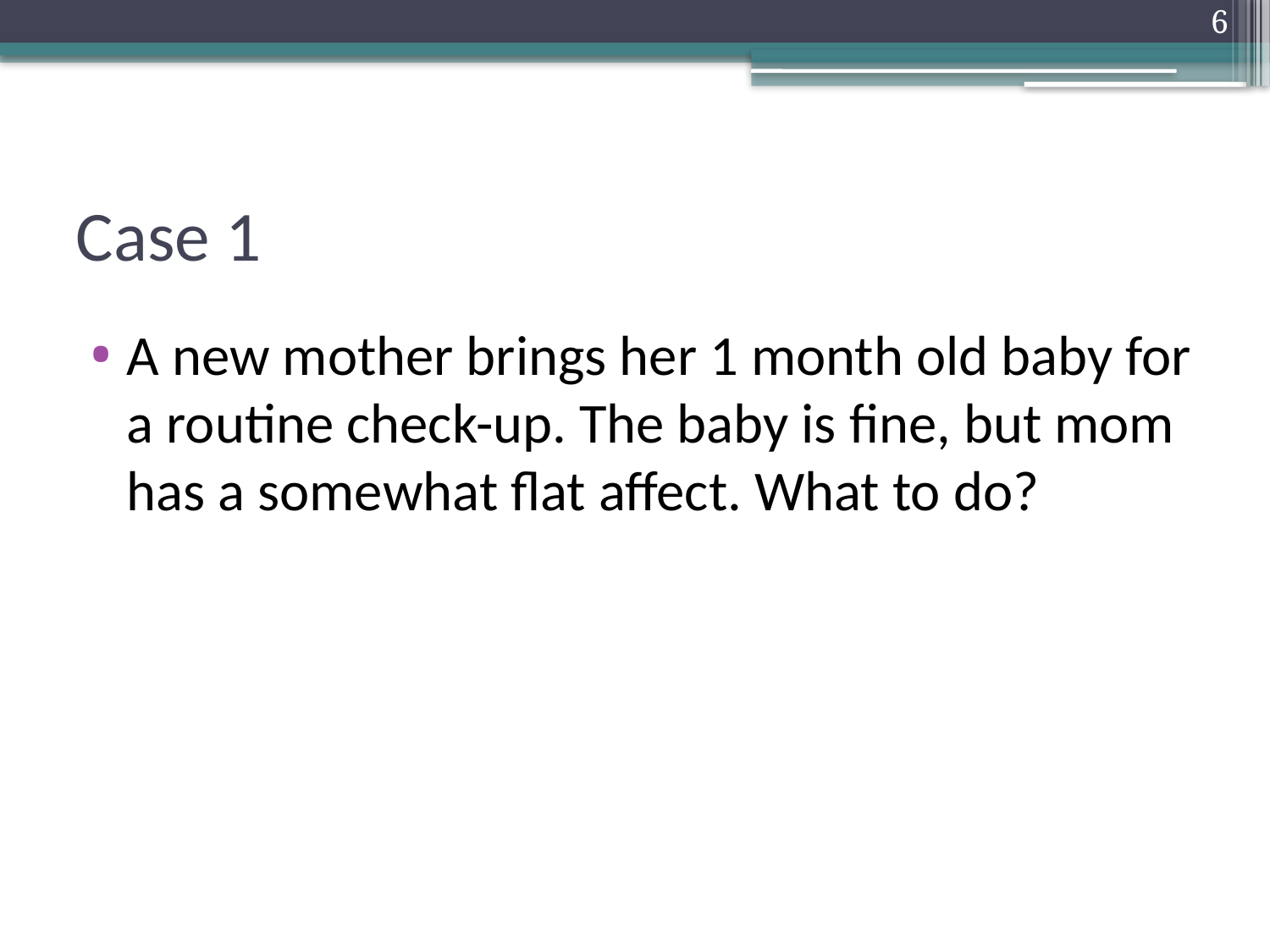

6
# Case 1
A new mother brings her 1 month old baby for a routine check-up. The baby is fine, but mom has a somewhat flat affect. What to do?

## Slide 7
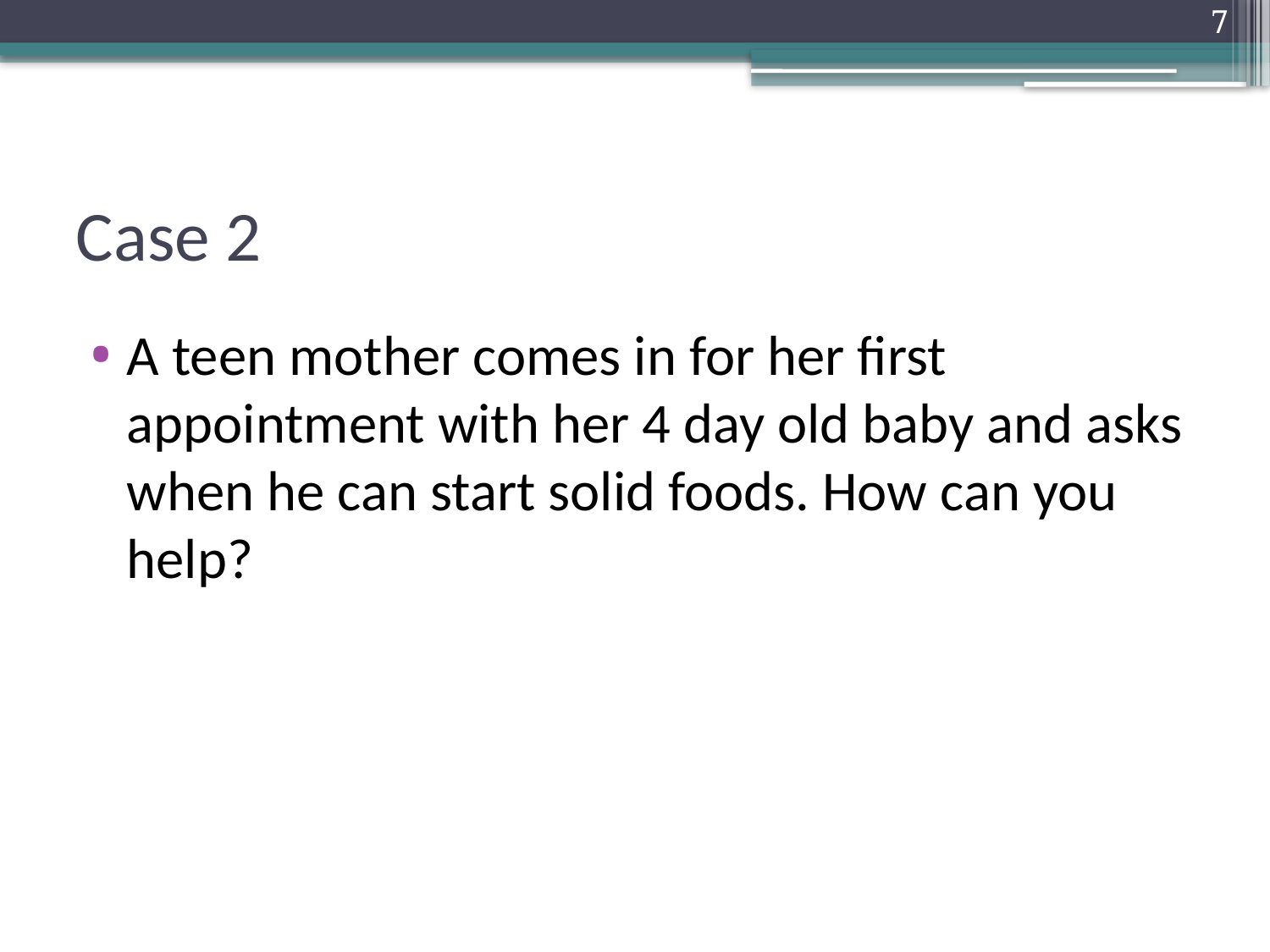

7
# Case 2
A teen mother comes in for her first appointment with her 4 day old baby and asks when he can start solid foods. How can you help?

## Slide 8
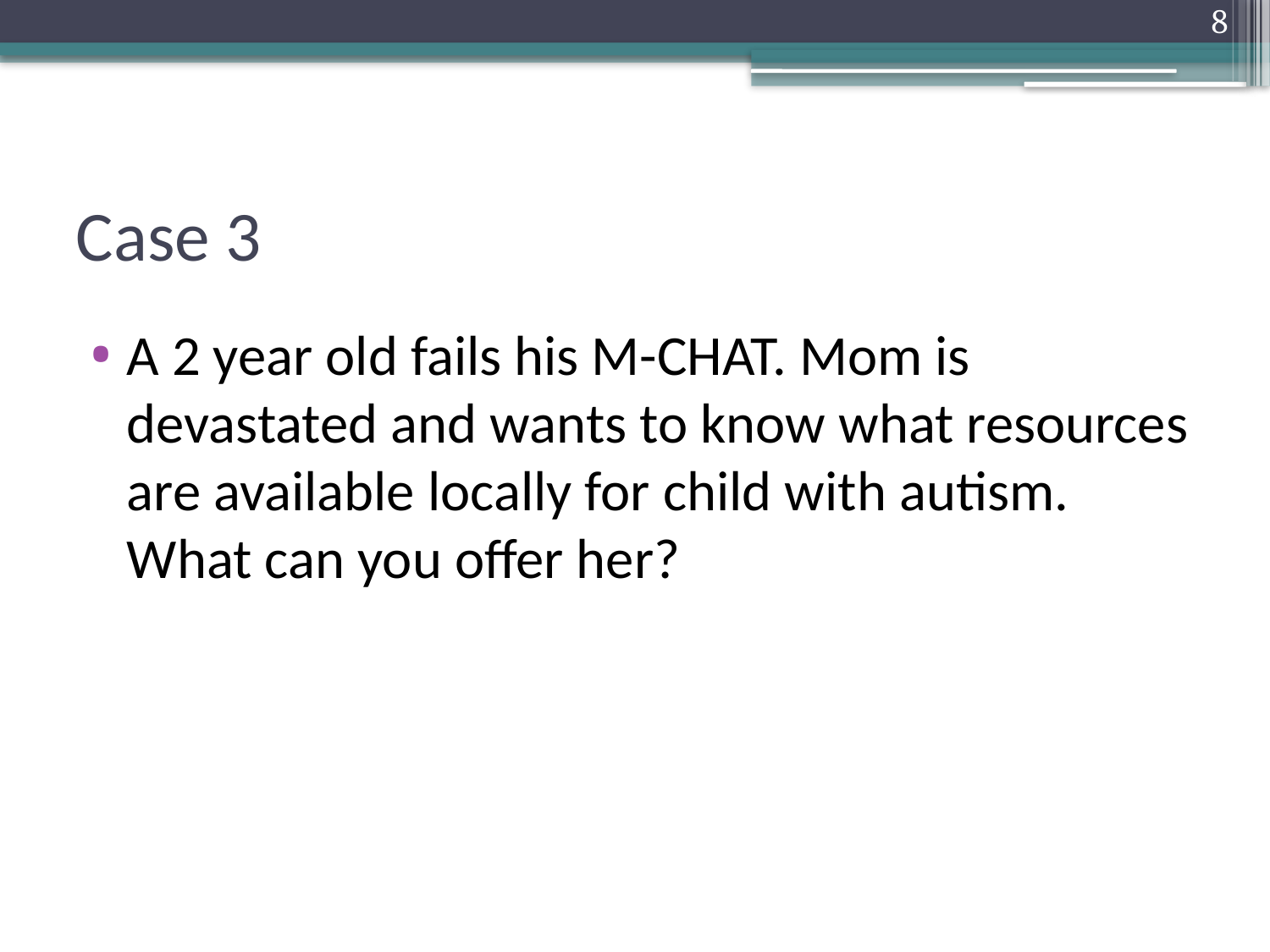

8
# Case 3
A 2 year old fails his M-CHAT. Mom is devastated and wants to know what resources are available locally for child with autism. What can you offer her?

## Slide 9
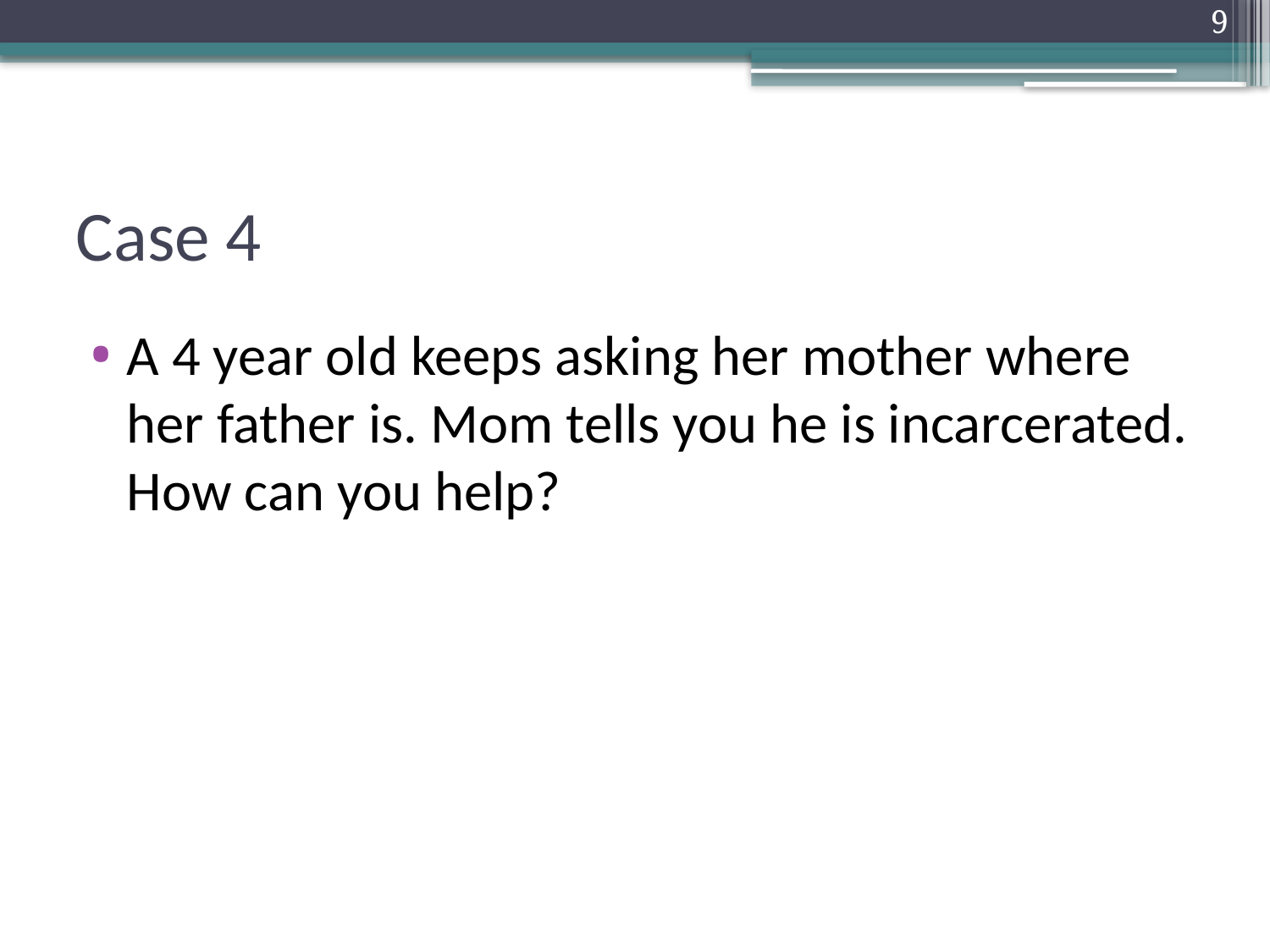

9
# Case 4
A 4 year old keeps asking her mother where her father is. Mom tells you he is incarcerated. How can you help?

## Slide 10
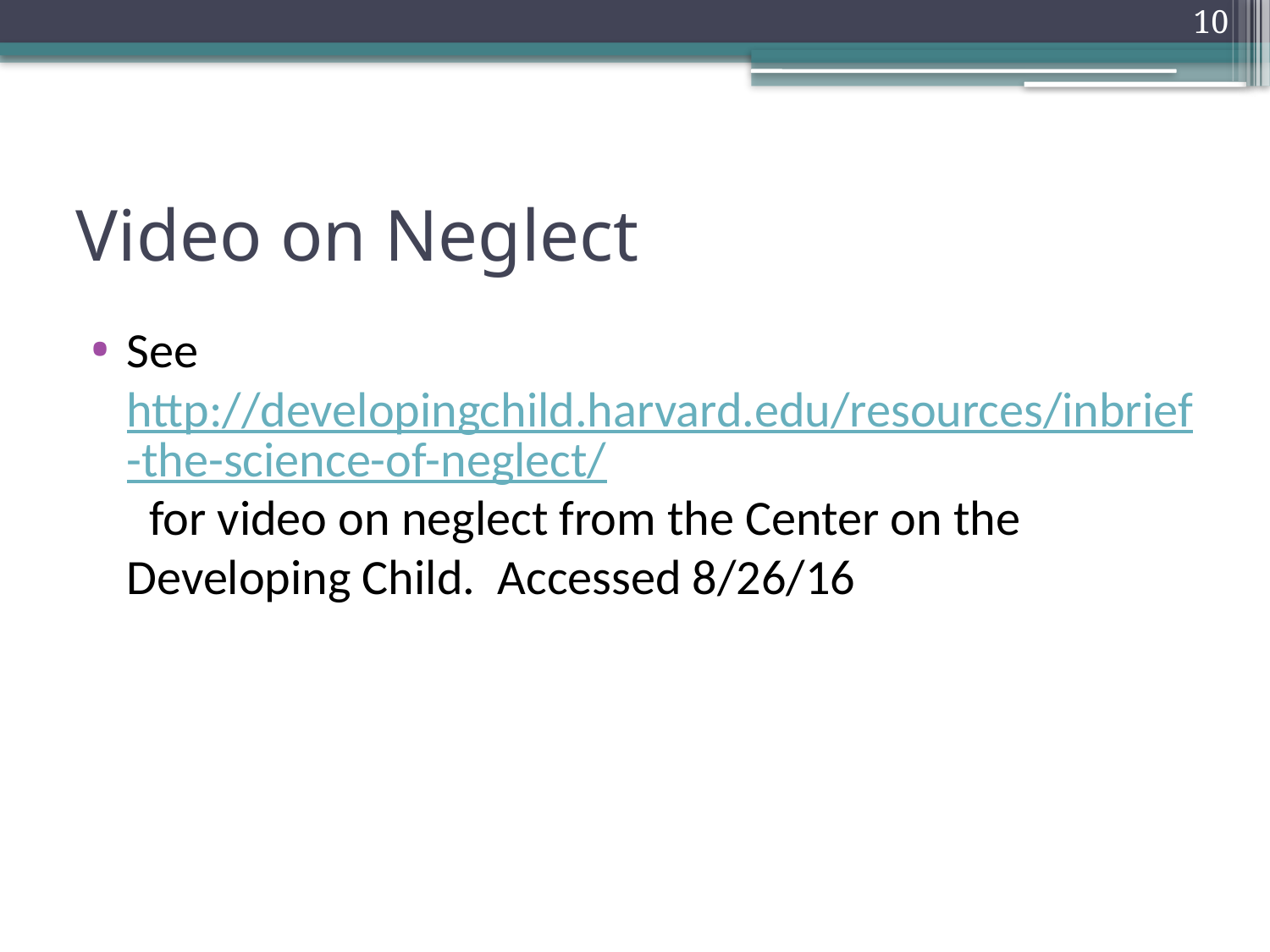

10
# Video on Neglect
See http://developingchild.harvard.edu/resources/inbrief-the-science-of-neglect/ for video on neglect from the Center on the Developing Child. Accessed 8/26/16

## Slide 11
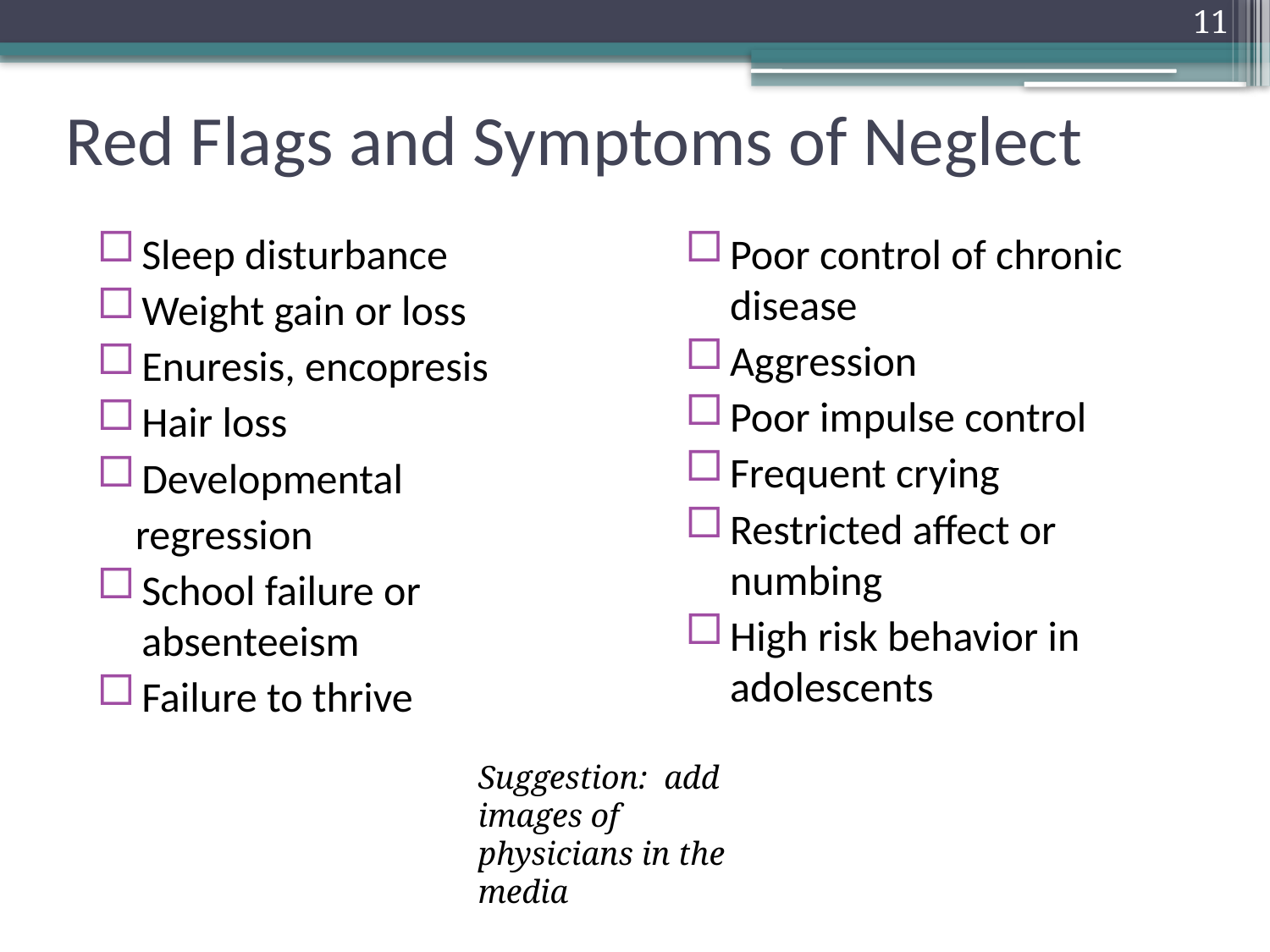

11
# Red Flags and Symptoms of Neglect
Sleep disturbance
Weight gain or loss
Enuresis, encopresis
Hair loss
Developmental
 regression
School failure or absenteeism
Failure to thrive
Poor control of chronic disease
Aggression
Poor impulse control
Frequent crying
Restricted affect or numbing
High risk behavior in adolescents
Suggestion: add images of physicians in the media

## Slide 12
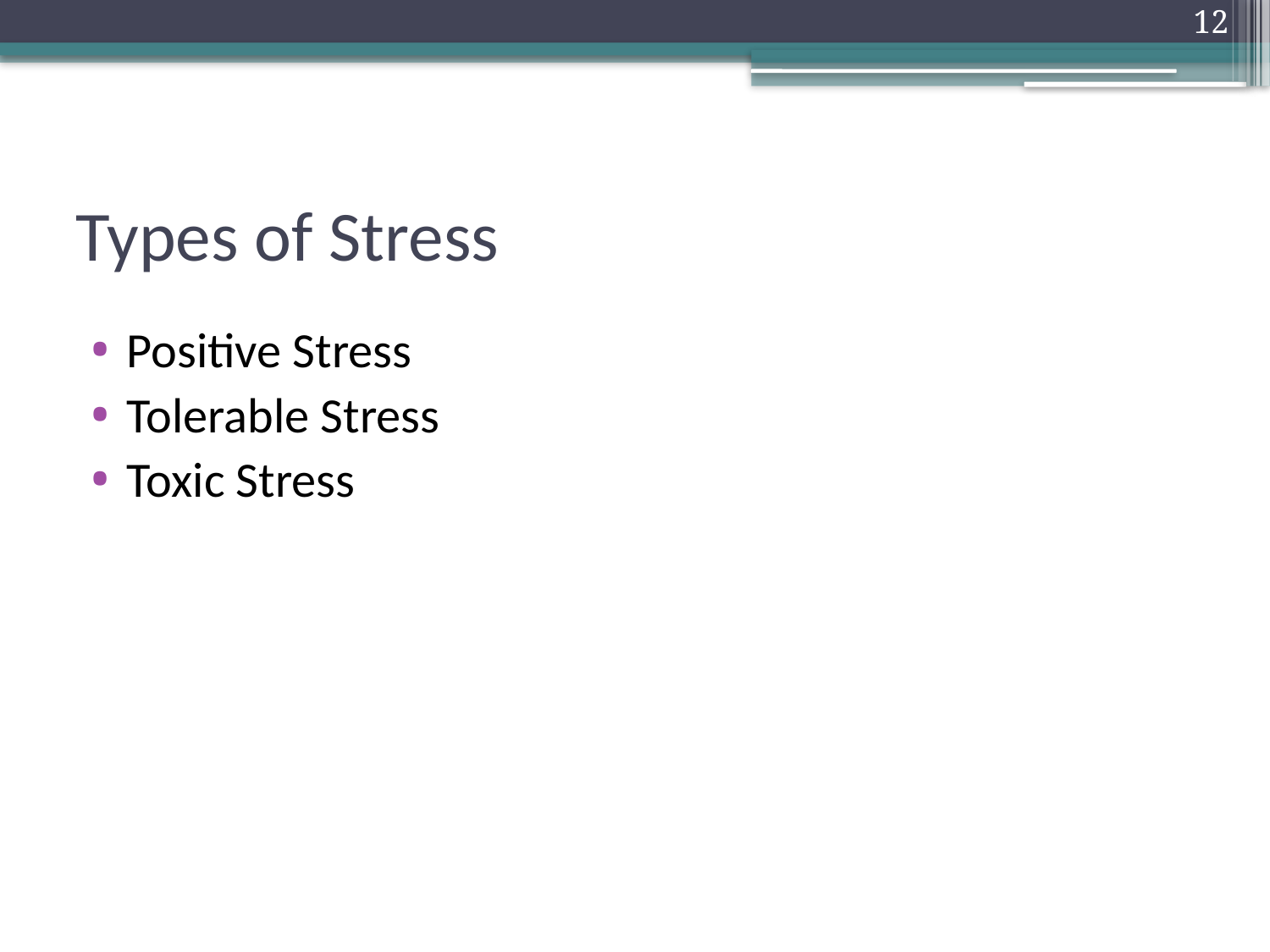

12
# Types of Stress
Positive Stress
Tolerable Stress
Toxic Stress

## Slide 13
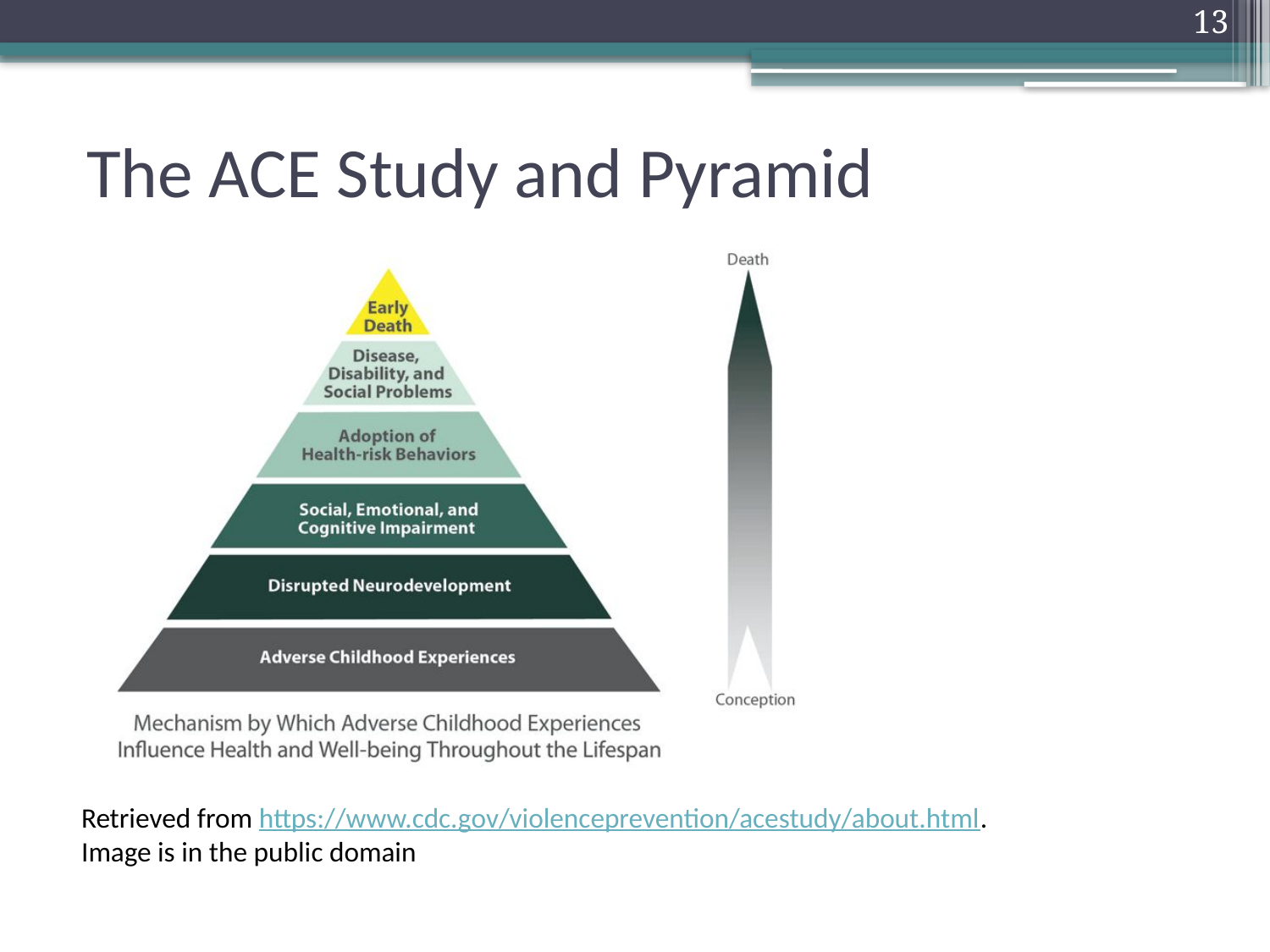

13
# The ACE Study and Pyramid
Retrieved from https://www.cdc.gov/violenceprevention/acestudy/about.html.
Image is in the public domain

## Slide 14
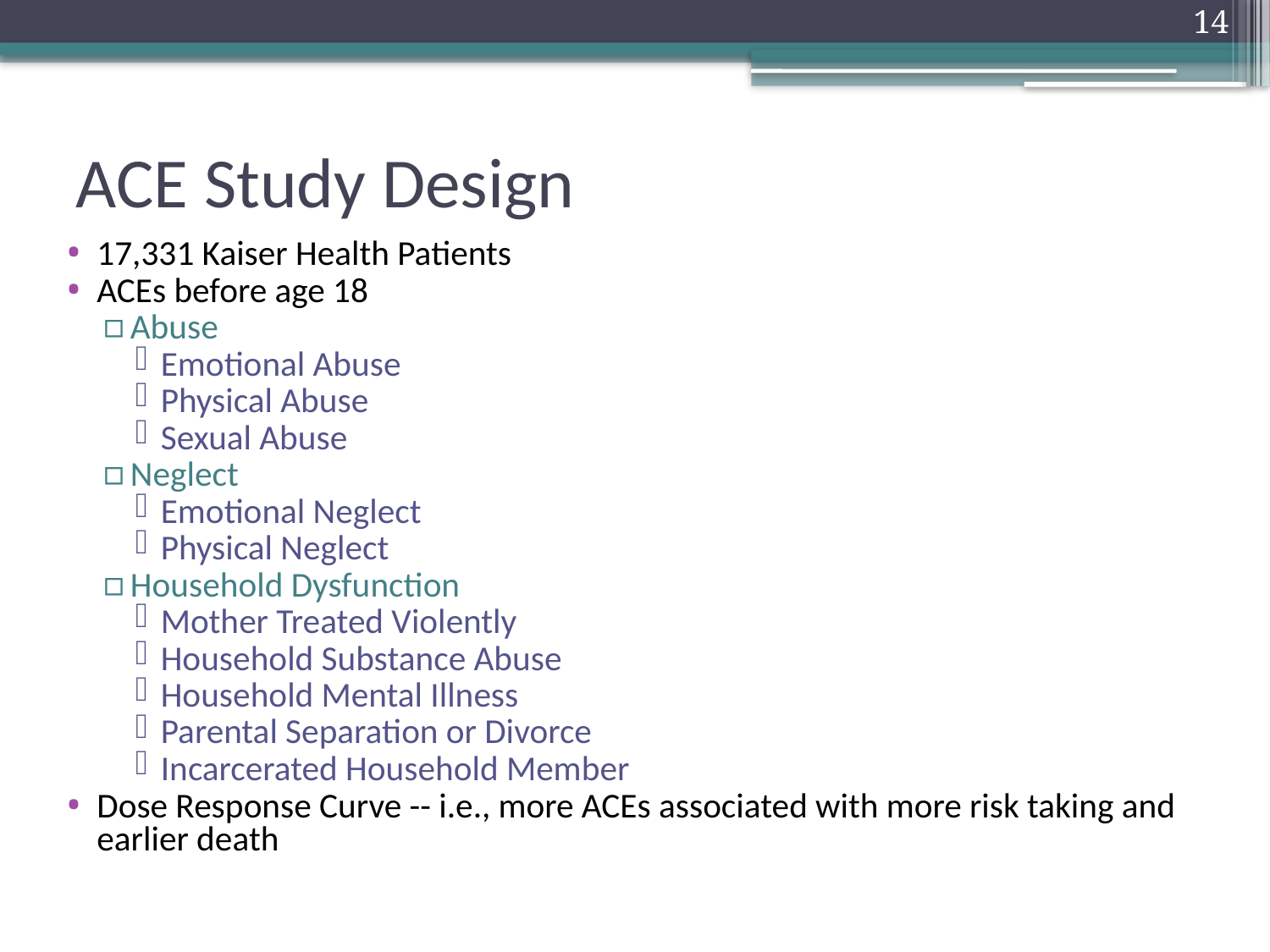

14
# ACE Study Design
17,331 Kaiser Health Patients
ACEs before age 18
Abuse
Emotional Abuse
Physical Abuse
Sexual Abuse
Neglect
Emotional Neglect
Physical Neglect
Household Dysfunction
Mother Treated Violently
Household Substance Abuse
Household Mental Illness
Parental Separation or Divorce
Incarcerated Household Member
Dose Response Curve -- i.e., more ACEs associated with more risk taking and earlier death

## Slide 15
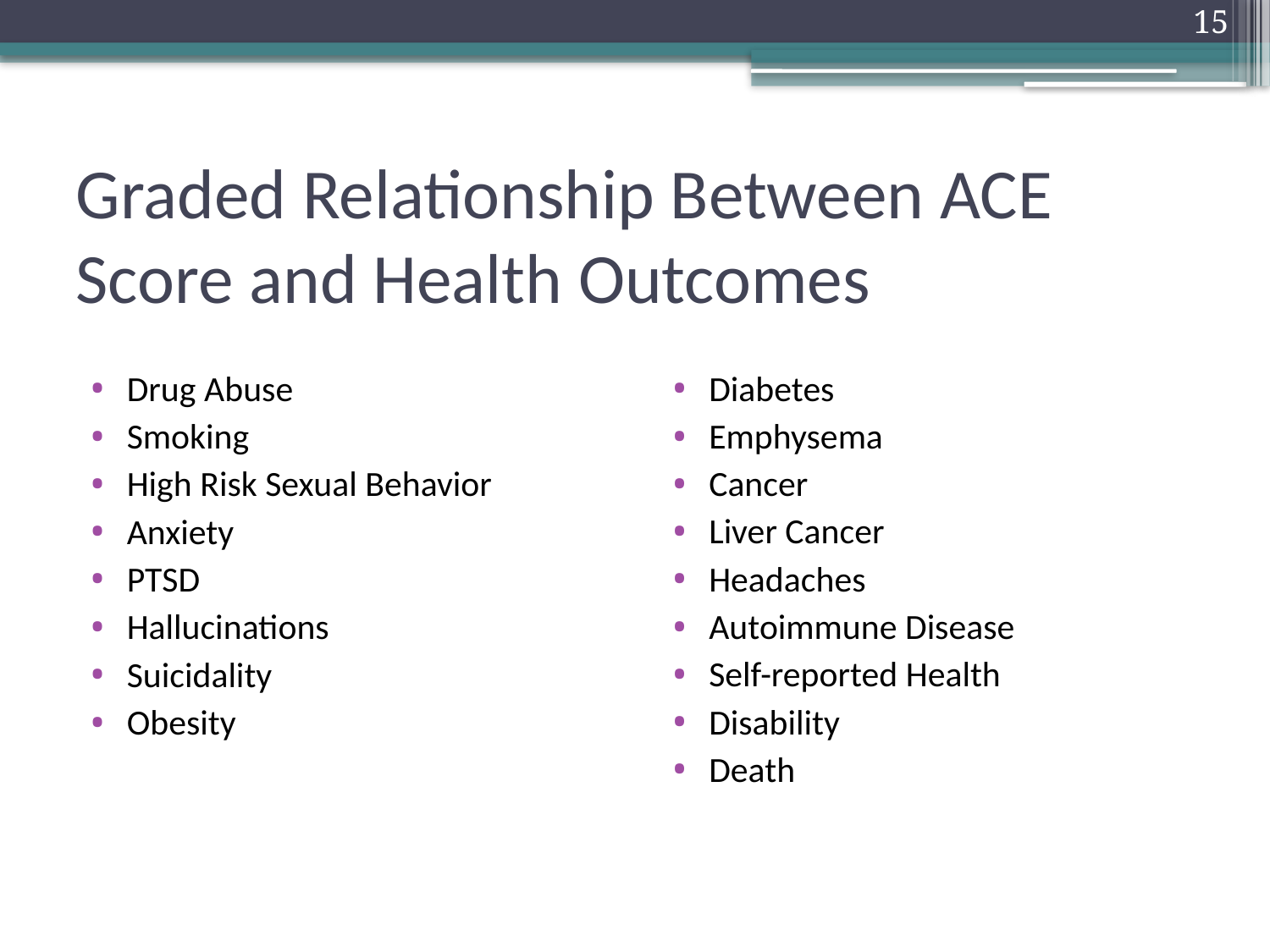

15
# Graded Relationship Between ACE Score and Health Outcomes
Drug Abuse
Smoking
High Risk Sexual Behavior
Anxiety
PTSD
Hallucinations
Suicidality
Obesity
Diabetes
Emphysema
Cancer
Liver Cancer
Headaches
Autoimmune Disease
Self-reported Health
Disability
Death

## Slide 16
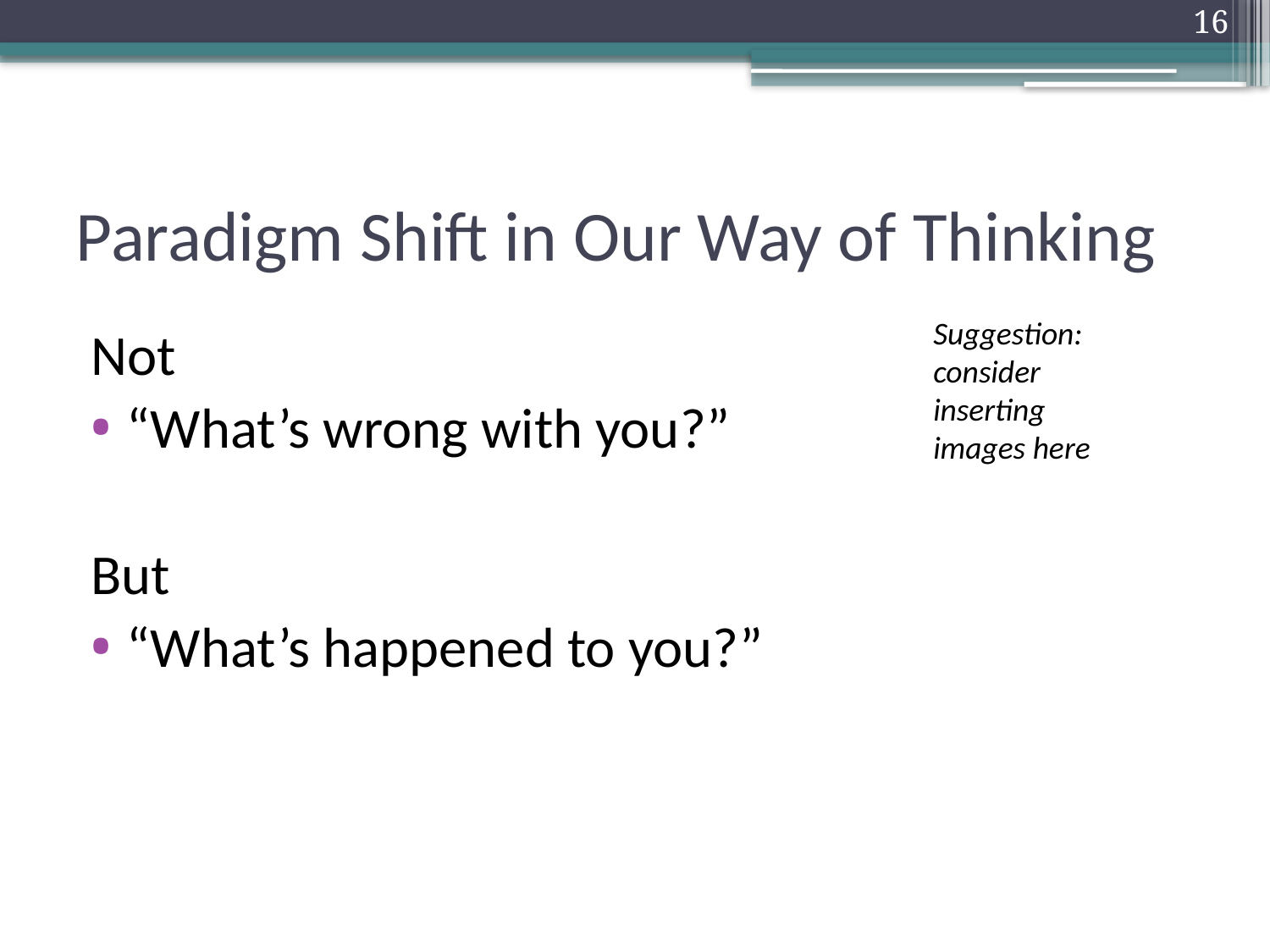

16
# Paradigm Shift in Our Way of Thinking
Suggestion: consider inserting images here
Not
“What’s wrong with you?”
But
“What’s happened to you?”

## Slide 17
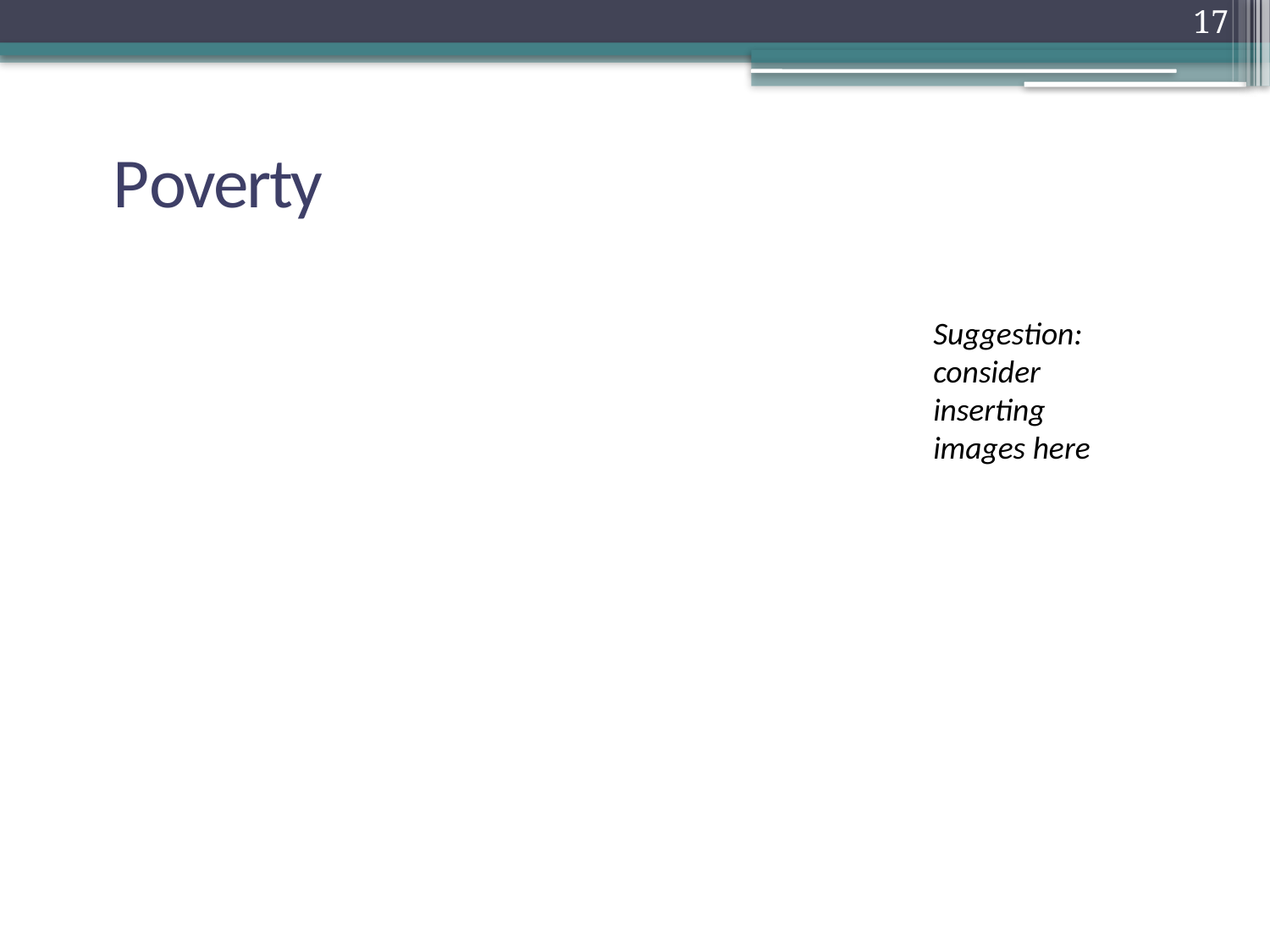

17
 Poverty
Suggestion: consider inserting images here

## Slide 18
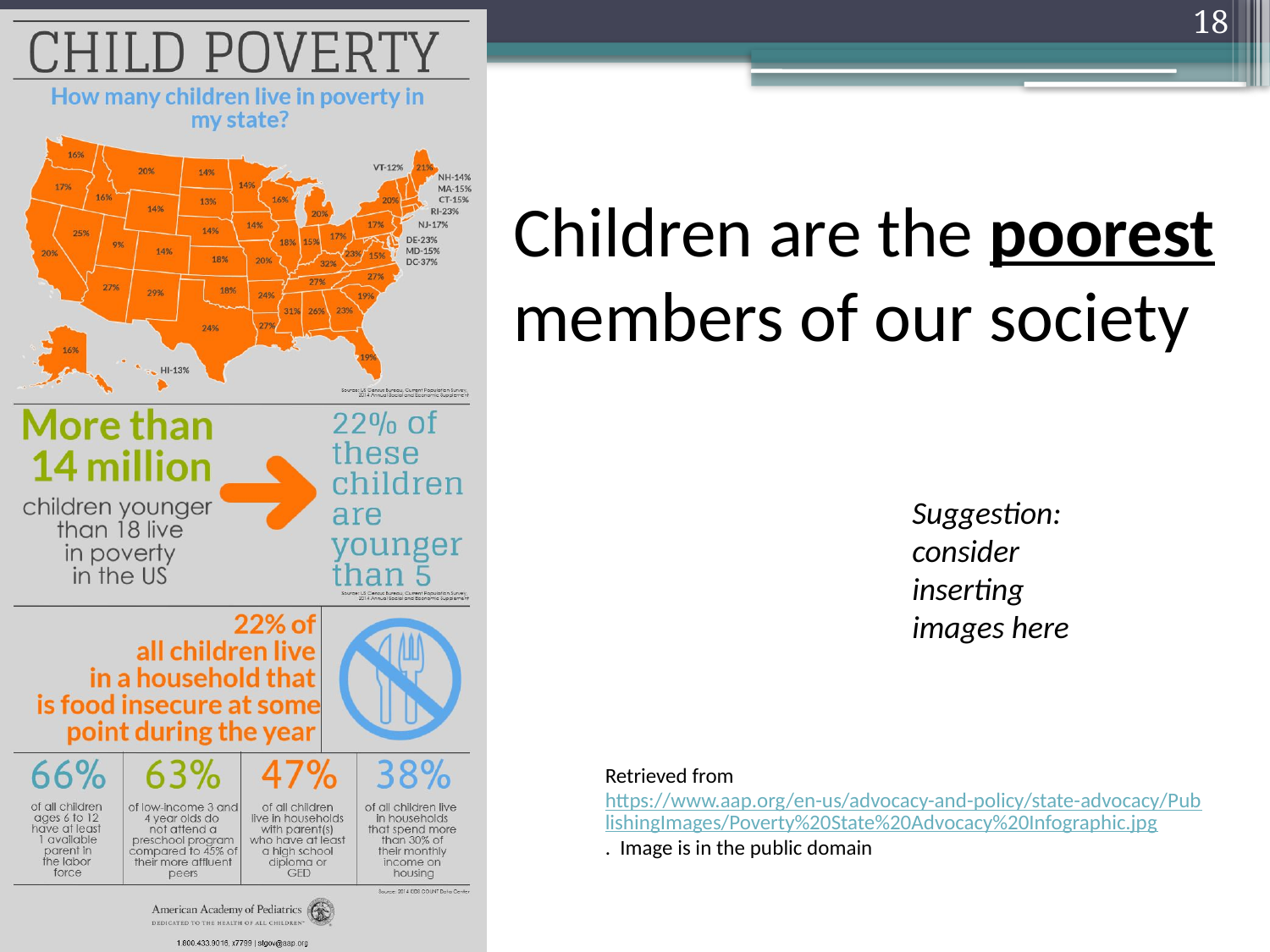

18
Children are the poorest
members of our society
Suggestion: consider inserting images here
Retrieved from https://www.aap.org/en-us/advocacy-and-policy/state-advocacy/PublishingImages/Poverty%20State%20Advocacy%20Infographic.jpg. Image is in the public domain

## Slide 19
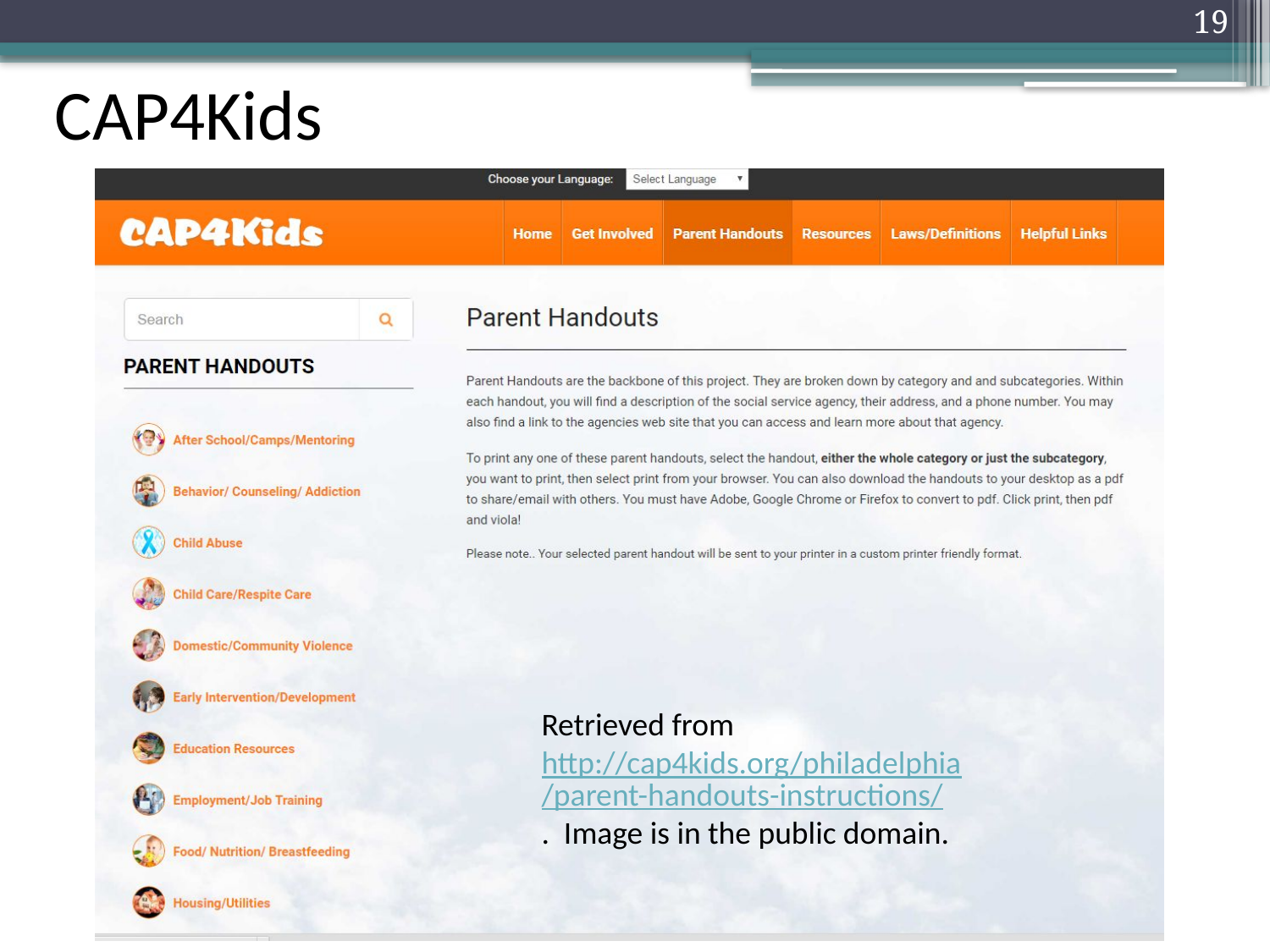

19
CAP4Kids
Retrieved from http://cap4kids.org/philadelphia/parent-handouts-instructions/. Image is in the public domain.

## Slide 20
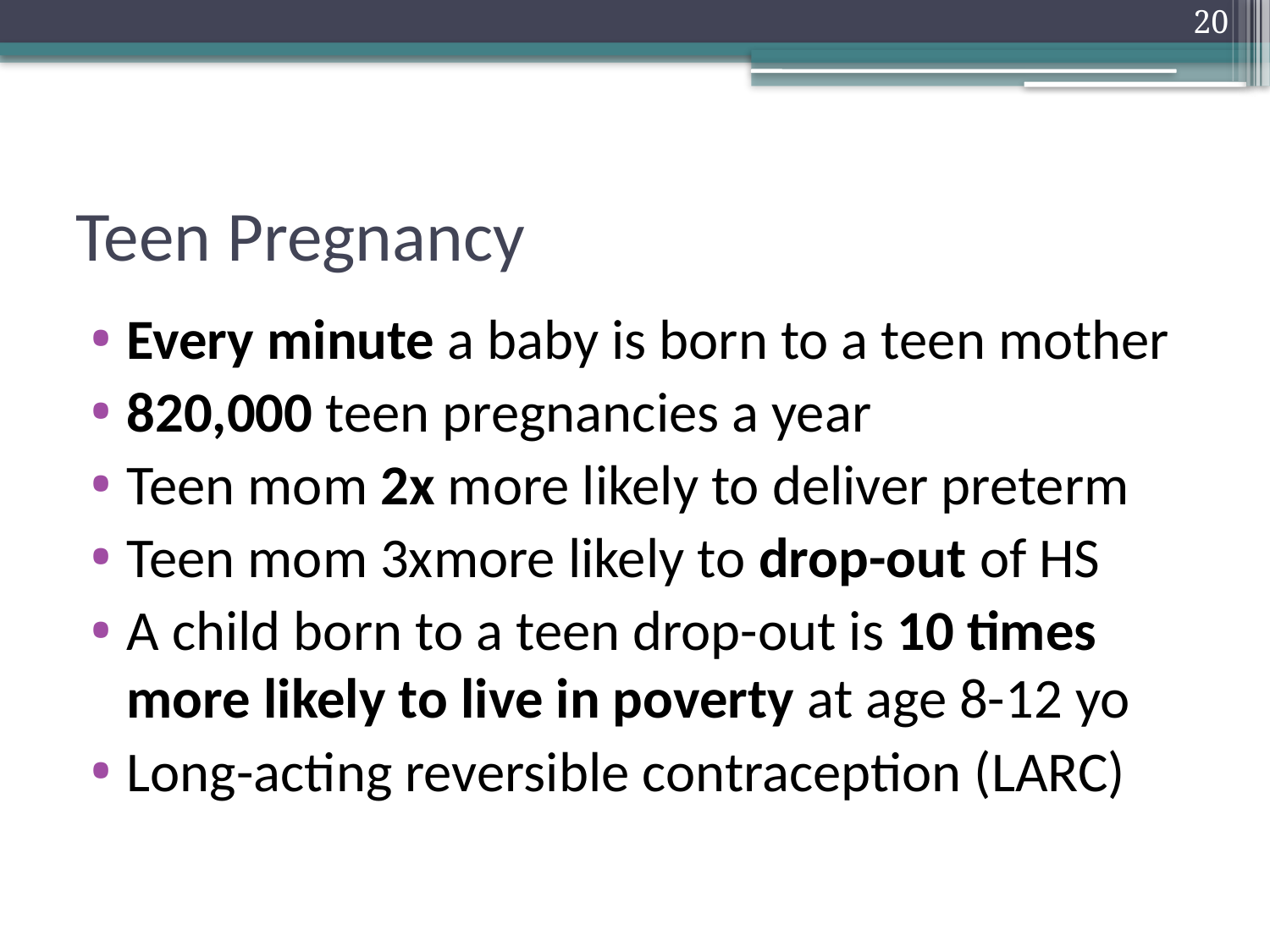

20
# Teen Pregnancy
Every minute a baby is born to a teen mother
820,000 teen pregnancies a year
Teen mom 2x more likely to deliver preterm
Teen mom 3xmore likely to drop-out of HS
A child born to a teen drop-out is 10 times more likely to live in poverty at age 8-12 yo
Long-acting reversible contraception (LARC)

## Slide 21
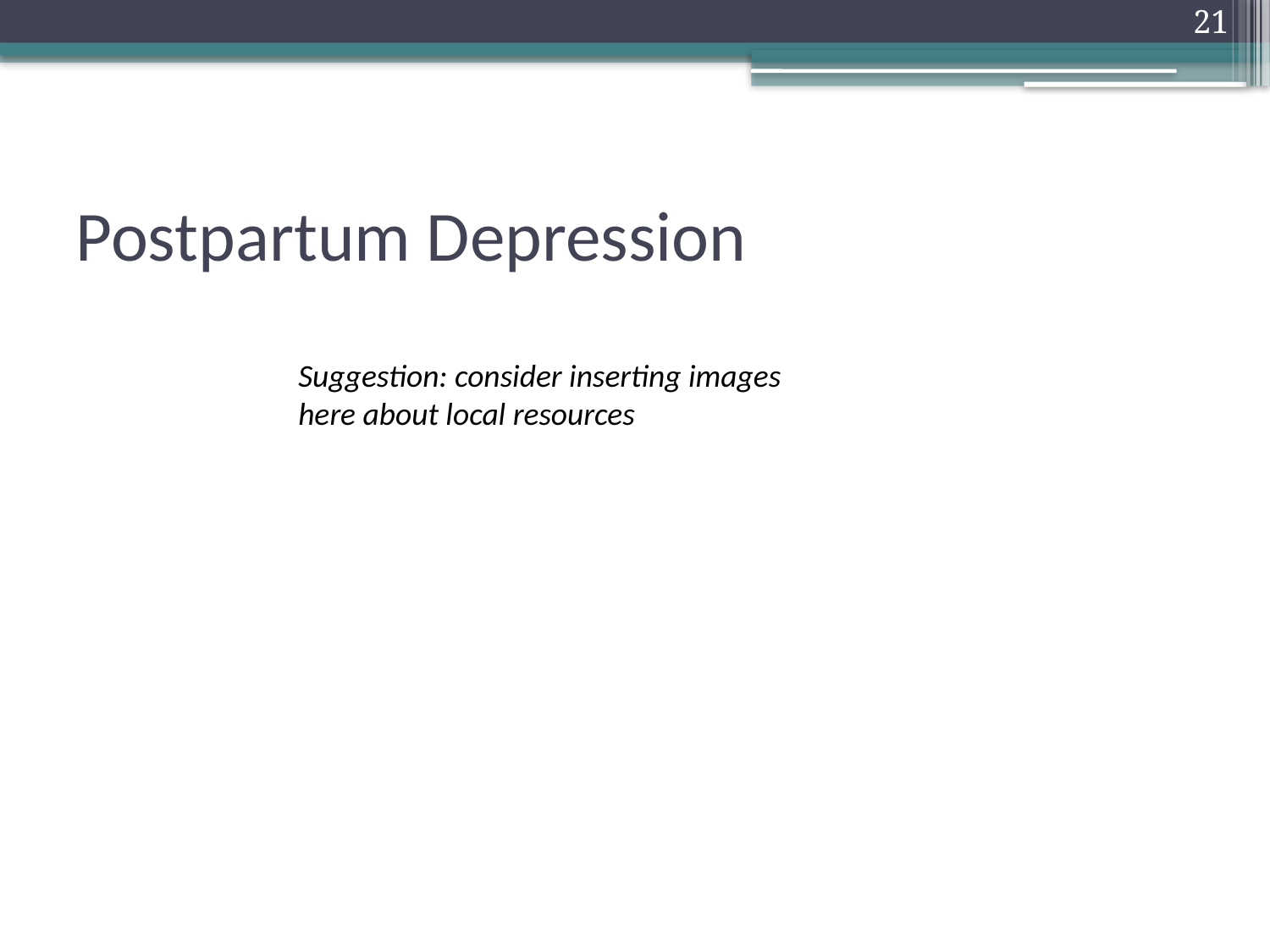

21
# Postpartum Depression
Suggestion: consider inserting images here about local resources

## Slide 22
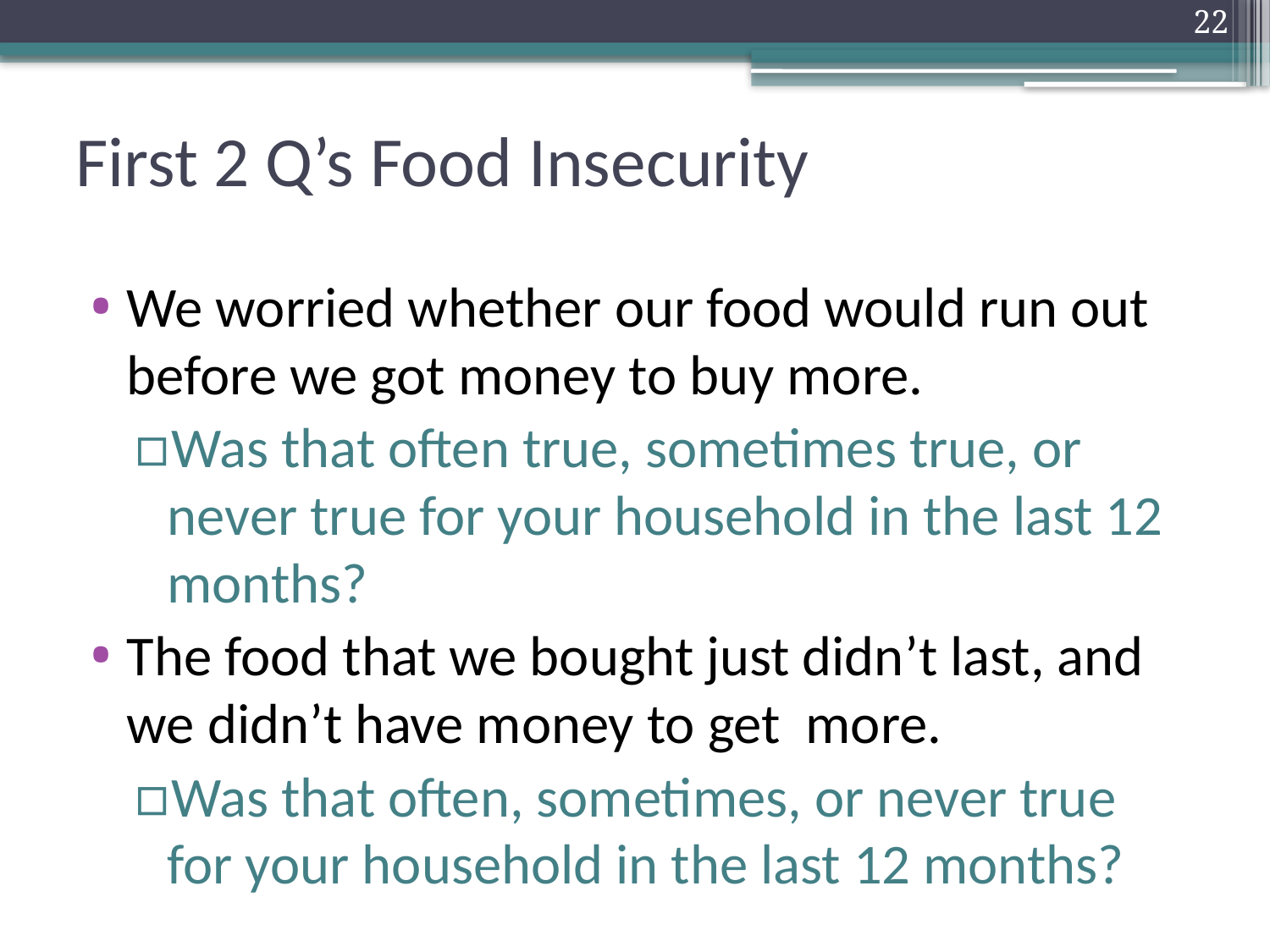

22
# First 2 Q’s Food Insecurity
We worried whether our food would run out before we got money to buy more.
Was that often true, sometimes true, or never true for your household in the last 12 months?
The food that we bought just didn’t last, and we didn’t have money to get more.
Was that often, sometimes, or never true for your household in the last 12 months?

## Slide 23
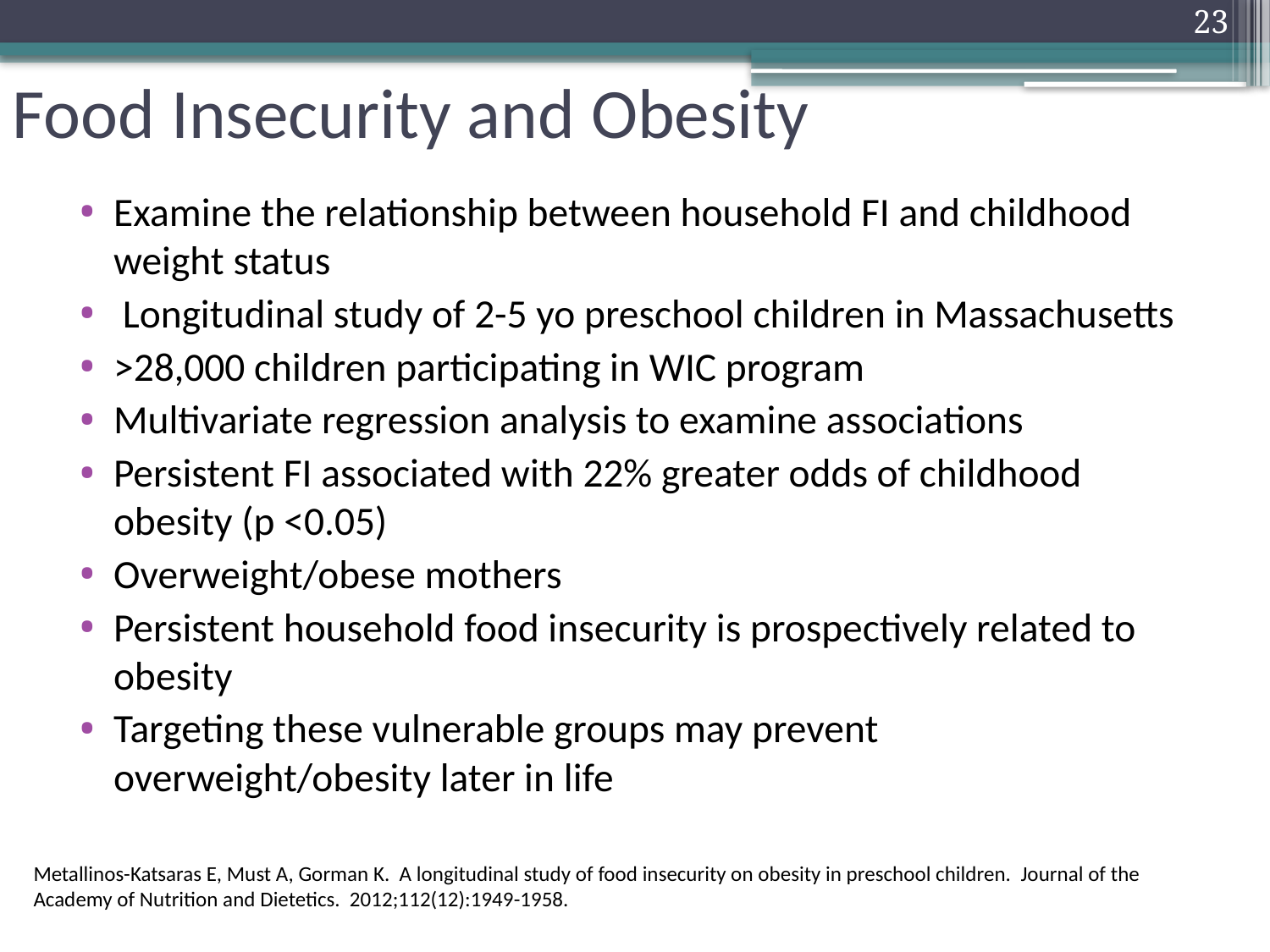

Food Insecurity and Obesity
23
Examine the relationship between household FI and childhood weight status
 Longitudinal study of 2-5 yo preschool children in Massachusetts
>28,000 children participating in WIC program
Multivariate regression analysis to examine associations
Persistent FI associated with 22% greater odds of childhood obesity (p <0.05)
Overweight/obese mothers
Persistent household food insecurity is prospectively related to obesity
Targeting these vulnerable groups may prevent overweight/obesity later in life
Metallinos-Katsaras E, Must A, Gorman K. A longitudinal study of food insecurity on obesity in preschool children. Journal of the Academy of Nutrition and Dietetics. 2012;112(12):1949-1958.

## Slide 24
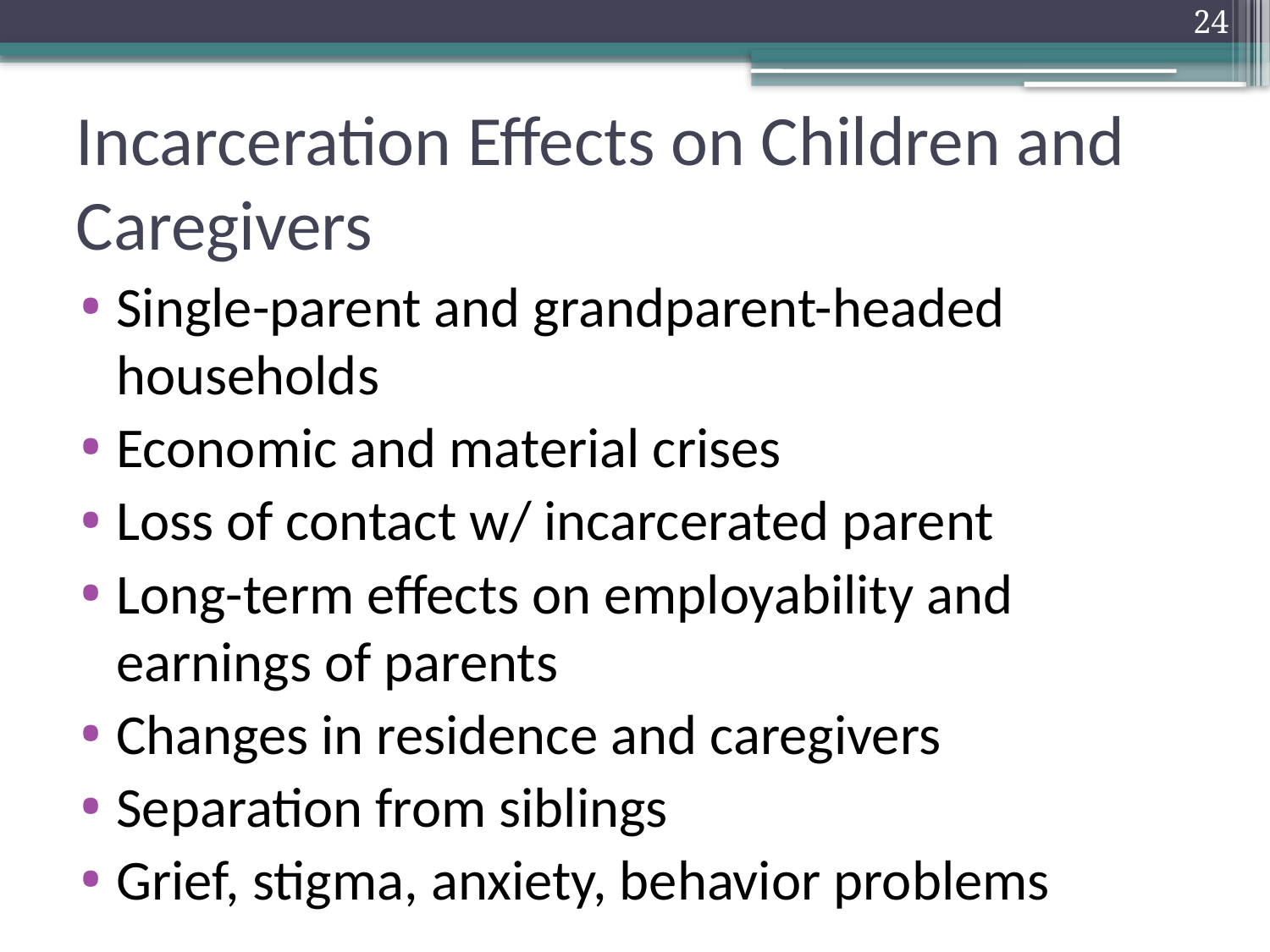

24
# Incarceration Effects on Children and Caregivers
Single-parent and grandparent-headed households
Economic and material crises
Loss of contact w/ incarcerated parent
Long-term effects on employability and earnings of parents
Changes in residence and caregivers
Separation from siblings
Grief, stigma, anxiety, behavior problems

## Slide 25
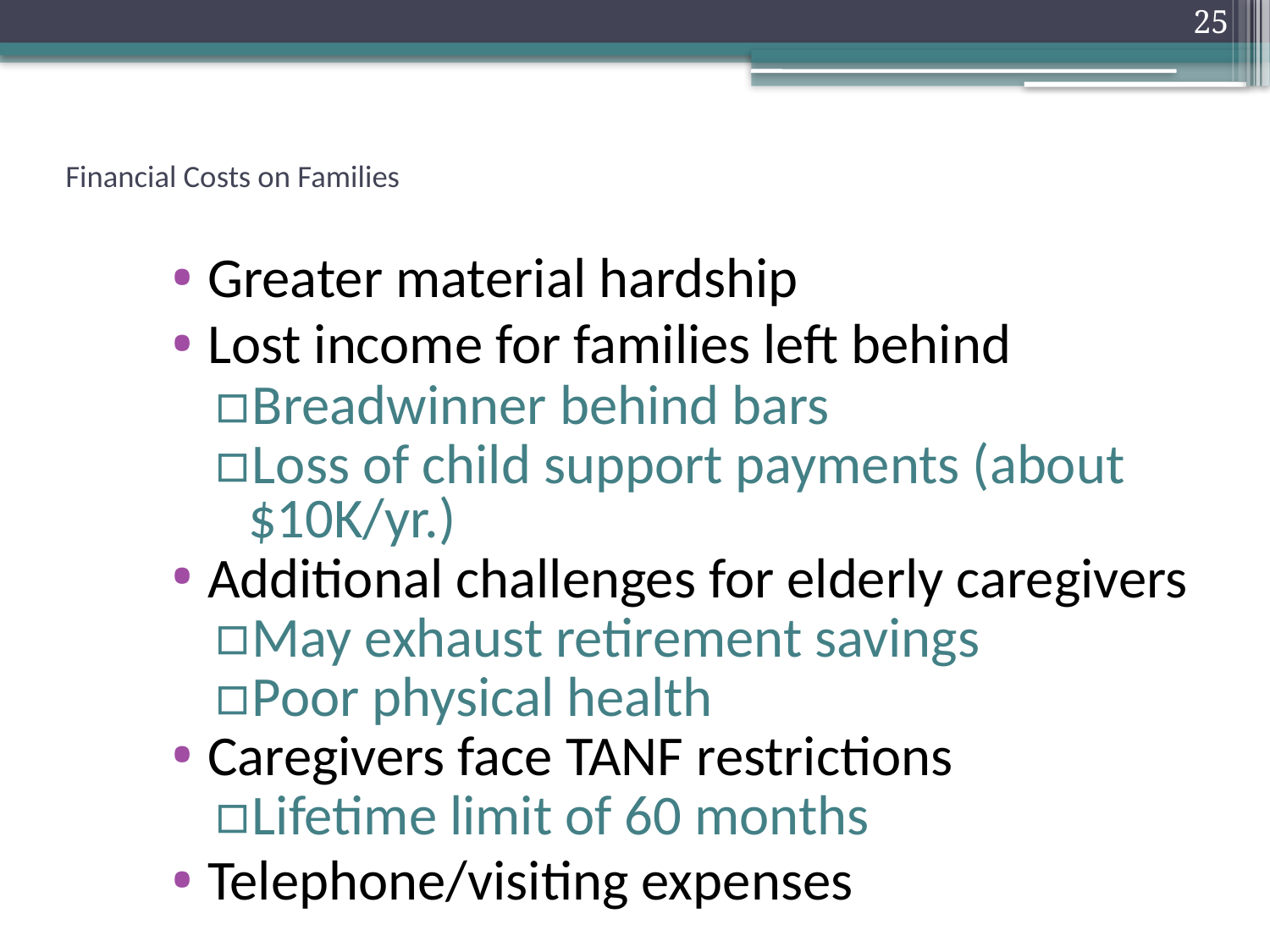

25
# Financial Costs on Families
Greater material hardship
Lost income for families left behind
Breadwinner behind bars
Loss of child support payments (about $10K/yr.)
Additional challenges for elderly caregivers
May exhaust retirement savings
Poor physical health
Caregivers face TANF restrictions
Lifetime limit of 60 months
Telephone/visiting expenses

## Slide 26
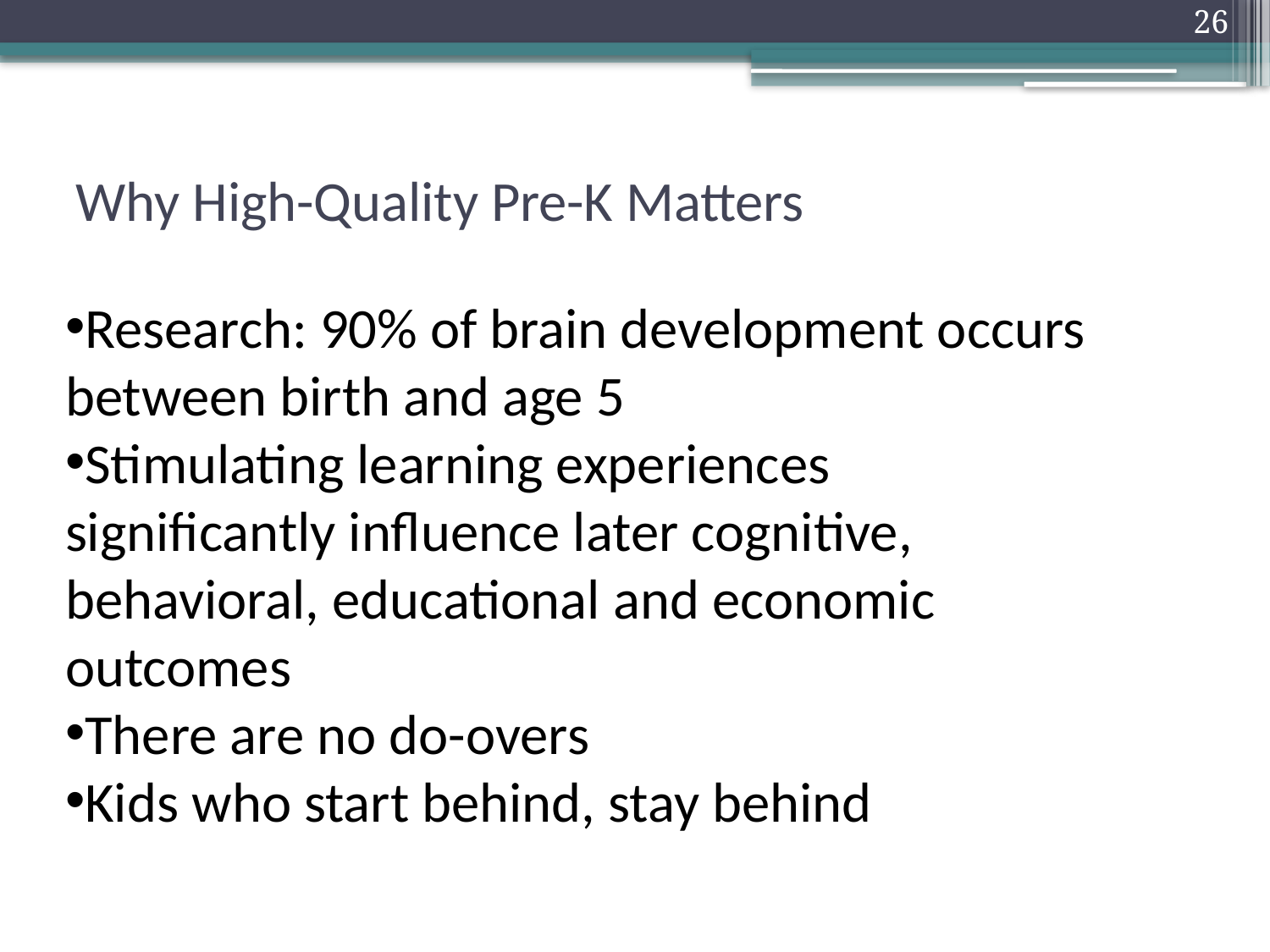

26
# Why High-Quality Pre-K Matters
Research: 90% of brain development occurs between birth and age 5
Stimulating learning experiences significantly influence later cognitive, behavioral, educational and economic outcomes
There are no do-overs
Kids who start behind, stay behind

## Slide 27
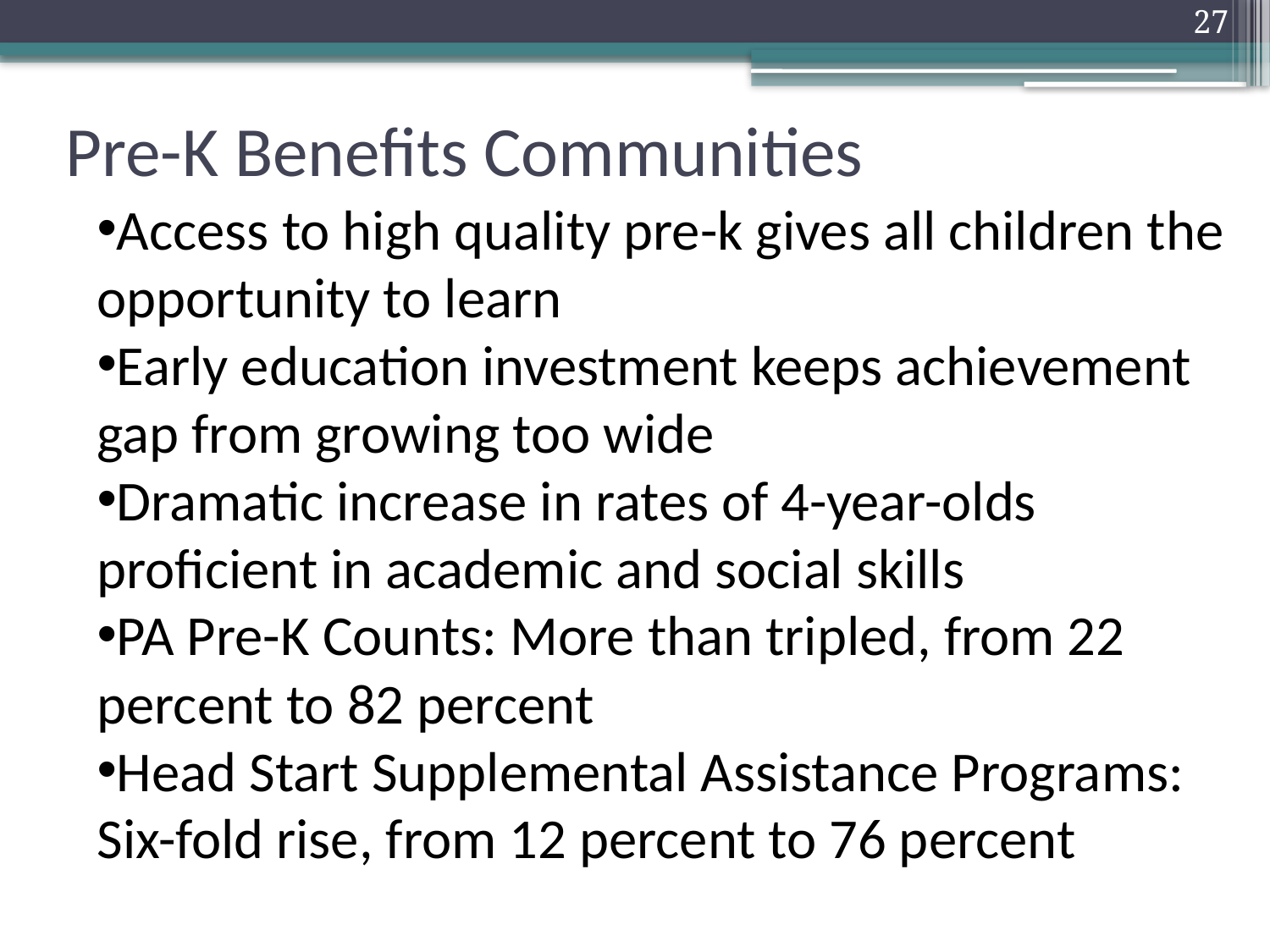

27
# Pre-K Benefits Communities
Access to high quality pre-k gives all children the opportunity to learn
Early education investment keeps achievement gap from growing too wide
Dramatic increase in rates of 4-year-olds proficient in academic and social skills
PA Pre-K Counts: More than tripled, from 22 percent to 82 percent
Head Start Supplemental Assistance Programs: Six-fold rise, from 12 percent to 76 percent

## Slide 28
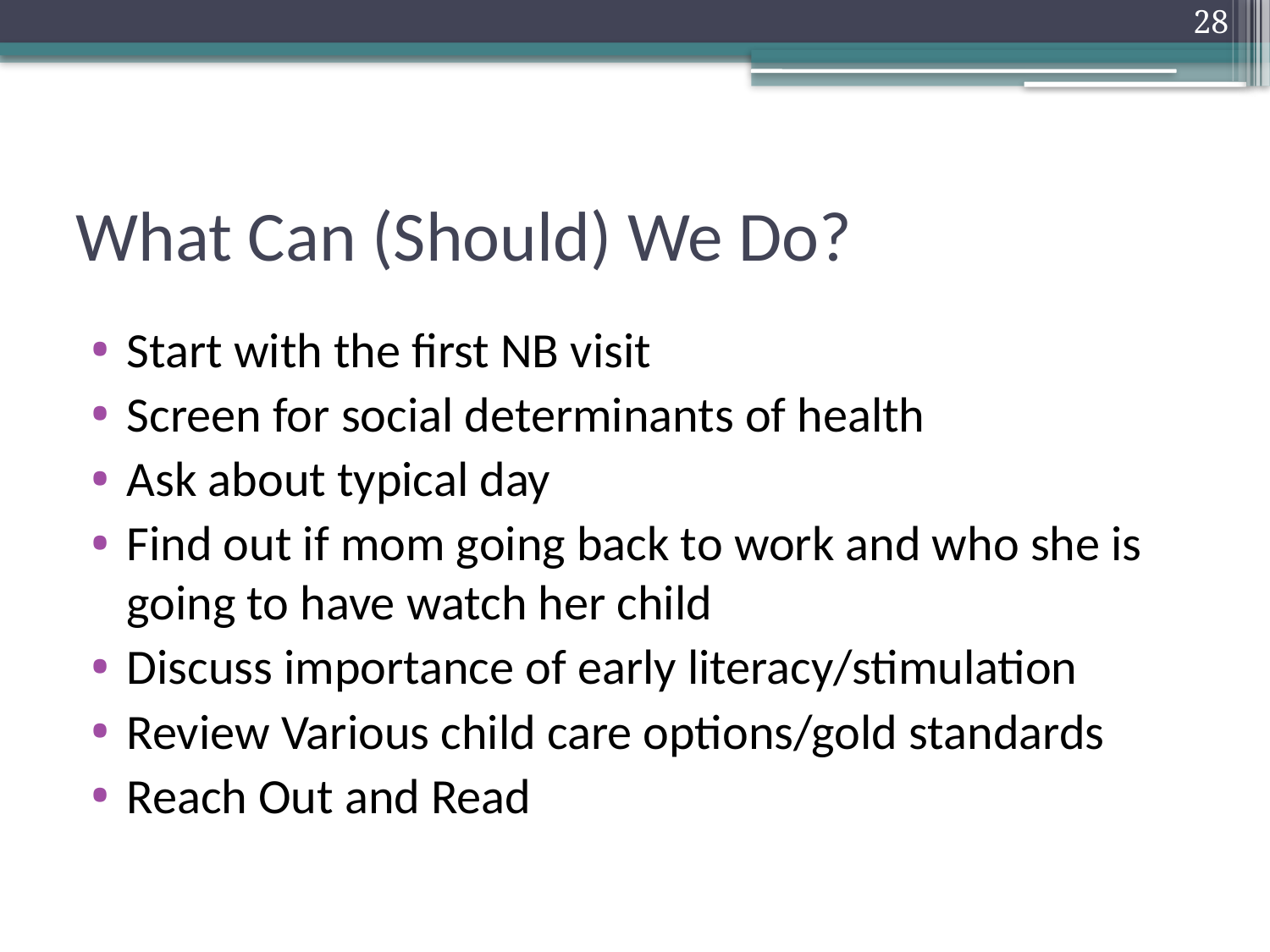

28
# What Can (Should) We Do?
Start with the first NB visit
Screen for social determinants of health
Ask about typical day
Find out if mom going back to work and who she is going to have watch her child
Discuss importance of early literacy/stimulation
Review Various child care options/gold standards
Reach Out and Read

## Slide 29
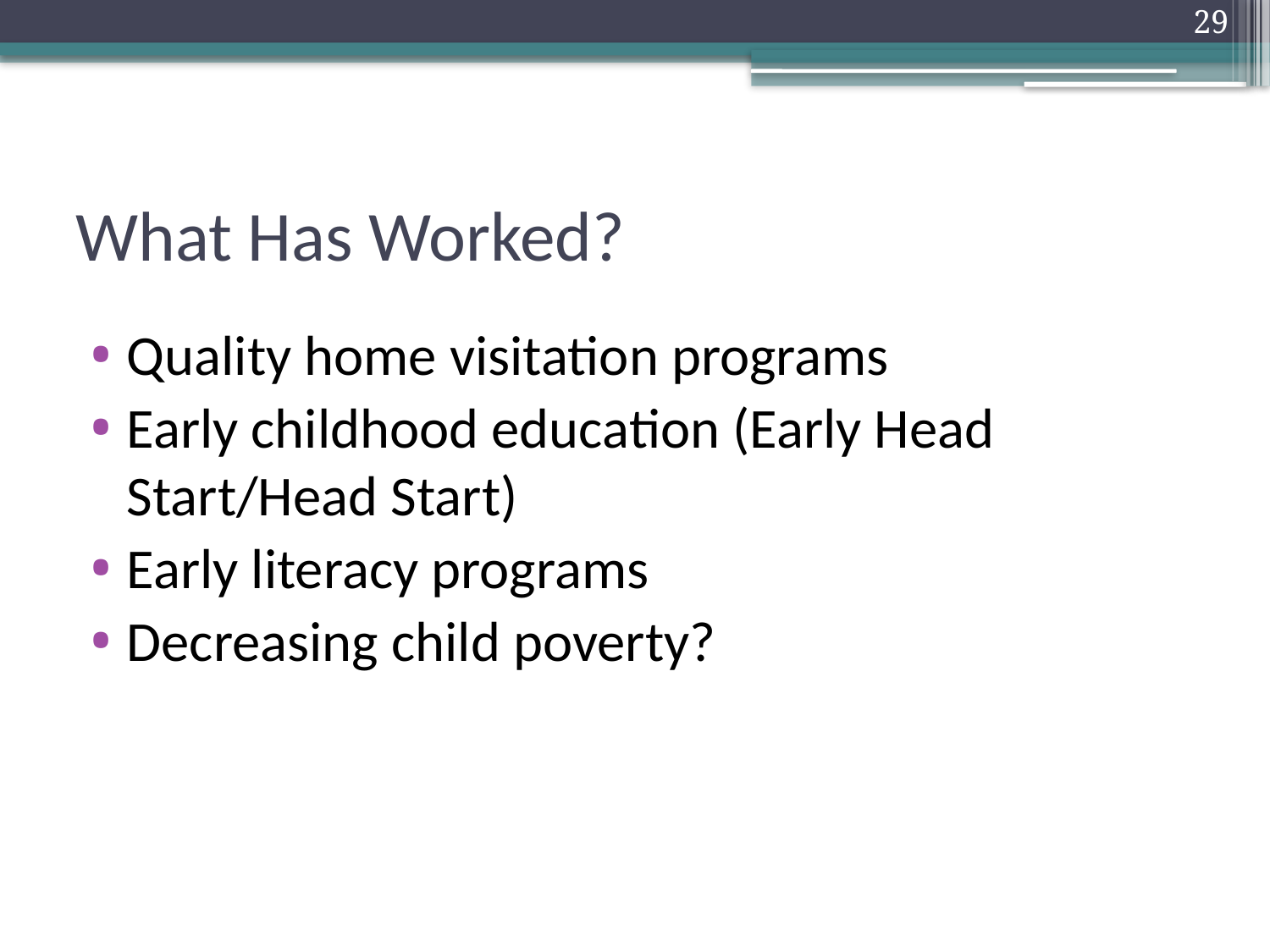

29
# What Has Worked?
Quality home visitation programs
Early childhood education (Early Head Start/Head Start)
Early literacy programs
Decreasing child poverty?
