# Supplementary material for: The JeffSTARS Advocacy and Community Partnership Elective: A Closer Look at Child Health Advocacy in Action
Source: MedEdPORTAL. 2016 Dec 31;12:10526. doi: 10.15766/mep_2374-8265.10526 (PMC6365684; doi:10.15766/mep_2374-8265.10526)
Supplement: Supplementary file 1 — A. CM1. Course Implementation at New Institution Checklist.docx B. CM2. Elective Checklist.docx C. CM3. Sample Schedule.docx D. CM4. Seminar Topic List With Learning Objectives.docx E. CM5. Syllabus Bibliography.docx F. CM6. List of Community Partners.docx G. CM7. Orientation for New Community Partner.docx H. CM8. Selected Past Projects.docx I. CM9. Sample Fact Sheets for Legislative Visits.docx J. Seminar Materials folder K. ET1. Advocacy Elective Assessment 1.pdf L. ET2. Advocacy Elective Assessment 2.pdf M. ET3. Trainee Evaluation by Community or Faculty Mentor.docx N. ET4. Trainee Evaluation of Seminar.docx O. ET5. Trainee Evaluation of Community Partner.docx P. ET6. Final Report Template.docx Q. Selected Trainee Abstracts and Presented Results folder [file mep-12-10526-s001.zip › J._Seminar_Materials_folder/13._Refugee_and_Immigrant_Health.pptx]

## Slide 1
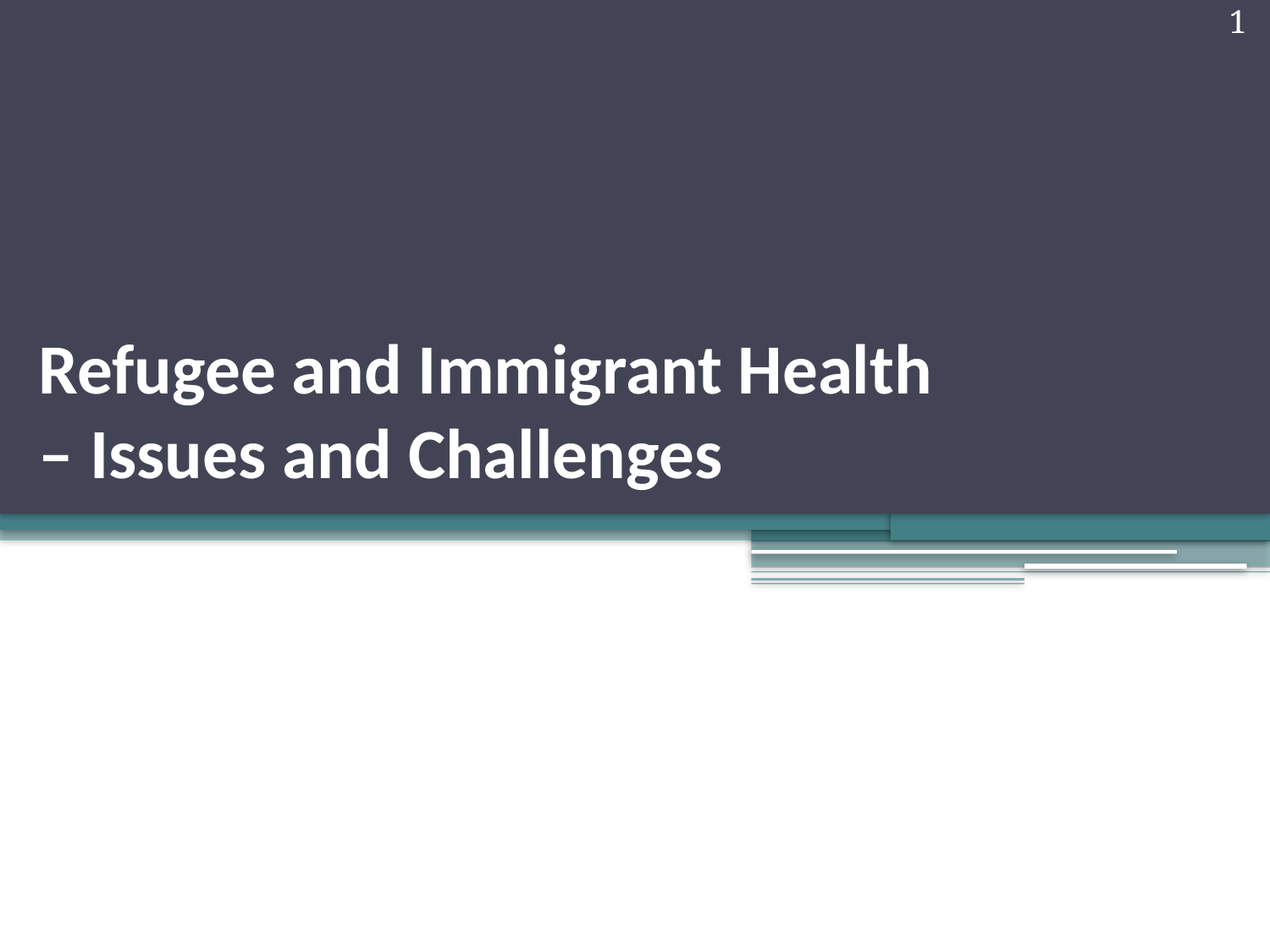

1
Refugee and Immigrant Health
– Issues and Challenges
# Advocacy Seminar: Refugee & Immigrant Health

## Slide 2
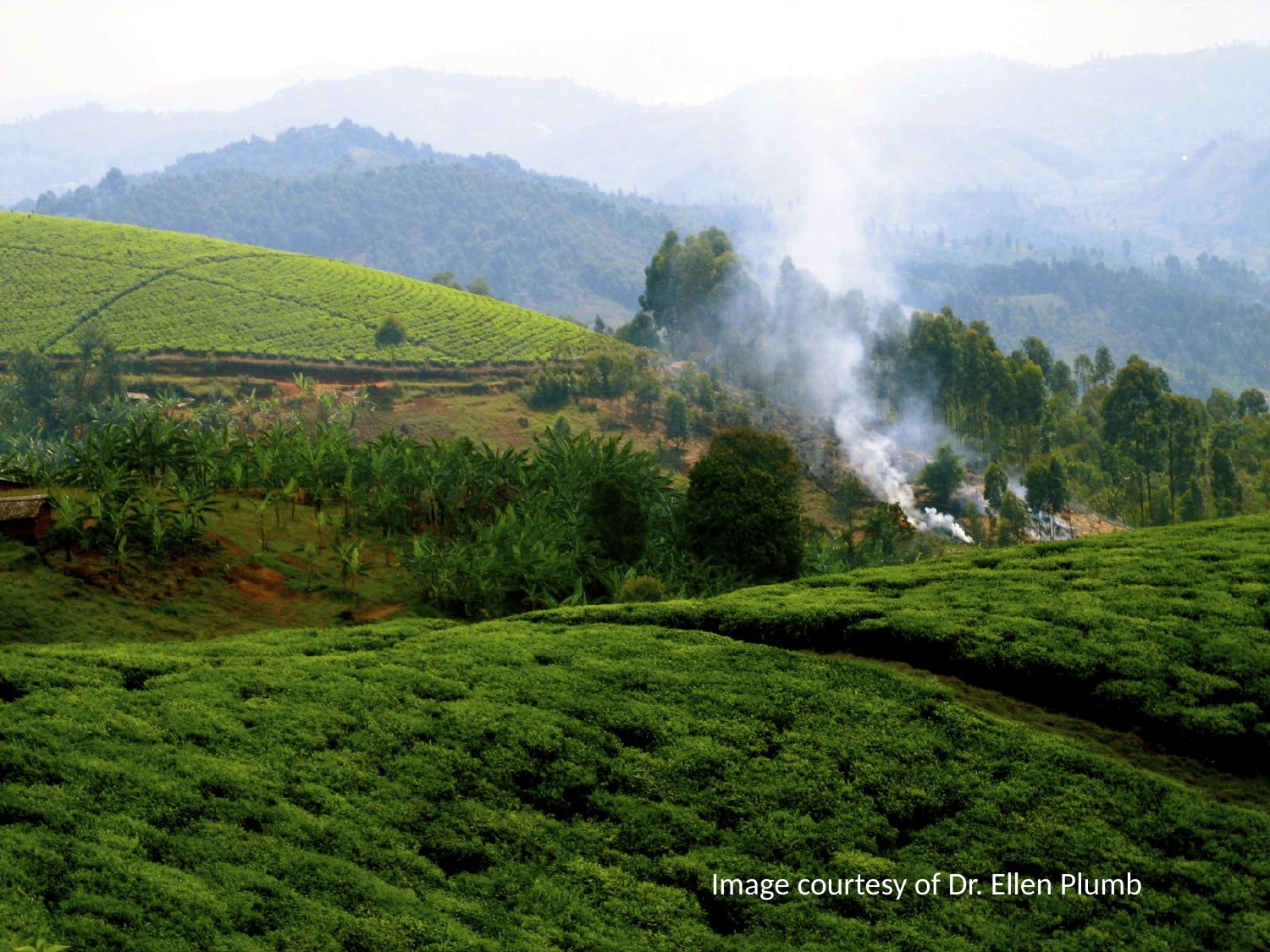

2
Image courtesy of Dr. Ellen Plumb

## Slide 3
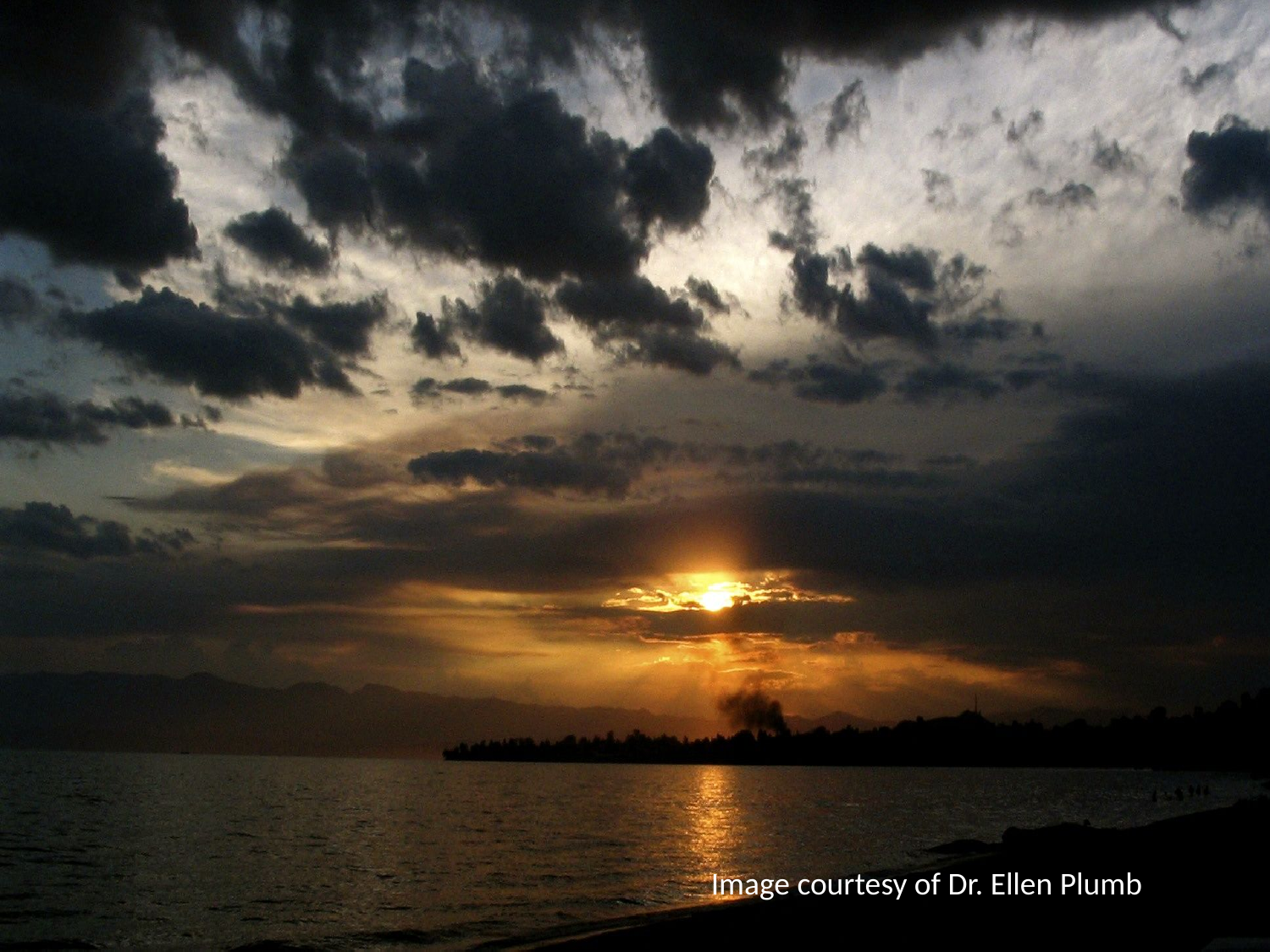

3
Image courtesy of Dr. Ellen Plumb

## Slide 4
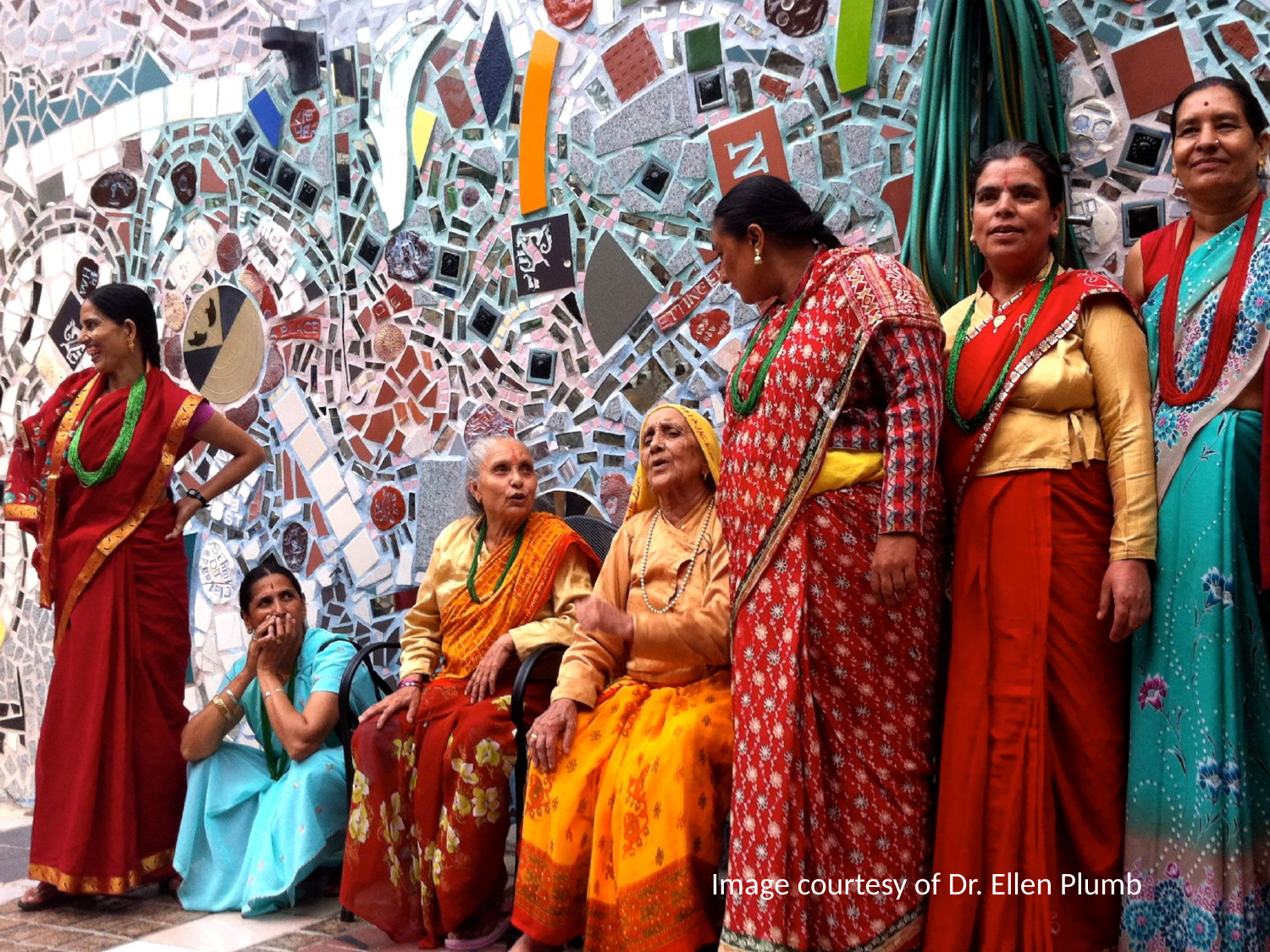

4
Image courtesy of Dr. Ellen Plumb

## Slide 5
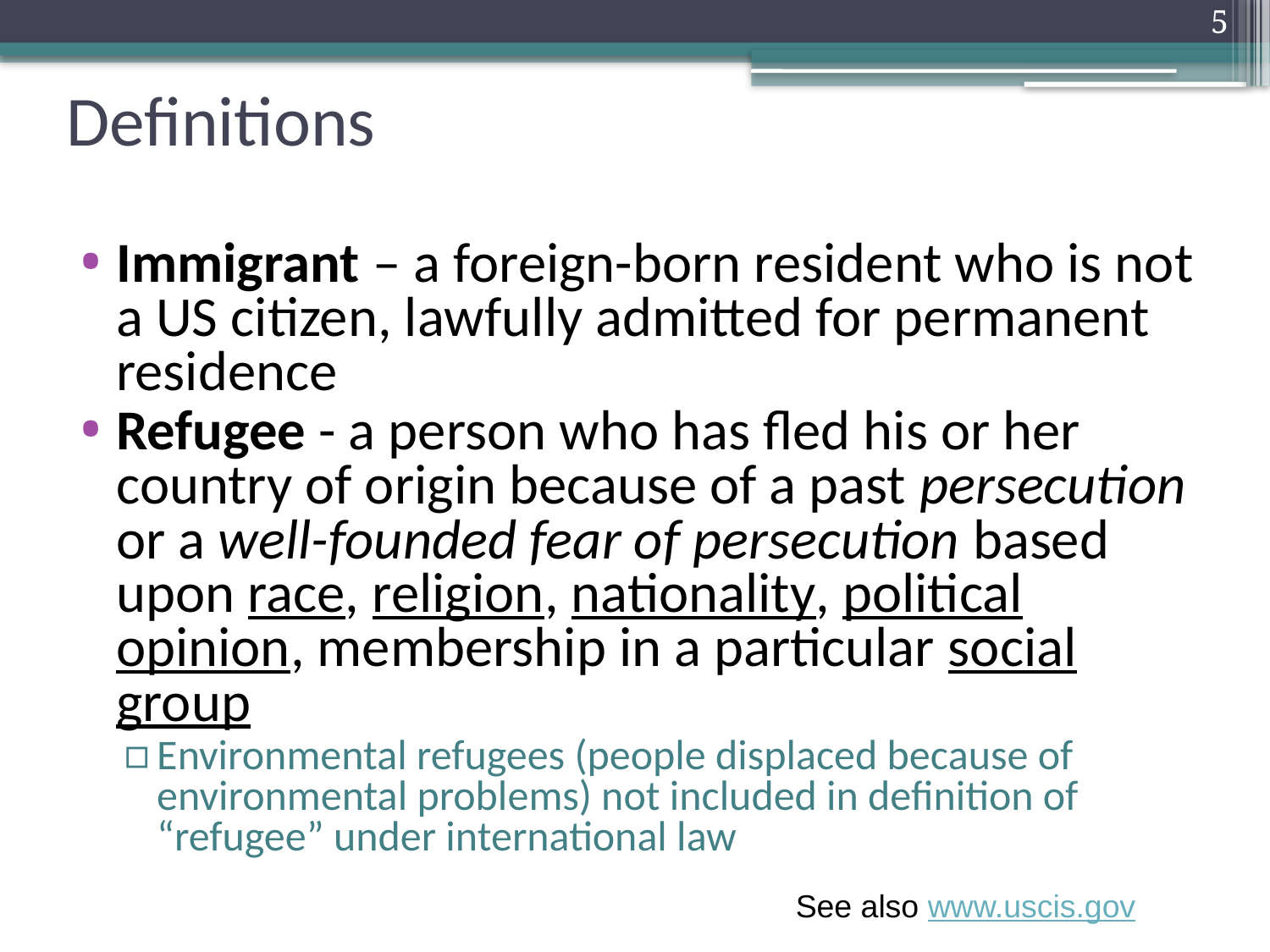

5
Definitions
Immigrant – a foreign-born resident who is not a US citizen, lawfully admitted for permanent residence
Refugee - a person who has fled his or her country of origin because of a past persecution or a well-founded fear of persecution based upon race, religion, nationality, political opinion, membership in a particular social group
Environmental refugees (people displaced because of environmental problems) not included in definition of “refugee” under international law
See also www.uscis.gov

## Slide 6
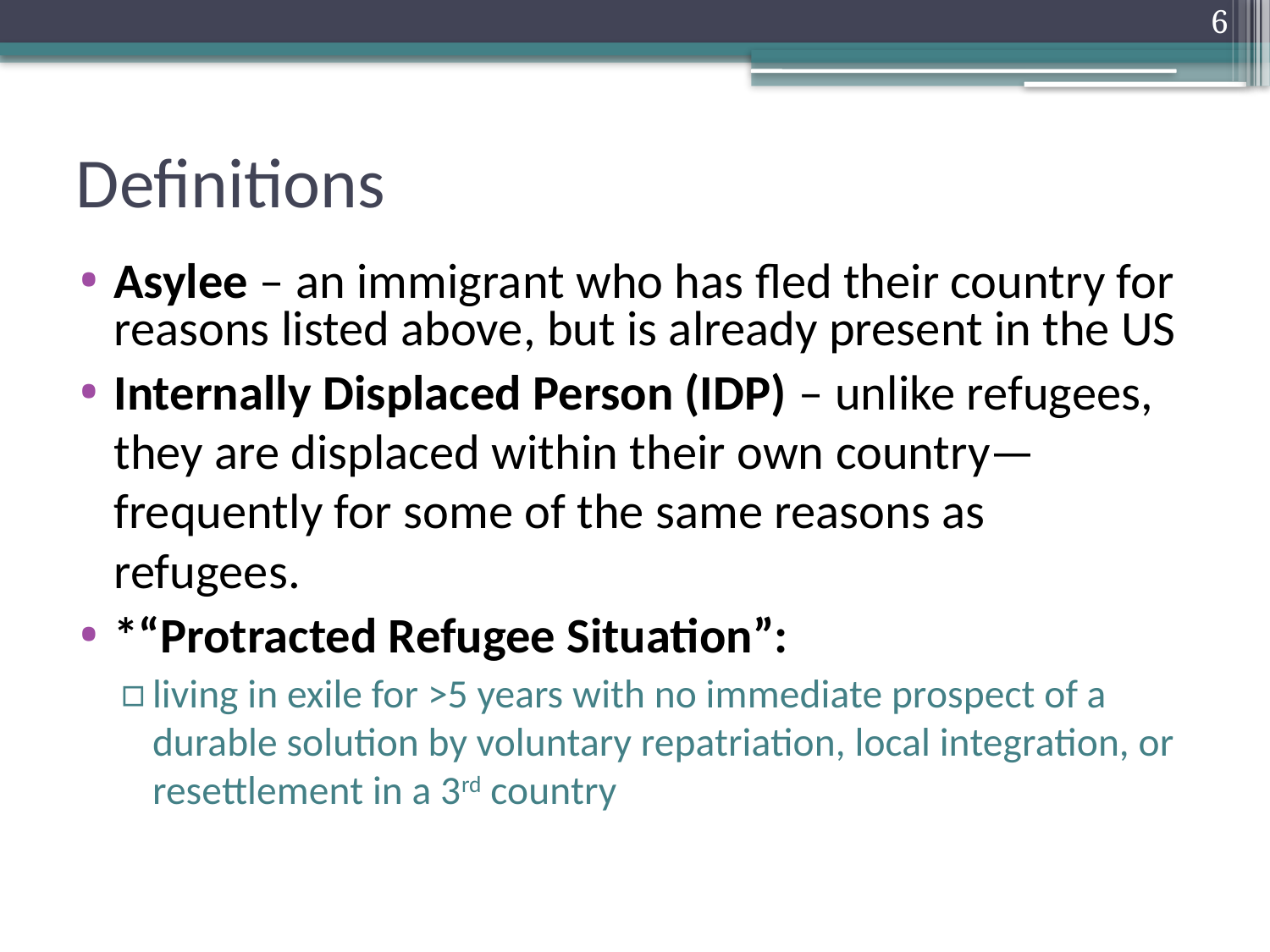

6
# Definitions
Asylee – an immigrant who has fled their country for reasons listed above, but is already present in the US
Internally Displaced Person (IDP) – unlike refugees, they are displaced within their own country—frequently for some of the same reasons as refugees.
*“Protracted Refugee Situation”:
living in exile for >5 years with no immediate prospect of a durable solution by voluntary repatriation, local integration, or resettlement in a 3rd country

## Slide 7
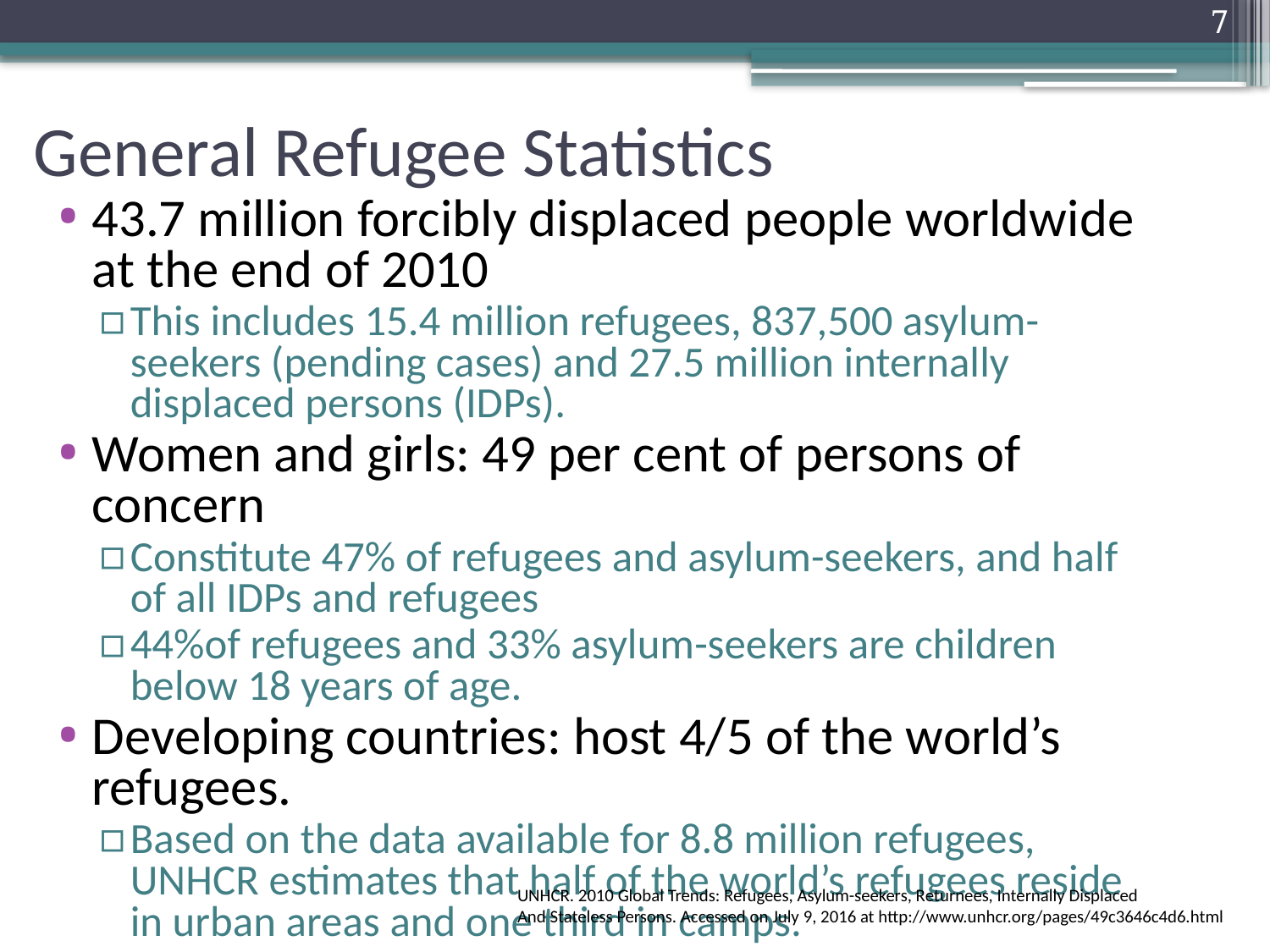

7
# General Refugee Statistics
43.7 million forcibly displaced people worldwide at the end of 2010
This includes 15.4 million refugees, 837,500 asylum-seekers (pending cases) and 27.5 million internally displaced persons (IDPs).
Women and girls: 49 per cent of persons of concern
Constitute 47% of refugees and asylum-seekers, and half of all IDPs and refugees
44%of refugees and 33% asylum-seekers are children below 18 years of age.
Developing countries: host 4/5 of the world’s refugees.
Based on the data available for 8.8 million refugees, UNHCR estimates that half of the world’s refugees reside in urban areas and one third in camps.
UNHCR. 2010 Global Trends: Refugees, Asylum-seekers, Returnees, Internally Displaced
And Stateless Persons. Accessed on July 9, 2016 at http://www.unhcr.org/pages/49c3646c4d6.html

## Slide 8
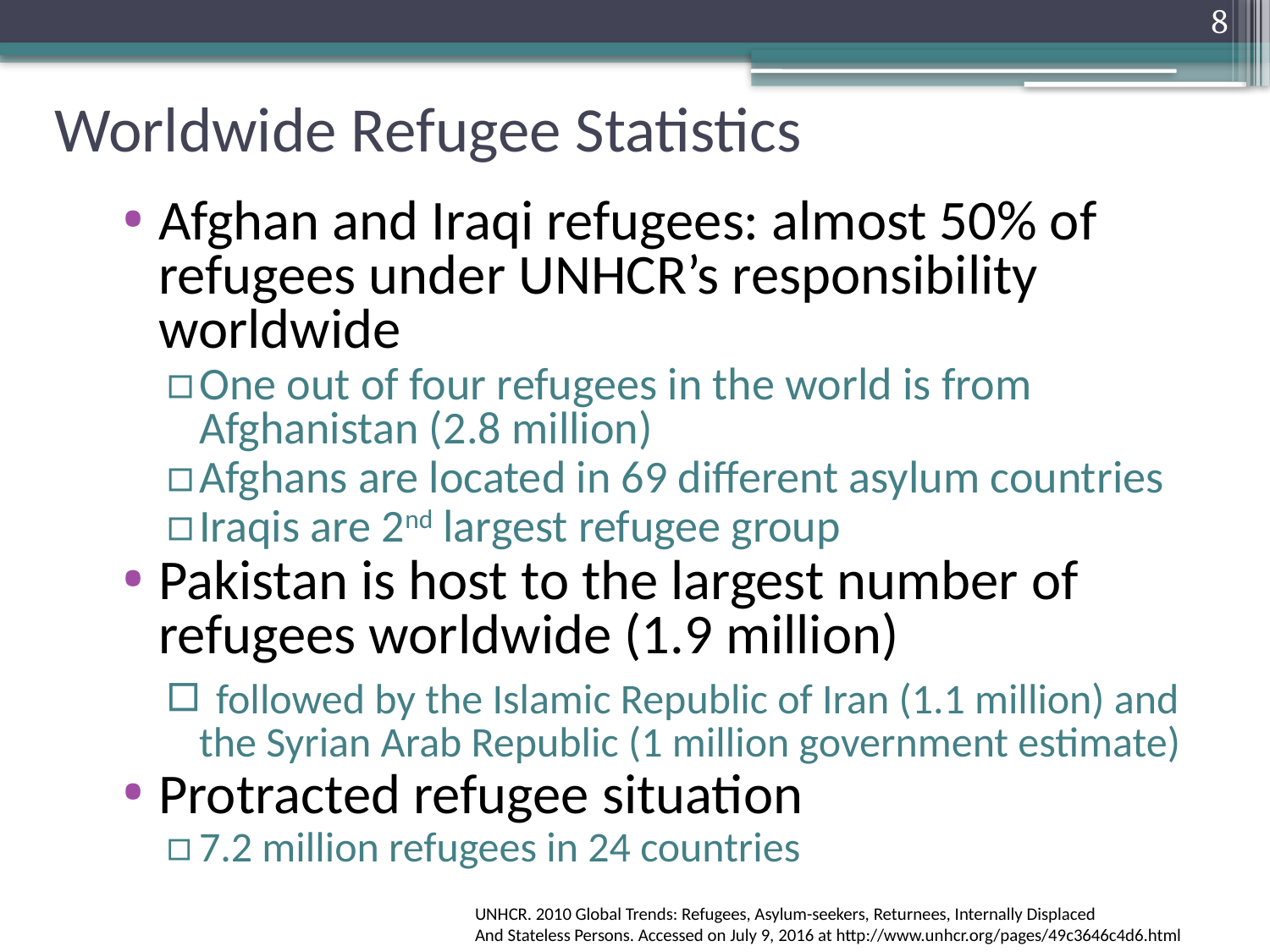

8
# Worldwide Refugee Statistics
Afghan and Iraqi refugees: almost 50% of refugees under UNHCR’s responsibility worldwide
One out of four refugees in the world is from Afghanistan (2.8 million)
Afghans are located in 69 different asylum countries
Iraqis are 2nd largest refugee group
Pakistan is host to the largest number of refugees worldwide (1.9 million)
 followed by the Islamic Republic of Iran (1.1 million) and the Syrian Arab Republic (1 million government estimate)
Protracted refugee situation
7.2 million refugees in 24 countries
UNHCR. 2010 Global Trends: Refugees, Asylum-seekers, Returnees, Internally Displaced
And Stateless Persons. Accessed on July 9, 2016 at http://www.unhcr.org/pages/49c3646c4d6.html

## Slide 9
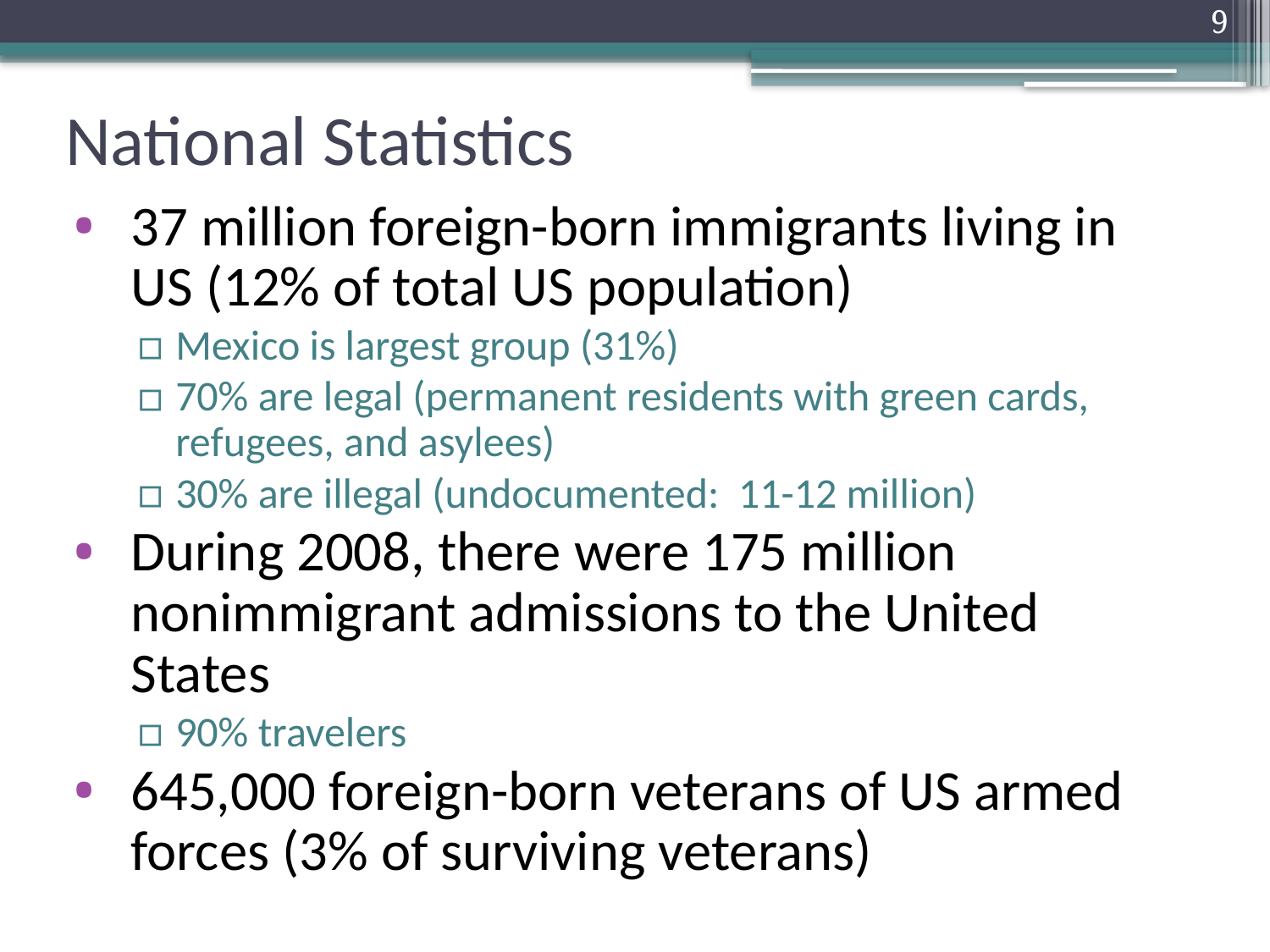

9
# National Statistics
37 million foreign-born immigrants living in US (12% of total US population)
Mexico is largest group (31%)
70% are legal (permanent residents with green cards, refugees, and asylees)
30% are illegal (undocumented: 11-12 million)
During 2008, there were 175 million nonimmigrant admissions to the United States
90% travelers
645,000 foreign-born veterans of US armed forces (3% of surviving veterans)

## Slide 10
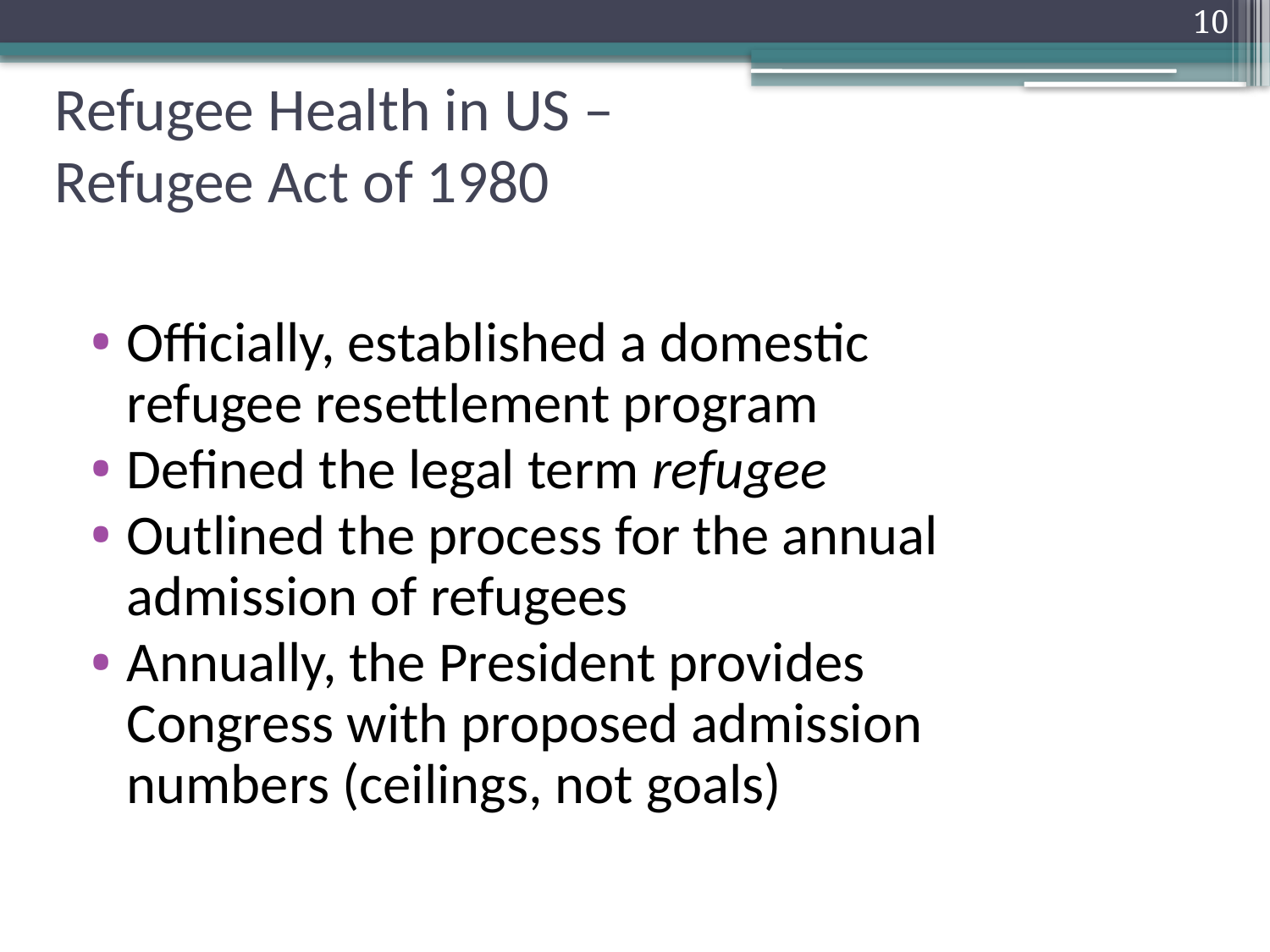

10
Refugee Health in US –
Refugee Act of 1980
Officially, established a domestic refugee resettlement program
Defined the legal term refugee
Outlined the process for the annual admission of refugees
Annually, the President provides Congress with proposed admission numbers (ceilings, not goals)

## Slide 11
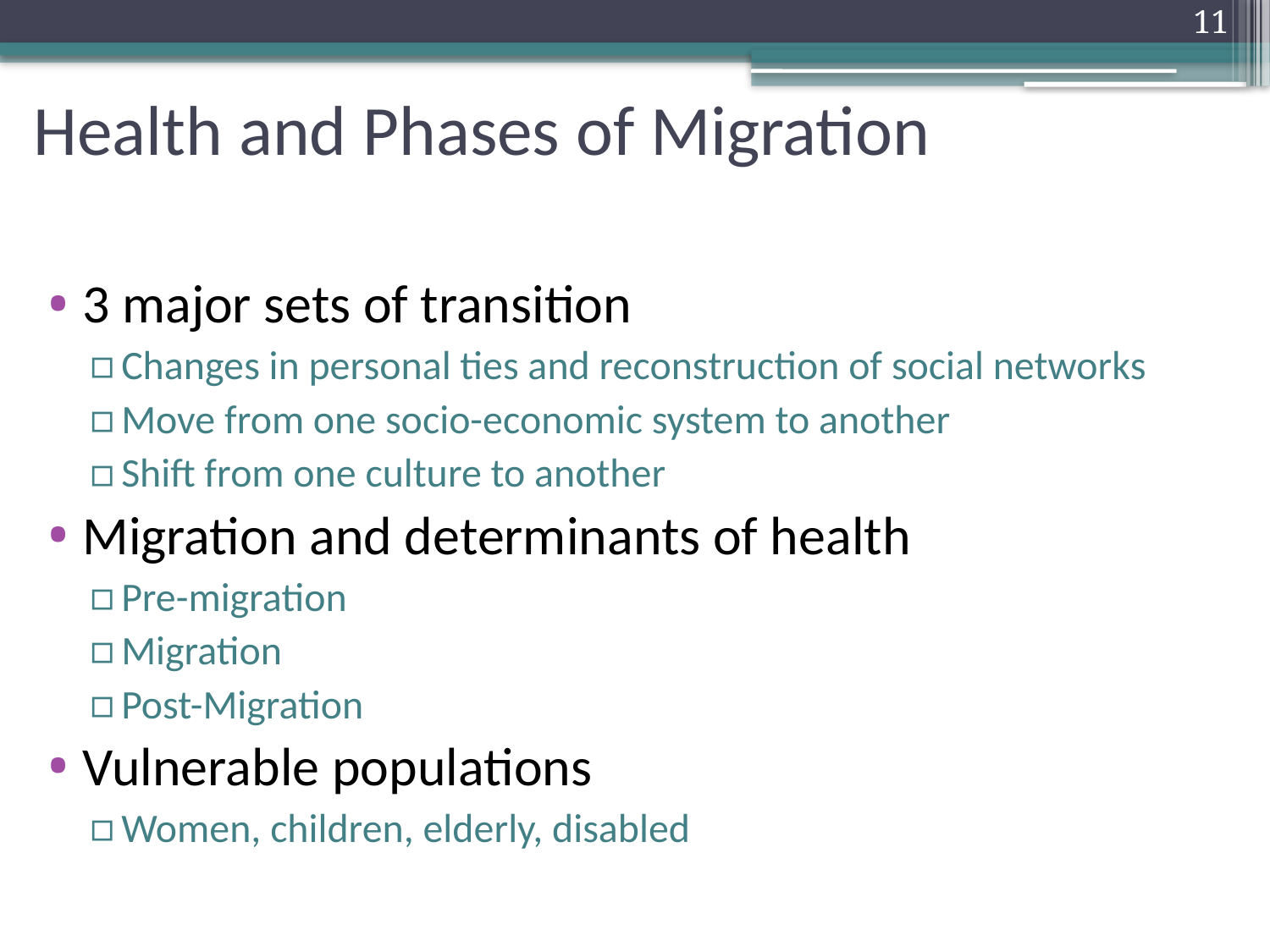

11
# Health and Phases of Migration
3 major sets of transition
Changes in personal ties and reconstruction of social networks
Move from one socio-economic system to another
Shift from one culture to another
Migration and determinants of health
Pre-migration
Migration
Post-Migration
Vulnerable populations
Women, children, elderly, disabled

## Slide 12
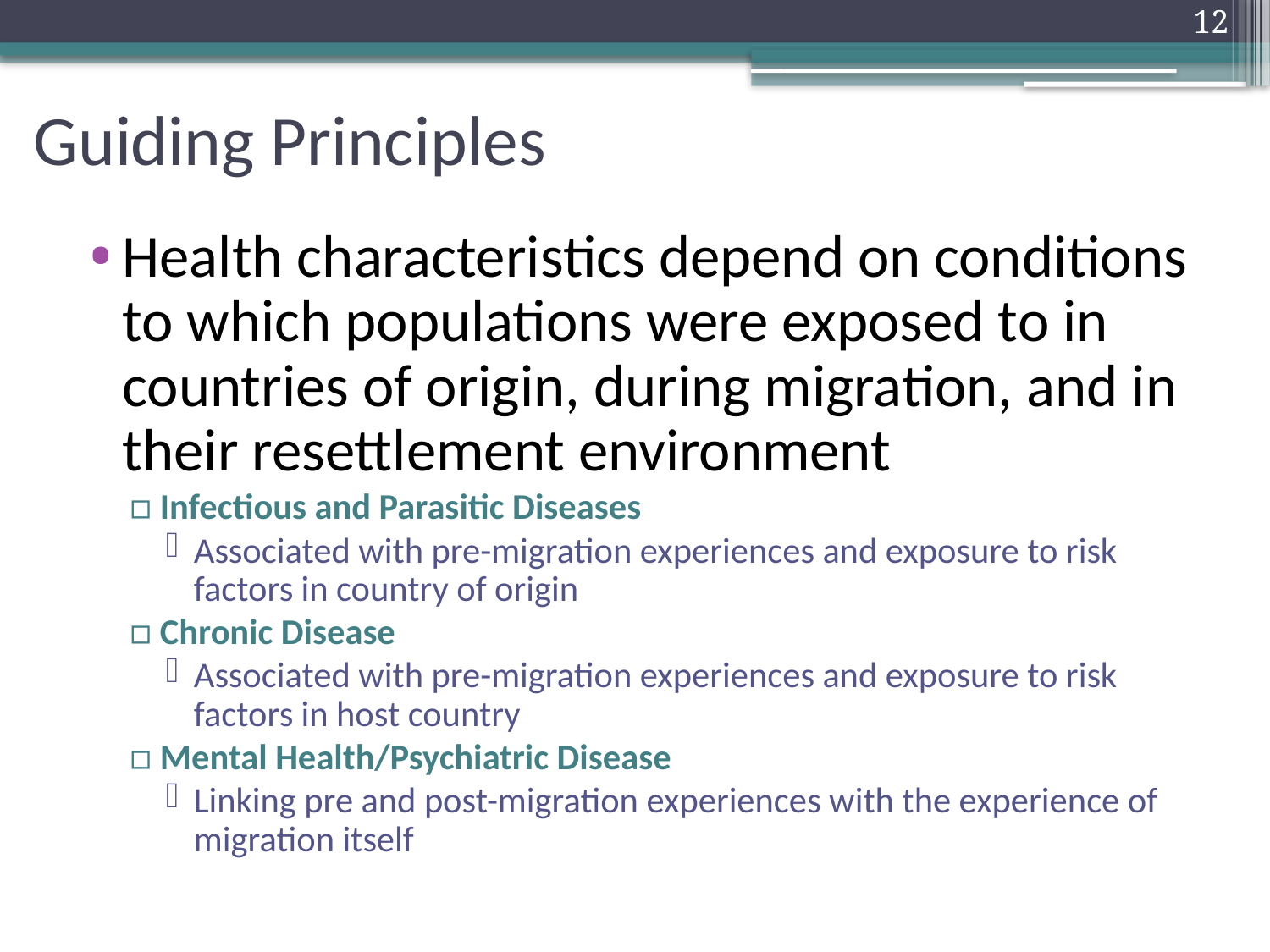

12
# Guiding Principles
Health characteristics depend on conditions to which populations were exposed to in countries of origin, during migration, and in their resettlement environment
Infectious and Parasitic Diseases
Associated with pre-migration experiences and exposure to risk factors in country of origin
Chronic Disease
Associated with pre-migration experiences and exposure to risk factors in host country
Mental Health/Psychiatric Disease
Linking pre and post-migration experiences with the experience of migration itself

## Slide 13
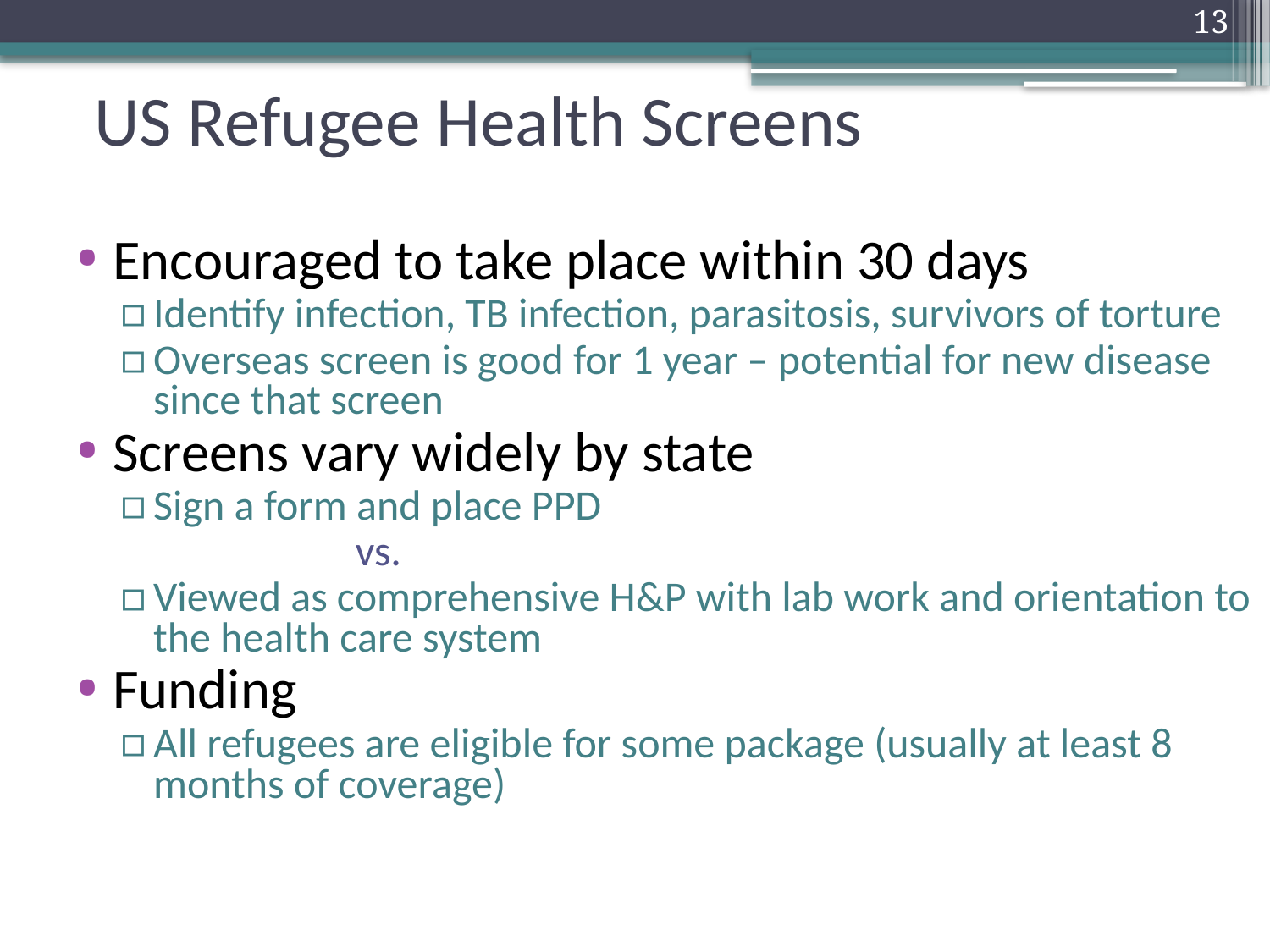

13
US Refugee Health Screens
Encouraged to take place within 30 days
Identify infection, TB infection, parasitosis, survivors of torture
Overseas screen is good for 1 year – potential for new disease since that screen
Screens vary widely by state
Sign a form and place PPD
 		 vs.
Viewed as comprehensive H&P with lab work and orientation to the health care system
Funding
All refugees are eligible for some package (usually at least 8 months of coverage)

## Slide 14
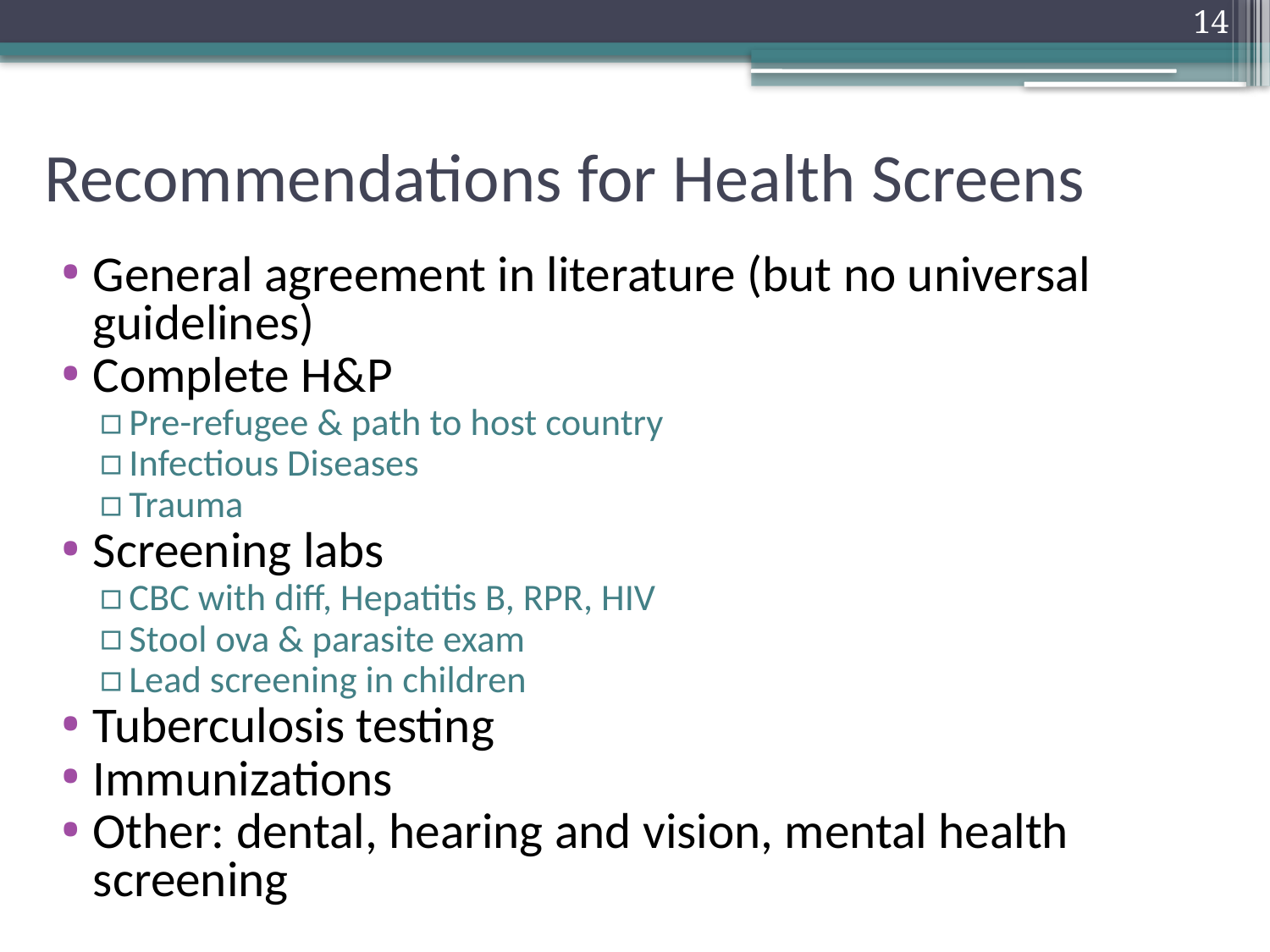

14
Recommendations for Health Screens
General agreement in literature (but no universal guidelines)
Complete H&P
Pre-refugee & path to host country
Infectious Diseases
Trauma
Screening labs
CBC with diff, Hepatitis B, RPR, HIV
Stool ova & parasite exam
Lead screening in children
Tuberculosis testing
Immunizations
Other: dental, hearing and vision, mental health screening

## Slide 15
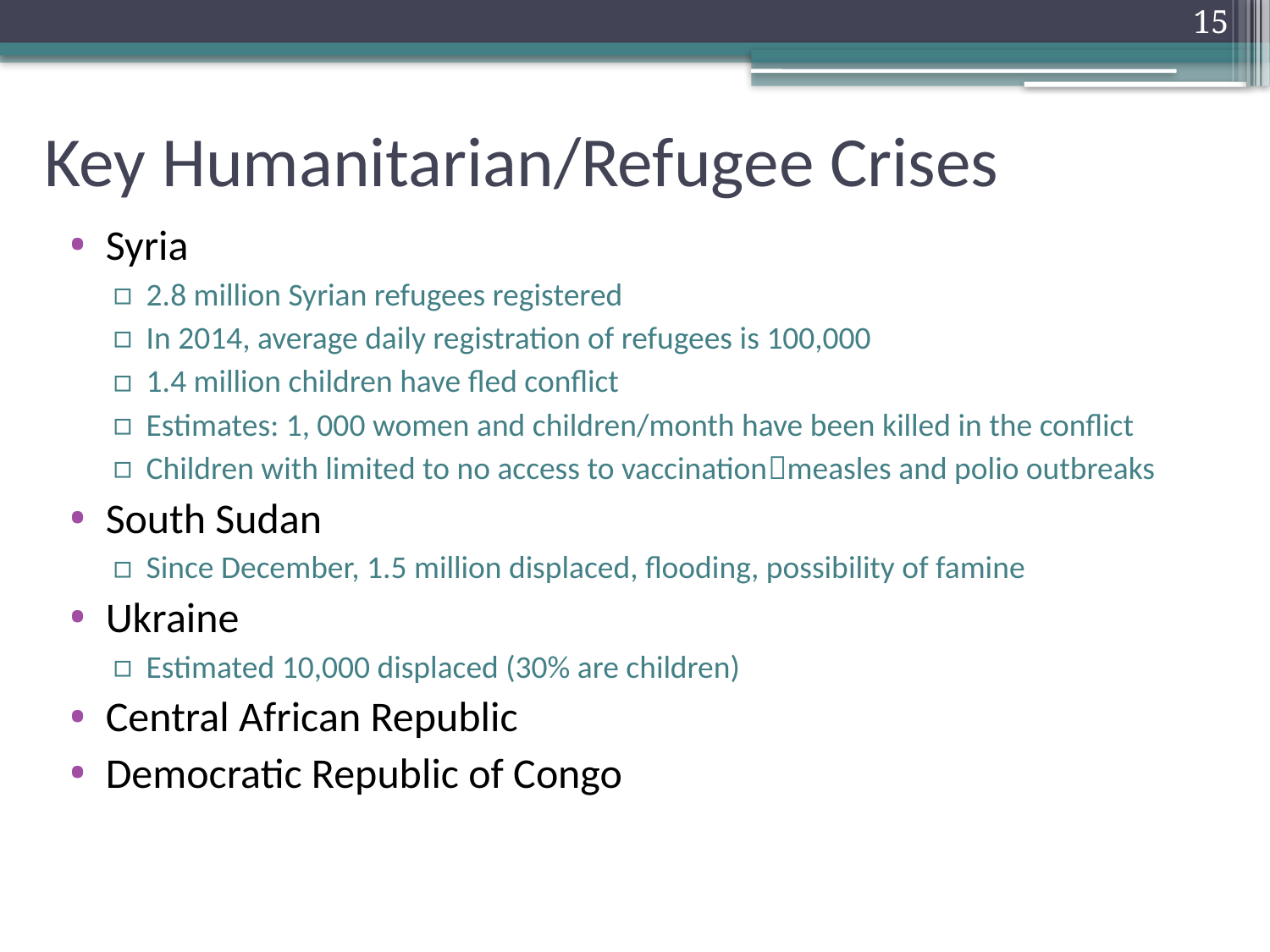

15
# Key Humanitarian/Refugee Crises
Syria
2.8 million Syrian refugees registered
In 2014, average daily registration of refugees is 100,000
1.4 million children have fled conflict
Estimates: 1, 000 women and children/month have been killed in the conflict
Children with limited to no access to vaccinationmeasles and polio outbreaks
South Sudan
Since December, 1.5 million displaced, flooding, possibility of famine
Ukraine
Estimated 10,000 displaced (30% are children)
Central African Republic
Democratic Republic of Congo

## Slide 16
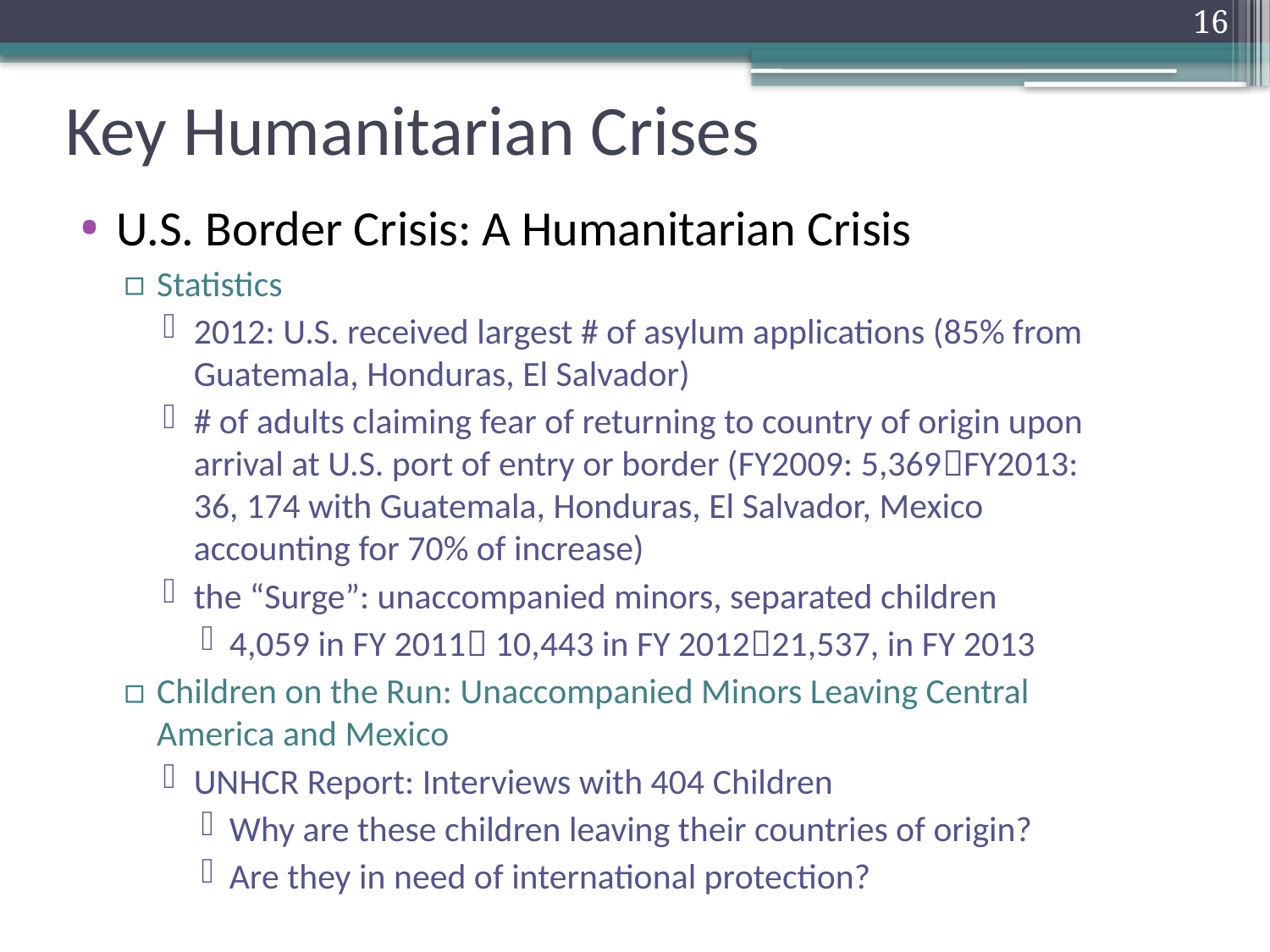

16
# Key Humanitarian Crises
U.S. Border Crisis: A Humanitarian Crisis
Statistics
2012: U.S. received largest # of asylum applications (85% from Guatemala, Honduras, El Salvador)
# of adults claiming fear of returning to country of origin upon arrival at U.S. port of entry or border (FY2009: 5,369FY2013: 36, 174 with Guatemala, Honduras, El Salvador, Mexico accounting for 70% of increase)
the “Surge”: unaccompanied minors, separated children
4,059 in FY 2011 10,443 in FY 201221,537, in FY 2013
Children on the Run: Unaccompanied Minors Leaving Central America and Mexico
UNHCR Report: Interviews with 404 Children
Why are these children leaving their countries of origin?
Are they in need of international protection?

## Slide 17
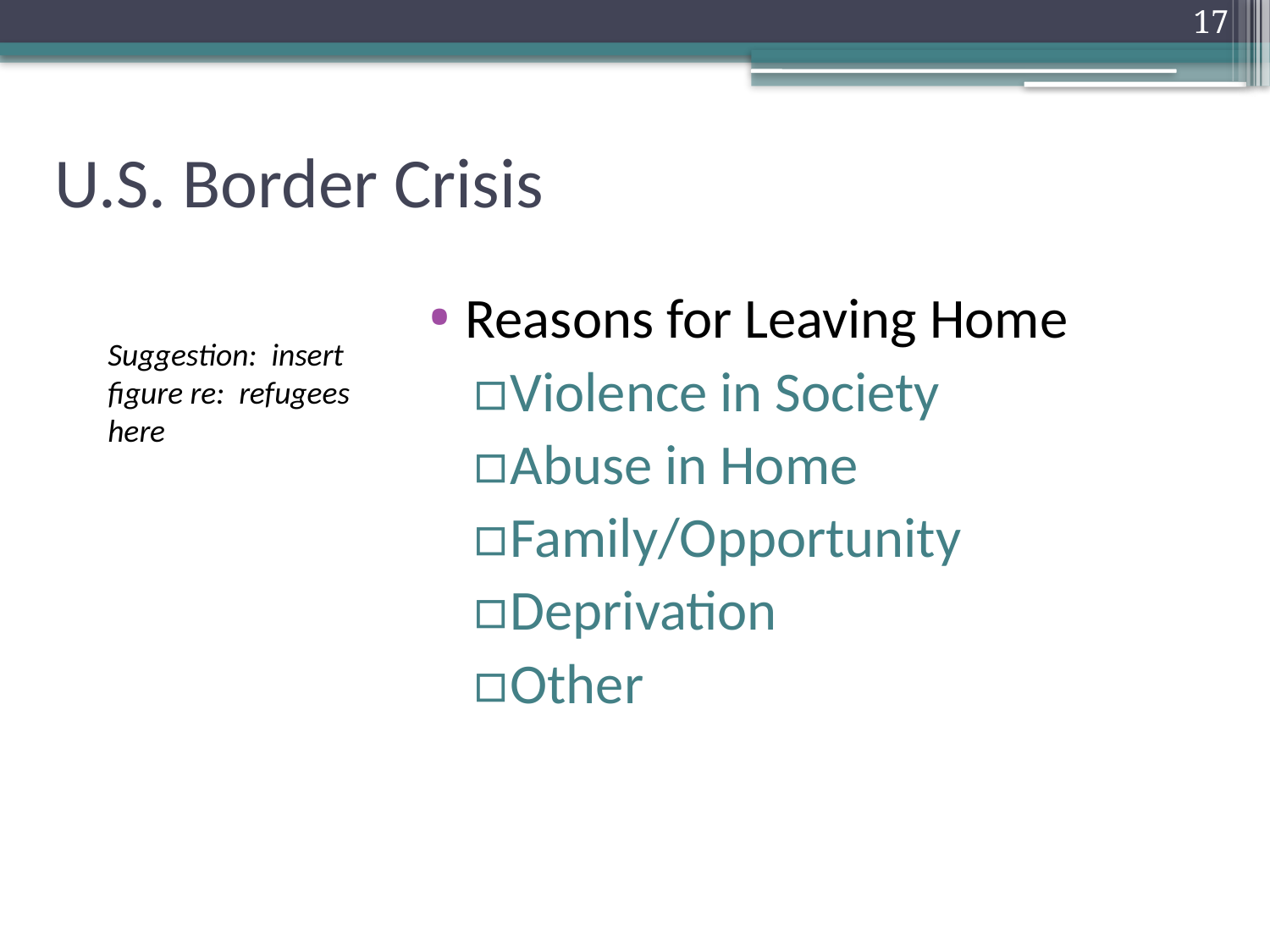

17
# U.S. Border Crisis
Reasons for Leaving Home
Violence in Society
Abuse in Home
Family/Opportunity
Deprivation
Other
Suggestion: insert figure re: refugees here

## Slide 18
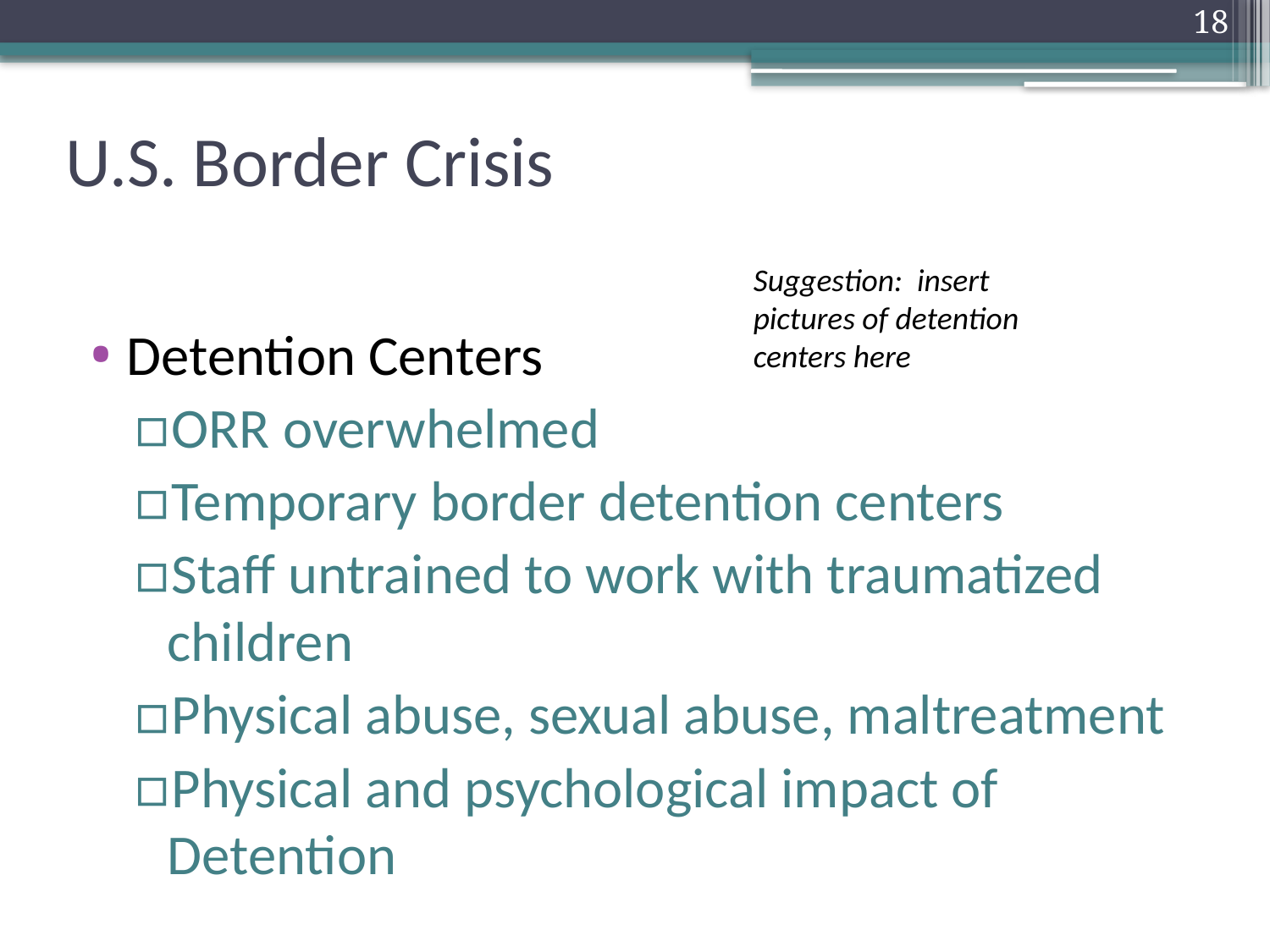

18
# U.S. Border Crisis
Suggestion: insert pictures of detention centers here
Detention Centers
ORR overwhelmed
Temporary border detention centers
Staff untrained to work with traumatized children
Physical abuse, sexual abuse, maltreatment
Physical and psychological impact of Detention

## Slide 19
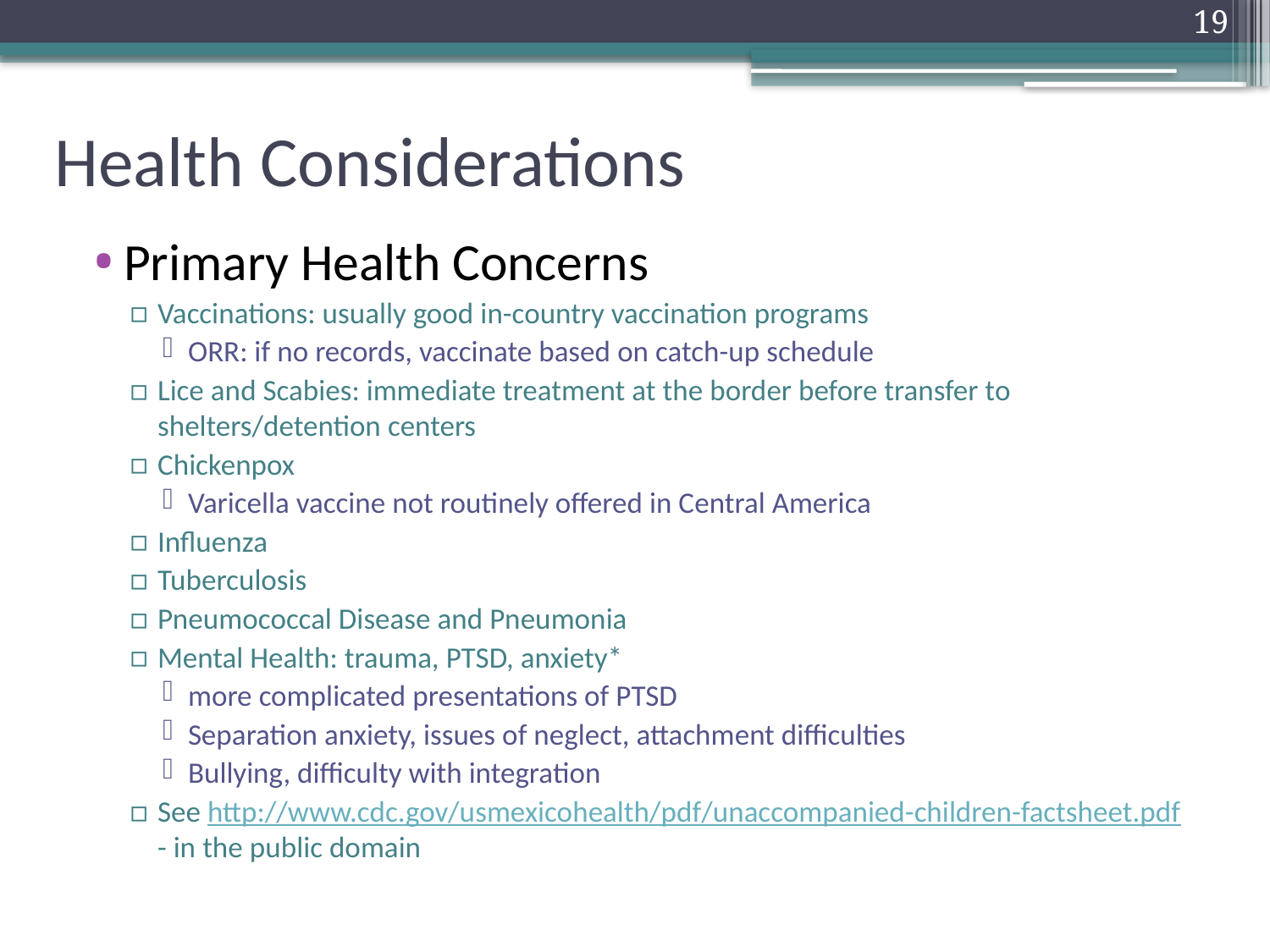

19
# Health Considerations
Primary Health Concerns
Vaccinations: usually good in-country vaccination programs
ORR: if no records, vaccinate based on catch-up schedule
Lice and Scabies: immediate treatment at the border before transfer to shelters/detention centers
Chickenpox
Varicella vaccine not routinely offered in Central America
Influenza
Tuberculosis
Pneumococcal Disease and Pneumonia
Mental Health: trauma, PTSD, anxiety*
more complicated presentations of PTSD
Separation anxiety, issues of neglect, attachment difficulties
Bullying, difficulty with integration
See http://www.cdc.gov/usmexicohealth/pdf/unaccompanied-children-factsheet.pdf - in the public domain

## Slide 20
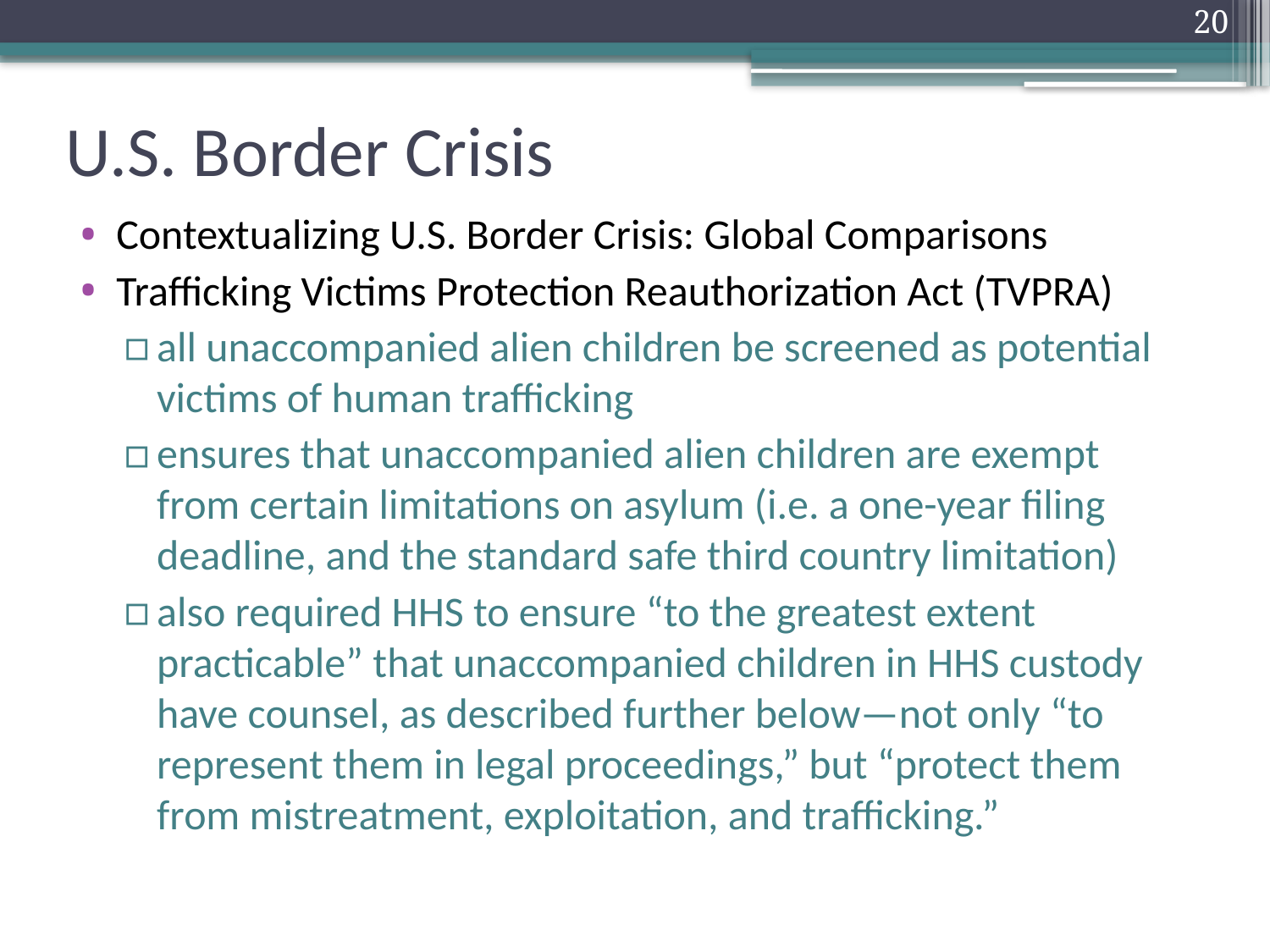

20
# U.S. Border Crisis
Contextualizing U.S. Border Crisis: Global Comparisons
Trafficking Victims Protection Reauthorization Act (TVPRA)
all unaccompanied alien children be screened as potential victims of human trafficking
ensures that unaccompanied alien children are exempt from certain limitations on asylum (i.e. a one-year filing deadline, and the standard safe third country limitation)
also required HHS to ensure “to the greatest extent practicable” that unaccompanied children in HHS custody have counsel, as described further below—not only “to represent them in legal proceedings,” but “protect them from mistreatment, exploitation, and trafficking.”

## Slide 21
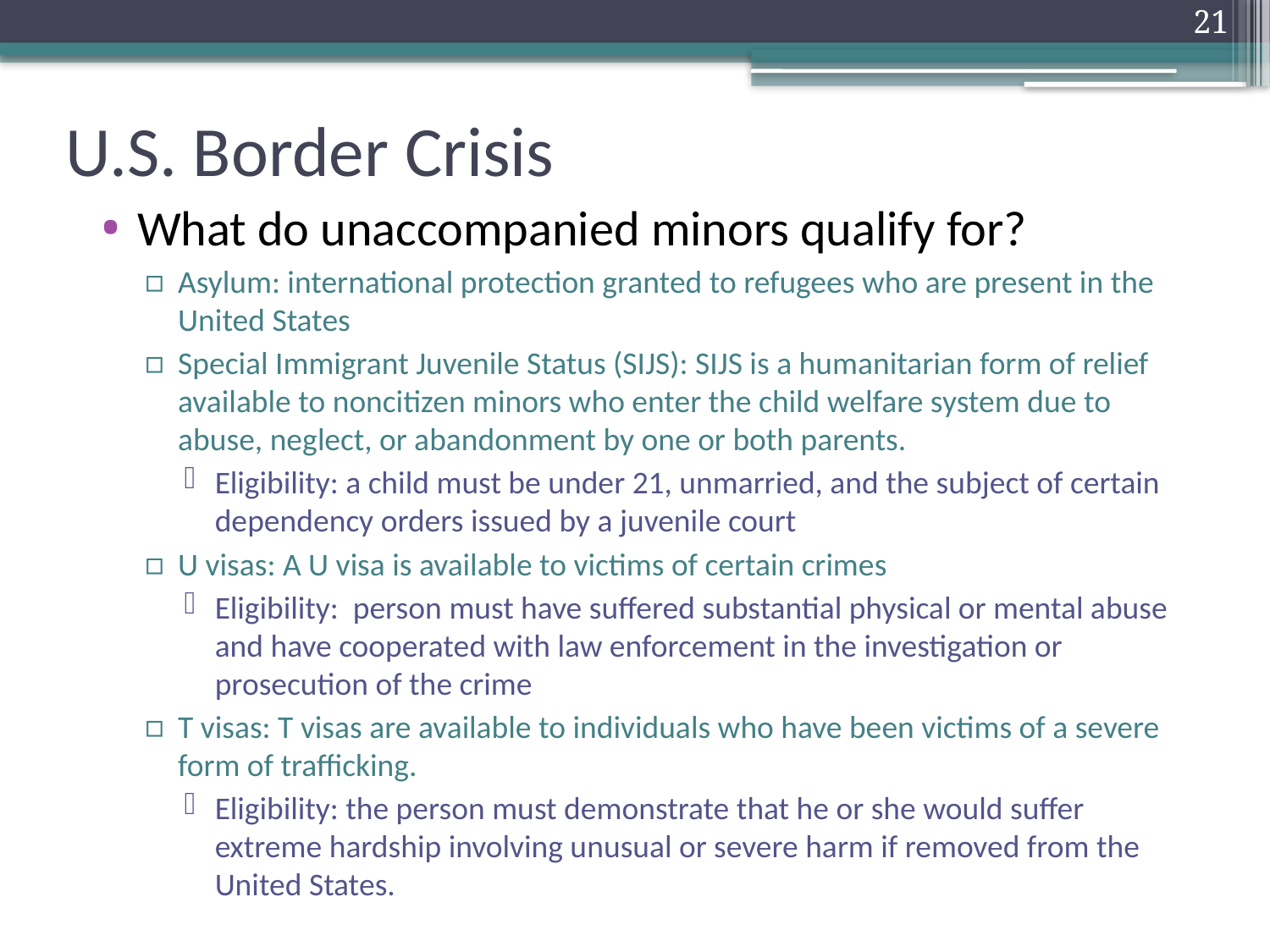

21
# U.S. Border Crisis
What do unaccompanied minors qualify for?
Asylum: international protection granted to refugees who are present in the United States
Special Immigrant Juvenile Status (SIJS): SIJS is a humanitarian form of relief available to noncitizen minors who enter the child welfare system due to abuse, neglect, or abandonment by one or both parents.
Eligibility: a child must be under 21, unmarried, and the subject of certain dependency orders issued by a juvenile court
U visas: A U visa is available to victims of certain crimes
Eligibility: person must have suffered substantial physical or mental abuse and have cooperated with law enforcement in the investigation or prosecution of the crime
T visas: T visas are available to individuals who have been victims of a severe form of trafficking.
Eligibility: the person must demonstrate that he or she would suffer extreme hardship involving unusual or severe harm if removed from the United States.

## Slide 22
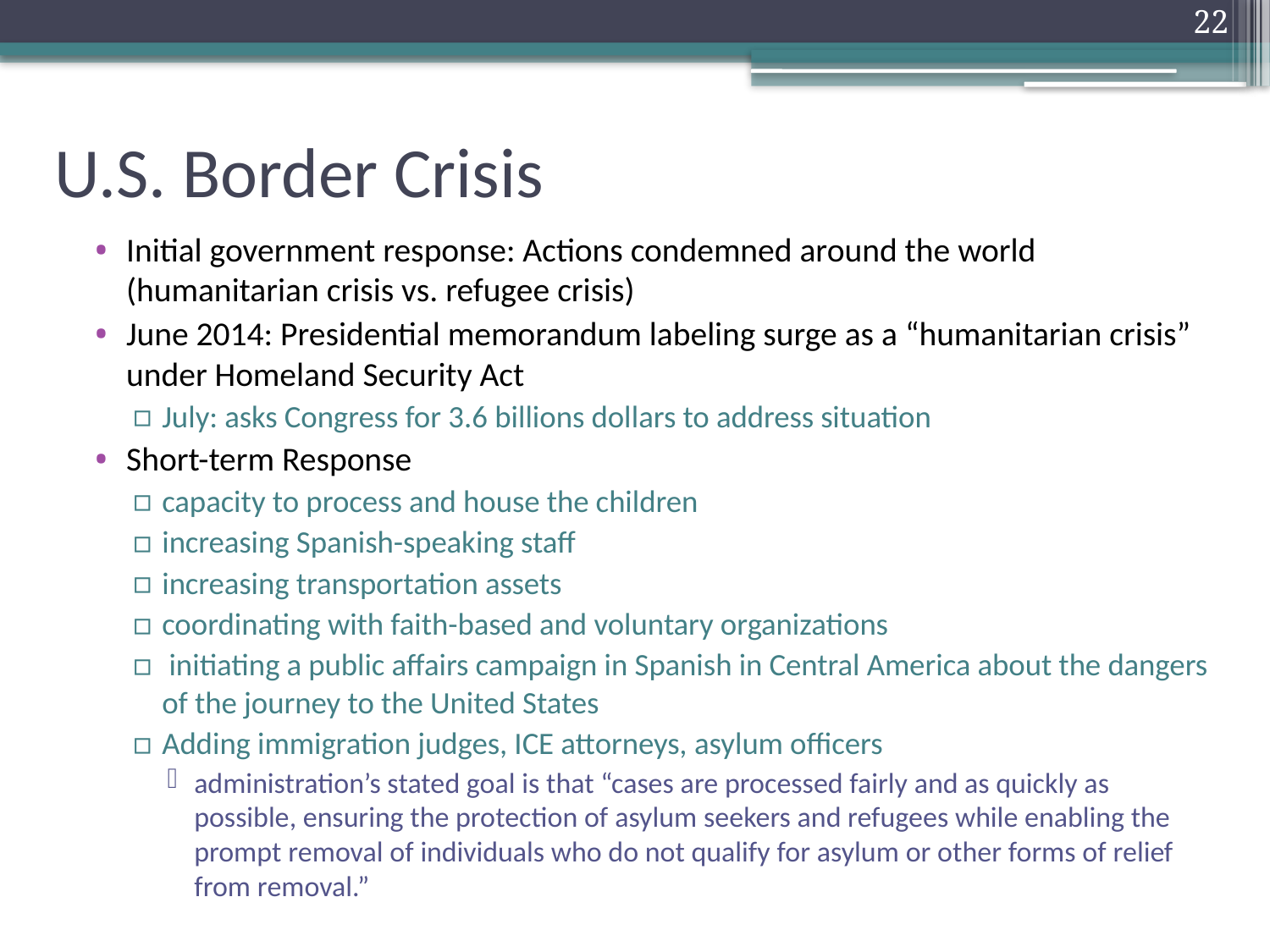

22
# U.S. Border Crisis
Initial government response: Actions condemned around the world (humanitarian crisis vs. refugee crisis)
June 2014: Presidential memorandum labeling surge as a “humanitarian crisis” under Homeland Security Act
July: asks Congress for 3.6 billions dollars to address situation
Short-term Response
capacity to process and house the children
increasing Spanish-speaking staff
increasing transportation assets
coordinating with faith-based and voluntary organizations
 initiating a public affairs campaign in Spanish in Central America about the dangers of the journey to the United States
Adding immigration judges, ICE attorneys, asylum officers
administration’s stated goal is that “cases are processed fairly and as quickly as possible, ensuring the protection of asylum seekers and refugees while enabling the prompt removal of individuals who do not qualify for asylum or other forms of relief from removal.”

## Slide 23
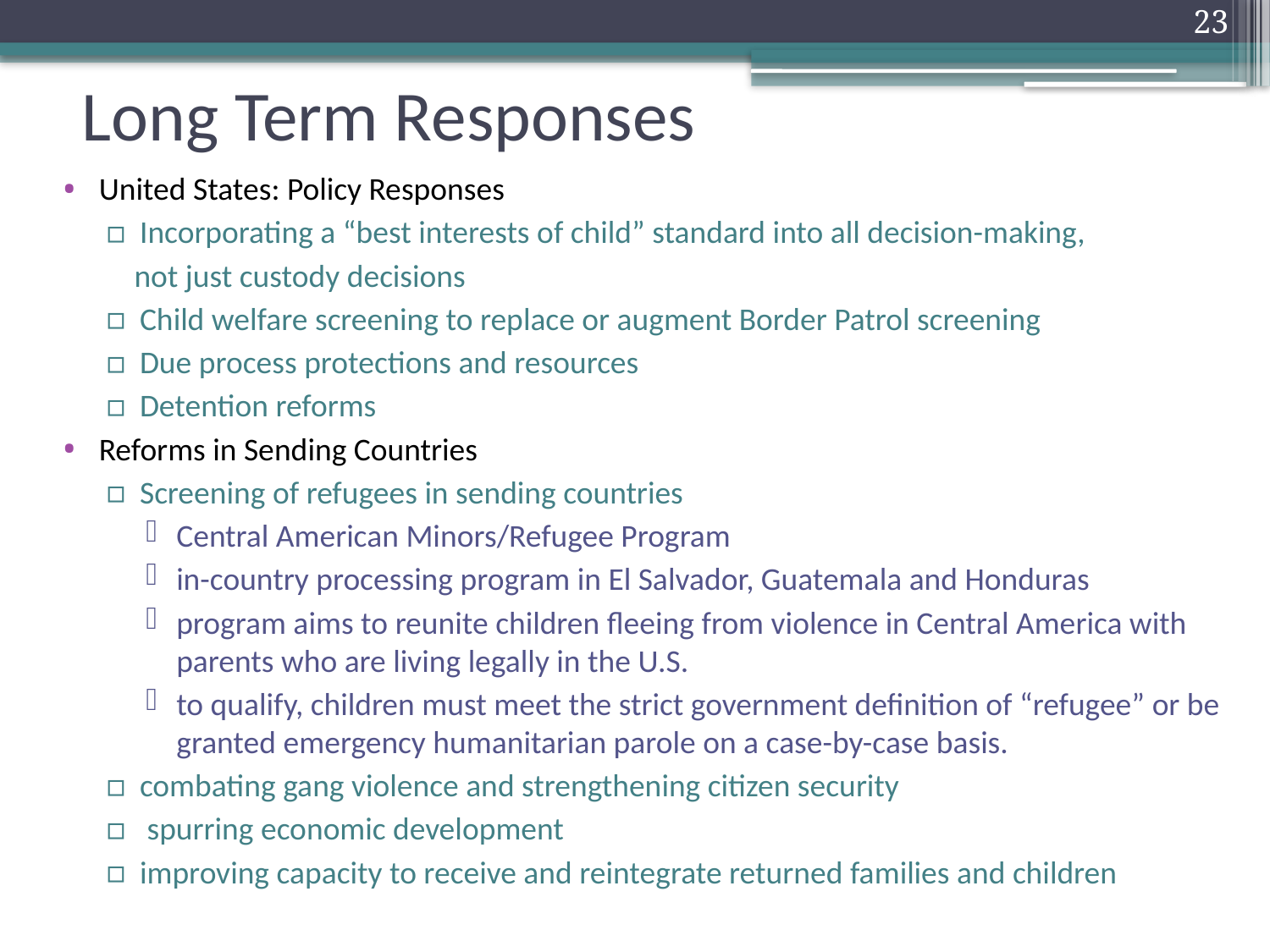

23
# Long Term Responses
United States: Policy Responses
Incorporating a “best interests of child” standard into all decision-making,
 not just custody decisions
Child welfare screening to replace or augment Border Patrol screening
Due process protections and resources
Detention reforms
Reforms in Sending Countries
Screening of refugees in sending countries
Central American Minors/Refugee Program
in-country processing program in El Salvador, Guatemala and Honduras
program aims to reunite children fleeing from violence in Central America with parents who are living legally in the U.S.
to qualify, children must meet the strict government definition of “refugee” or be granted emergency humanitarian parole on a case-by-case basis.
combating gang violence and strengthening citizen security
 spurring economic development
improving capacity to receive and reintegrate returned families and children

## Slide 24
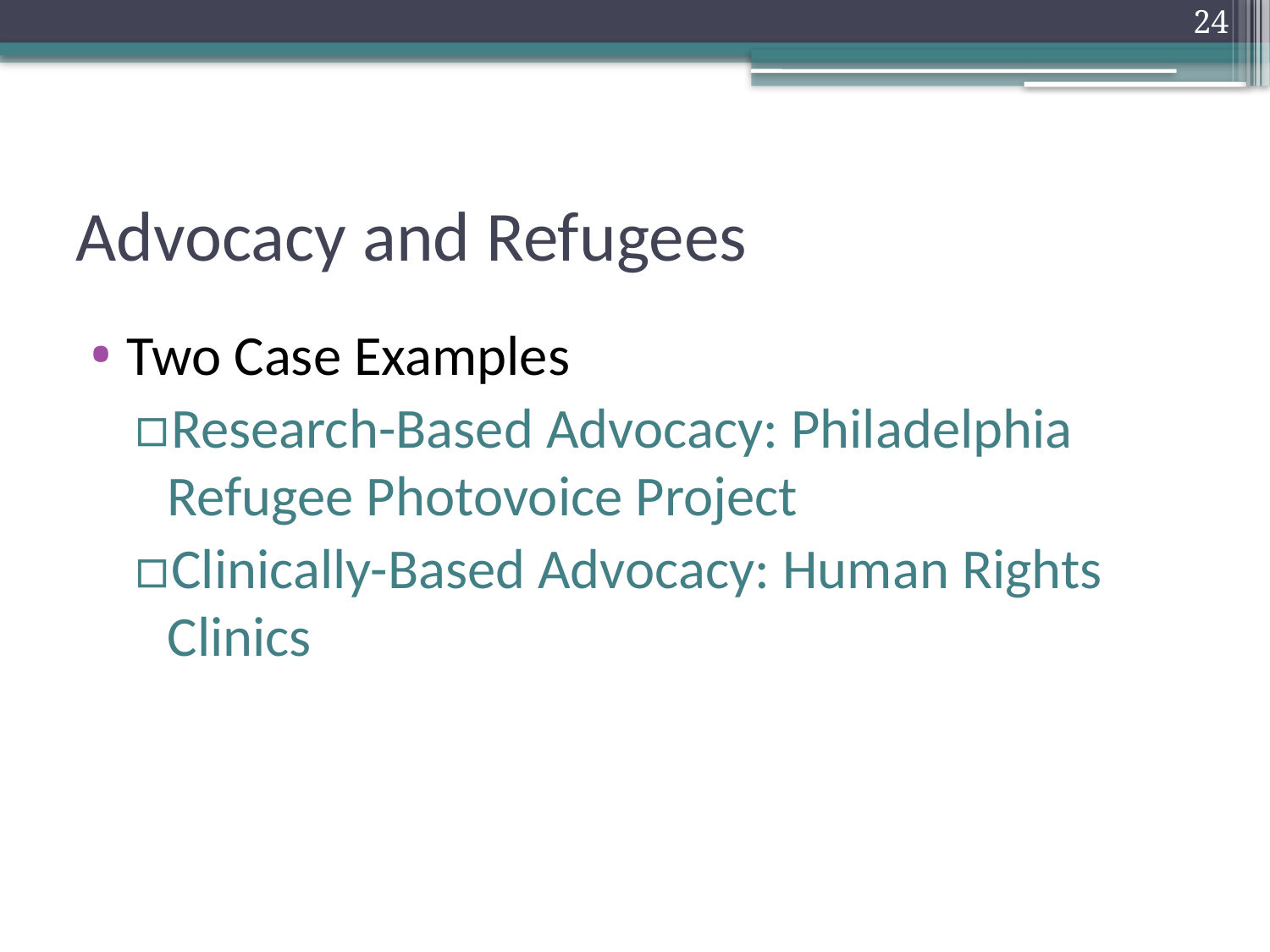

24
# Advocacy and Refugees
Two Case Examples
Research-Based Advocacy: Philadelphia Refugee Photovoice Project
Clinically-Based Advocacy: Human Rights Clinics

## Slide 25
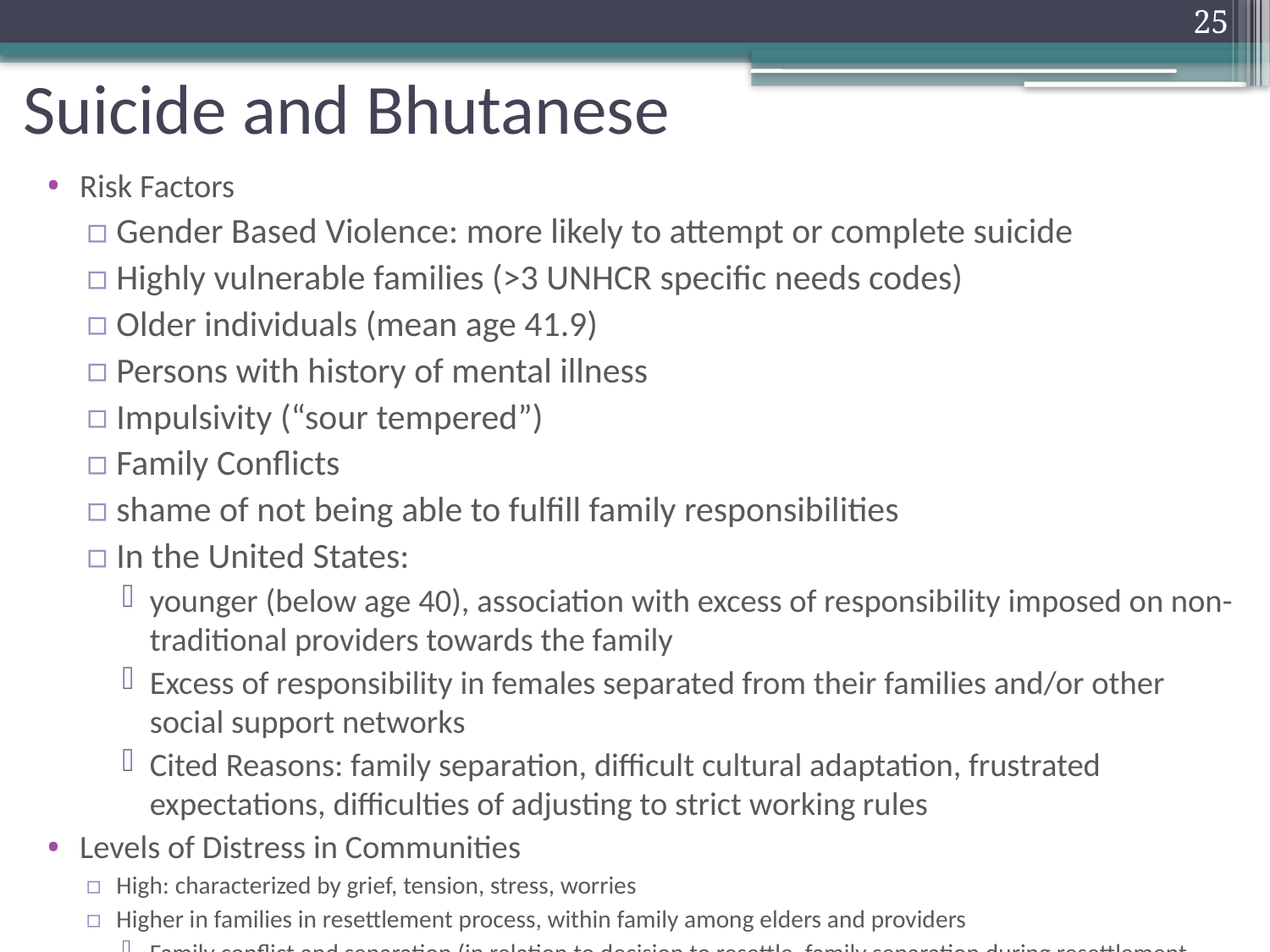

25
# Suicide and Bhutanese
Risk Factors
Gender Based Violence: more likely to attempt or complete suicide
Highly vulnerable families (>3 UNHCR specific needs codes)
Older individuals (mean age 41.9)
Persons with history of mental illness
Impulsivity (“sour tempered”)
Family Conflicts
shame of not being able to fulfill family responsibilities
In the United States:
younger (below age 40), association with excess of responsibility imposed on non-traditional providers towards the family
Excess of responsibility in females separated from their families and/or other social support networks
Cited Reasons: family separation, difficult cultural adaptation, frustrated expectations, difficulties of adjusting to strict working rules
Levels of Distress in Communities
High: characterized by grief, tension, stress, worries
Higher in families in resettlement process, within family among elders and providers
Family conflict and separation (in relation to decision to resettle, family separation during resettlement, maternal-paternal family issues)

## Slide 26
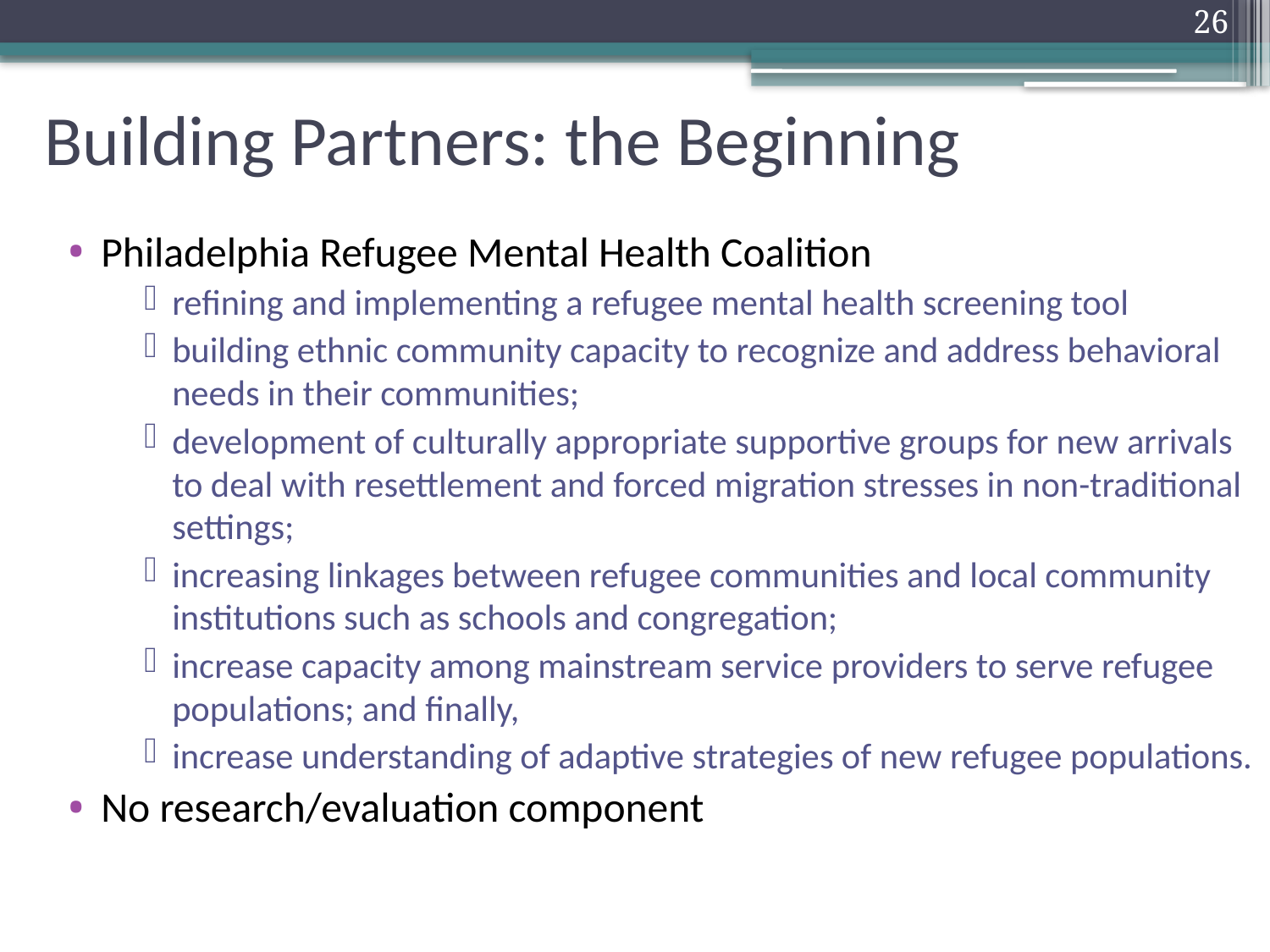

26
# Building Partners: the Beginning
Philadelphia Refugee Mental Health Coalition
refining and implementing a refugee mental health screening tool
building ethnic community capacity to recognize and address behavioral needs in their communities;
development of culturally appropriate supportive groups for new arrivals to deal with resettlement and forced migration stresses in non-traditional settings;
increasing linkages between refugee communities and local community institutions such as schools and congregation;
increase capacity among mainstream service providers to serve refugee populations; and finally,
increase understanding of adaptive strategies of new refugee populations.
No research/evaluation component

## Slide 27
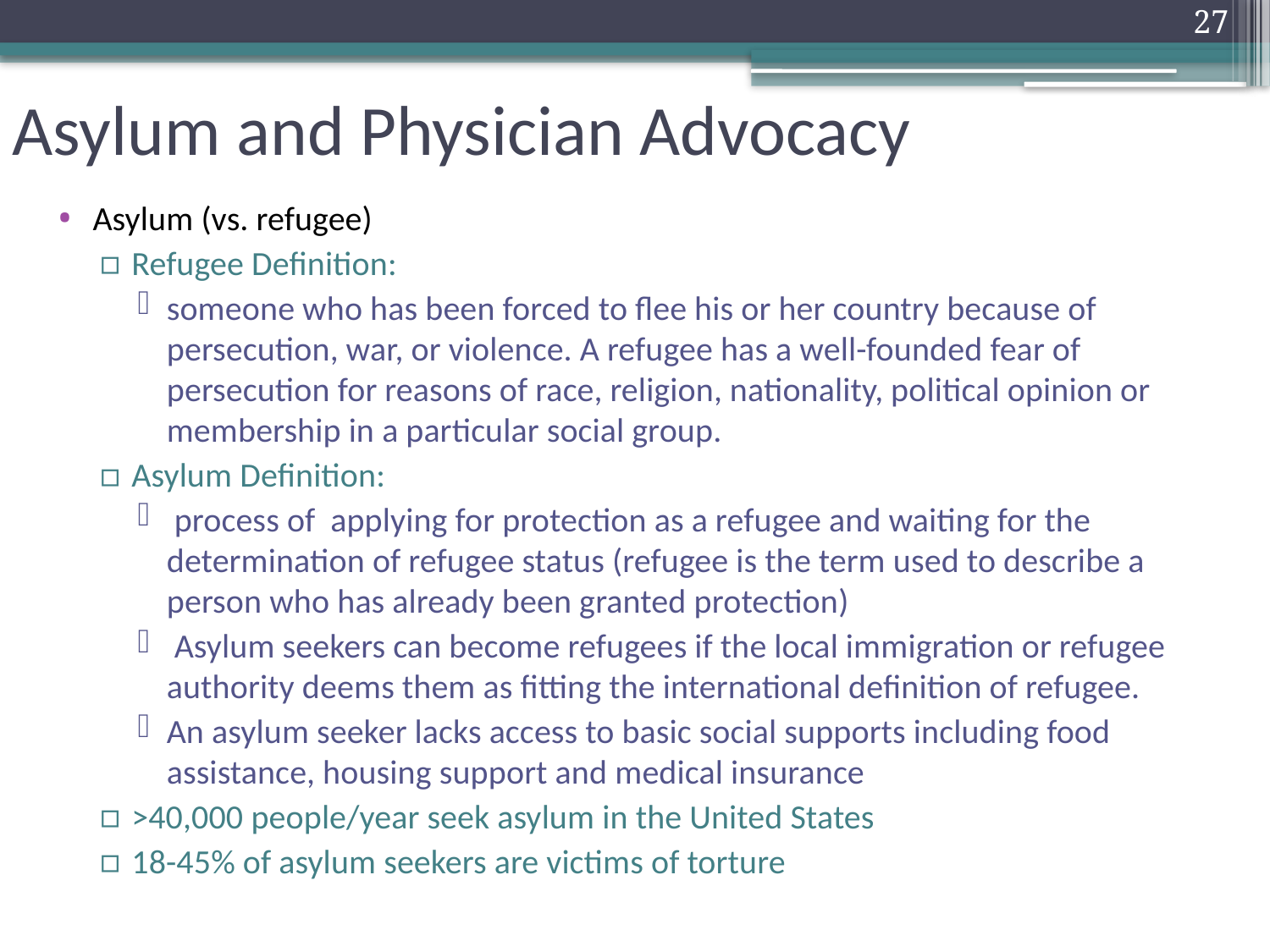

27
# Asylum and Physician Advocacy
Asylum (vs. refugee)
Refugee Definition:
someone who has been forced to flee his or her country because of persecution, war, or violence. A refugee has a well-founded fear of persecution for reasons of race, religion, nationality, political opinion or membership in a particular social group.
Asylum Definition:
 process of applying for protection as a refugee and waiting for the determination of refugee status (refugee is the term used to describe a person who has already been granted protection)
 Asylum seekers can become refugees if the local immigration or refugee authority deems them as fitting the international definition of refugee.
An asylum seeker lacks access to basic social supports including food assistance, housing support and medical insurance
>40,000 people/year seek asylum in the United States
18-45% of asylum seekers are victims of torture

## Slide 28
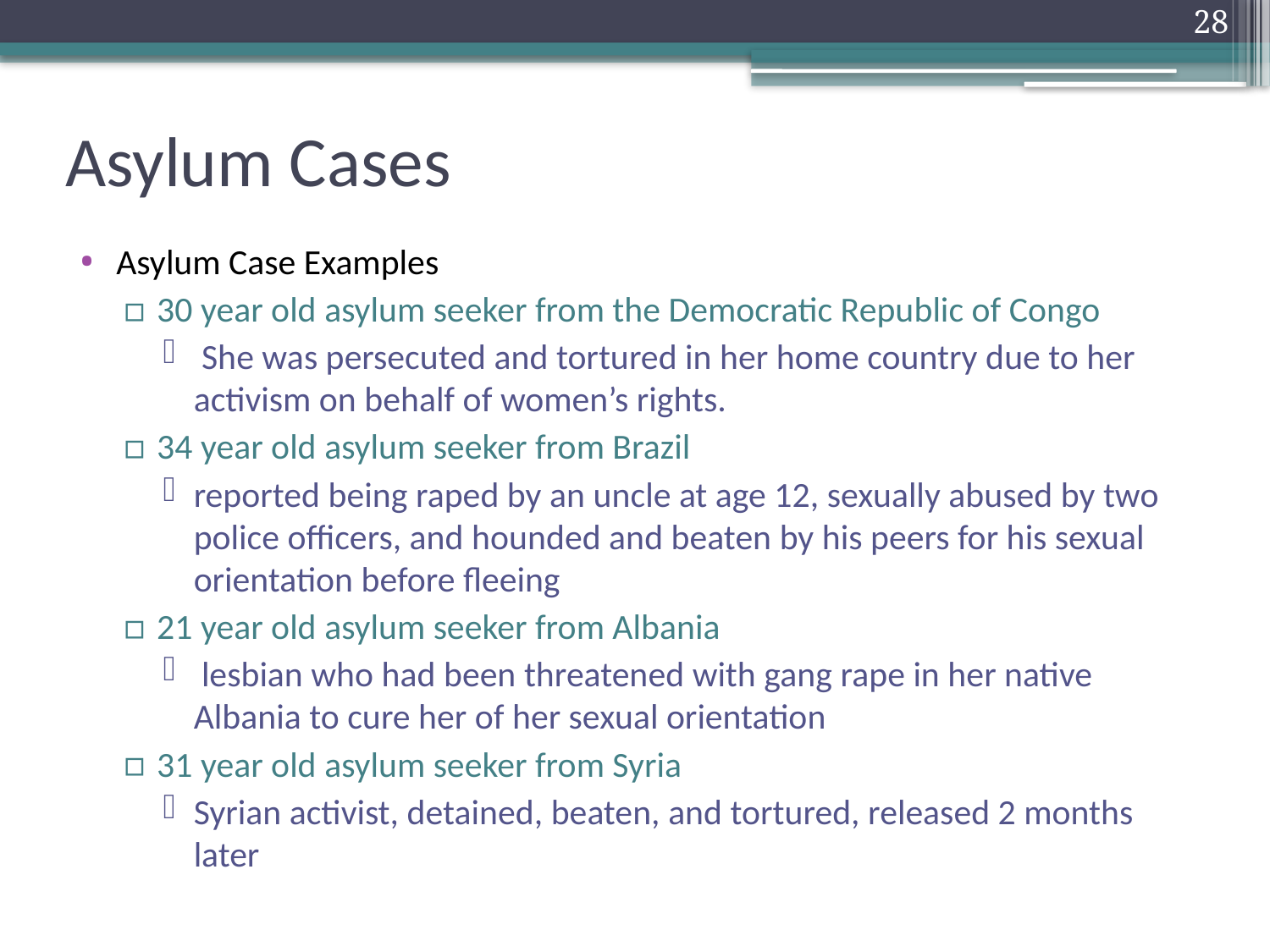

28
# Asylum Cases
Asylum Case Examples
30 year old asylum seeker from the Democratic Republic of Congo
 She was persecuted and tortured in her home country due to her activism on behalf of women’s rights.
34 year old asylum seeker from Brazil
reported being raped by an uncle at age 12, sexually abused by two police officers, and hounded and beaten by his peers for his sexual orientation before fleeing
21 year old asylum seeker from Albania
 lesbian who had been threatened with gang rape in her native Albania to cure her of her sexual orientation
31 year old asylum seeker from Syria
Syrian activist, detained, beaten, and tortured, released 2 months later

## Slide 29
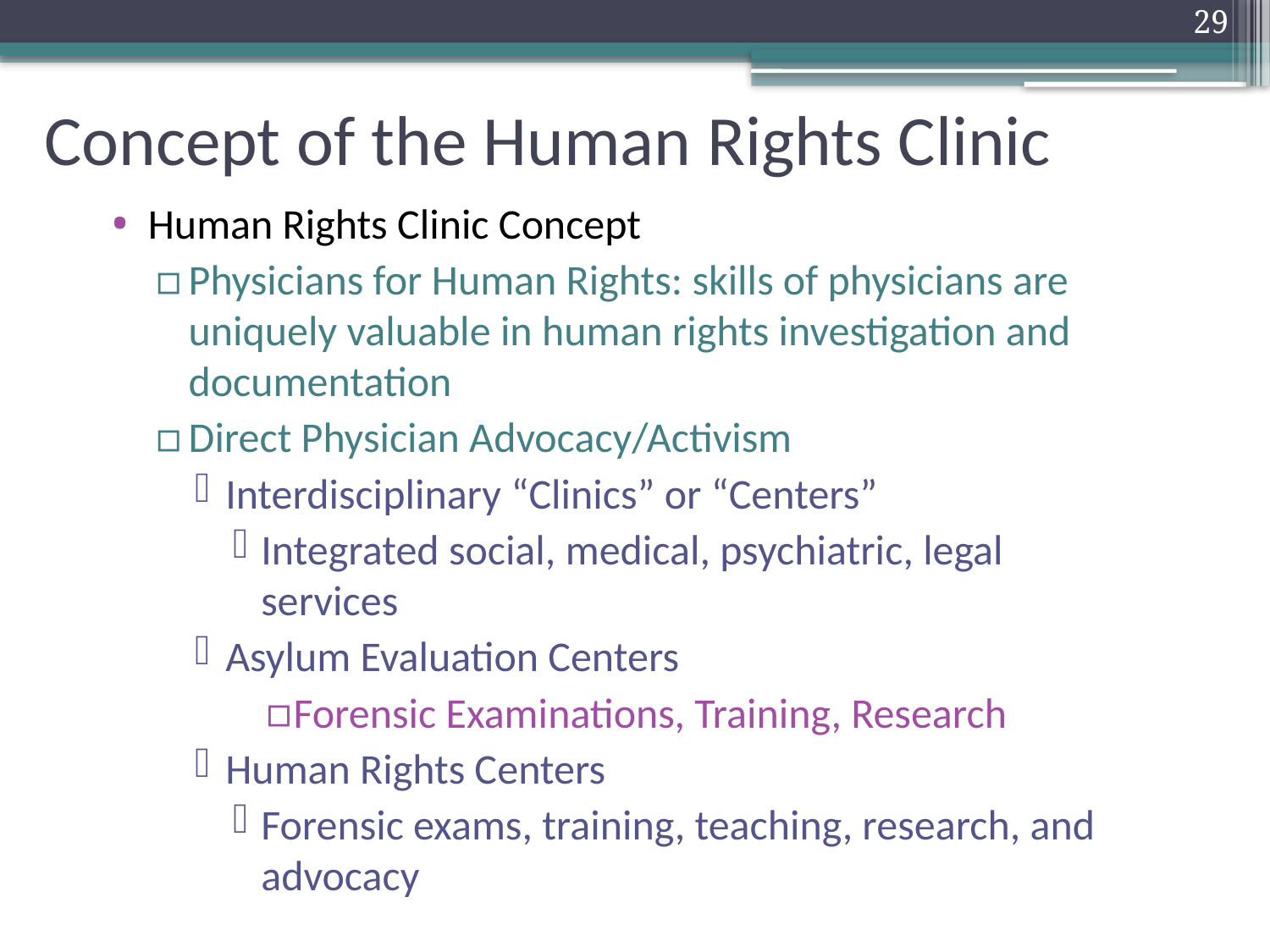

29
# Concept of the Human Rights Clinic
Human Rights Clinic Concept
Physicians for Human Rights: skills of physicians are uniquely valuable in human rights investigation and documentation
Direct Physician Advocacy/Activism
Interdisciplinary “Clinics” or “Centers”
Integrated social, medical, psychiatric, legal services
Asylum Evaluation Centers
Forensic Examinations, Training, Research
Human Rights Centers
Forensic exams, training, teaching, research, and advocacy

## Slide 30
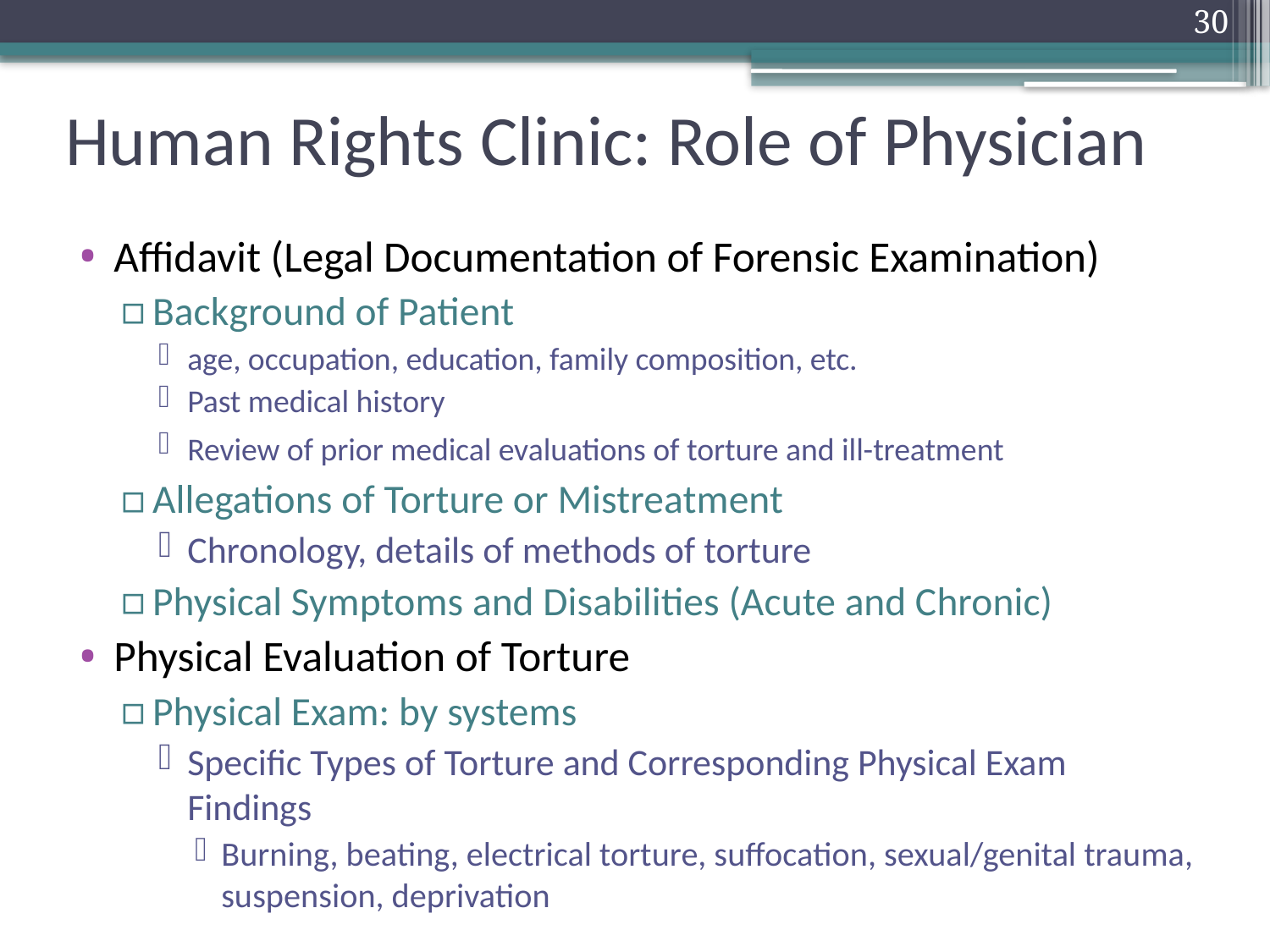

30
# Human Rights Clinic: Role of Physician
Affidavit (Legal Documentation of Forensic Examination)
Background of Patient
age, occupation, education, family composition, etc.
Past medical history
Review of prior medical evaluations of torture and ill-treatment
Allegations of Torture or Mistreatment
Chronology, details of methods of torture
Physical Symptoms and Disabilities (Acute and Chronic)
Physical Evaluation of Torture
Physical Exam: by systems
Specific Types of Torture and Corresponding Physical Exam Findings
Burning, beating, electrical torture, suffocation, sexual/genital trauma, suspension, deprivation

## Slide 31
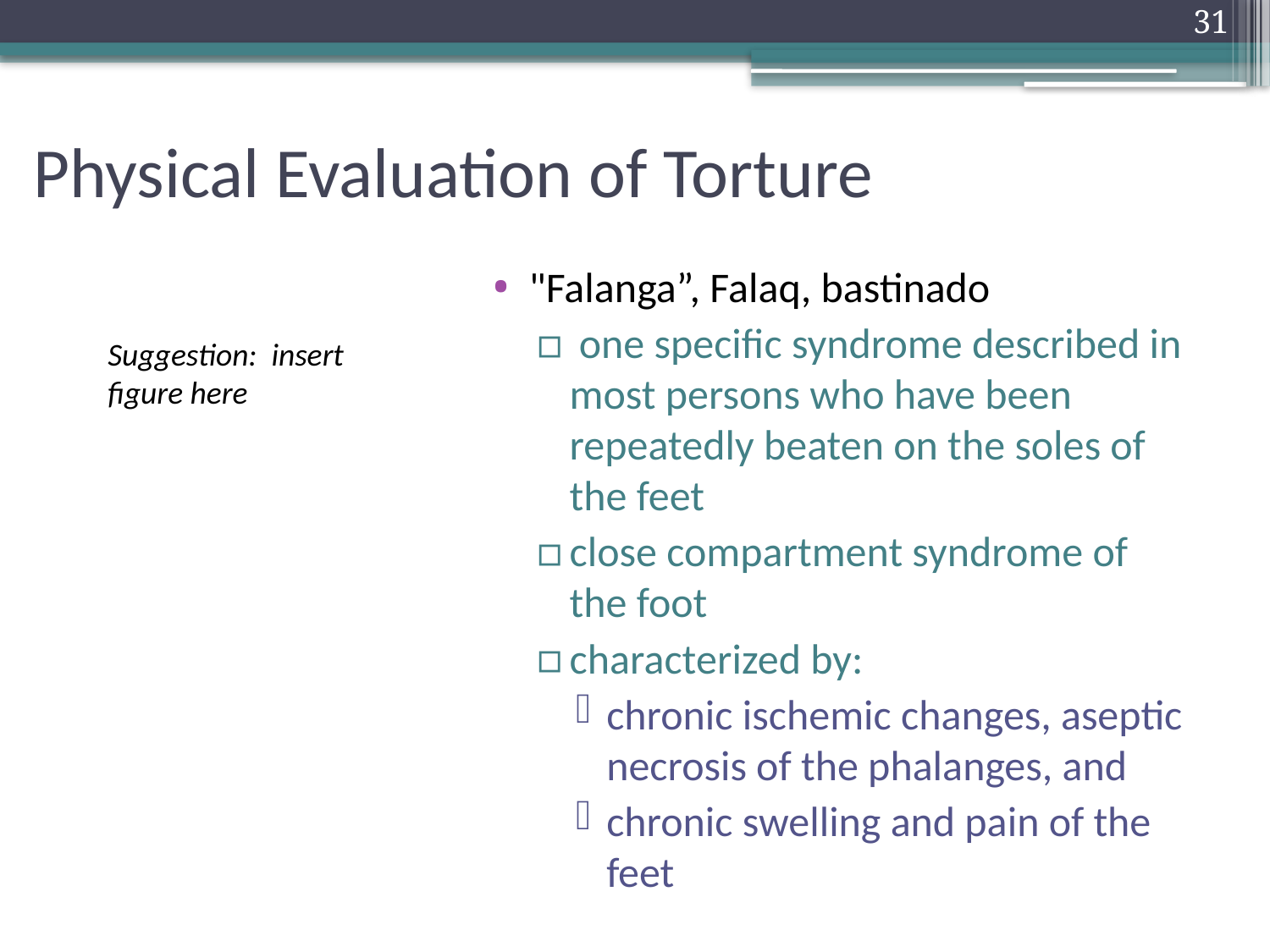

31
# Physical Evaluation of Torture
"Falanga”, Falaq, bastinado
 one specific syndrome described in most persons who have been repeatedly beaten on the soles of the feet
close compartment syndrome of the foot
characterized by:
chronic ischemic changes, aseptic necrosis of the phalanges, and
chronic swelling and pain of the feet
Suggestion: insert figure here

## Slide 32
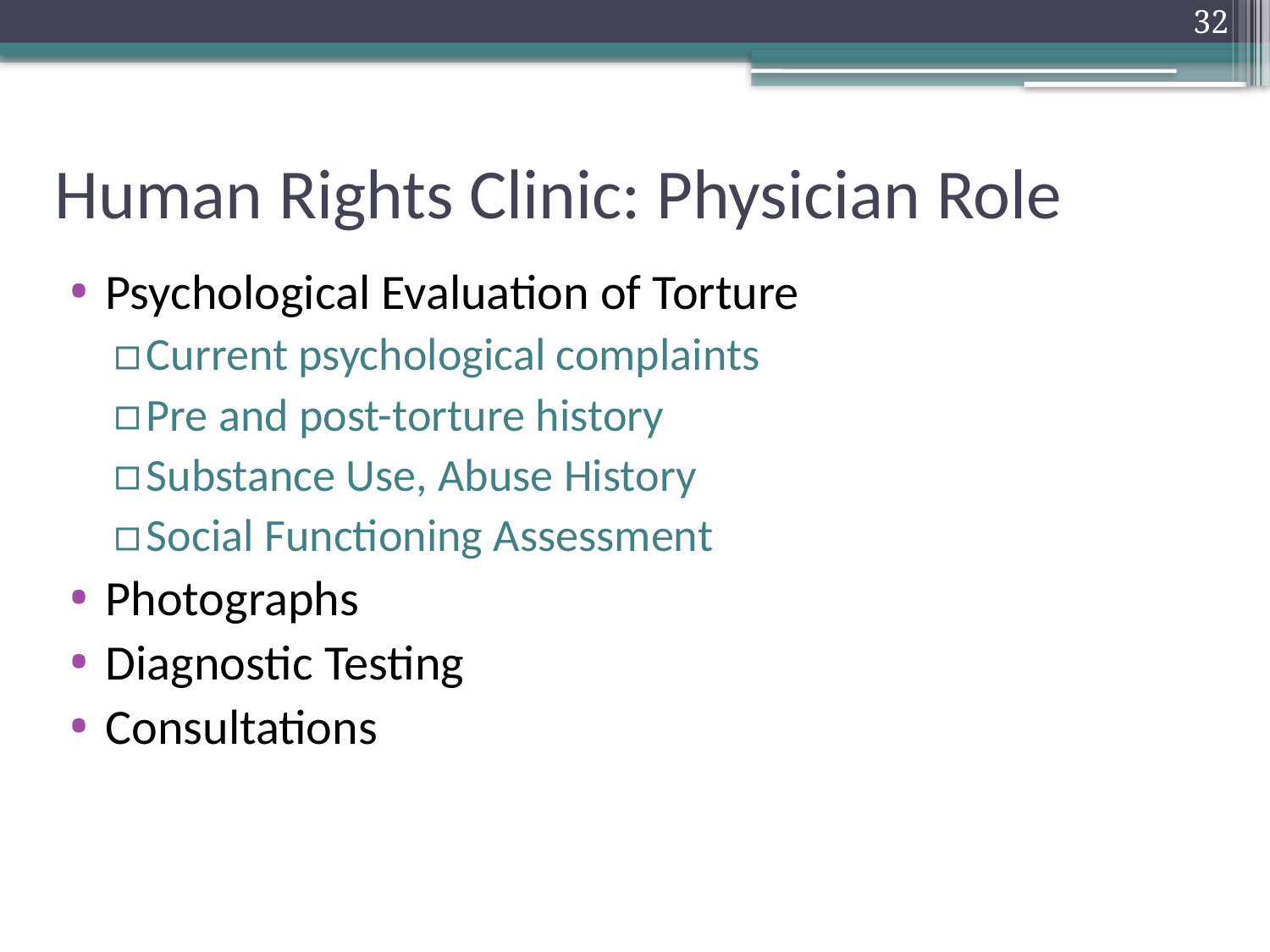

32
# Human Rights Clinic: Physician Role
Psychological Evaluation of Torture
Current psychological complaints
Pre and post-torture history
Substance Use, Abuse History
Social Functioning Assessment
Photographs
Diagnostic Testing
Consultations

## Slide 33
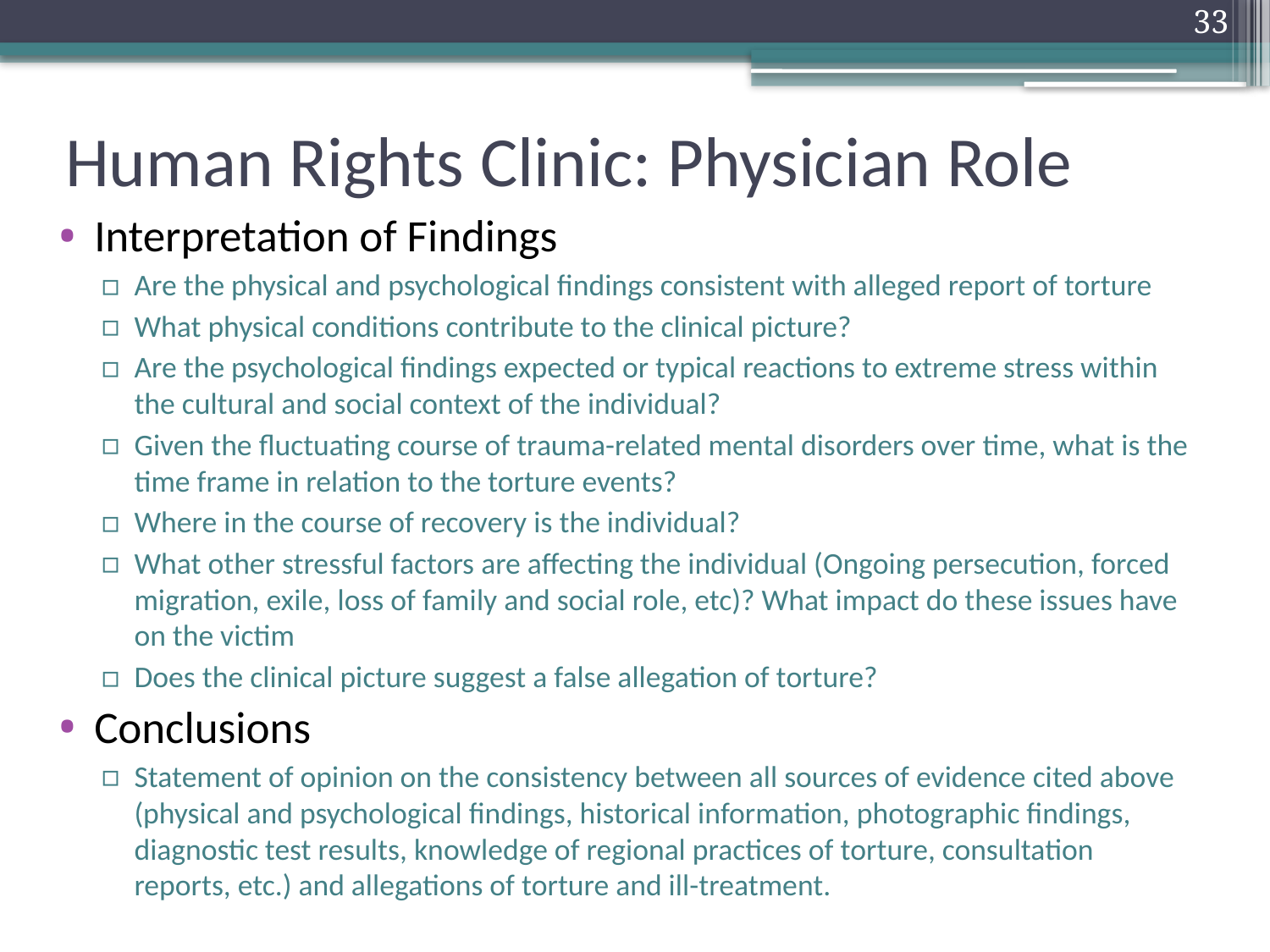

33
# Human Rights Clinic: Physician Role
Interpretation of Findings
Are the physical and psychological findings consistent with alleged report of torture
What physical conditions contribute to the clinical picture?
Are the psychological findings expected or typical reactions to extreme stress within the cultural and social context of the individual?
Given the fluctuating course of trauma-related mental disorders over time, what is the time frame in relation to the torture events?
Where in the course of recovery is the individual?
What other stressful factors are affecting the individual (Ongoing persecution, forced migration, exile, loss of family and social role, etc)? What impact do these issues have on the victim
Does the clinical picture suggest a false allegation of torture?
Conclusions
Statement of opinion on the consistency between all sources of evidence cited above (physical and psychological findings, historical information, photographic findings, diagnostic test results, knowledge of regional practices of torture, consultation reports, etc.) and allegations of torture and ill-treatment.

## Slide 34
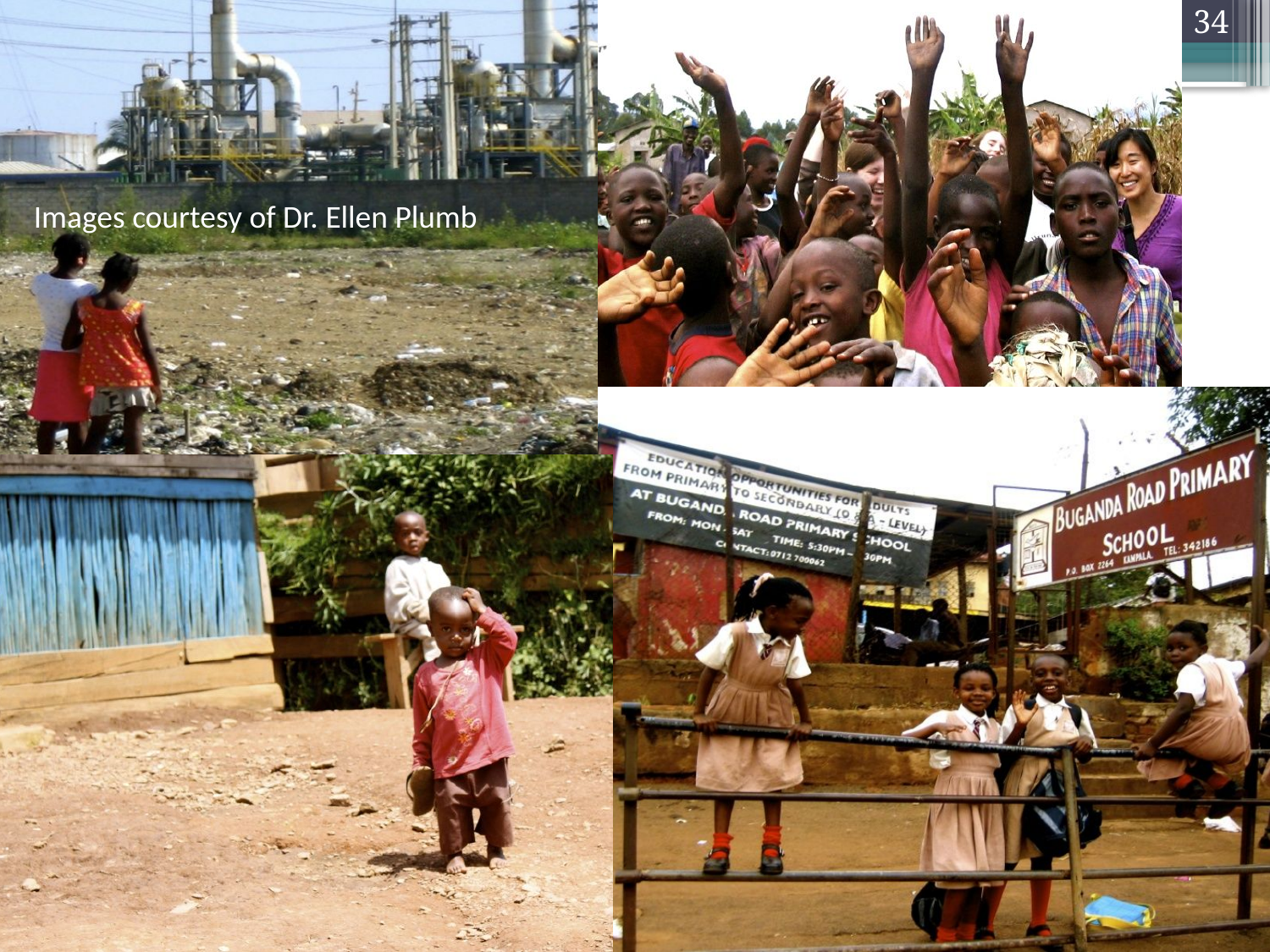

34
#
Images courtesy of Dr. Ellen Plumb
