# Supplementary material for: The JeffSTARS Advocacy and Community Partnership Elective: A Closer Look at Child Health Advocacy in Action
Source: MedEdPORTAL. 2016 Dec 31;12:10526. doi: 10.15766/mep_2374-8265.10526 (PMC6365684; doi:10.15766/mep_2374-8265.10526)
Supplement: Supplementary file 1 — A. CM1. Course Implementation at New Institution Checklist.docx B. CM2. Elective Checklist.docx C. CM3. Sample Schedule.docx D. CM4. Seminar Topic List With Learning Objectives.docx E. CM5. Syllabus Bibliography.docx F. CM6. List of Community Partners.docx G. CM7. Orientation for New Community Partner.docx H. CM8. Selected Past Projects.docx I. CM9. Sample Fact Sheets for Legislative Visits.docx J. Seminar Materials folder K. ET1. Advocacy Elective Assessment 1.pdf L. ET2. Advocacy Elective Assessment 2.pdf M. ET3. Trainee Evaluation by Community or Faculty Mentor.docx N. ET4. Trainee Evaluation of Seminar.docx O. ET5. Trainee Evaluation of Community Partner.docx P. ET6. Final Report Template.docx Q. Selected Trainee Abstracts and Presented Results folder [file mep-12-10526-s001.zip › J._Seminar_Materials_folder/3._Social_Factors.pptx]

## Slide 1
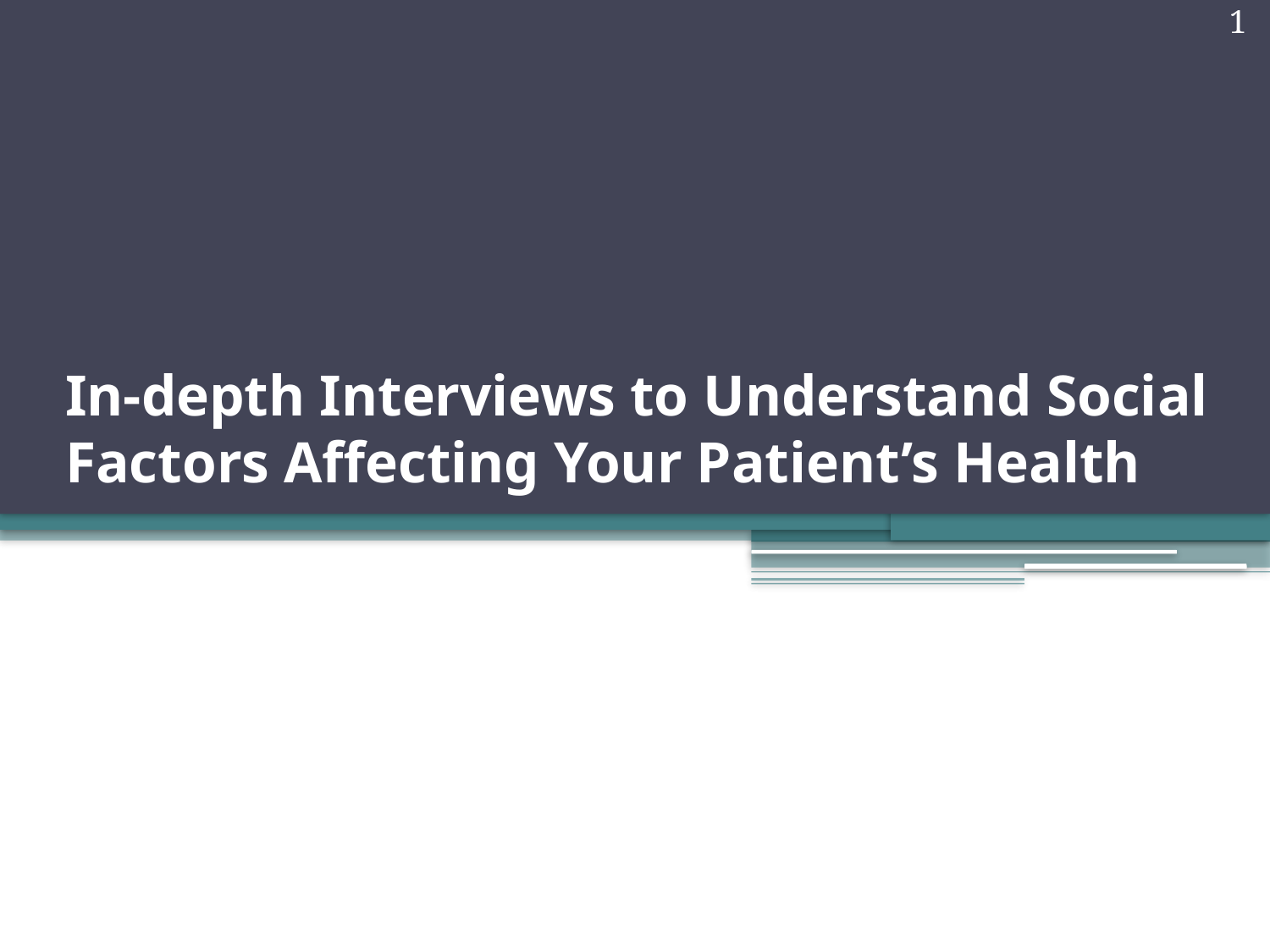

1
# In-depth Interviews to Understand Social Factors Affecting Your Patient’s Health

## Slide 2
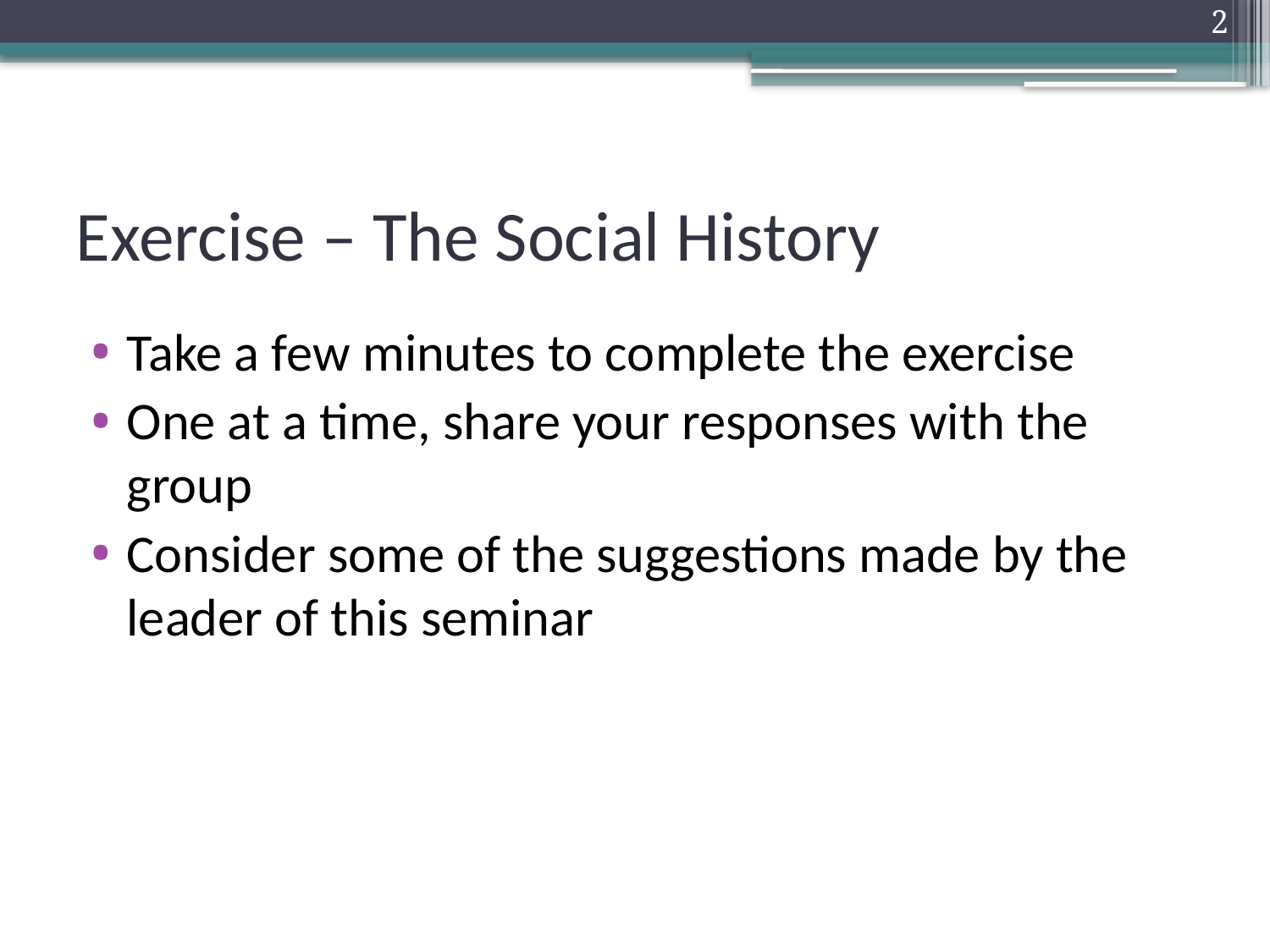

2
# Exercise – The Social History
Take a few minutes to complete the exercise
One at a time, share your responses with the group
Consider some of the suggestions made by the leader of this seminar

## Slide 3
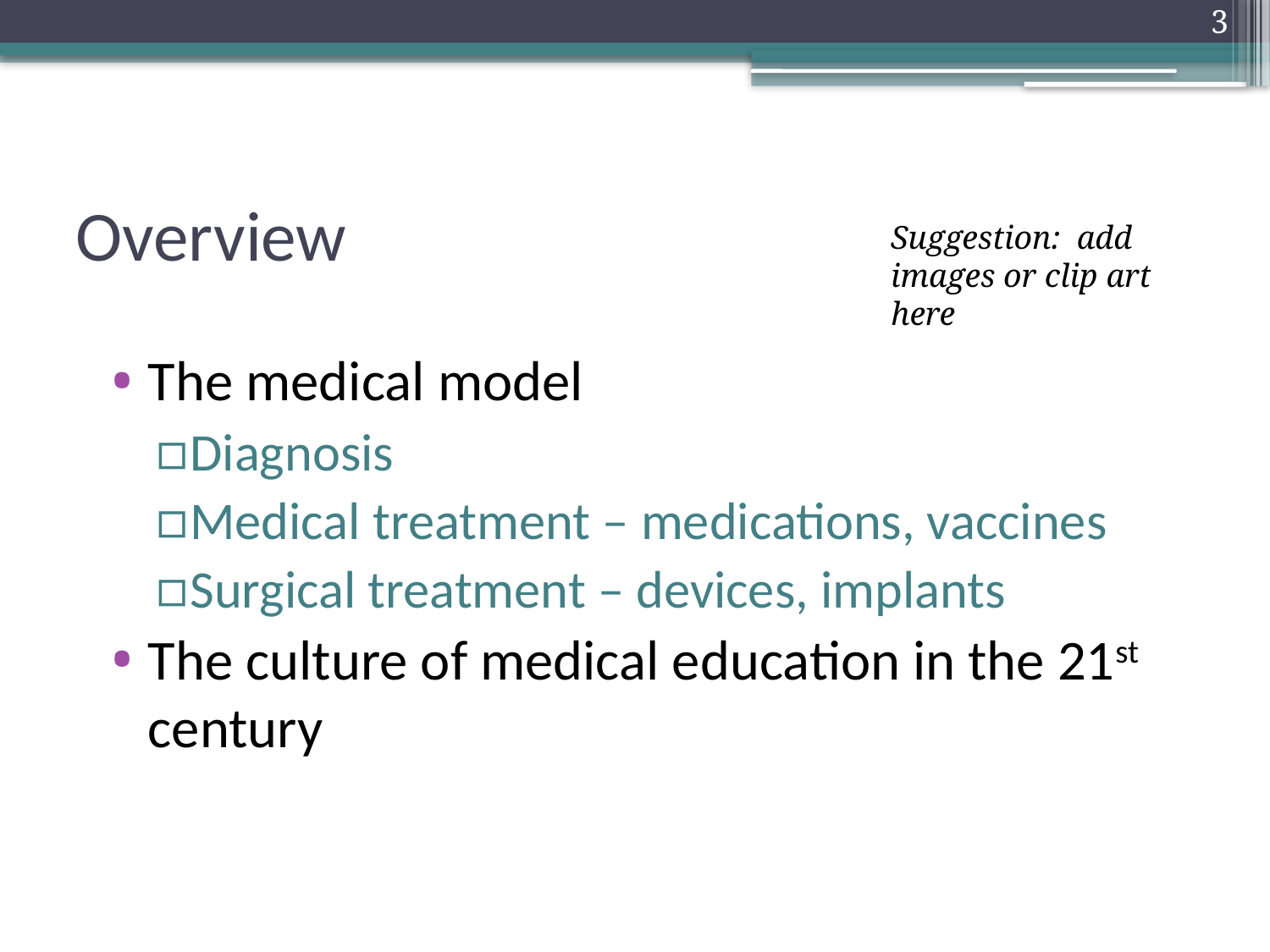

3
# Overview
Suggestion: add images or clip art here
The medical model
Diagnosis
Medical treatment – medications, vaccines
Surgical treatment – devices, implants
The culture of medical education in the 21st century

## Slide 4
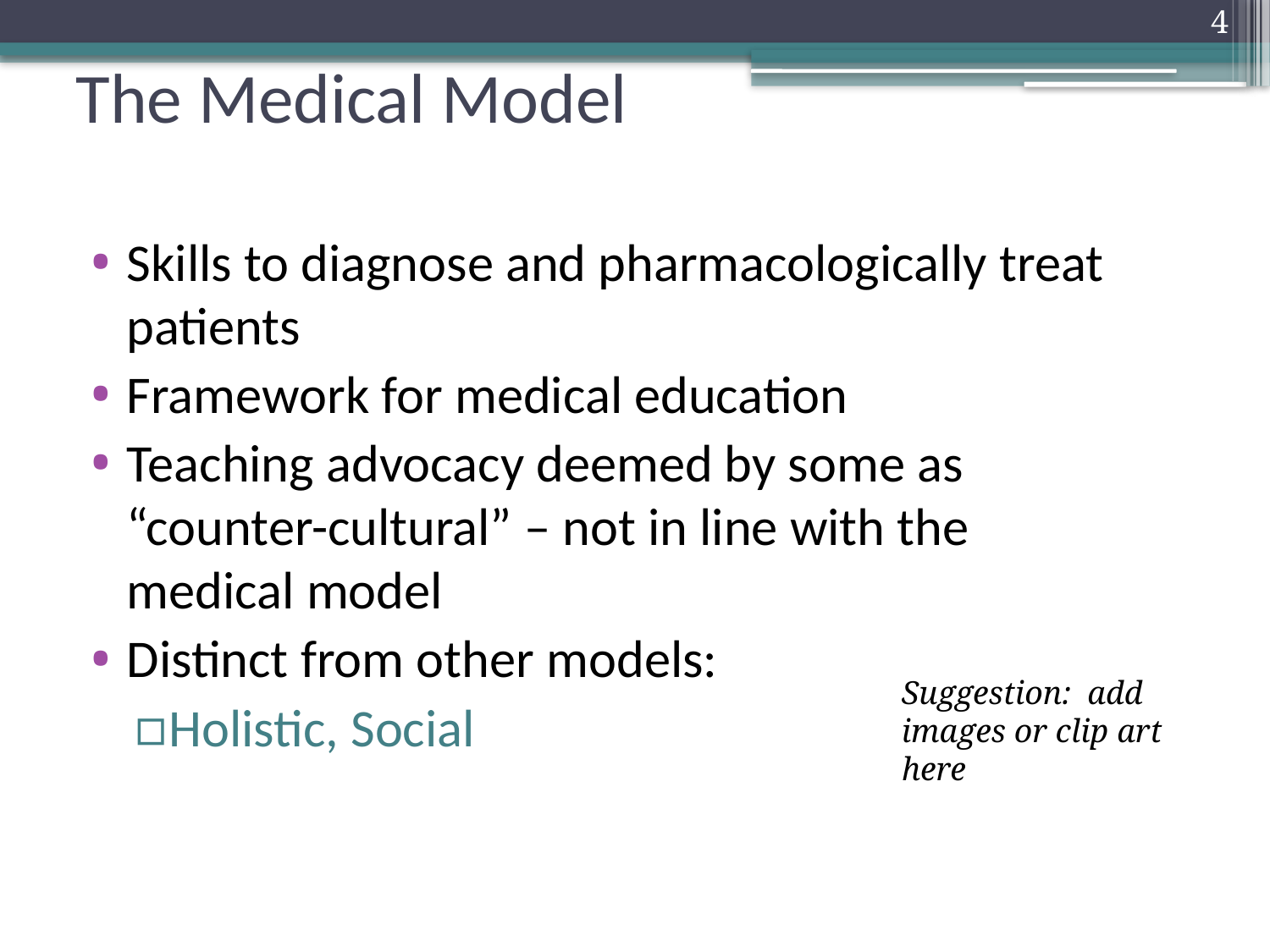

# The Medical Model
4
Skills to diagnose and pharmacologically treat patients
Framework for medical education
Teaching advocacy deemed by some as “counter-cultural” – not in line with the medical model
Distinct from other models:
Holistic, Social
Suggestion: add images or clip art here

## Slide 5
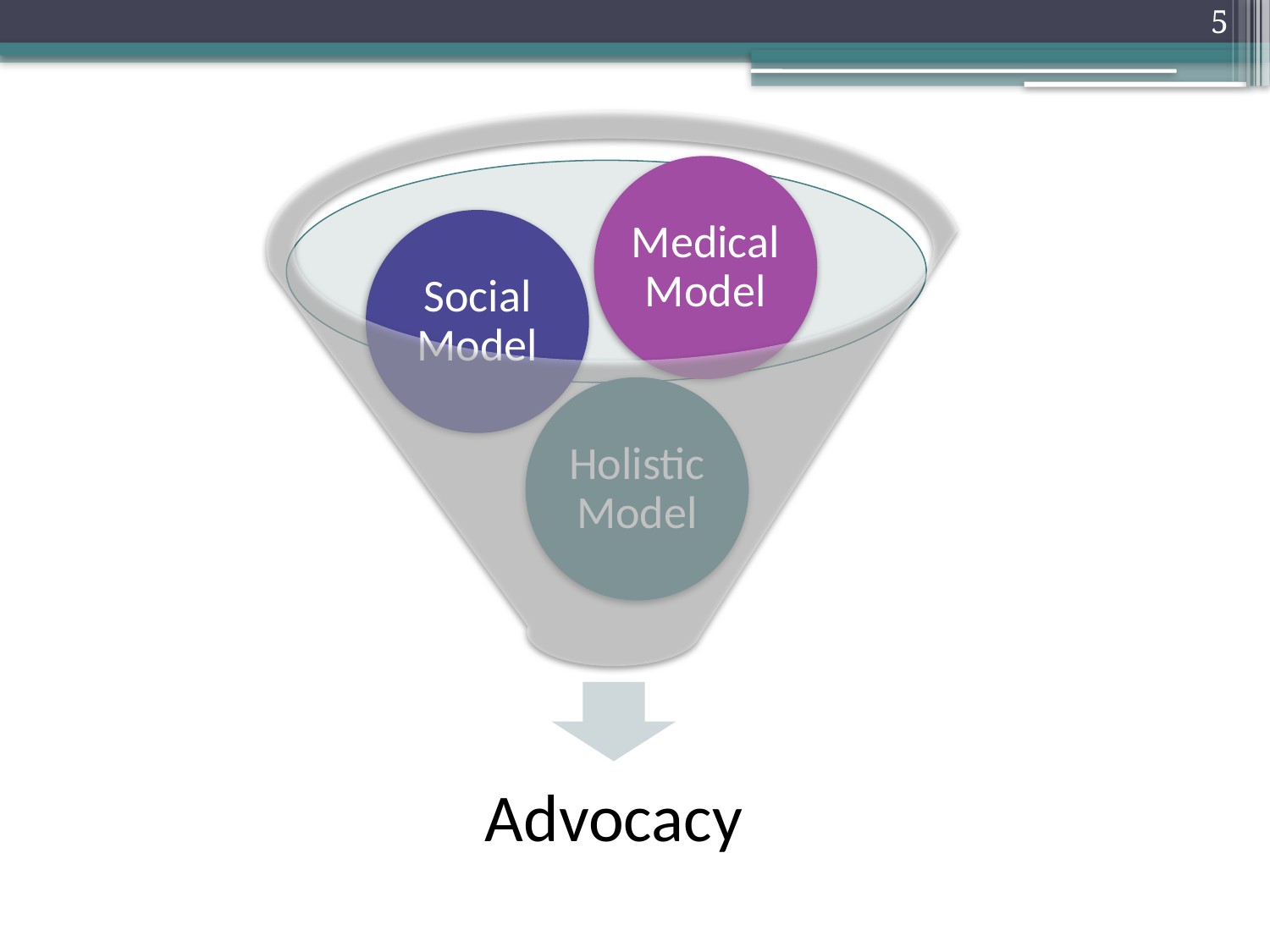

5

## Slide 6
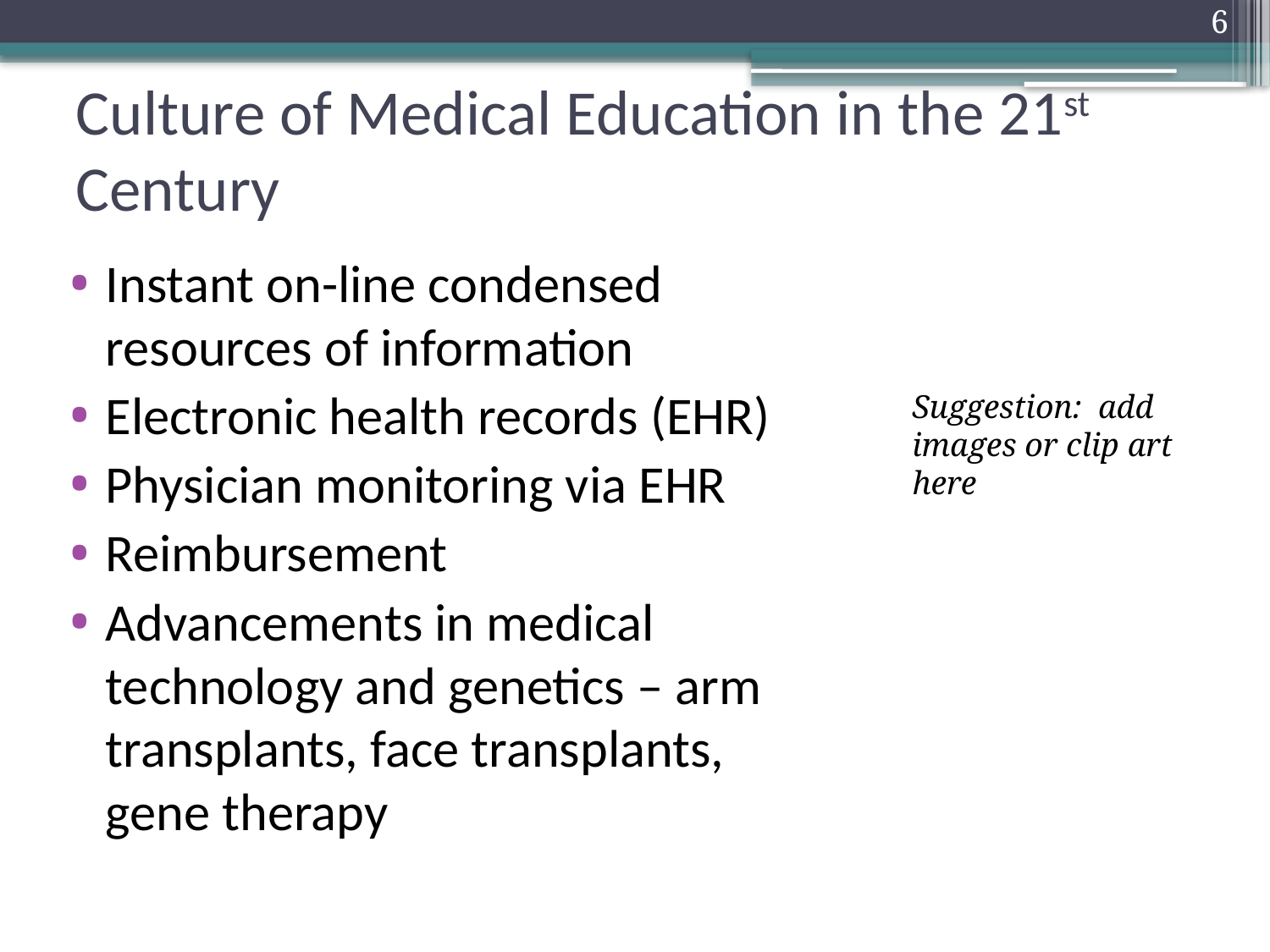

6
# Culture of Medical Education in the 21st Century
Instant on-line condensed resources of information
Electronic health records (EHR)
Physician monitoring via EHR
Reimbursement
Advancements in medical technology and genetics – arm transplants, face transplants, gene therapy
Suggestion: add images or clip art here

## Slide 7
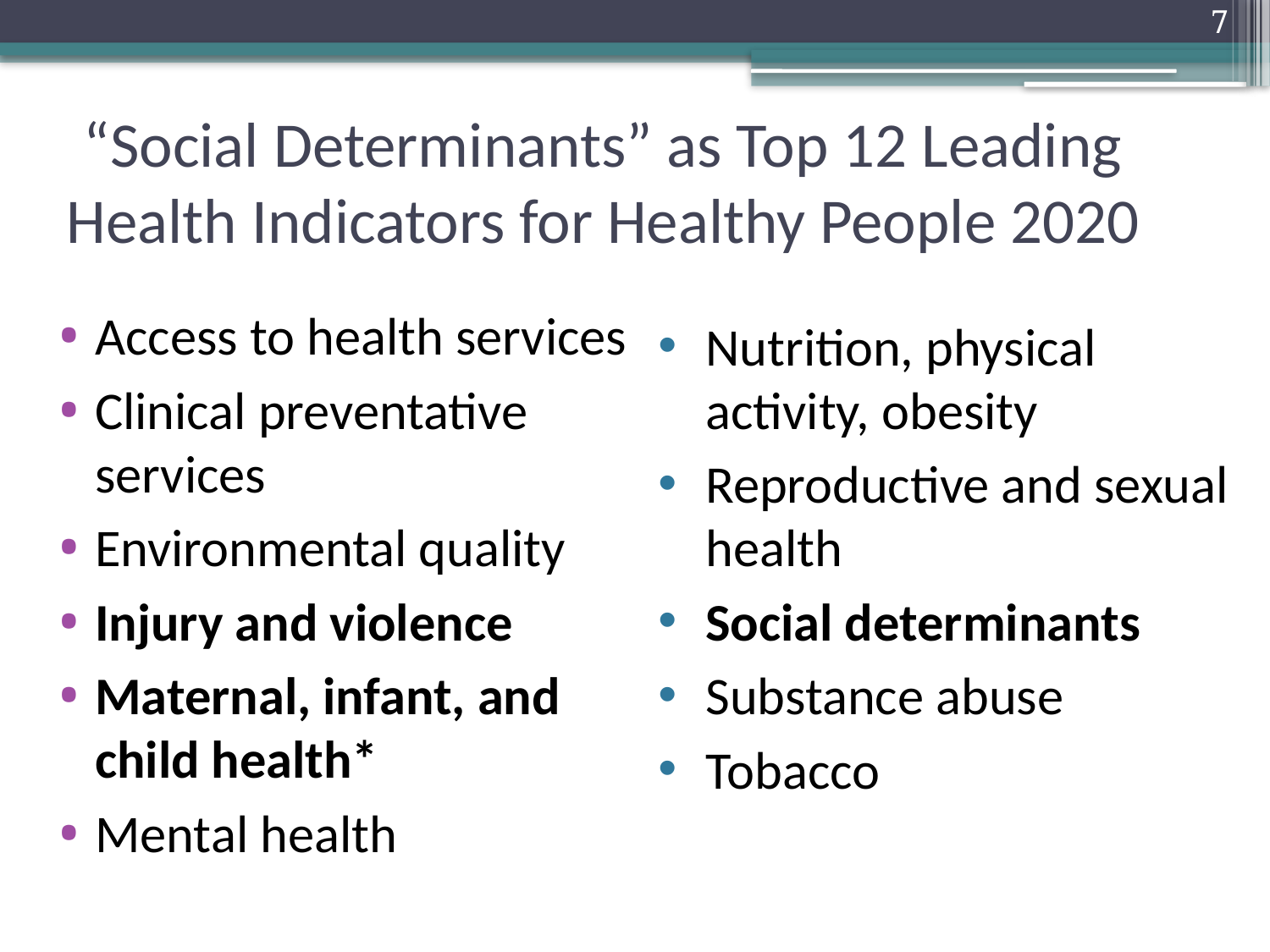

7
# “Social Determinants” as Top 12 Leading Health Indicators for Healthy People 2020
Access to health services
Clinical preventative services
Environmental quality
Injury and violence
Maternal, infant, and child health*
Mental health
Nutrition, physical activity, obesity
Reproductive and sexual health
Social determinants
Substance abuse
Tobacco

## Slide 8
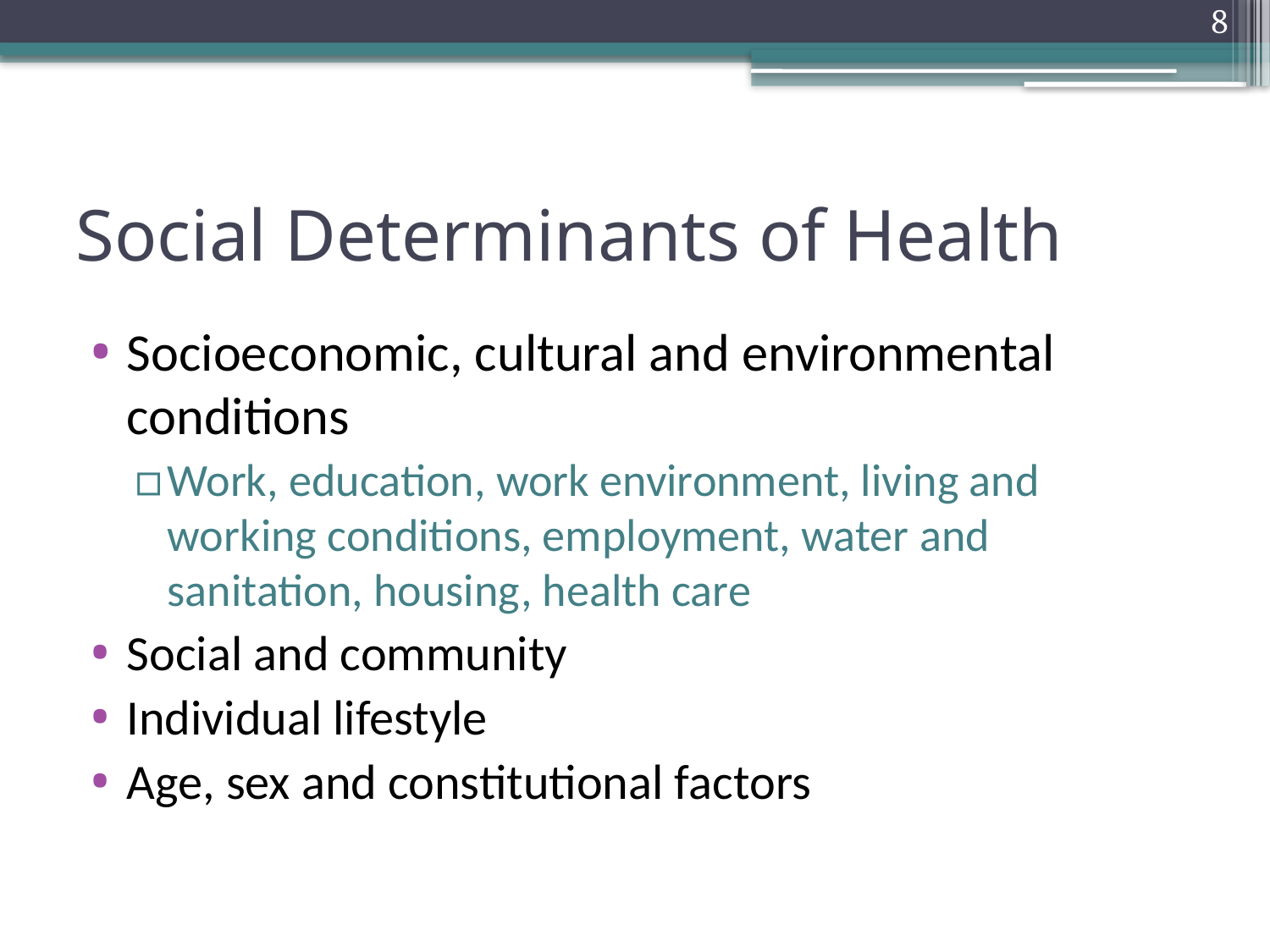

8
# Social Determinants of Health
Socioeconomic, cultural and environmental conditions
Work, education, work environment, living and working conditions, employment, water and sanitation, housing, health care
Social and community
Individual lifestyle
Age, sex and constitutional factors

## Slide 9
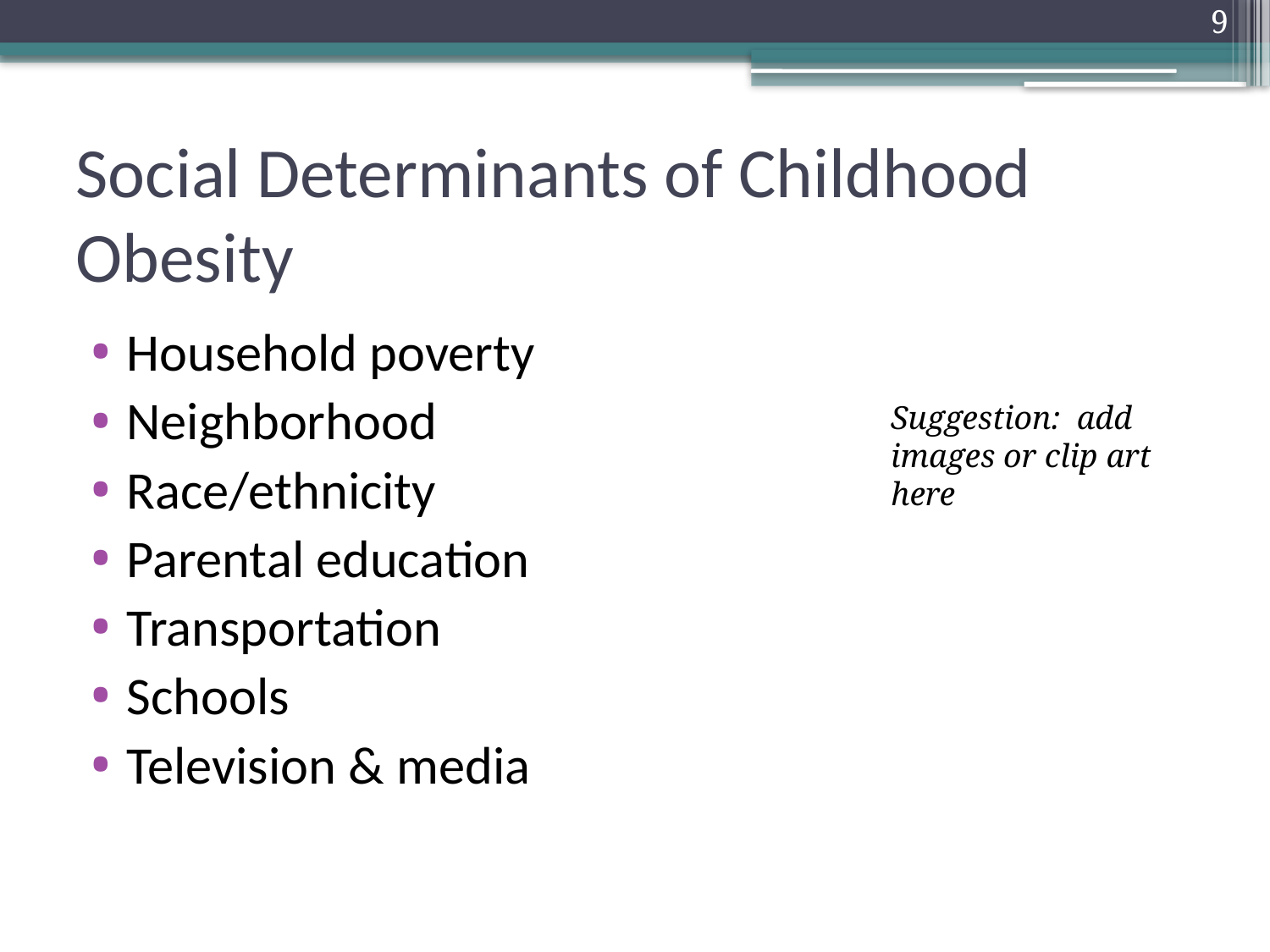

9
# Social Determinants of Childhood Obesity
Household poverty
Neighborhood
Race/ethnicity
Parental education
Transportation
Schools
Television & media
Suggestion: add images or clip art here

## Slide 10
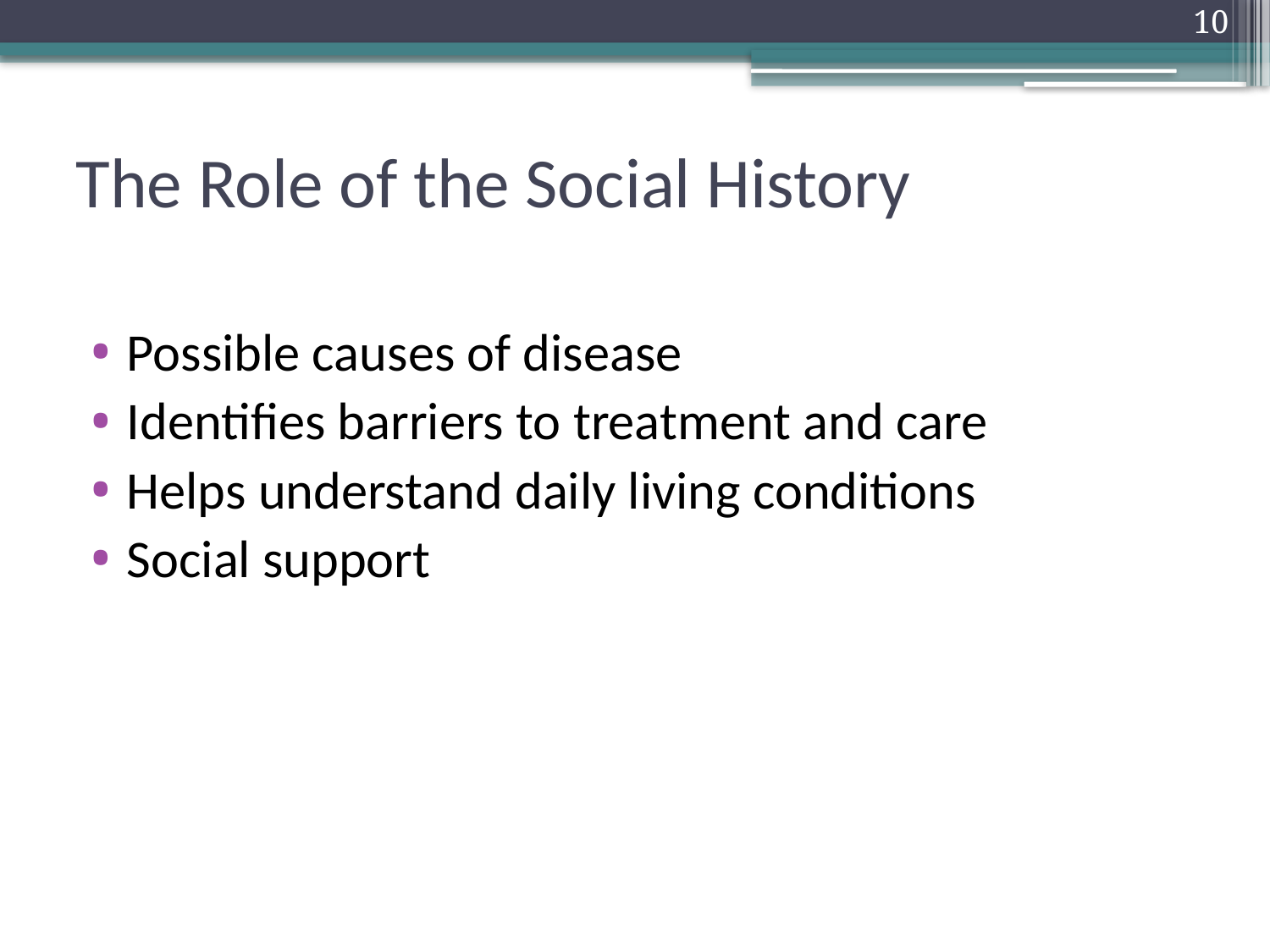

10
# The Role of the Social History
Possible causes of disease
Identifies barriers to treatment and care
Helps understand daily living conditions
Social support

## Slide 11
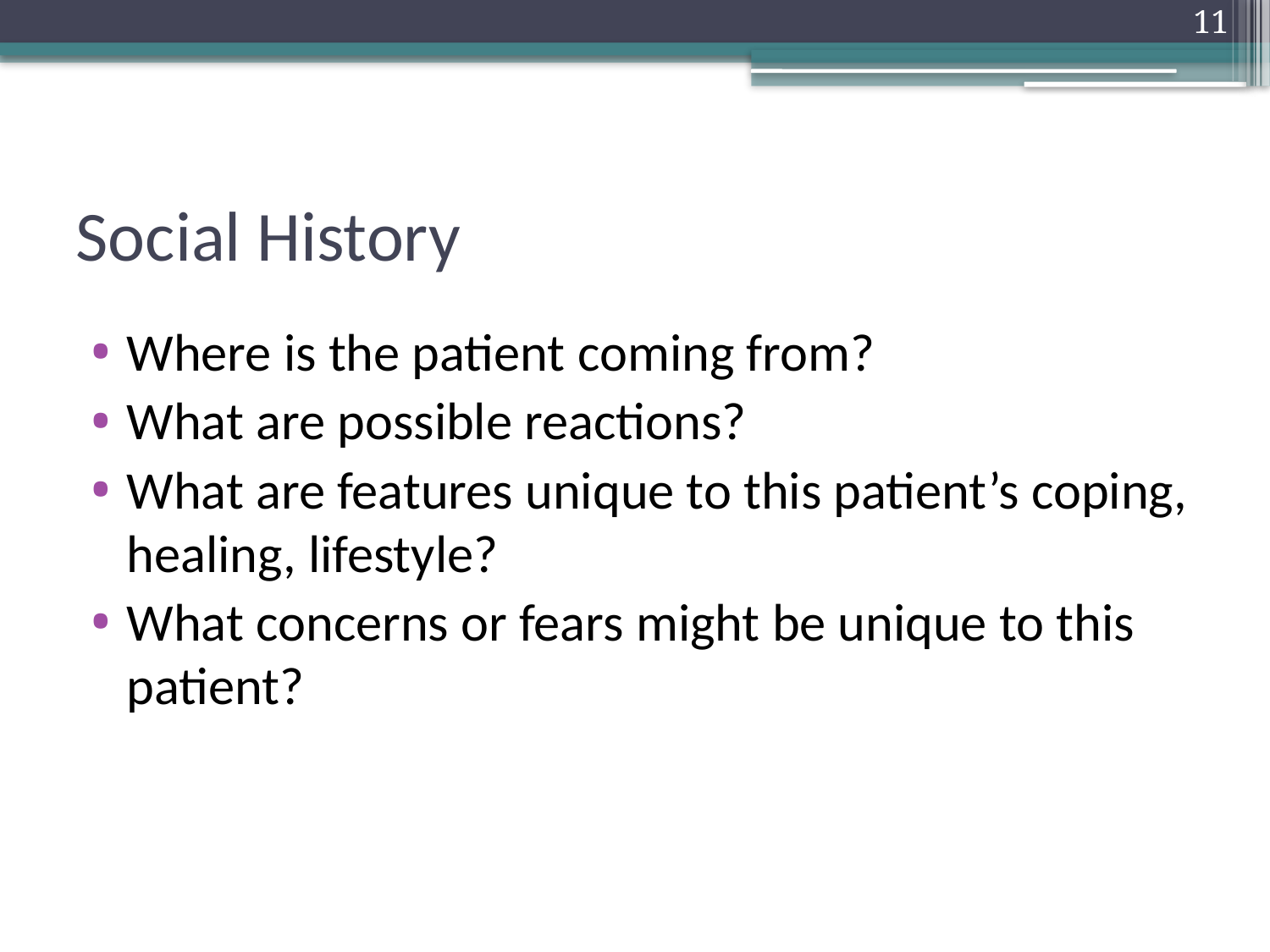

11
# Social History
Where is the patient coming from?
What are possible reactions?
What are features unique to this patient’s coping, healing, lifestyle?
What concerns or fears might be unique to this patient?

## Slide 12
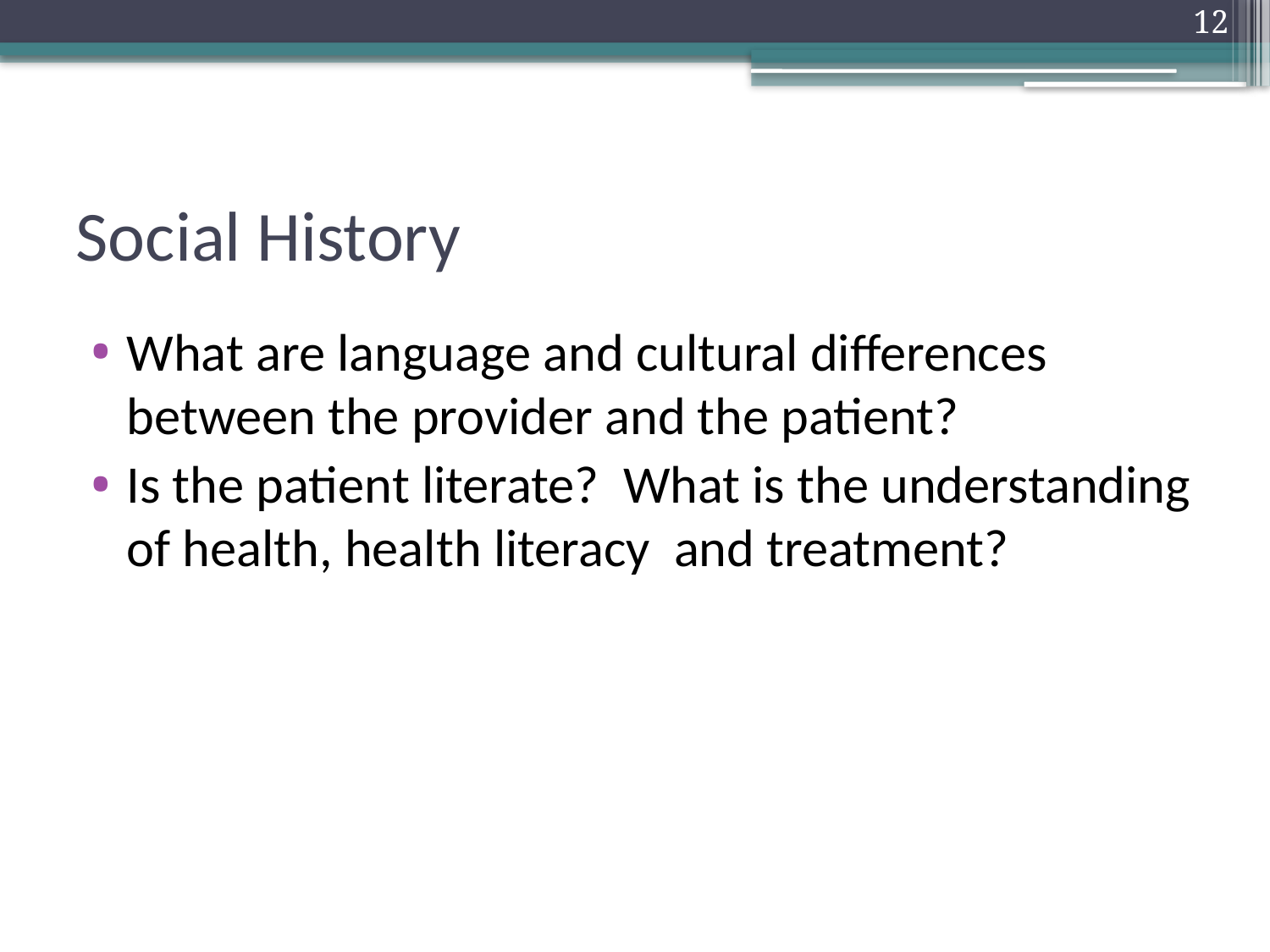

12
# Social History
What are language and cultural differences between the provider and the patient?
Is the patient literate? What is the understanding of health, health literacy and treatment?

## Slide 13
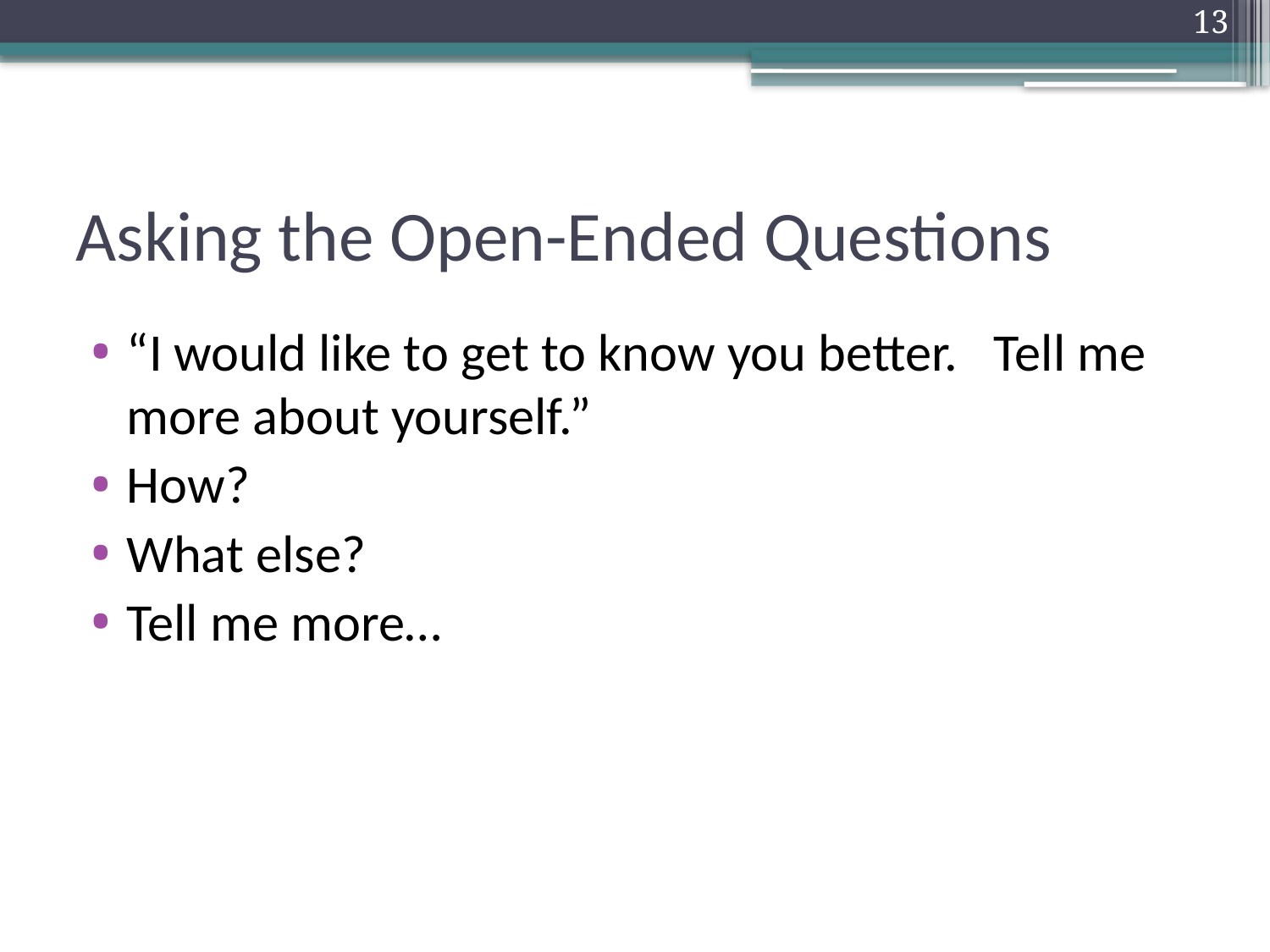

13
# Asking the Open-Ended Questions
“I would like to get to know you better. Tell me more about yourself.”
How?
What else?
Tell me more…

## Slide 14
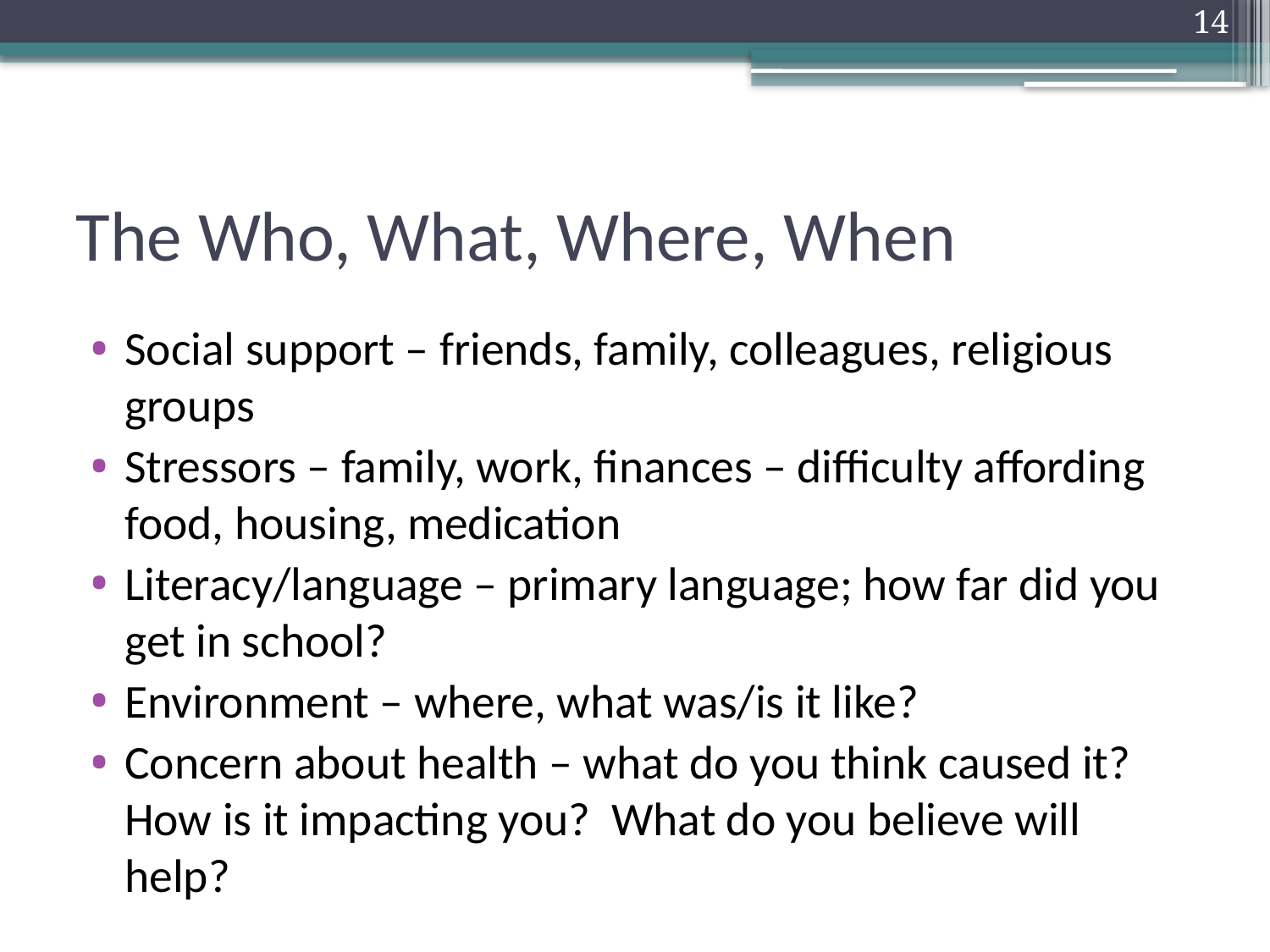

14
# The Who, What, Where, When
Social support – friends, family, colleagues, religious groups
Stressors – family, work, finances – difficulty affording food, housing, medication
Literacy/language – primary language; how far did you get in school?
Environment – where, what was/is it like?
Concern about health – what do you think caused it? How is it impacting you? What do you believe will help?

## Slide 15
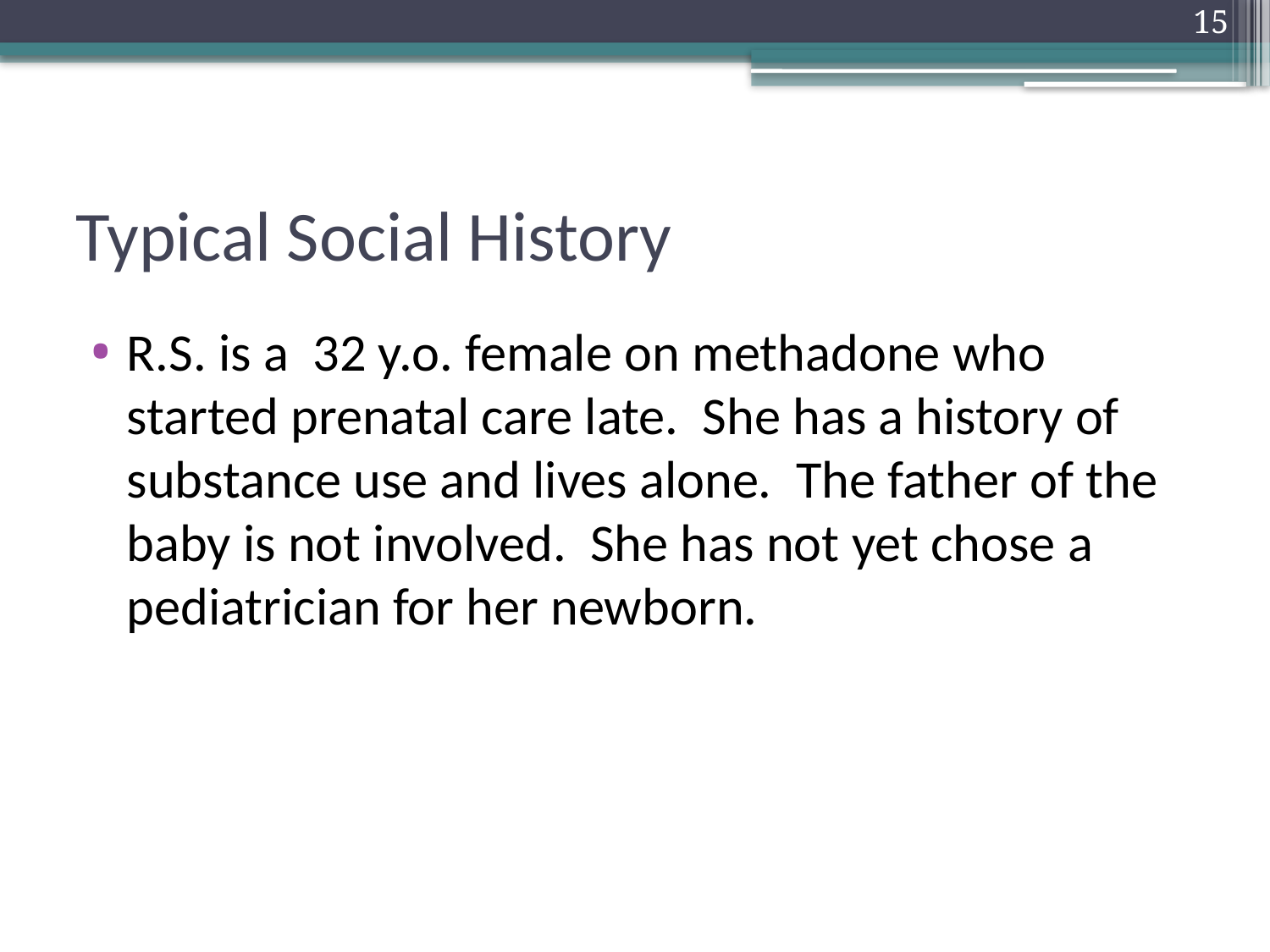

15
# Typical Social History
R.S. is a 32 y.o. female on methadone who started prenatal care late. She has a history of substance use and lives alone. The father of the baby is not involved. She has not yet chose a pediatrician for her newborn.

## Slide 16
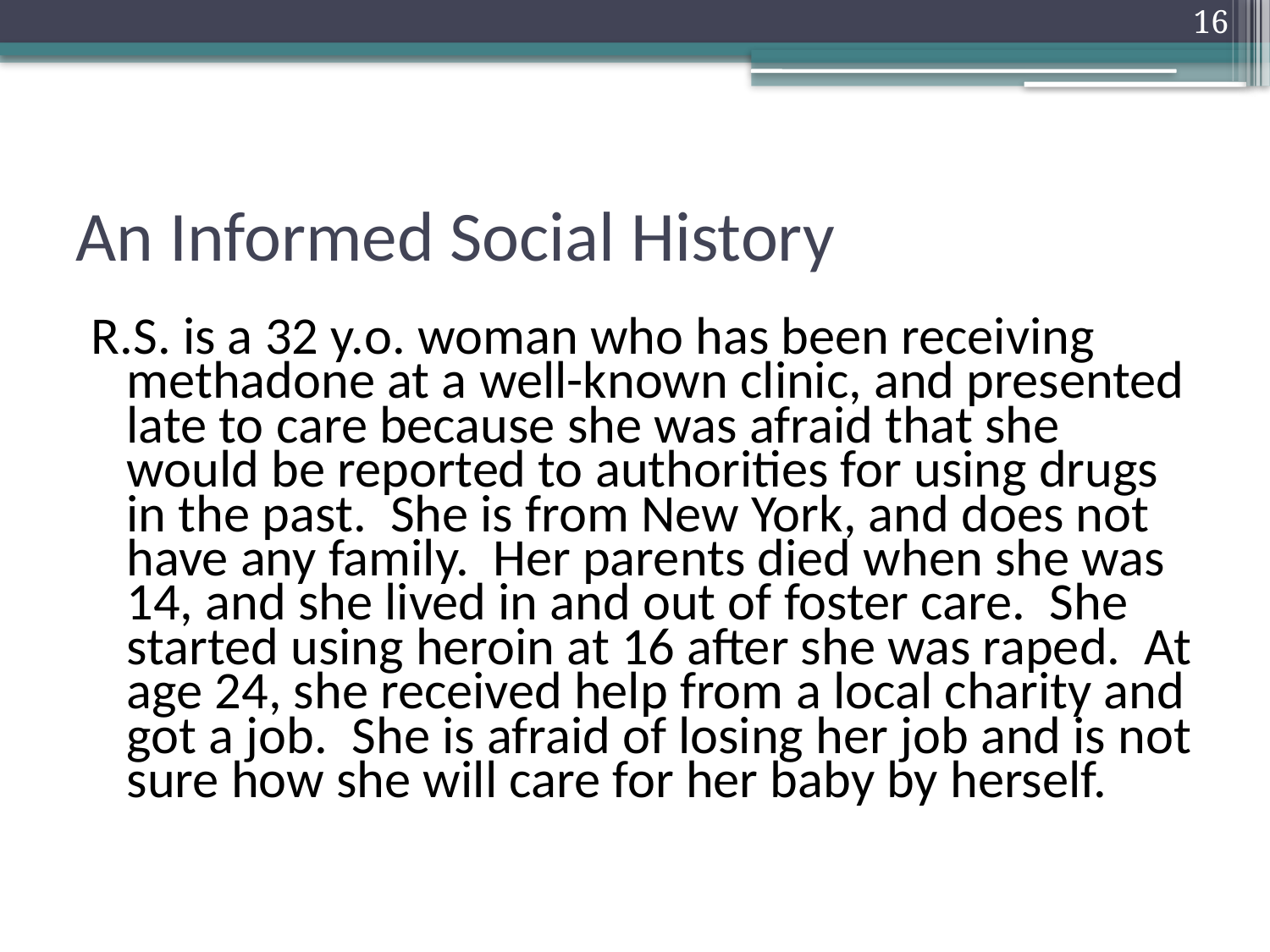

16
# An Informed Social History
R.S. is a 32 y.o. woman who has been receiving methadone at a well-known clinic, and presented late to care because she was afraid that she would be reported to authorities for using drugs in the past. She is from New York, and does not have any family. Her parents died when she was 14, and she lived in and out of foster care. She started using heroin at 16 after she was raped. At age 24, she received help from a local charity and got a job. She is afraid of losing her job and is not sure how she will care for her baby by herself.

## Slide 17
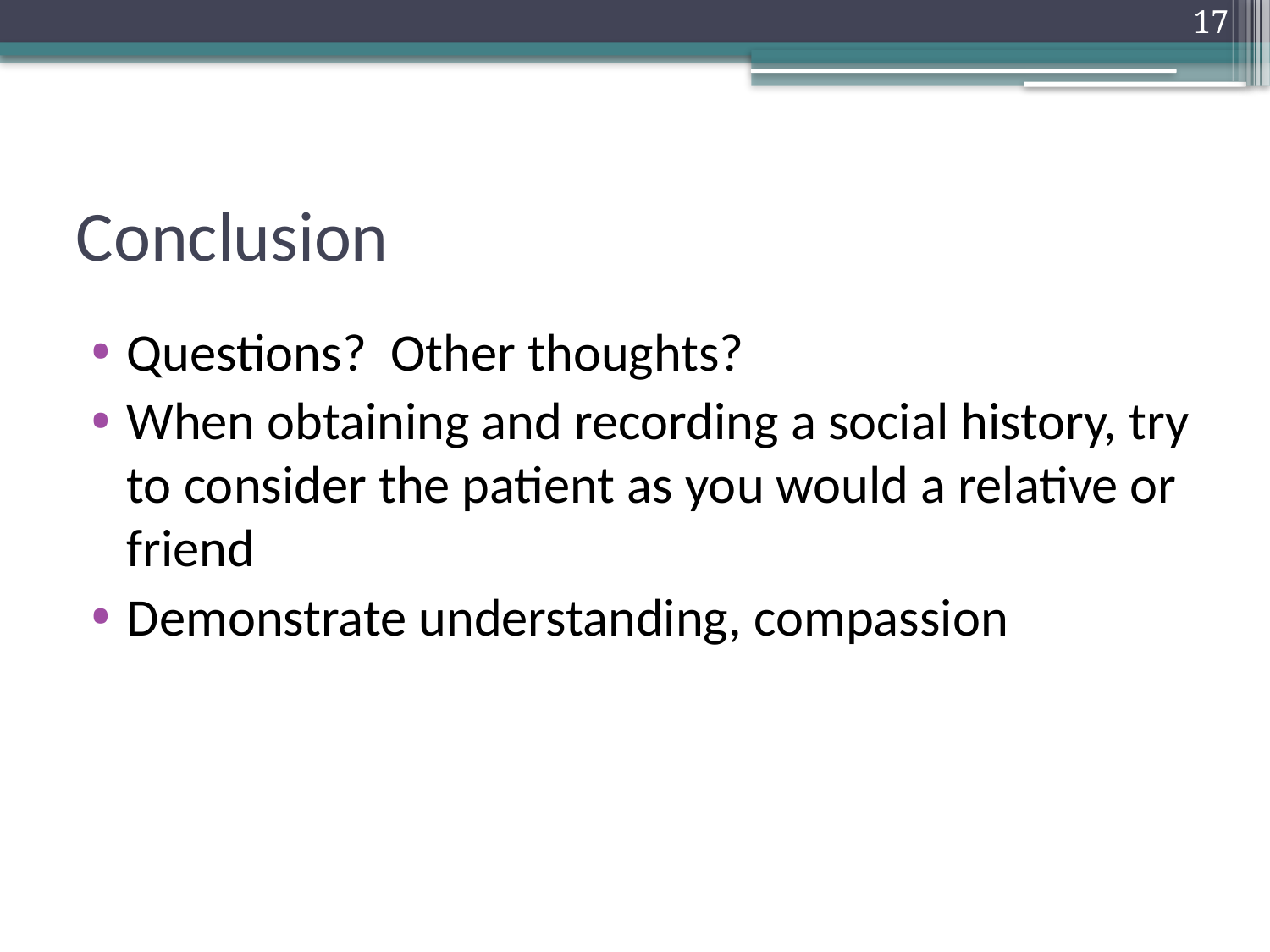

17
# Conclusion
Questions? Other thoughts?
When obtaining and recording a social history, try to consider the patient as you would a relative or friend
Demonstrate understanding, compassion
