# Supplementary material for: The JeffSTARS Advocacy and Community Partnership Elective: A Closer Look at Child Health Advocacy in Action
Source: MedEdPORTAL. 2016 Dec 31;12:10526. doi: 10.15766/mep_2374-8265.10526 (PMC6365684; doi:10.15766/mep_2374-8265.10526)
Supplement: Supplementary file 1 — A. CM1. Course Implementation at New Institution Checklist.docx B. CM2. Elective Checklist.docx C. CM3. Sample Schedule.docx D. CM4. Seminar Topic List With Learning Objectives.docx E. CM5. Syllabus Bibliography.docx F. CM6. List of Community Partners.docx G. CM7. Orientation for New Community Partner.docx H. CM8. Selected Past Projects.docx I. CM9. Sample Fact Sheets for Legislative Visits.docx J. Seminar Materials folder K. ET1. Advocacy Elective Assessment 1.pdf L. ET2. Advocacy Elective Assessment 2.pdf M. ET3. Trainee Evaluation by Community or Faculty Mentor.docx N. ET4. Trainee Evaluation of Seminar.docx O. ET5. Trainee Evaluation of Community Partner.docx P. ET6. Final Report Template.docx Q. Selected Trainee Abstracts and Presented Results folder [file mep-12-10526-s001.zip › J._Seminar_Materials_folder/6._Maternal_Child_Global.pptx]

## Slide 1
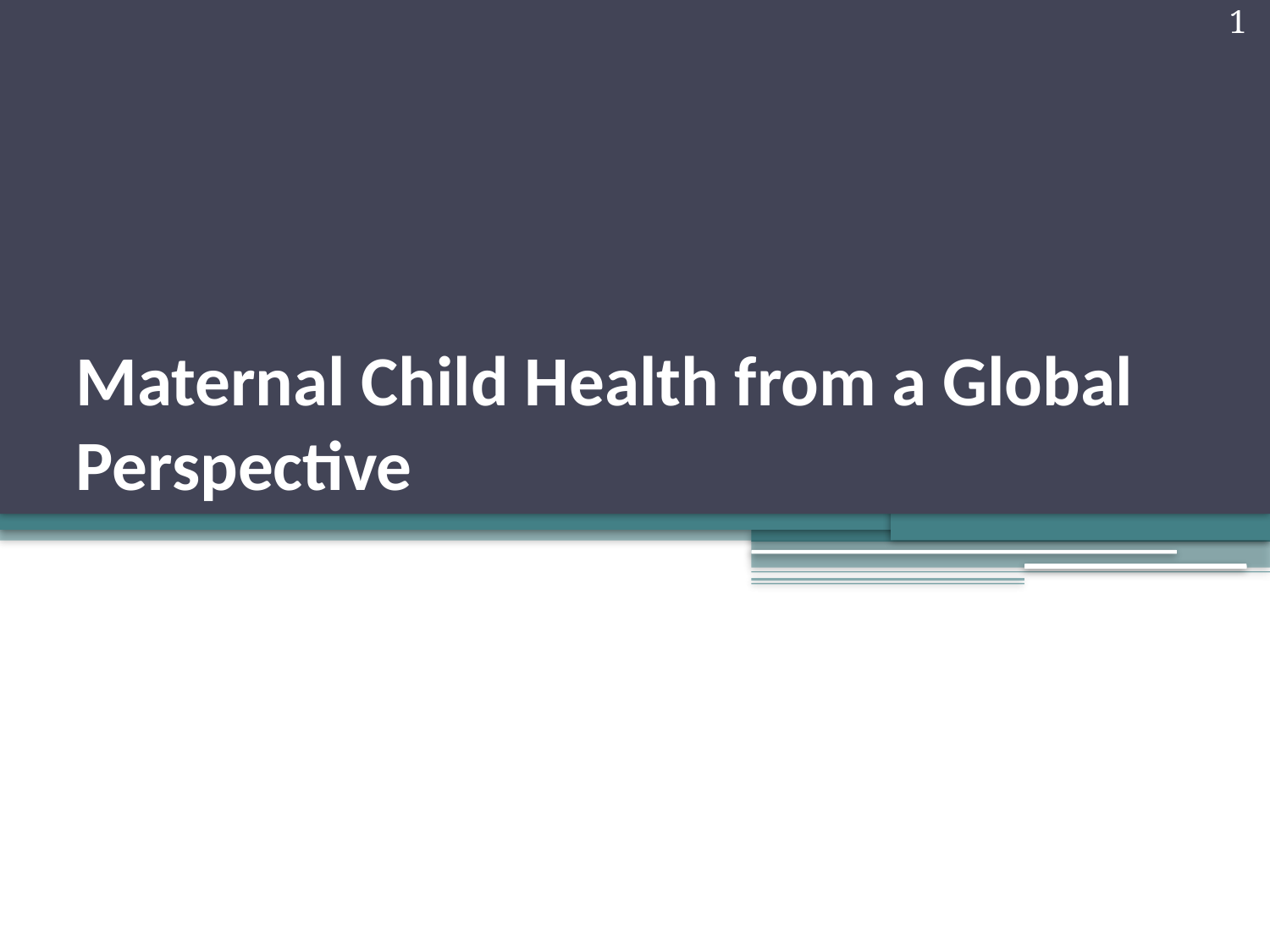

1
# Maternal Child Health from a Global Perspective

## Slide 2
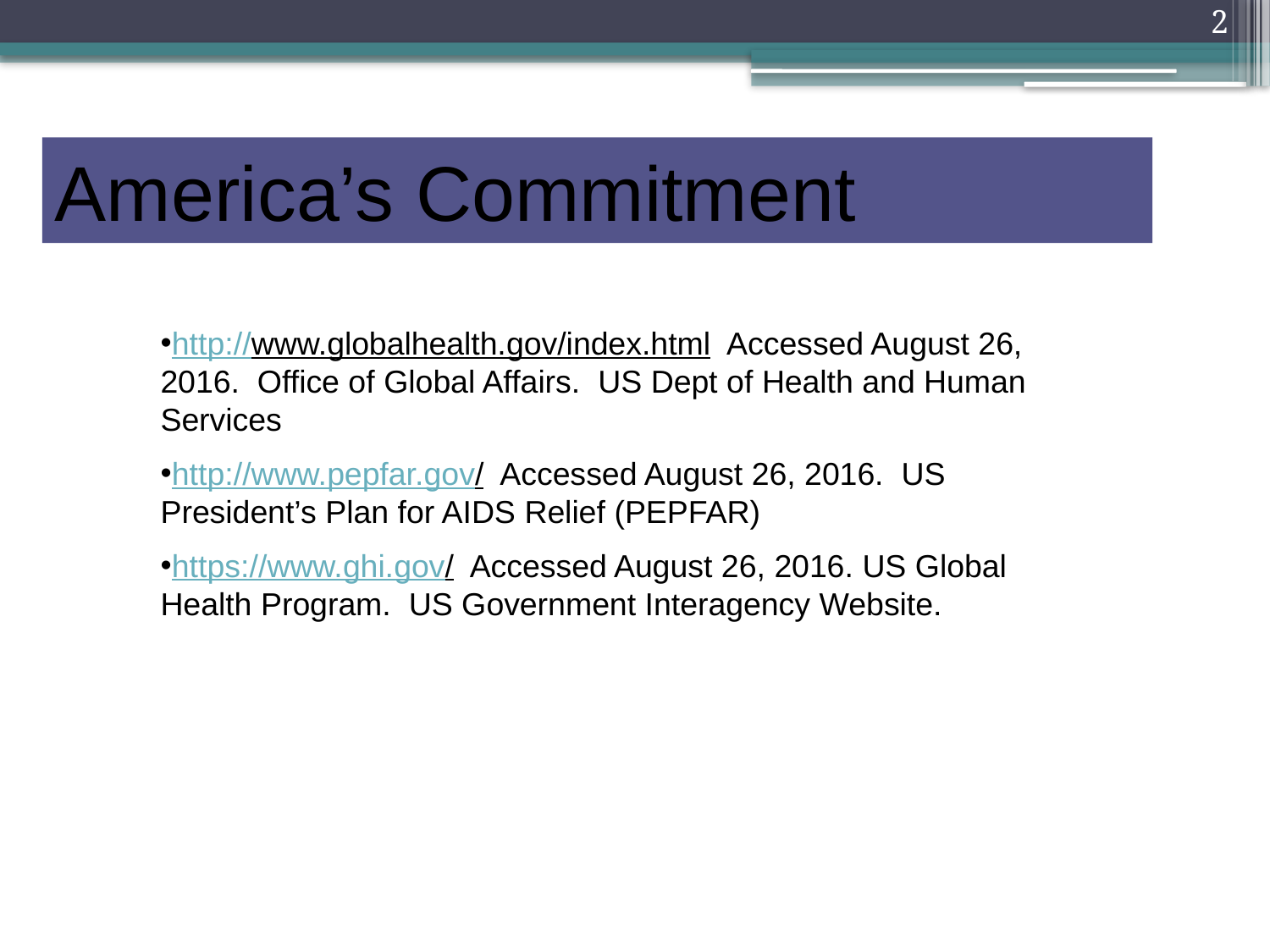

2
America’s Commitment
http://www.globalhealth.gov/index.html Accessed August 26, 2016. Office of Global Affairs. US Dept of Health and Human Services
http://www.pepfar.gov/ Accessed August 26, 2016. US President’s Plan for AIDS Relief (PEPFAR)
https://www.ghi.gov/ Accessed August 26, 2016. US Global Health Program. US Government Interagency Website.

## Slide 3
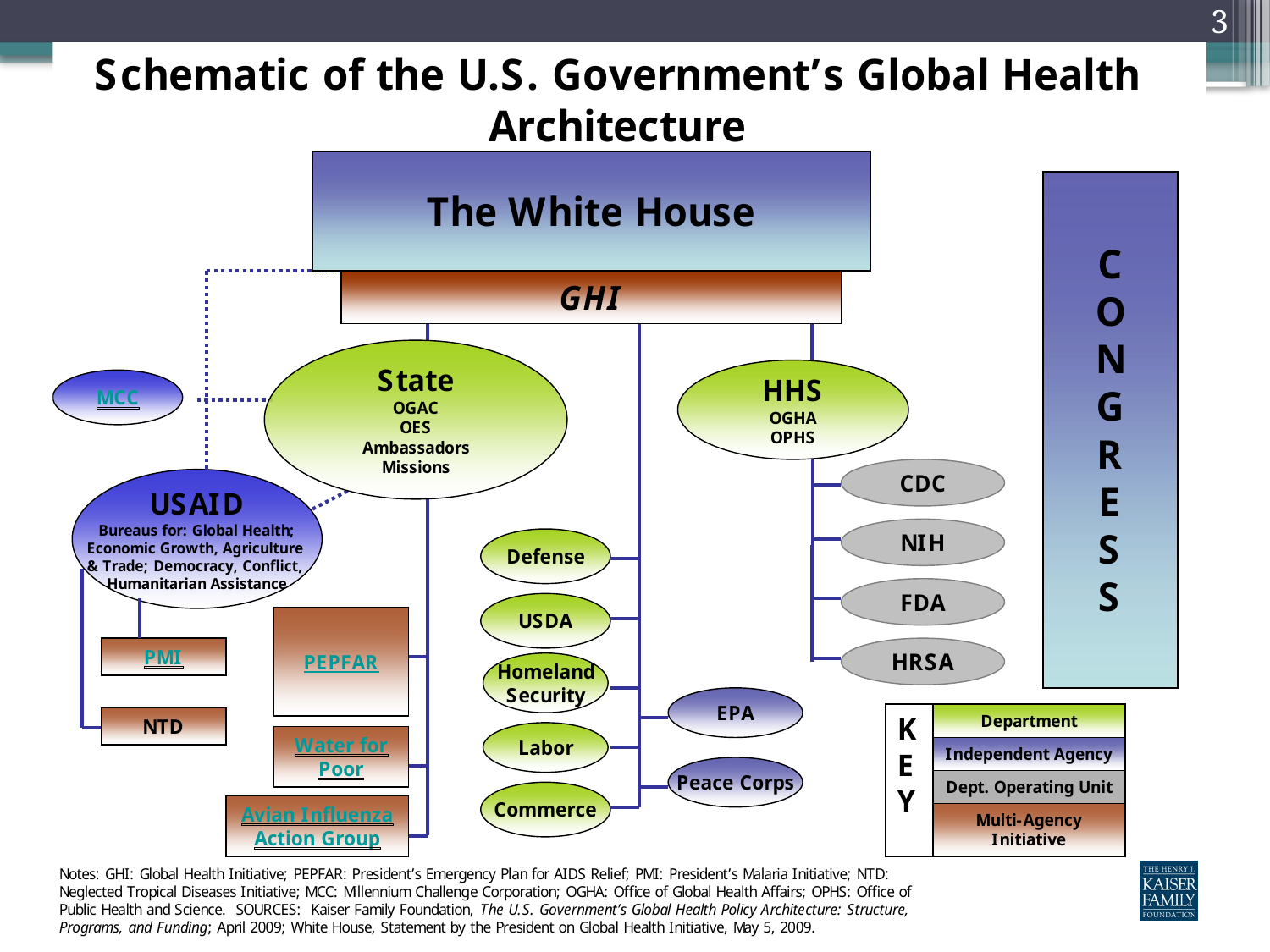

3
#

## Slide 4
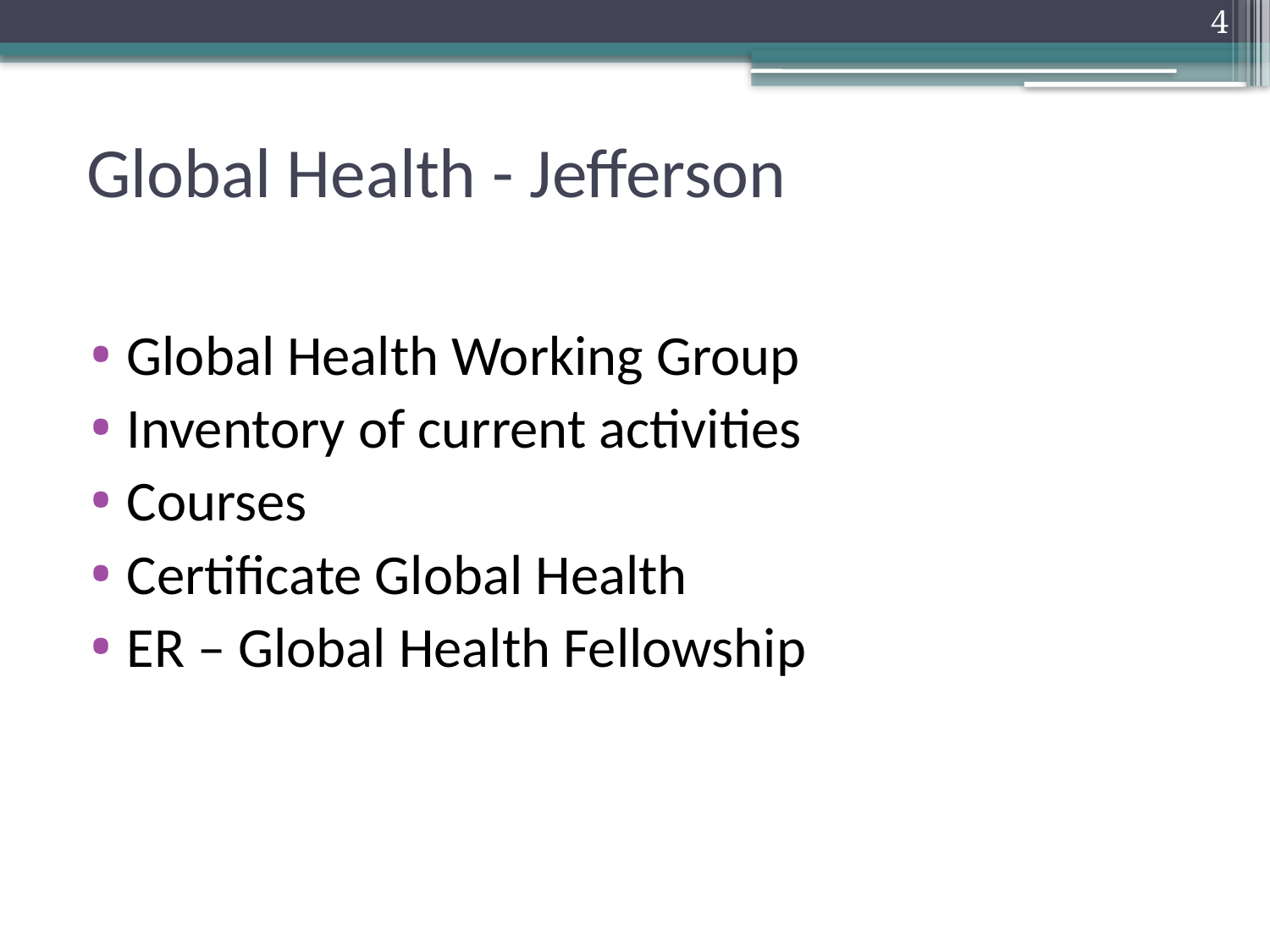

4
# Global Health - Jefferson
Global Health Working Group
Inventory of current activities
Courses
Certificate Global Health
ER – Global Health Fellowship

## Slide 5
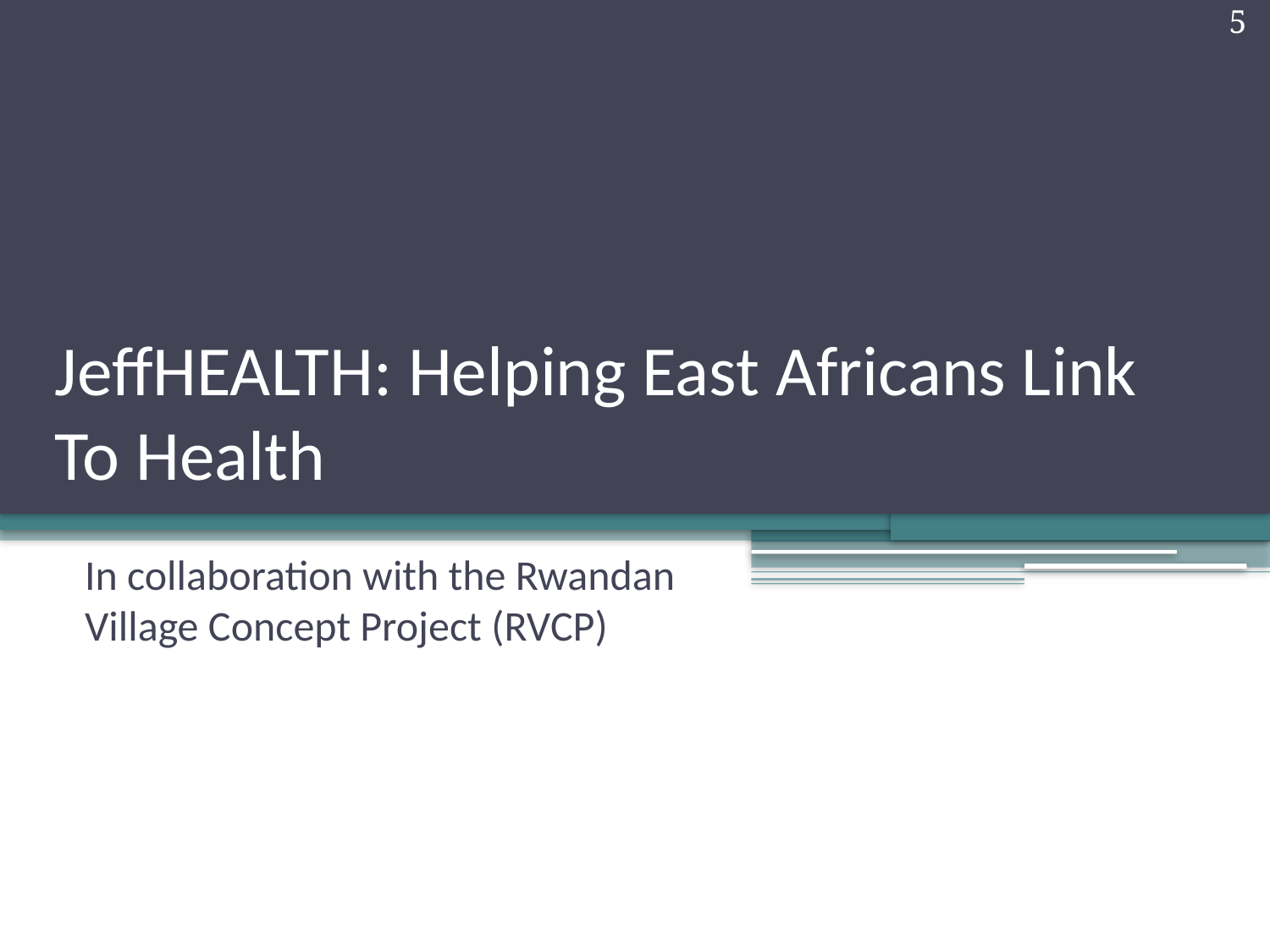

5
# JeffHEALTH: Helping East Africans Link To Health
In collaboration with the Rwandan Village Concept Project (RVCP)

## Slide 6
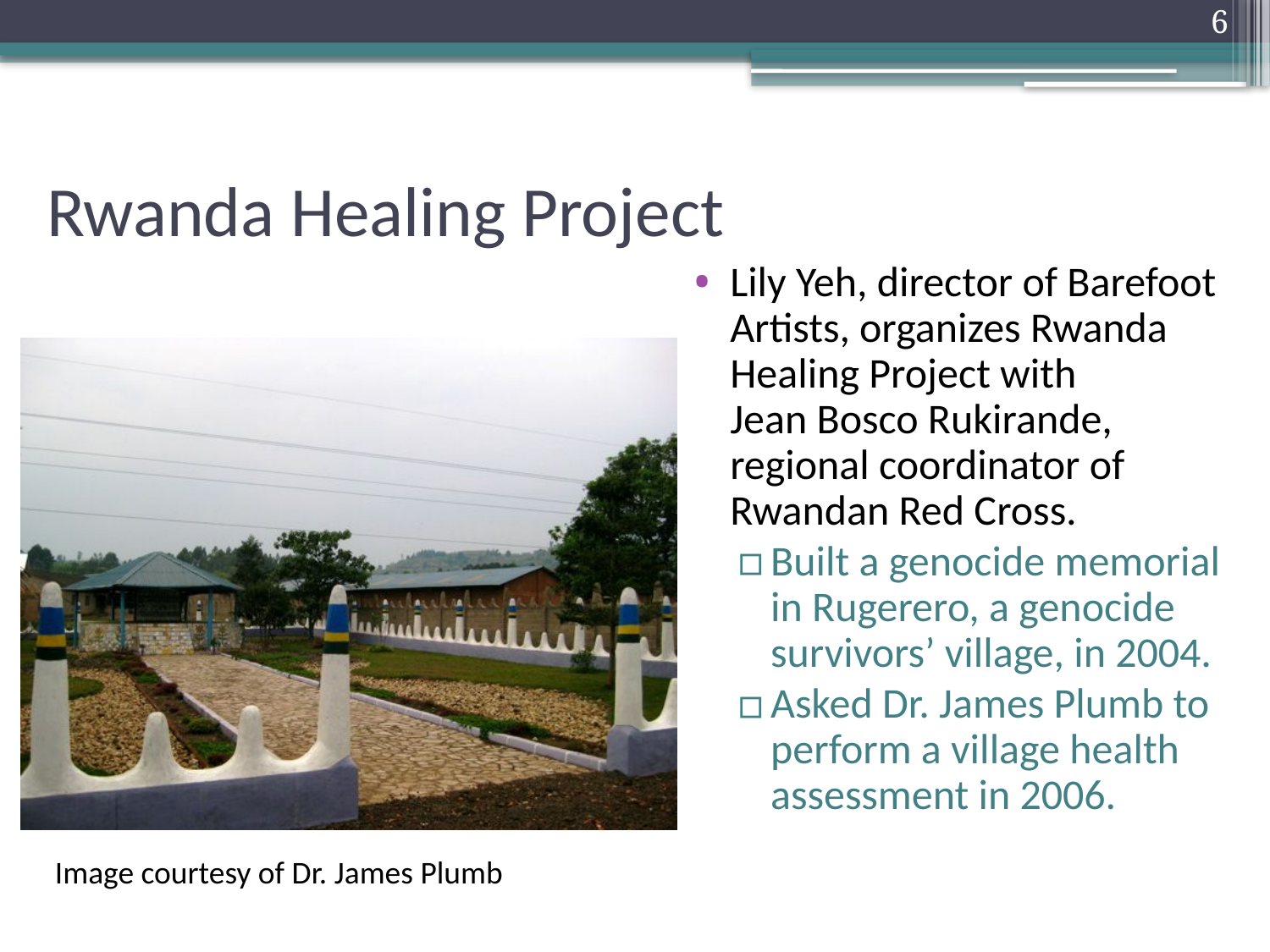

6
# Rwanda Healing Project
Lily Yeh, director of Barefoot Artists, organizes Rwanda Healing Project with Jean Bosco Rukirande, regional coordinator of Rwandan Red Cross.
Built a genocide memorial in Rugerero, a genocide survivors’ village, in 2004.
Asked Dr. James Plumb to perform a village health assessment in 2006.
Image courtesy of Dr. James Plumb

## Slide 7
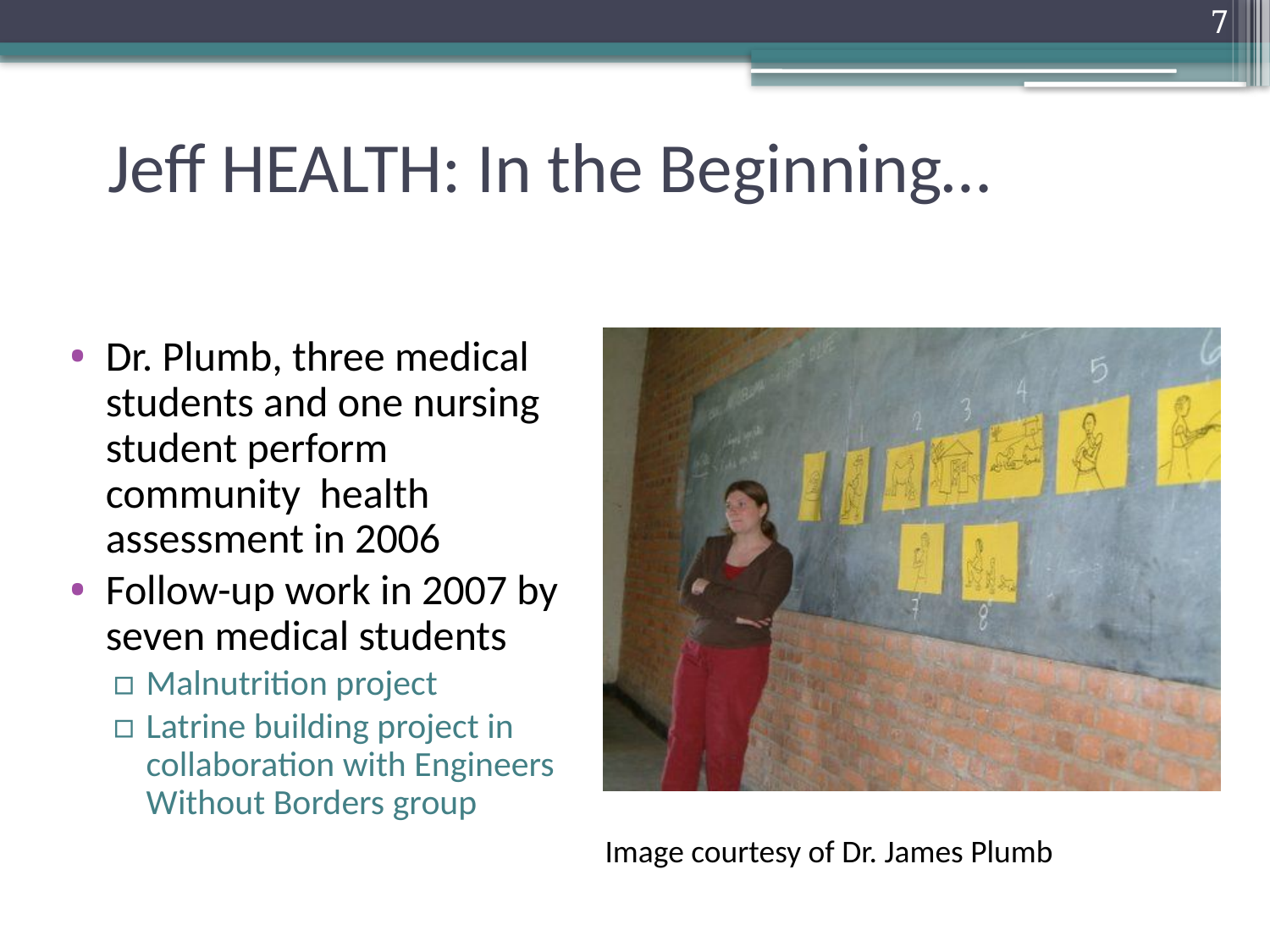

7
# Jeff HEALTH: In the Beginning…
Dr. Plumb, three medical students and one nursing student perform community health assessment in 2006
Follow-up work in 2007 by seven medical students
Malnutrition project
Latrine building project in collaboration with Engineers Without Borders group
Image courtesy of Dr. James Plumb

## Slide 8
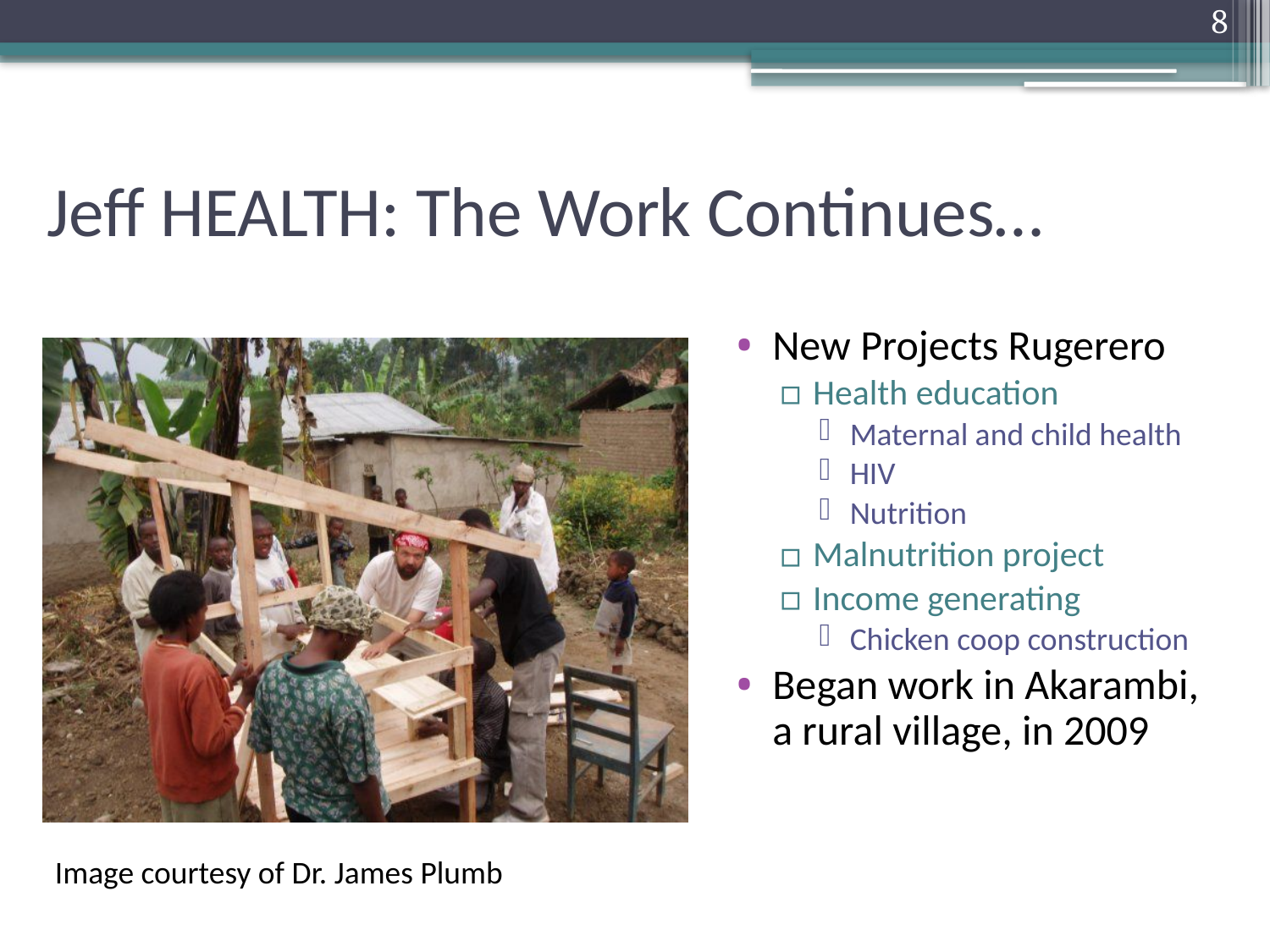

8
# Jeff HEALTH: The Work Continues…
New Projects Rugerero
Health education
Maternal and child health
HIV
Nutrition
Malnutrition project
Income generating
Chicken coop construction
Began work in Akarambi, a rural village, in 2009
Image courtesy of Dr. James Plumb

## Slide 9
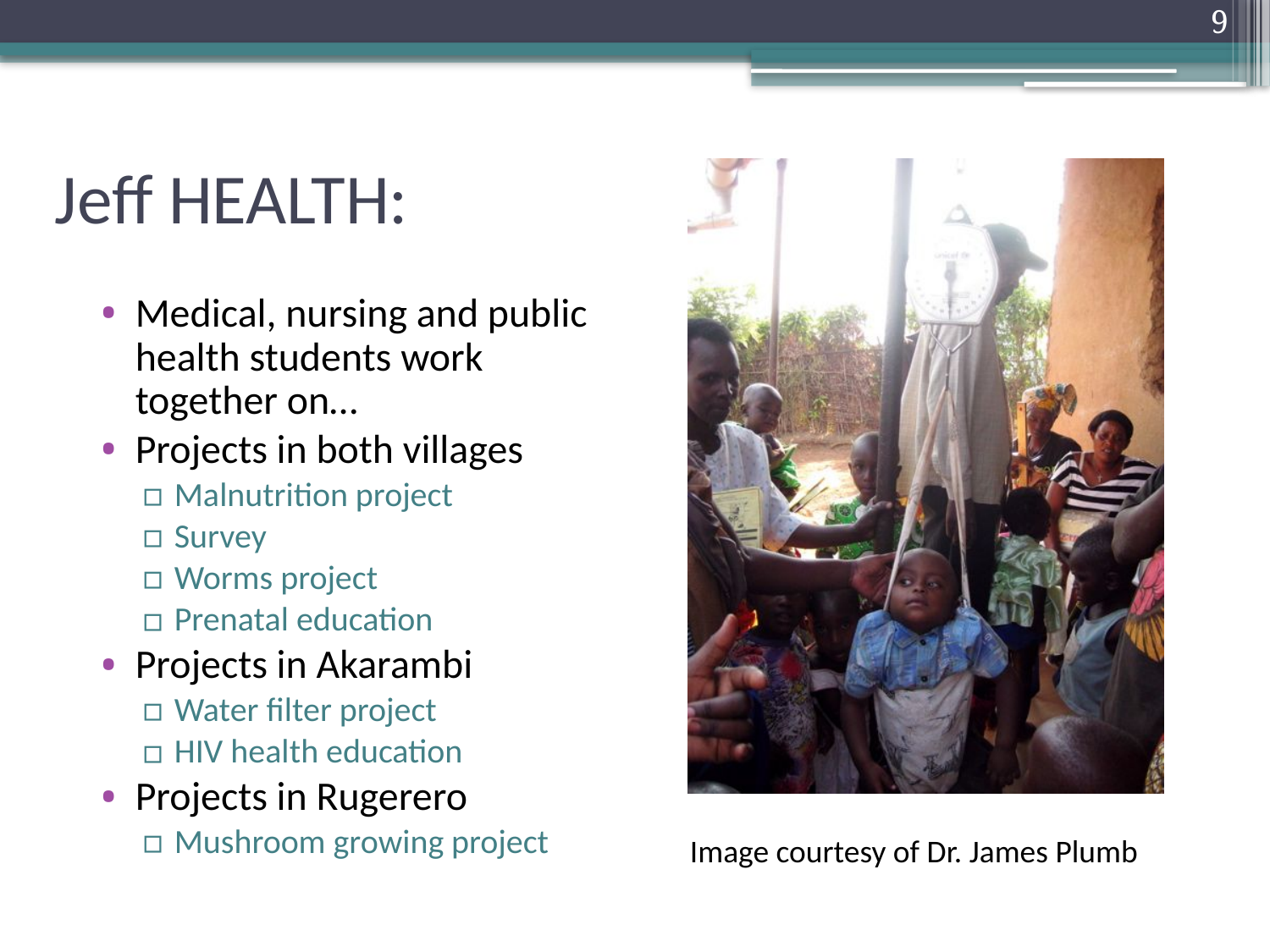

9
# Jeff HEALTH:
Medical, nursing and public health students work together on…
Projects in both villages
Malnutrition project
Survey
Worms project
Prenatal education
Projects in Akarambi
Water filter project
HIV health education
Projects in Rugerero
Mushroom growing project
Image courtesy of Dr. James Plumb

## Slide 10
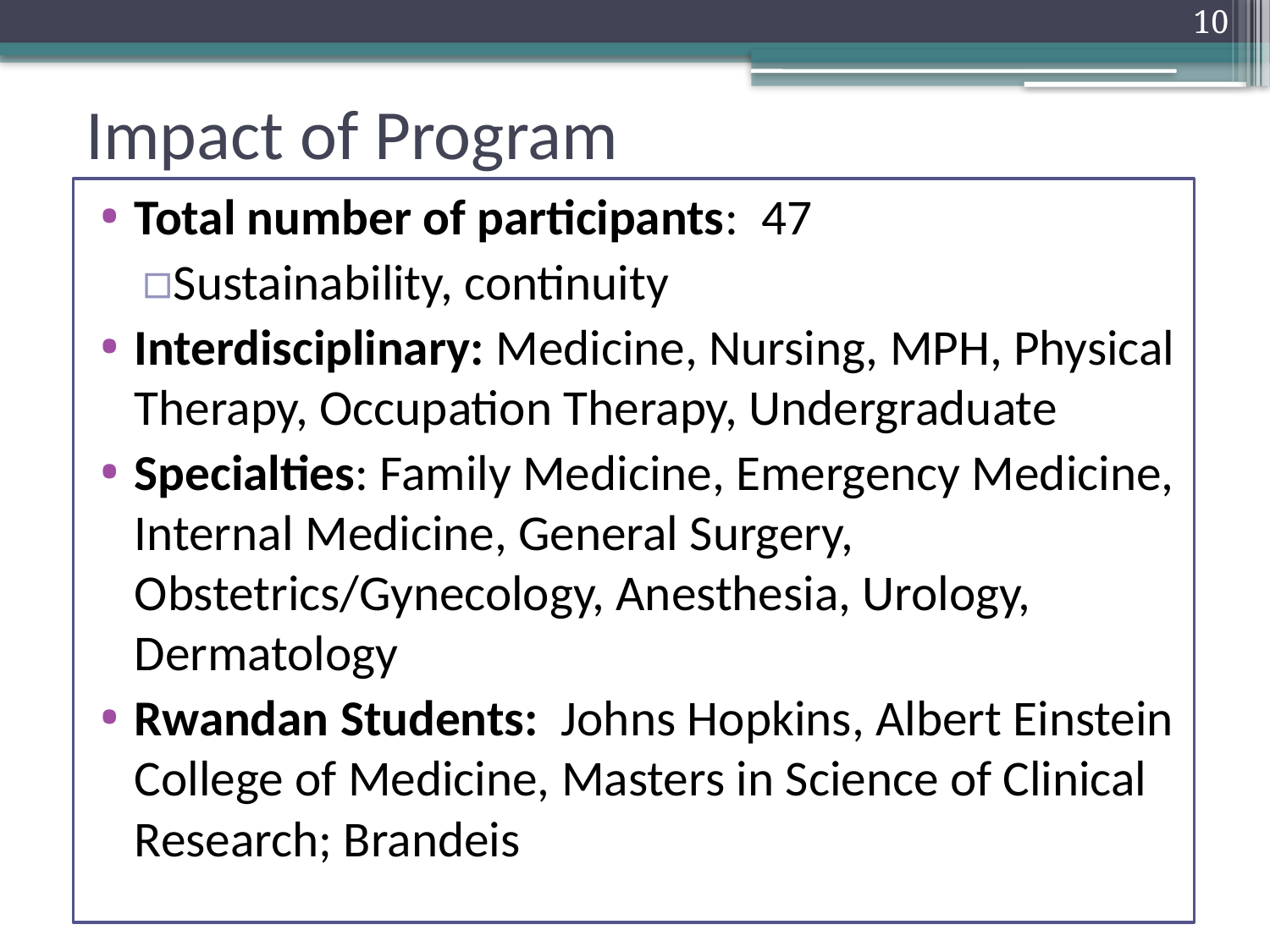

10
Impact of Program
Total number of participants: 47
Sustainability, continuity
Interdisciplinary: Medicine, Nursing, MPH, Physical Therapy, Occupation Therapy, Undergraduate
Specialties: Family Medicine, Emergency Medicine, Internal Medicine, General Surgery, Obstetrics/Gynecology, Anesthesia, Urology, Dermatology
Rwandan Students: Johns Hopkins, Albert Einstein College of Medicine, Masters in Science of Clinical Research; Brandeis

## Slide 11
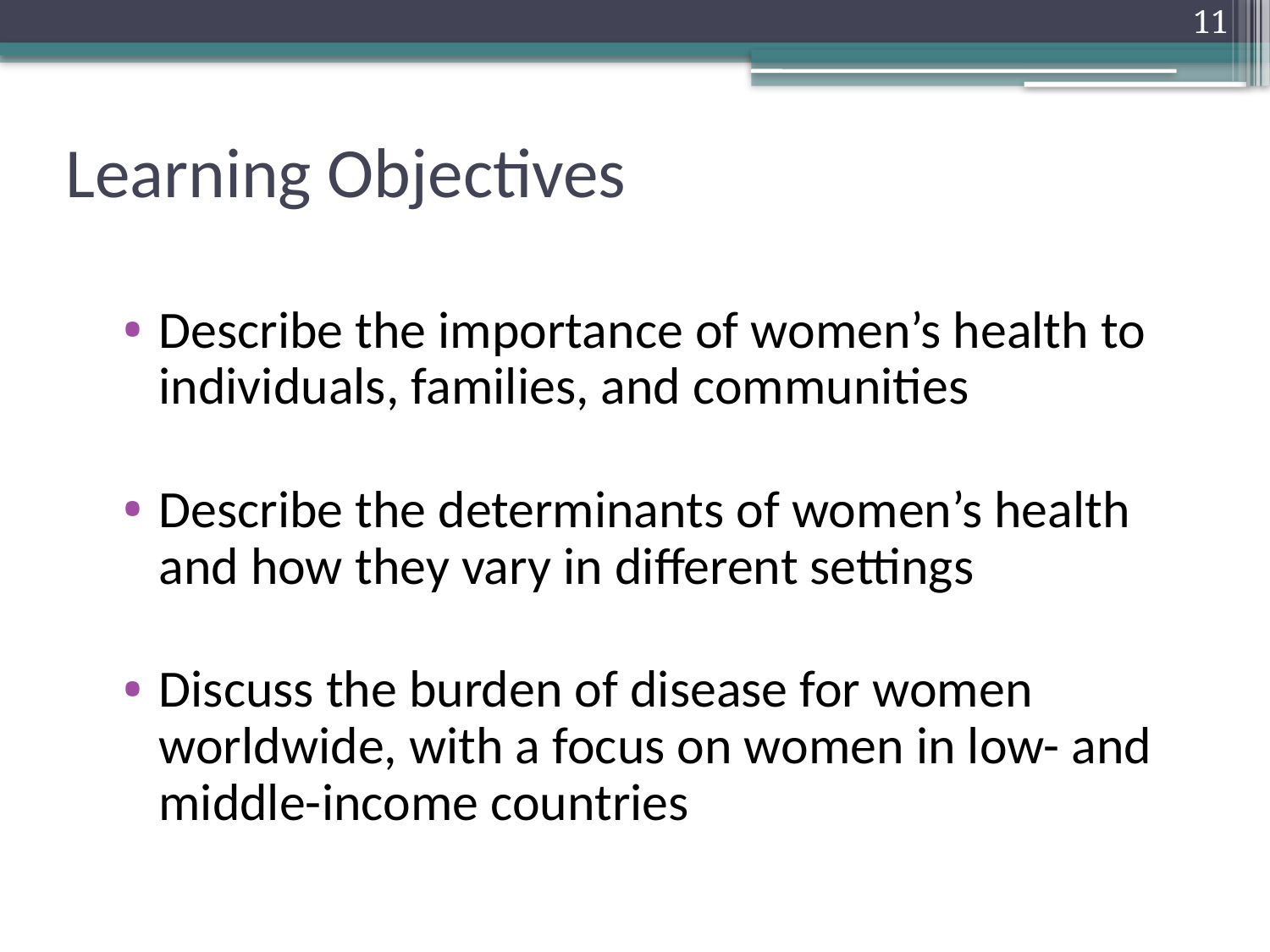

11
# Learning Objectives
Describe the importance of women’s health to individuals, families, and communities
Describe the determinants of women’s health and how they vary in different settings
Discuss the burden of disease for women worldwide, with a focus on women in low- and middle-income countries

## Slide 12
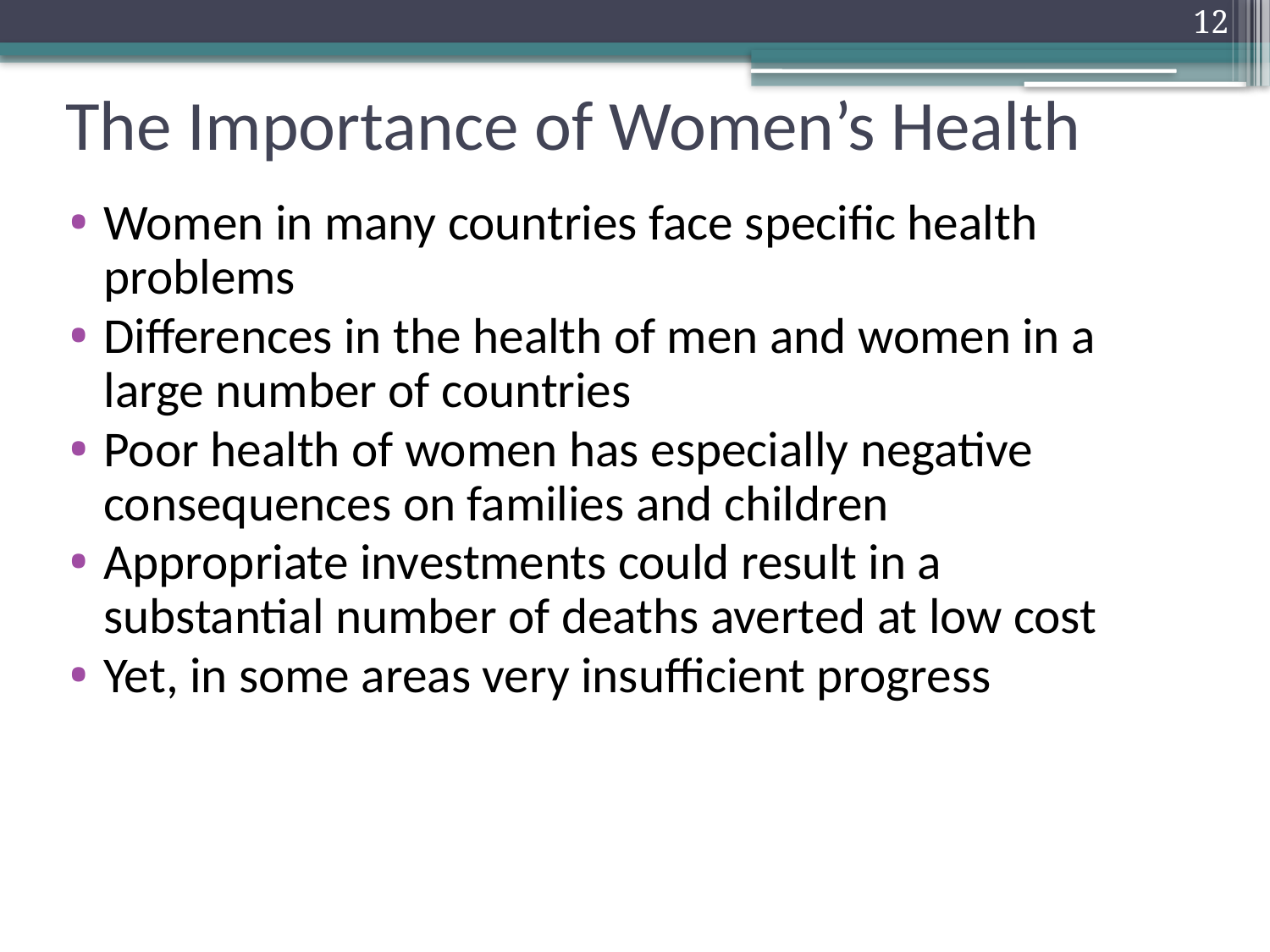

12
# The Importance of Women’s Health
Women in many countries face specific health problems
Differences in the health of men and women in a large number of countries
Poor health of women has especially negative consequences on families and children
Appropriate investments could result in a substantial number of deaths averted at low cost
Yet, in some areas very insufficient progress

## Slide 13
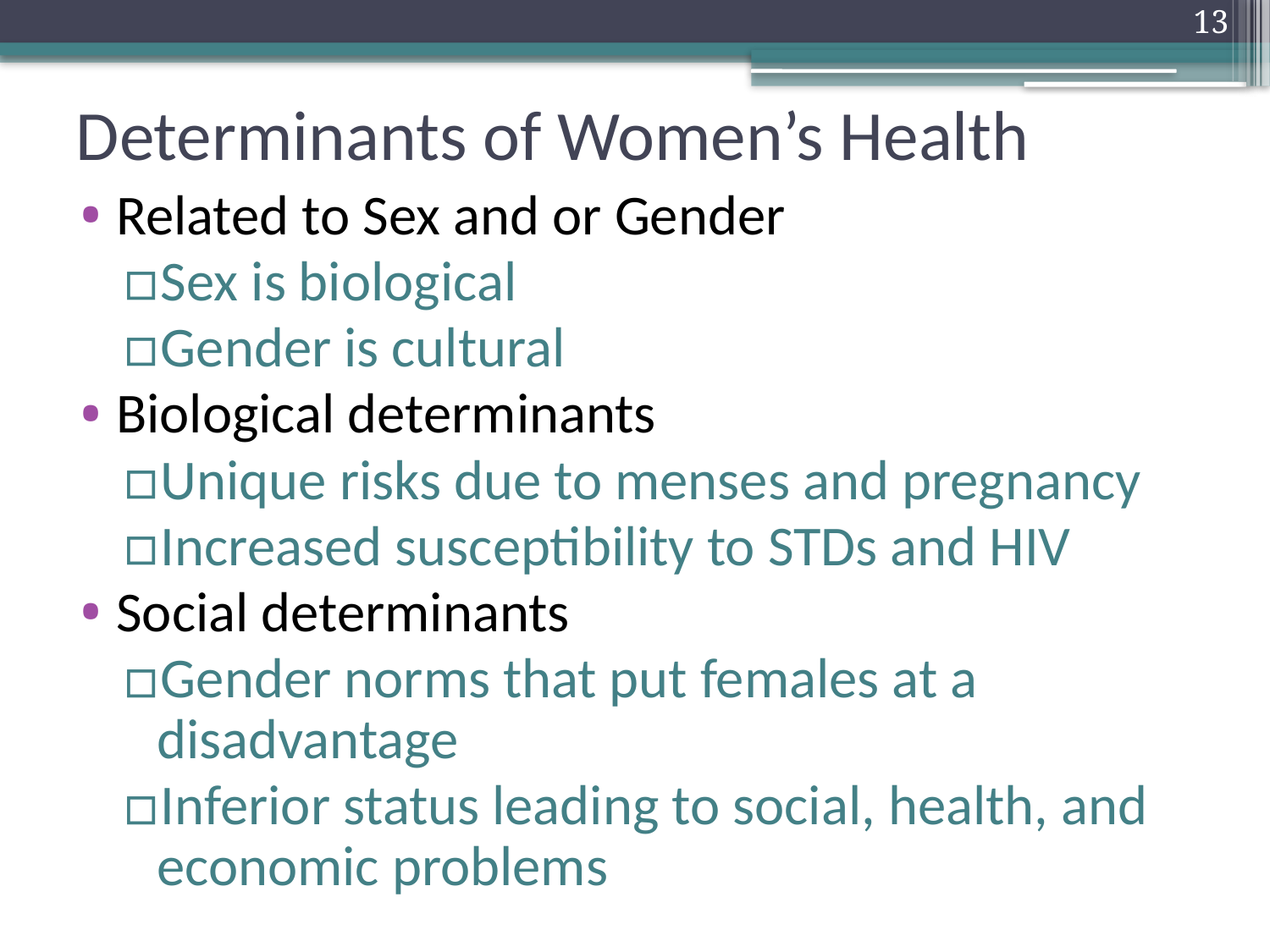

13
# Determinants of Women’s Health
Related to Sex and or Gender
Sex is biological
Gender is cultural
Biological determinants
Unique risks due to menses and pregnancy
Increased susceptibility to STDs and HIV
Social determinants
Gender norms that put females at a disadvantage
Inferior status leading to social, health, and economic problems

## Slide 14
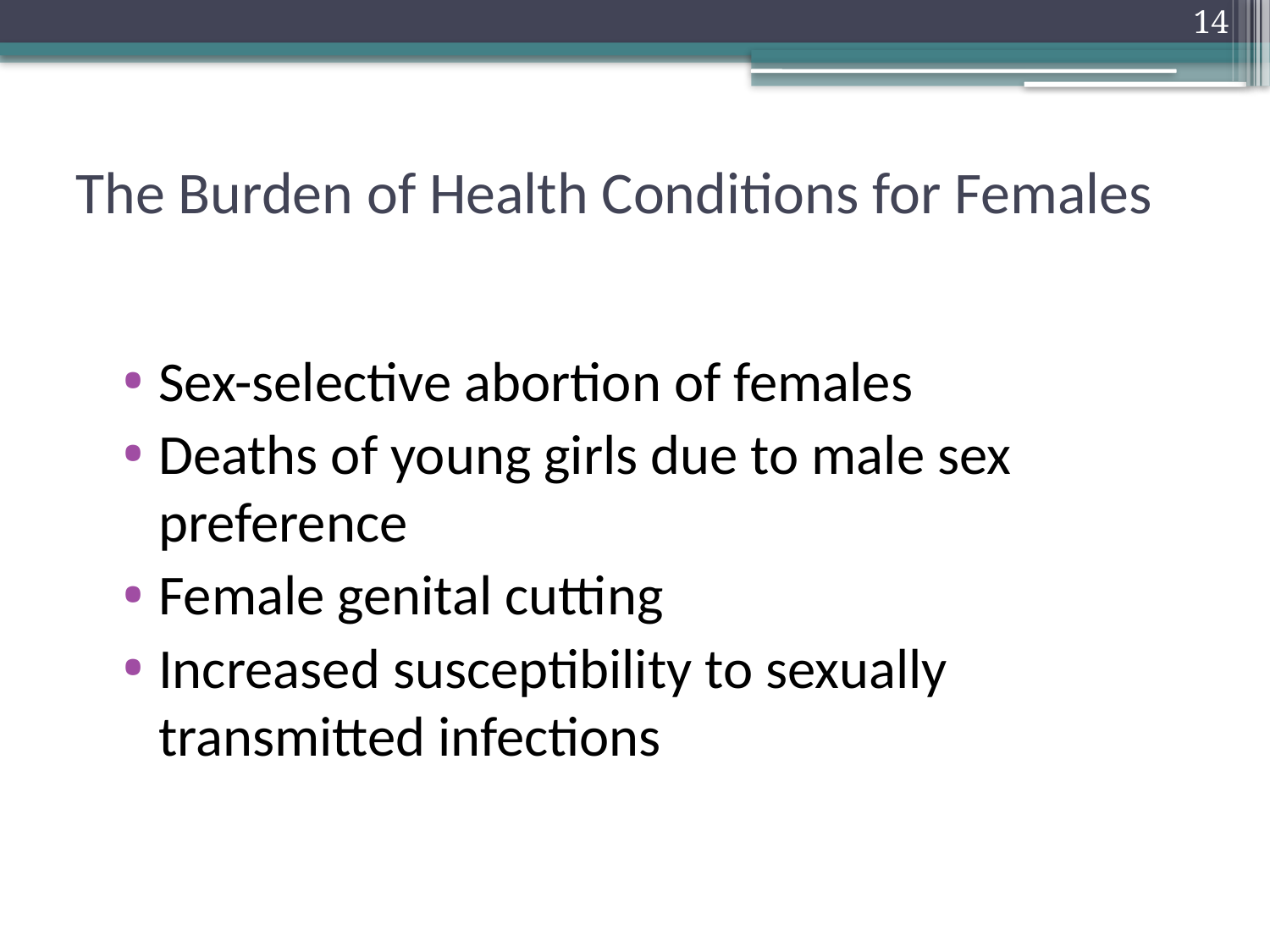

14
# The Burden of Health Conditions for Females
Sex-selective abortion of females
Deaths of young girls due to male sex preference
Female genital cutting
Increased susceptibility to sexually transmitted infections

## Slide 15
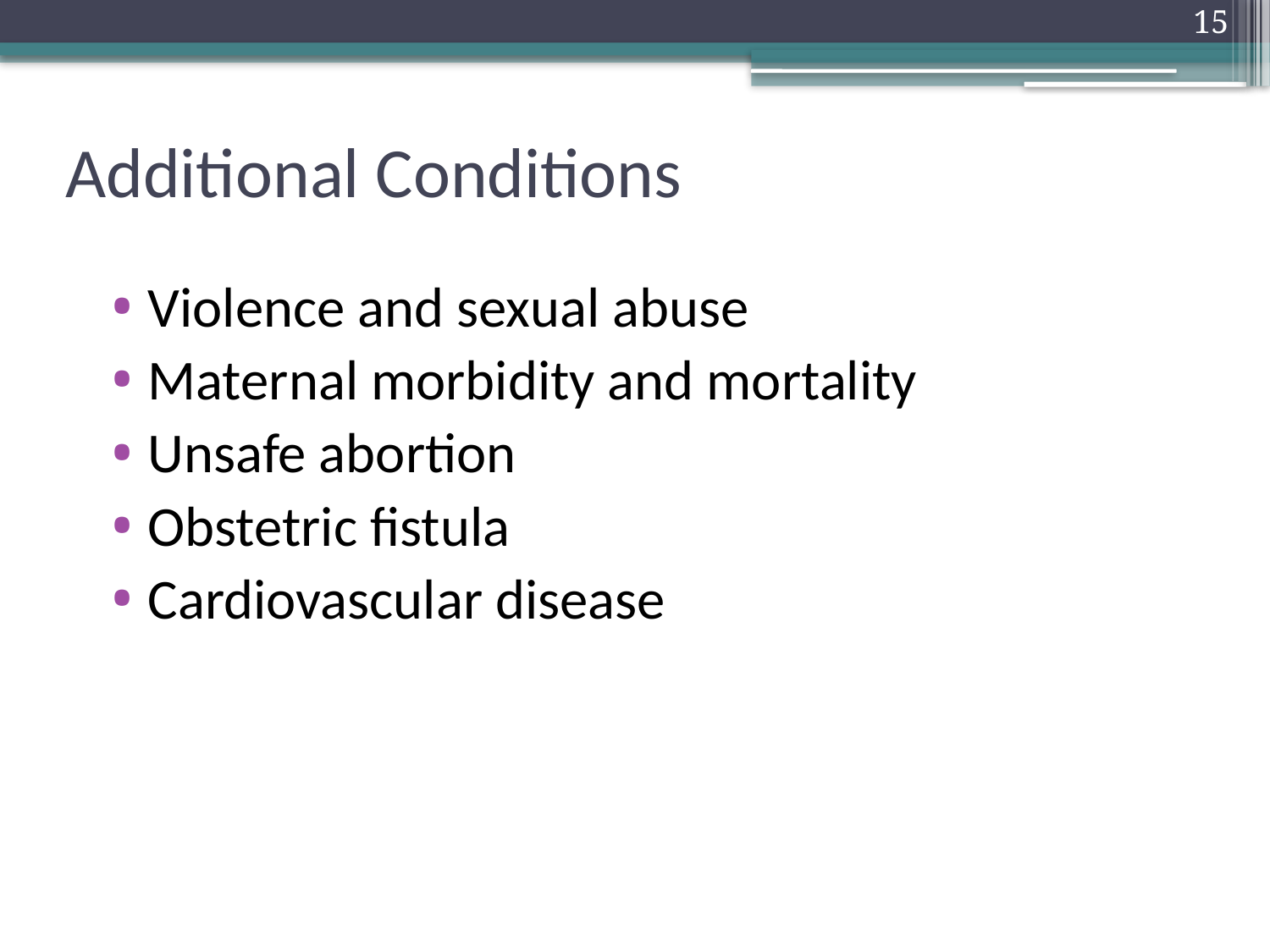

15
# Additional Conditions
Violence and sexual abuse
Maternal morbidity and mortality
Unsafe abortion
Obstetric fistula
Cardiovascular disease

## Slide 16
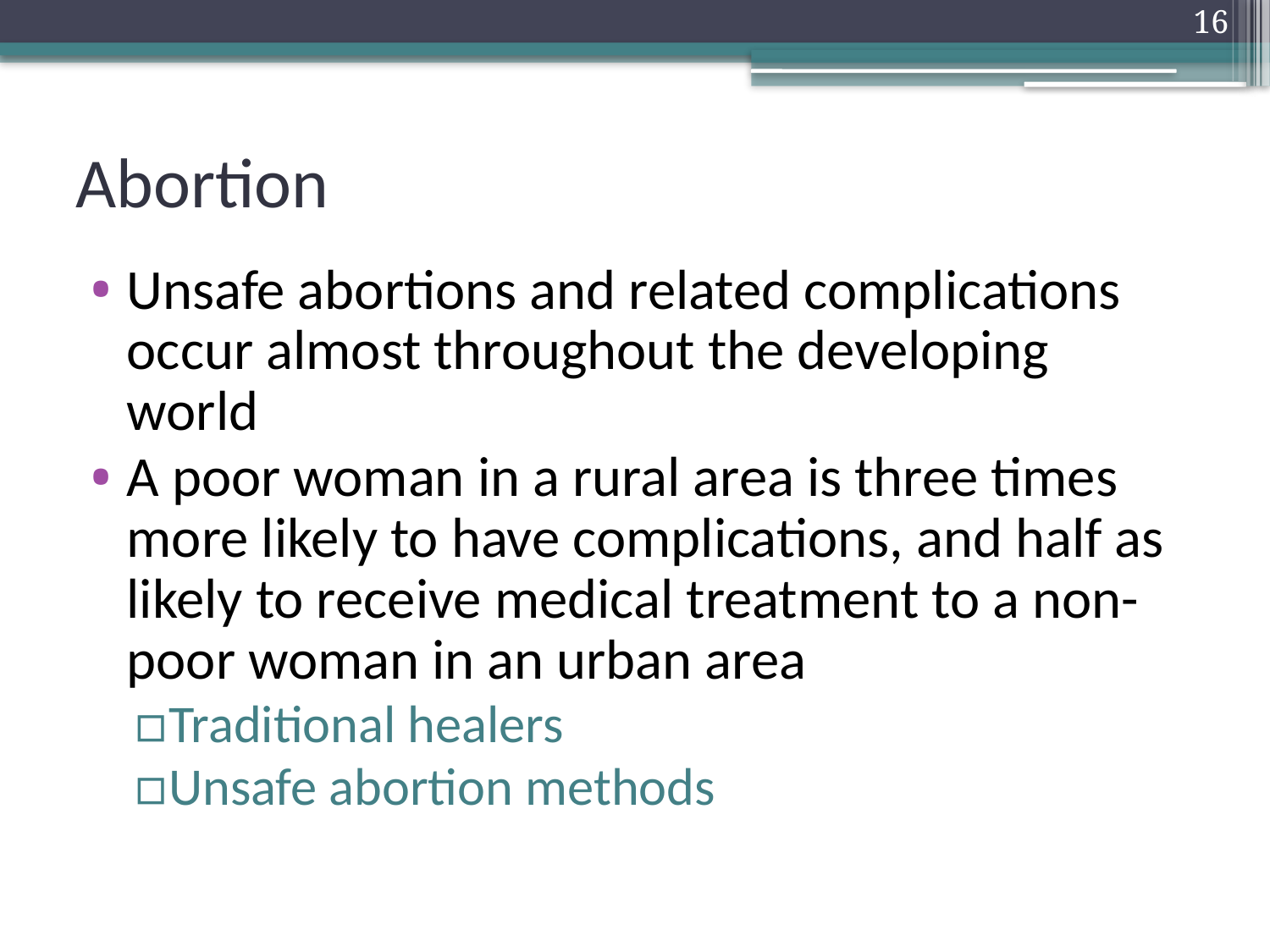

16
# Abortion
Unsafe abortions and related complications occur almost throughout the developing world
A poor woman in a rural area is three times more likely to have complications, and half as likely to receive medical treatment to a non-poor woman in an urban area
Traditional healers
Unsafe abortion methods

## Slide 17
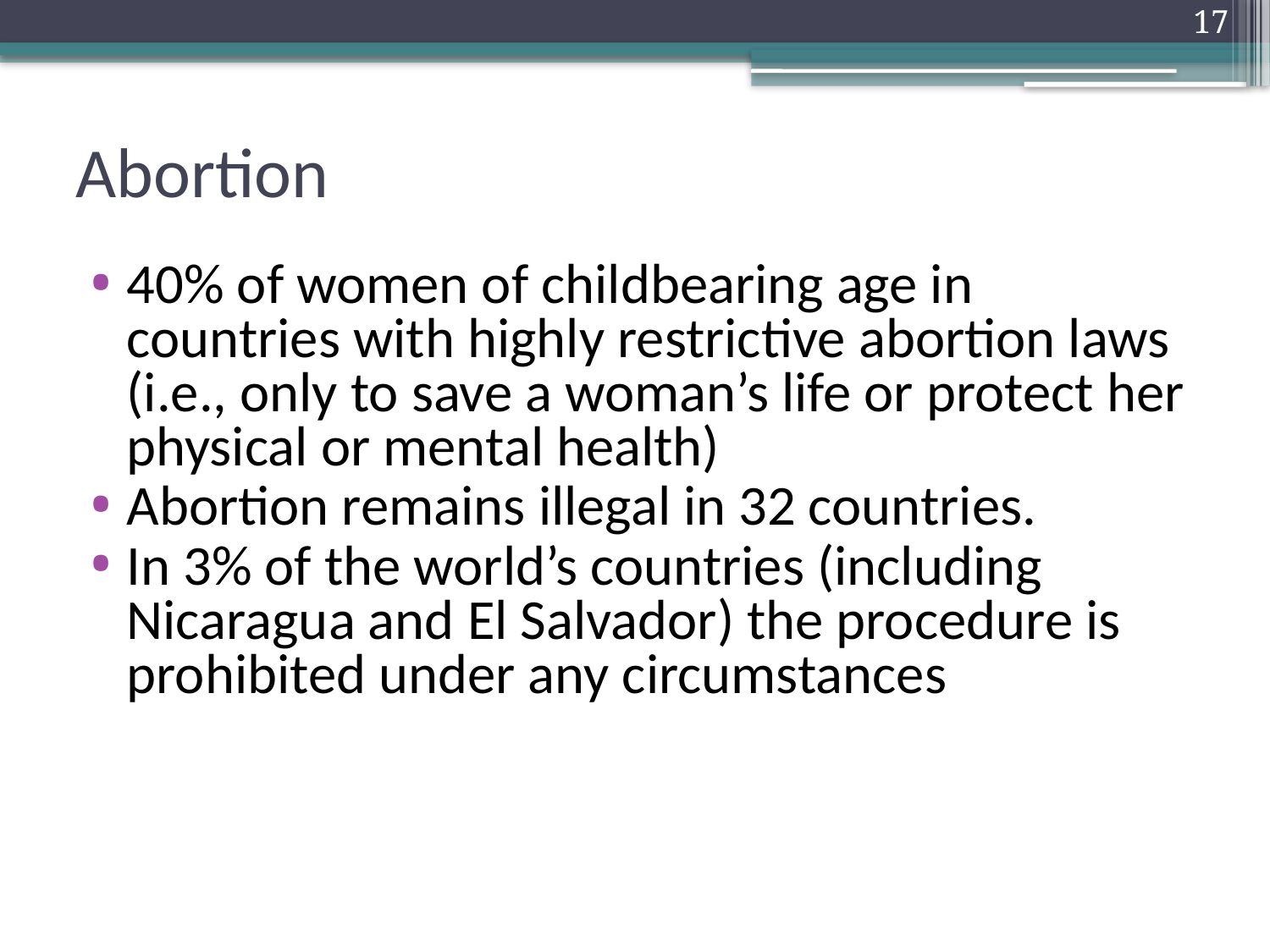

17
# Abortion
40% of women of childbearing age in countries with highly restrictive abortion laws (i.e., only to save a woman’s life or protect her physical or mental health)
Abortion remains illegal in 32 countries.
In 3% of the world’s countries (including Nicaragua and El Salvador) the procedure is prohibited under any circumstances

## Slide 18
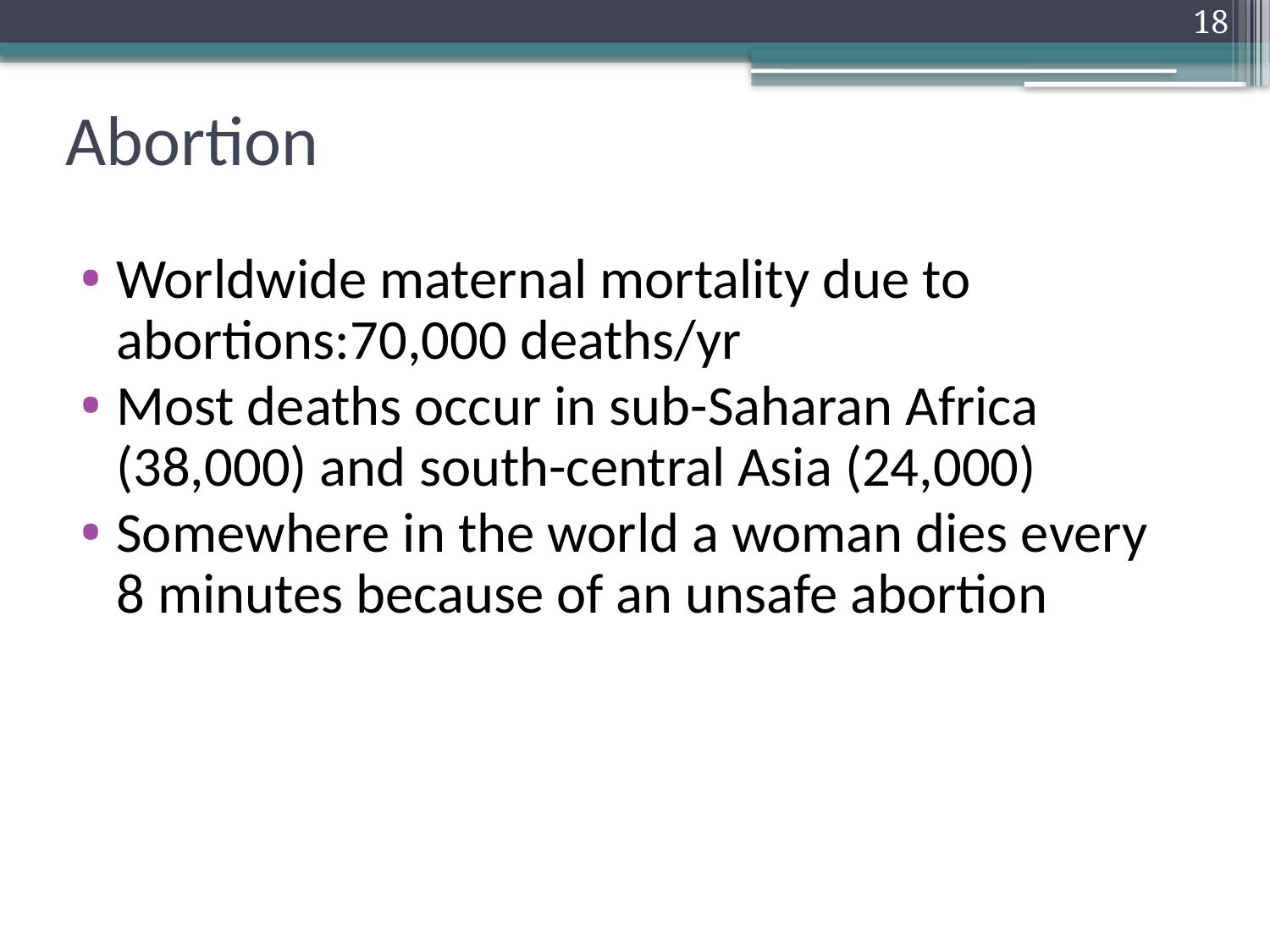

18
# Abortion
Worldwide maternal mortality due to abortions:70,000 deaths/yr
Most deaths occur in sub-Saharan Africa (38,000) and south-central Asia (24,000)
Somewhere in the world a woman dies every 8 minutes because of an unsafe abortion

## Slide 19
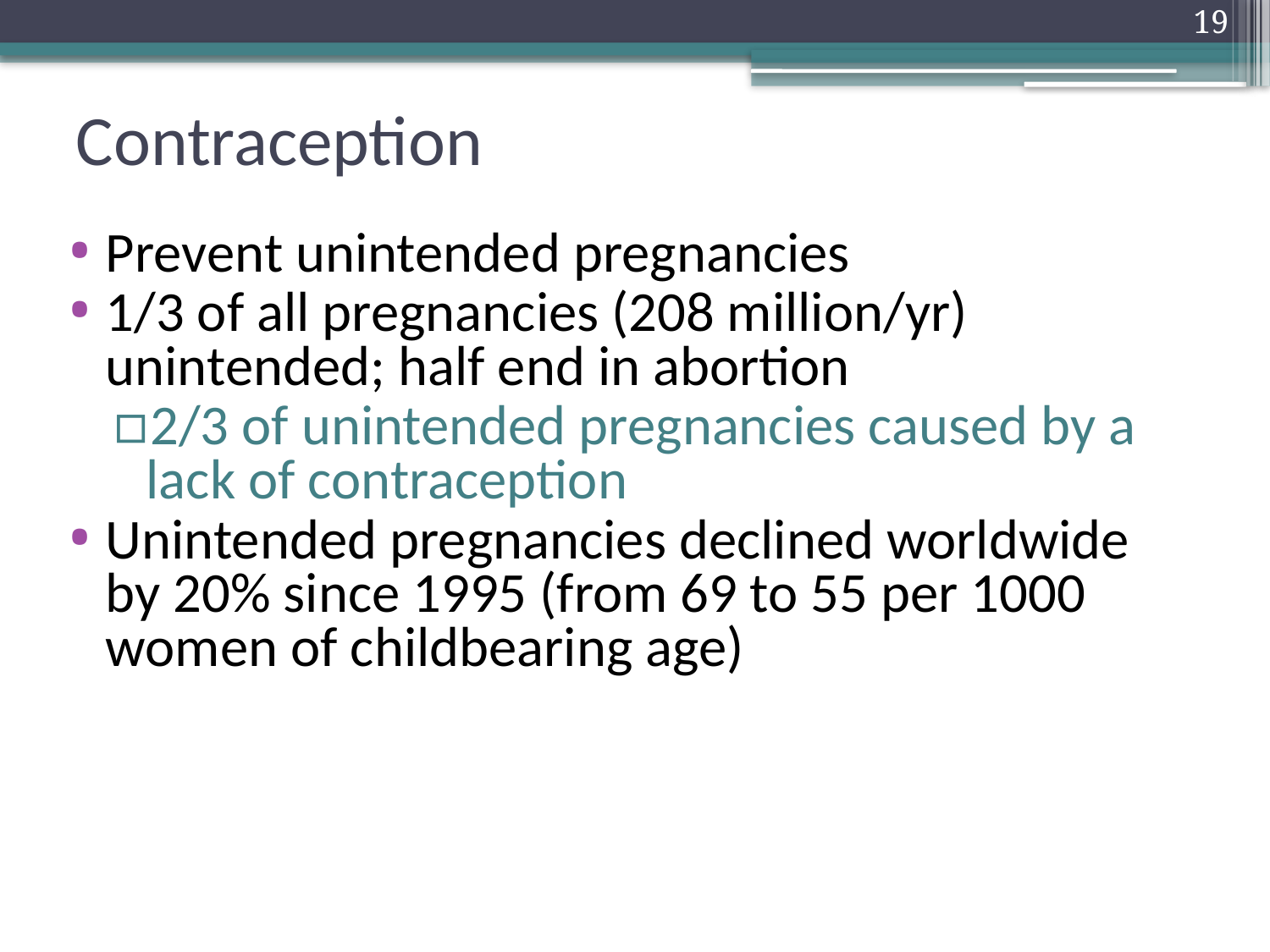

19
# Contraception
Prevent unintended pregnancies
1/3 of all pregnancies (208 million/yr) unintended; half end in abortion
2/3 of unintended pregnancies caused by a lack of contraception
Unintended pregnancies declined worldwide by 20% since 1995 (from 69 to 55 per 1000 women of childbearing age)

## Slide 20
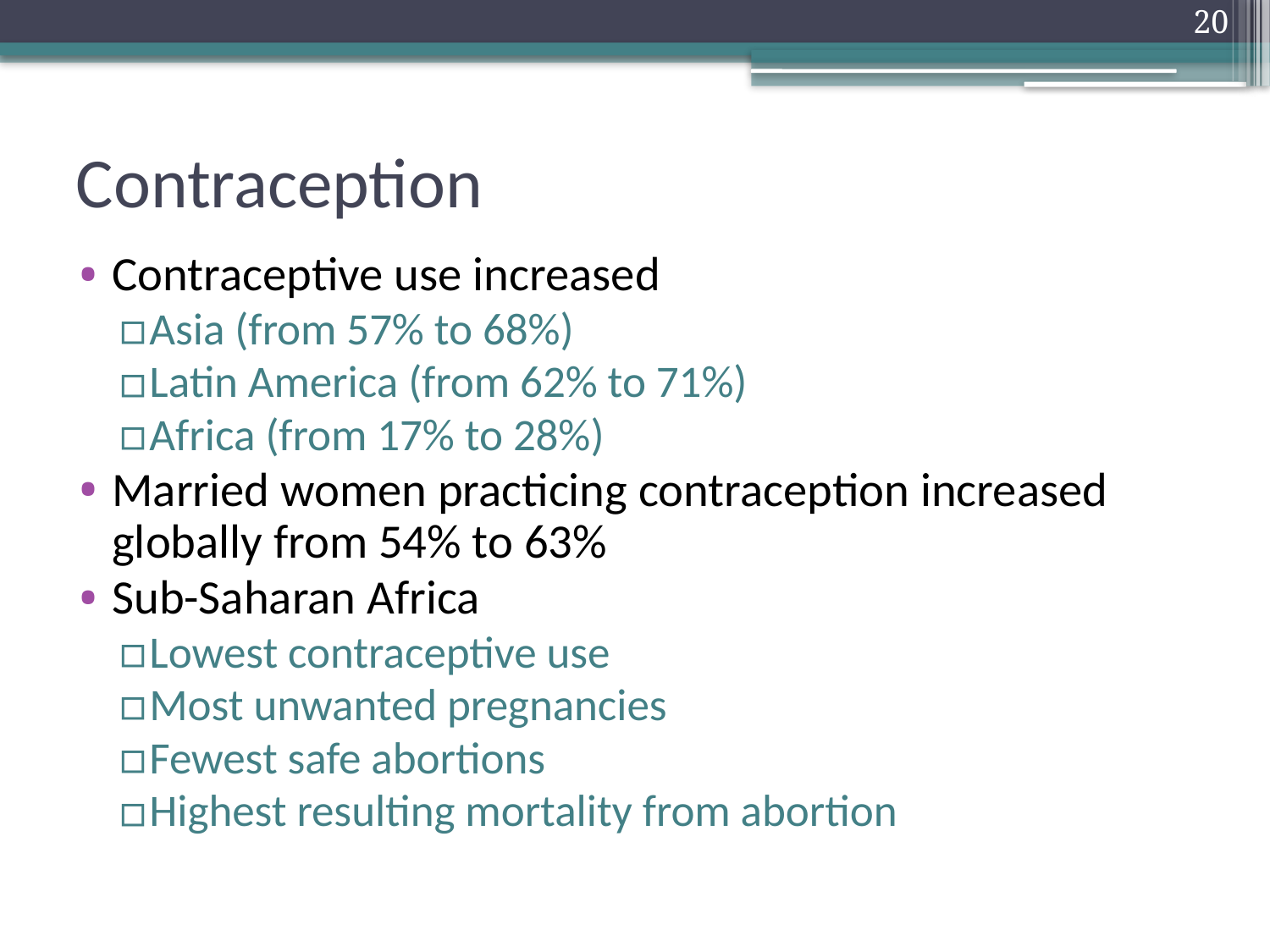

20
# Contraception
Contraceptive use increased
Asia (from 57% to 68%)
Latin America (from 62% to 71%)
Africa (from 17% to 28%)
Married women practicing contraception increased globally from 54% to 63%
Sub-Saharan Africa
Lowest contraceptive use
Most unwanted pregnancies
Fewest safe abortions
Highest resulting mortality from abortion

## Slide 21
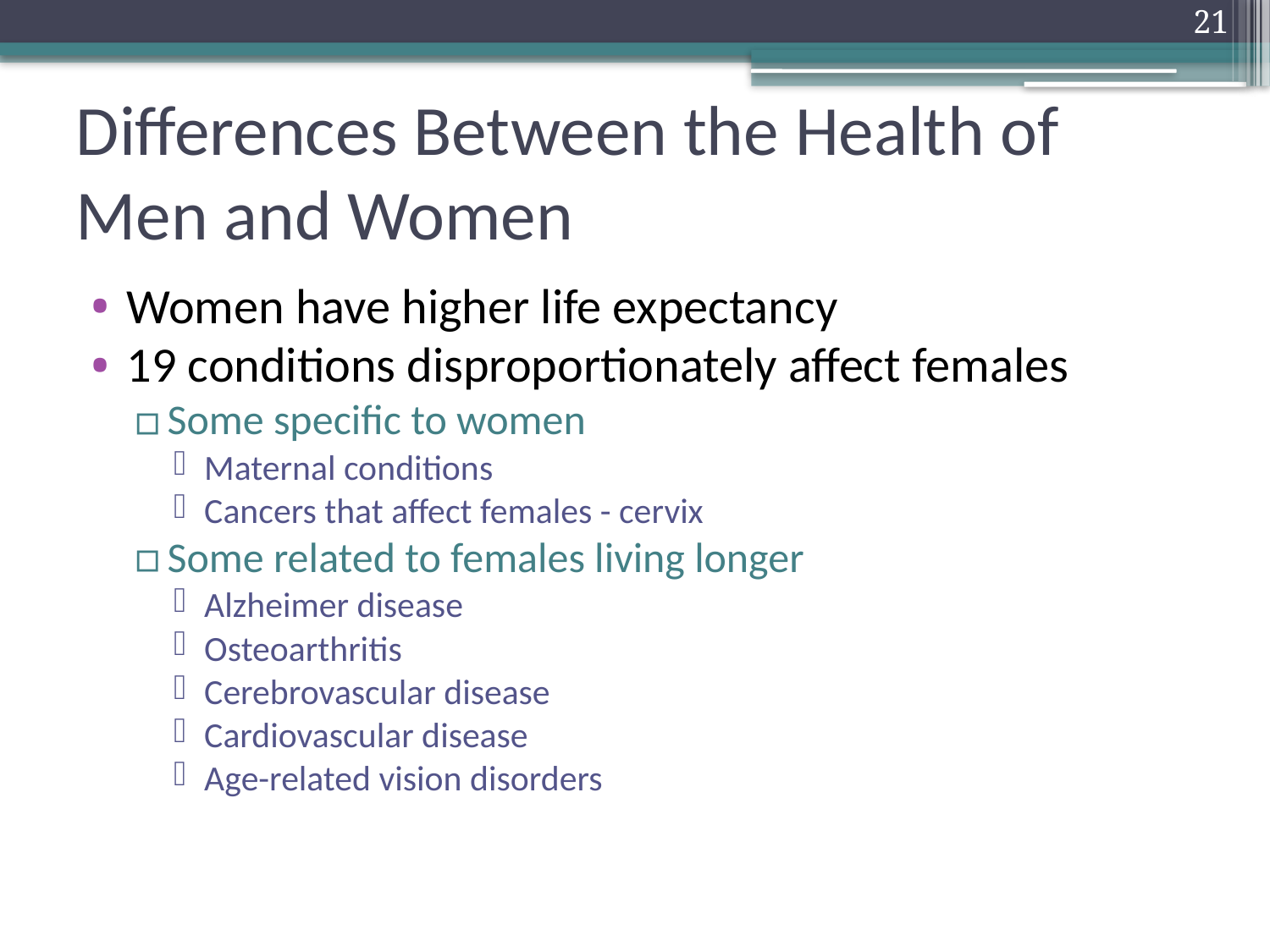

21
# Differences Between the Health of Men and Women
Women have higher life expectancy
19 conditions disproportionately affect females
Some specific to women
Maternal conditions
Cancers that affect females - cervix
Some related to females living longer
Alzheimer disease
Osteoarthritis
Cerebrovascular disease
Cardiovascular disease
Age-related vision disorders

## Slide 22
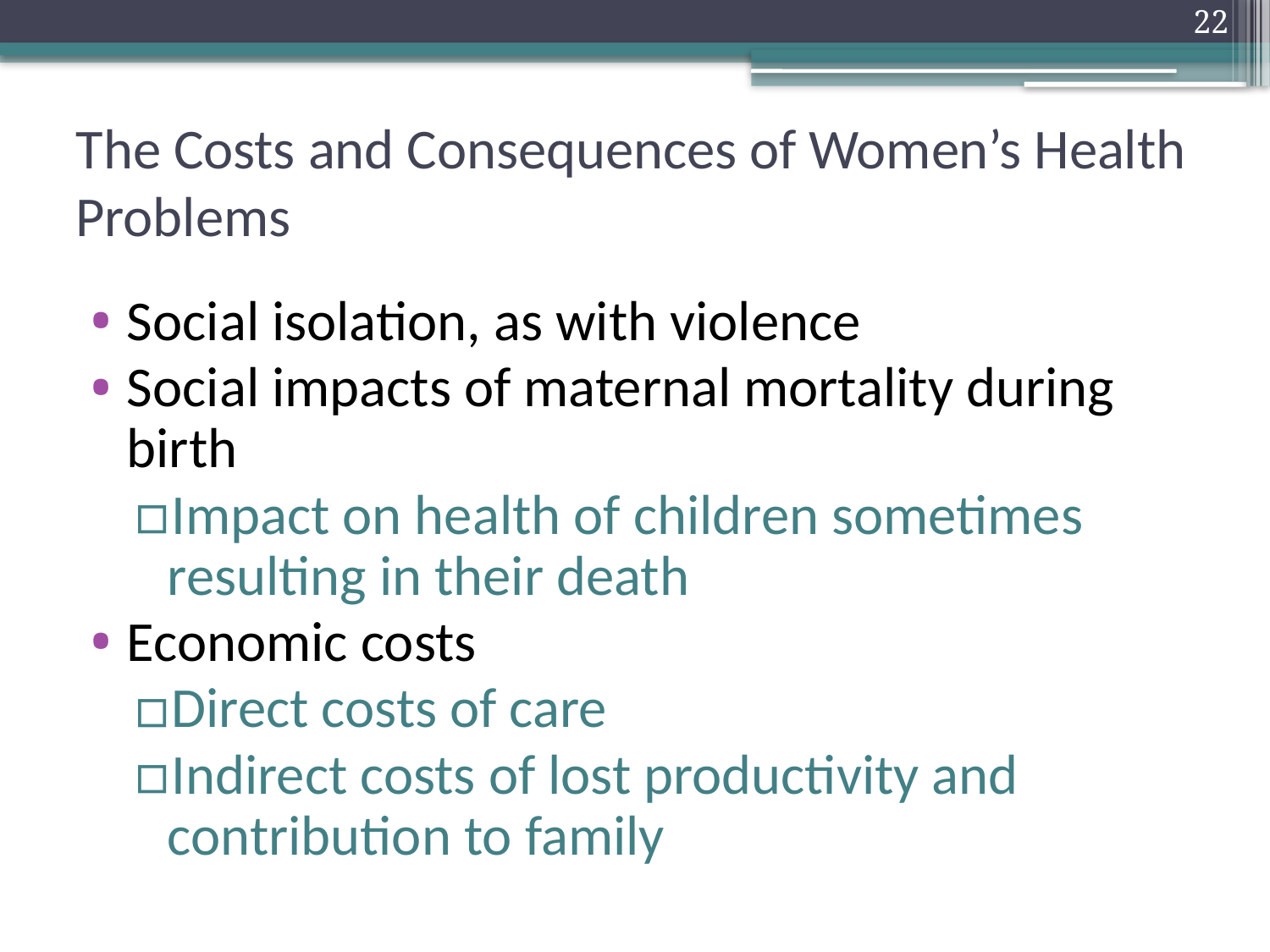

22
# The Costs and Consequences of Women’s Health Problems
Social isolation, as with violence
Social impacts of maternal mortality during birth
Impact on health of children sometimes resulting in their death
Economic costs
Direct costs of care
Indirect costs of lost productivity and contribution to family

## Slide 23
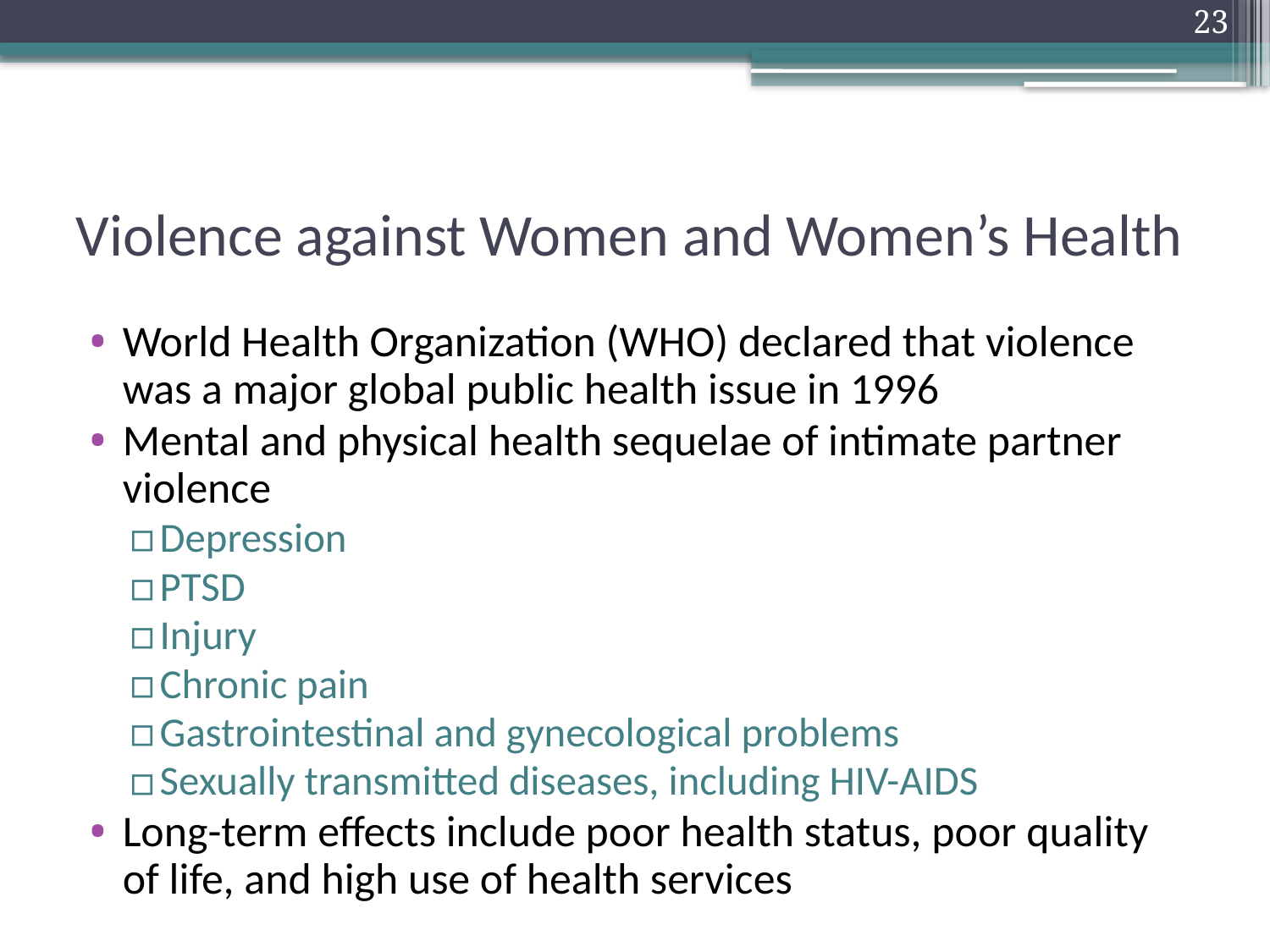

23
# Violence against Women and Women’s Health
World Health Organization (WHO) declared that violence was a major global public health issue in 1996
Mental and physical health sequelae of intimate partner violence
Depression
PTSD
Injury
Chronic pain
Gastrointestinal and gynecological problems
Sexually transmitted diseases, including HIV-AIDS
Long-term effects include poor health status, poor quality of life, and high use of health services

## Slide 24
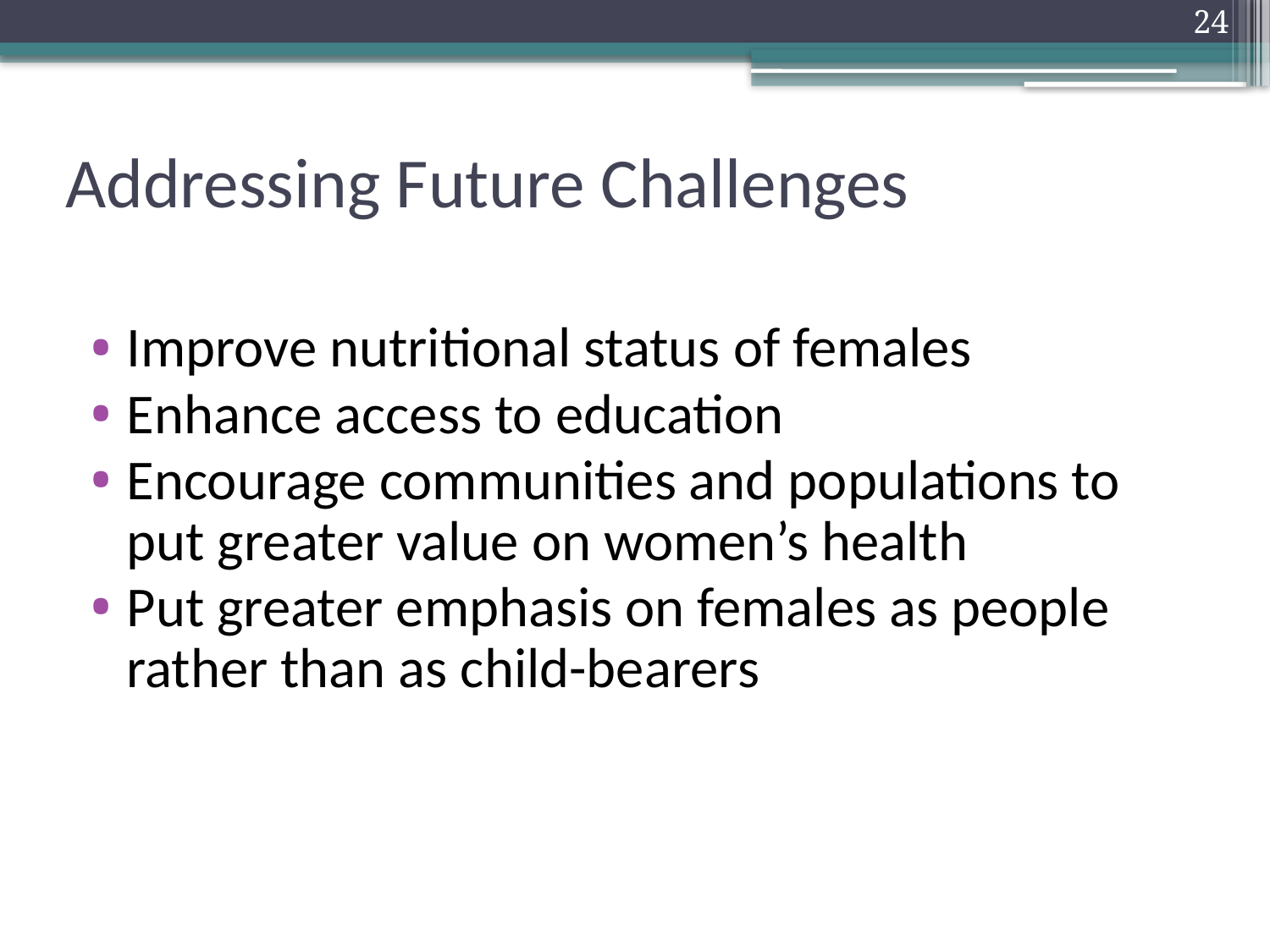

24
# Addressing Future Challenges
Improve nutritional status of females
Enhance access to education
Encourage communities and populations to put greater value on women’s health
Put greater emphasis on females as people rather than as child-bearers

## Slide 25
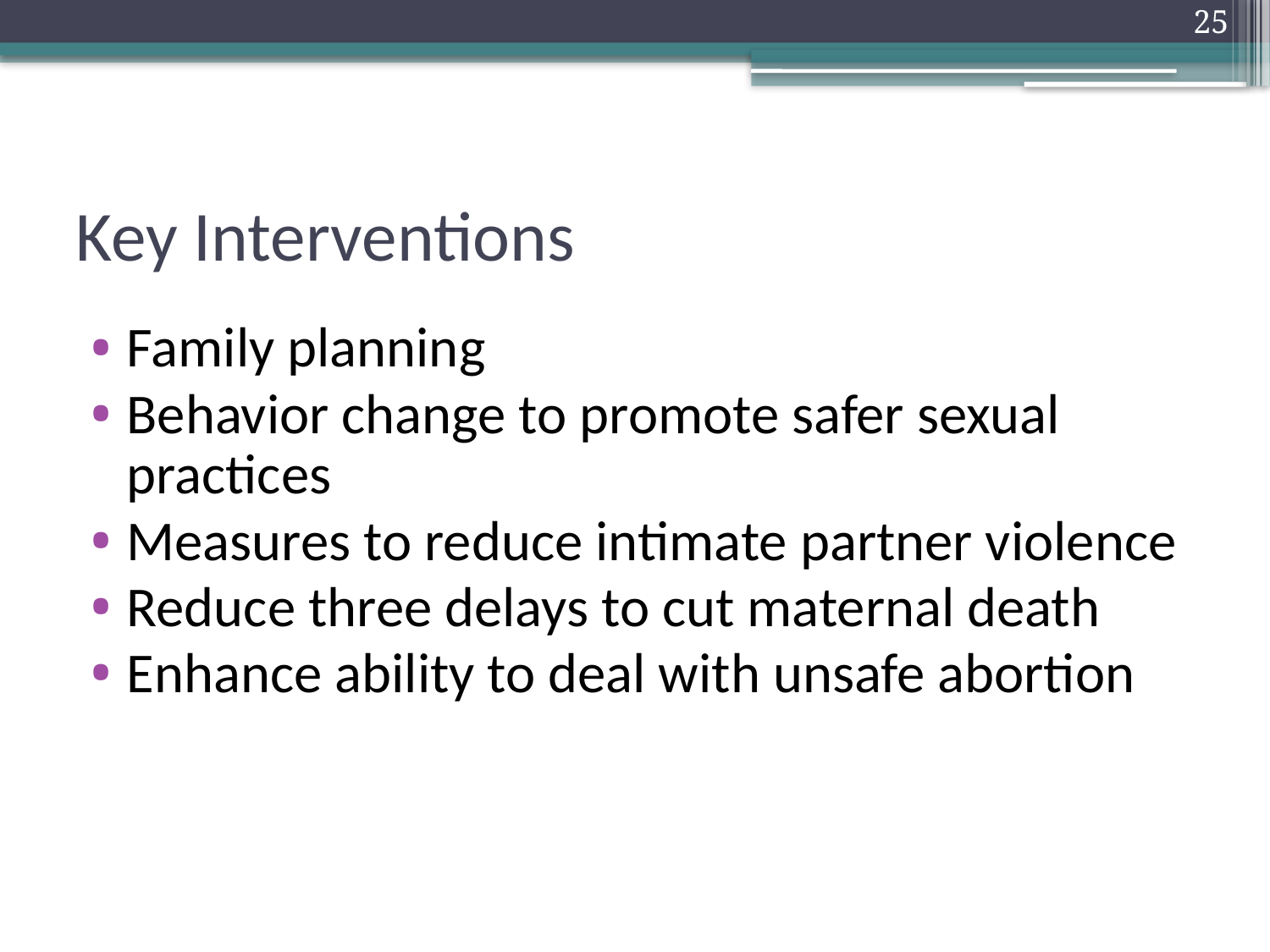

25
# Key Interventions
Family planning
Behavior change to promote safer sexual practices
Measures to reduce intimate partner violence
Reduce three delays to cut maternal death
Enhance ability to deal with unsafe abortion

## Slide 26
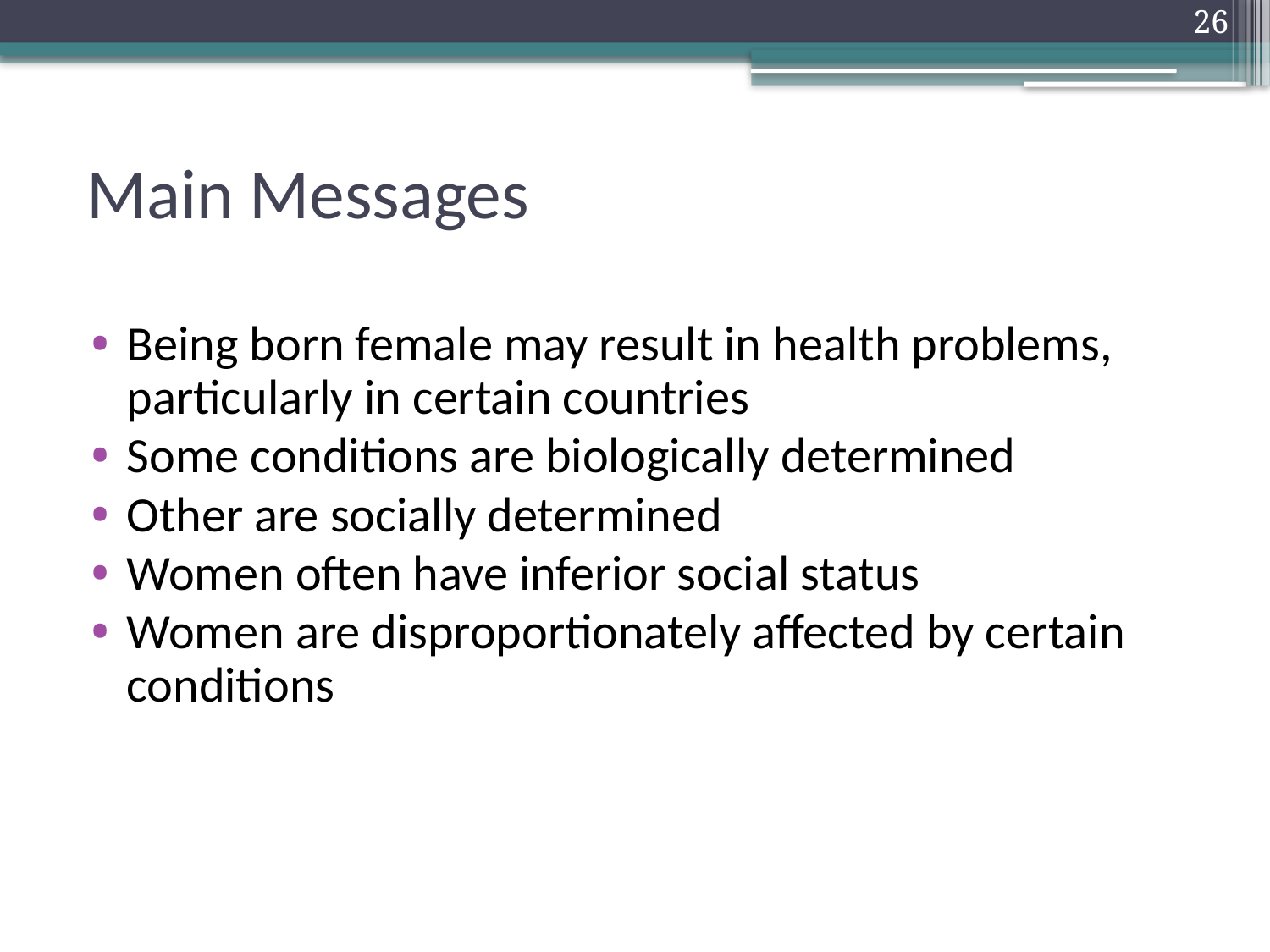

26
# Main Messages
Being born female may result in health problems, particularly in certain countries
Some conditions are biologically determined
Other are socially determined
Women often have inferior social status
Women are disproportionately affected by certain conditions

## Slide 27
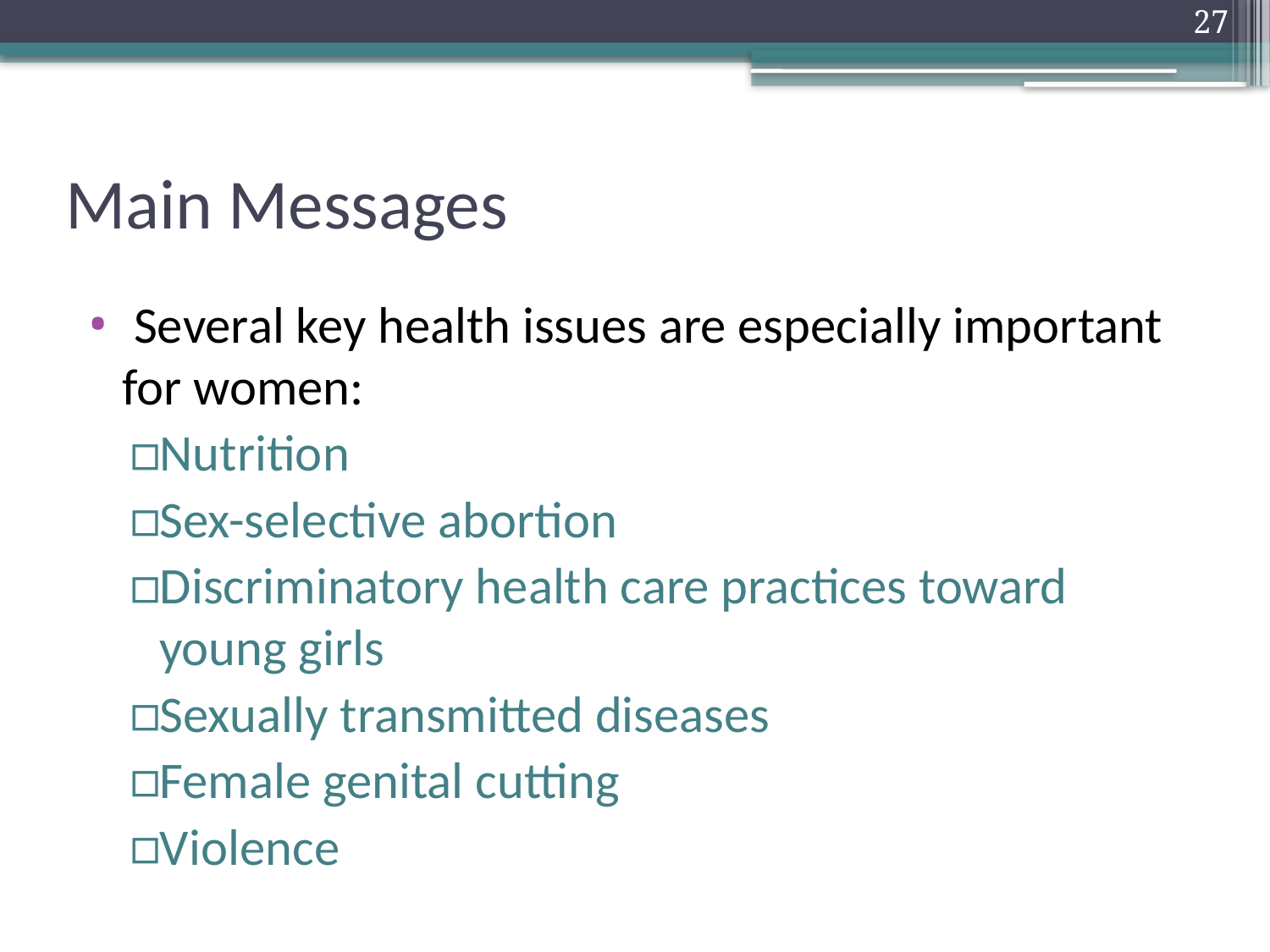

27
# Main Messages
 Several key health issues are especially important for women:
Nutrition
Sex-selective abortion
Discriminatory health care practices toward young girls
Sexually transmitted diseases
Female genital cutting
Violence

## Slide 28
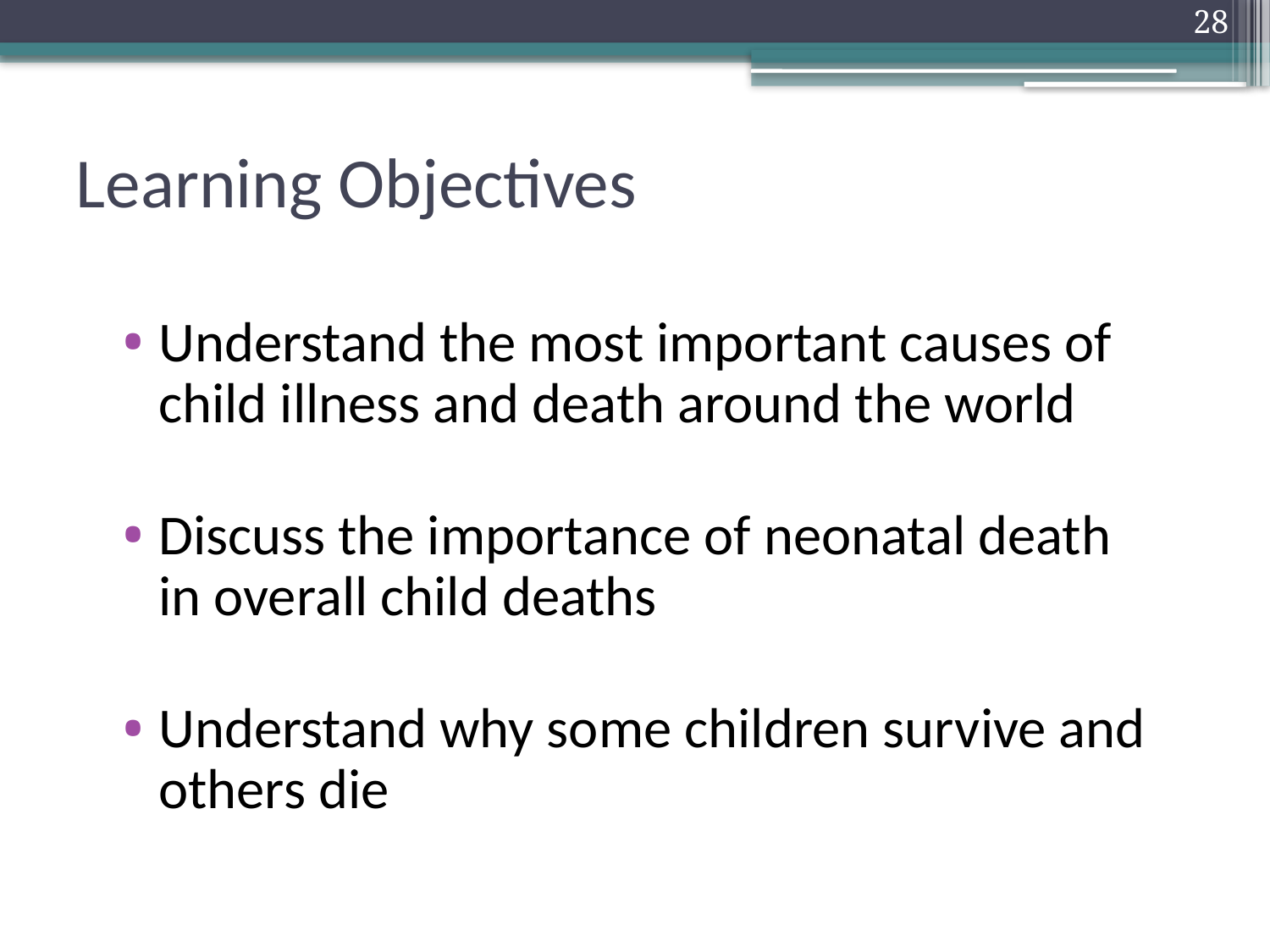

28
# Learning Objectives
Understand the most important causes of child illness and death around the world
Discuss the importance of neonatal death in overall child deaths
Understand why some children survive and others die

## Slide 29
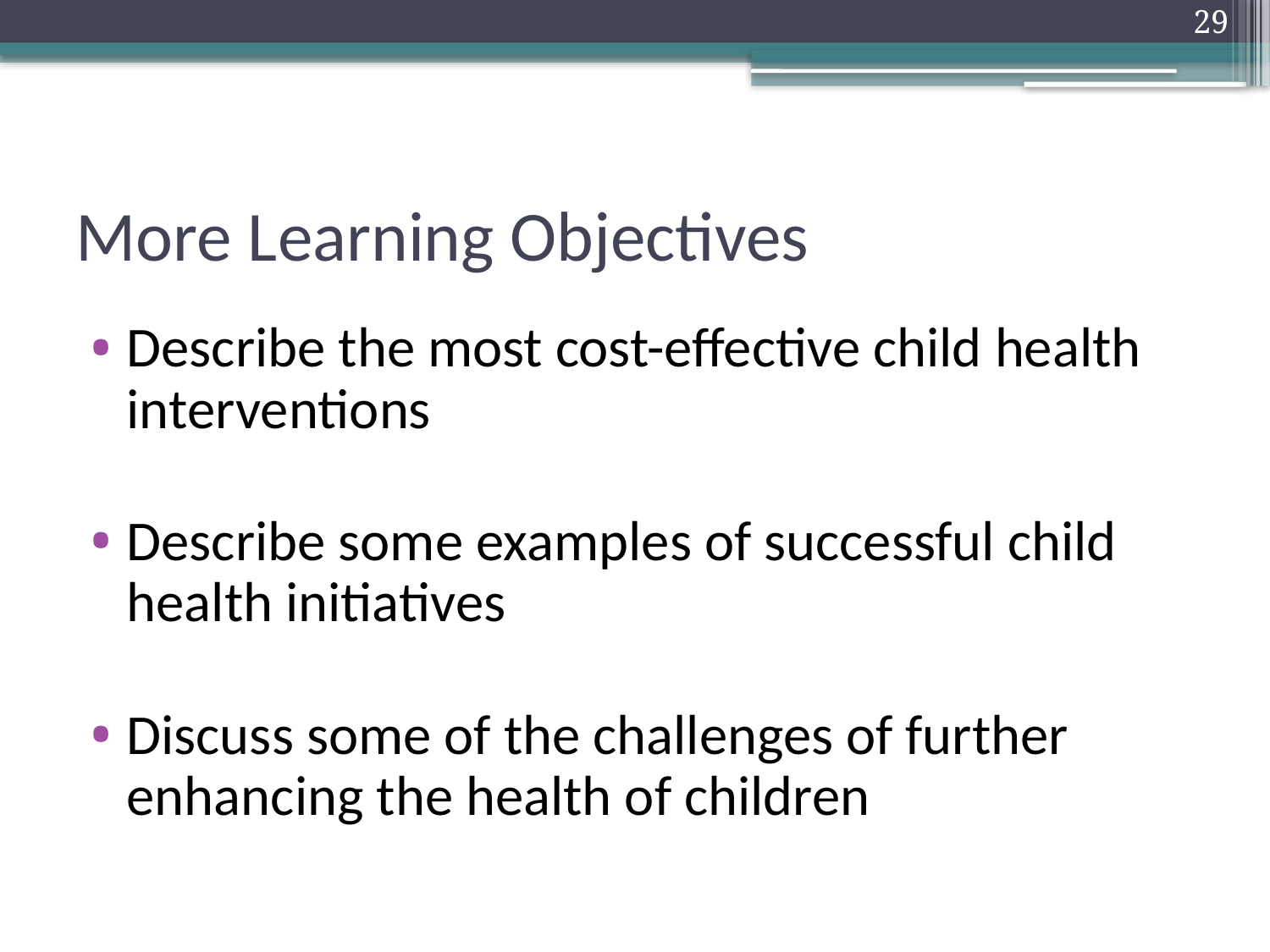

29
# More Learning Objectives
Describe the most cost-effective child health interventions
Describe some examples of successful child health initiatives
Discuss some of the challenges of further enhancing the health of children

## Slide 30
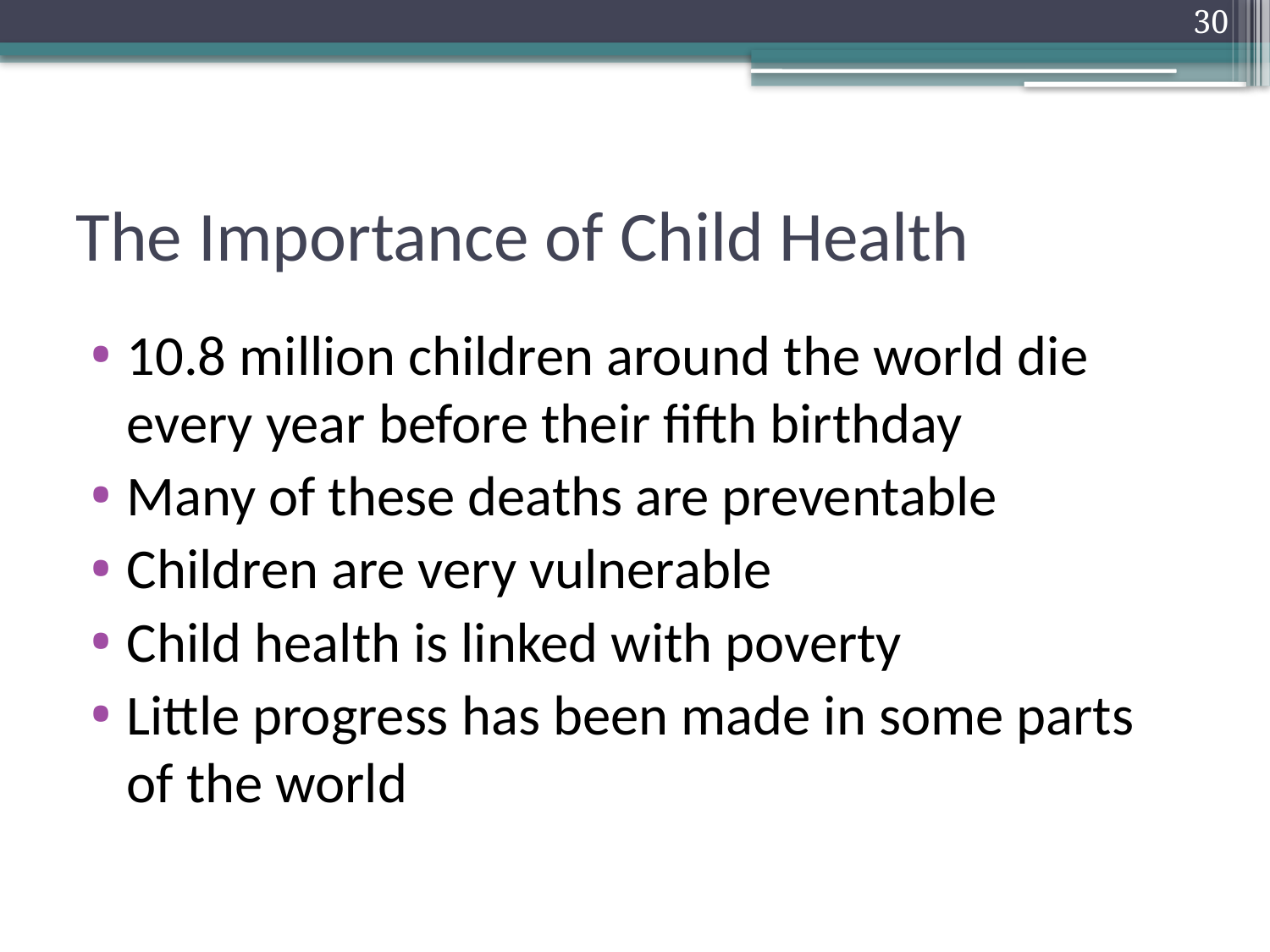

30
# The Importance of Child Health
10.8 million children around the world die every year before their fifth birthday
Many of these deaths are preventable
Children are very vulnerable
Child health is linked with poverty
Little progress has been made in some parts of the world

## Slide 31
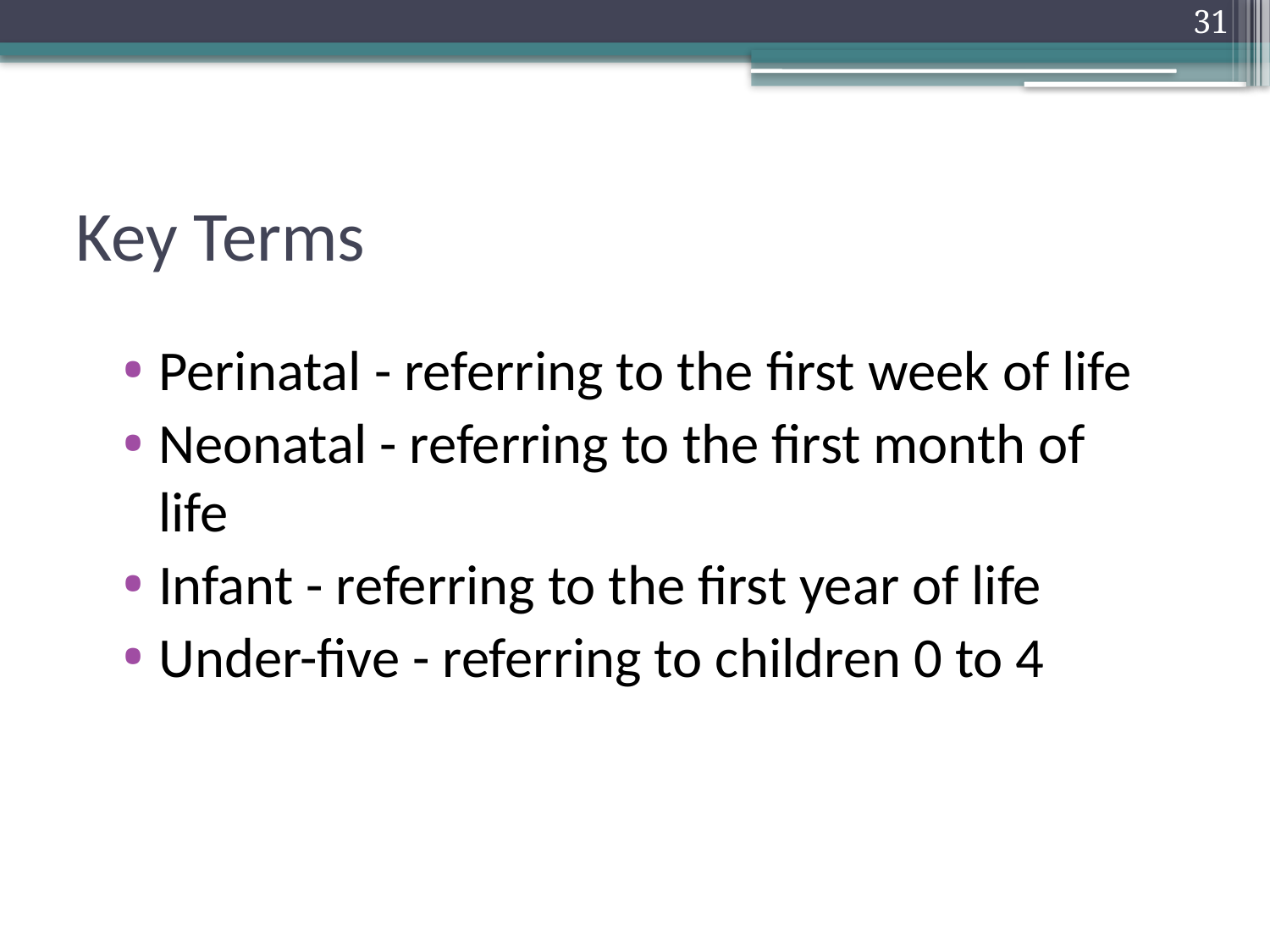

31
# Key Terms
Perinatal - referring to the first week of life
Neonatal - referring to the first month of life
Infant - referring to the first year of life
Under-five - referring to children 0 to 4

## Slide 32
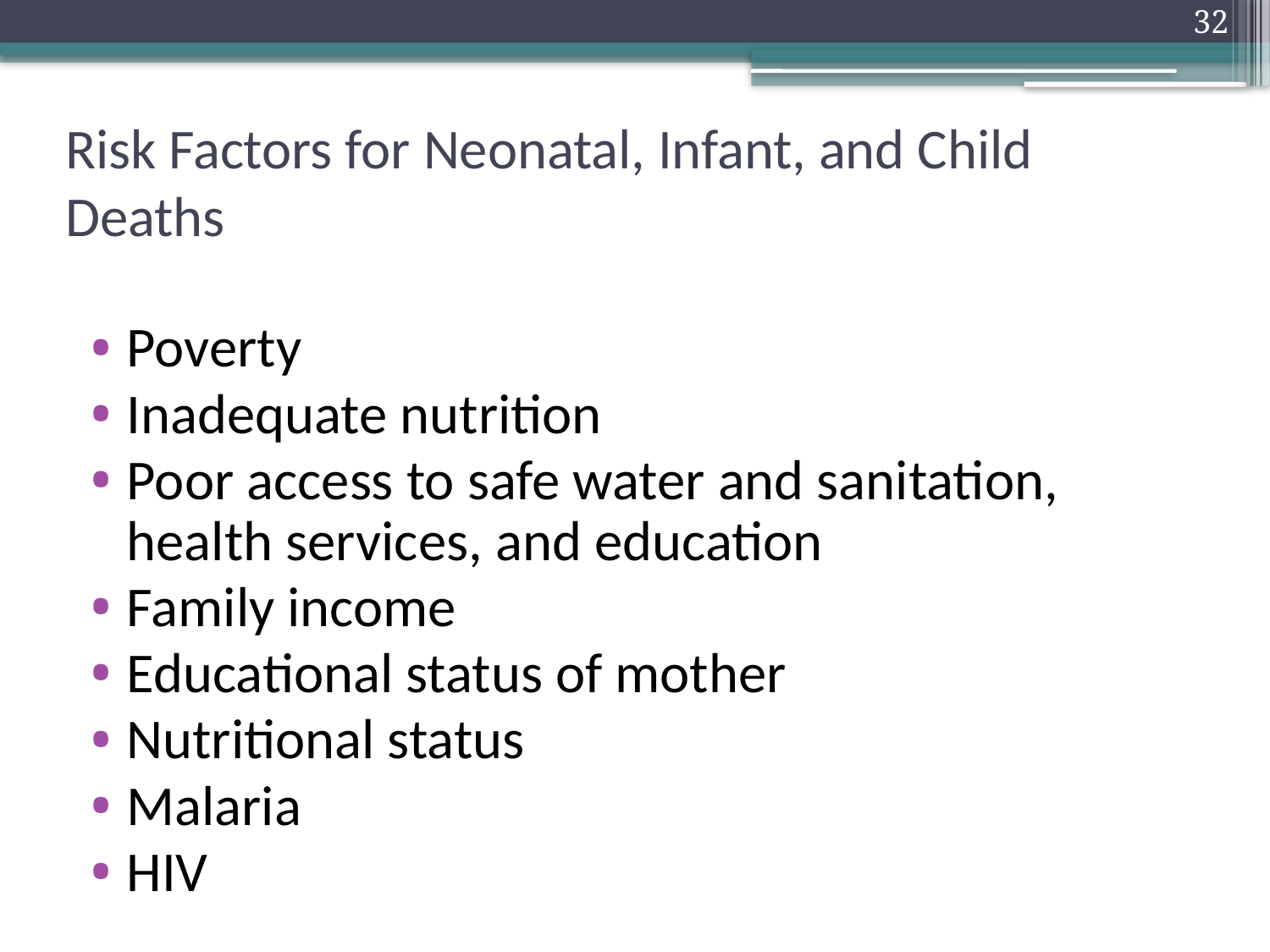

32
# Risk Factors for Neonatal, Infant, and Child Deaths
Poverty
Inadequate nutrition
Poor access to safe water and sanitation, health services, and education
Family income
Educational status of mother
Nutritional status
Malaria
HIV

## Slide 33
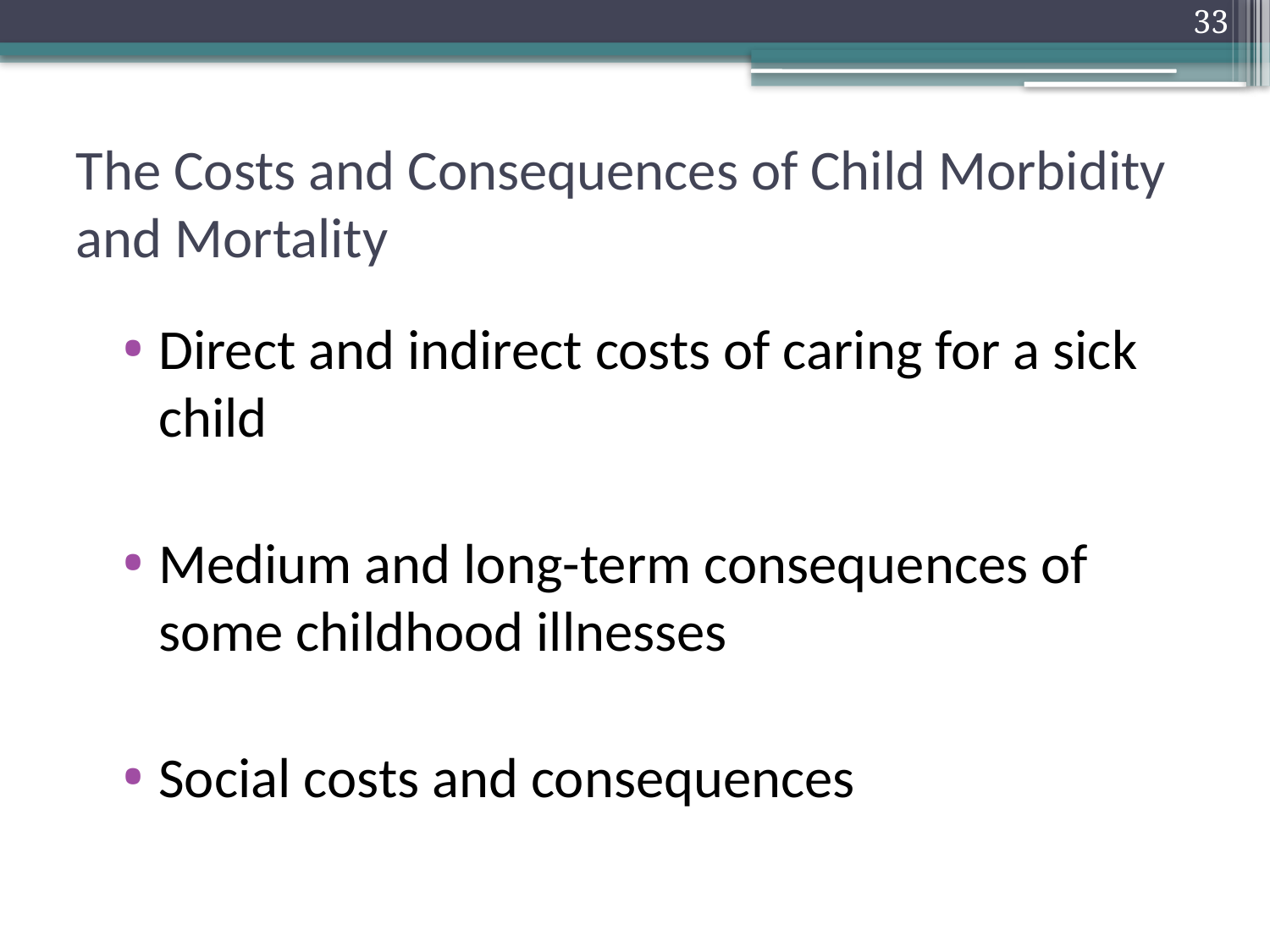

33
# The Costs and Consequences of Child Morbidity and Mortality
Direct and indirect costs of caring for a sick child
Medium and long-term consequences of some childhood illnesses
Social costs and consequences

## Slide 34
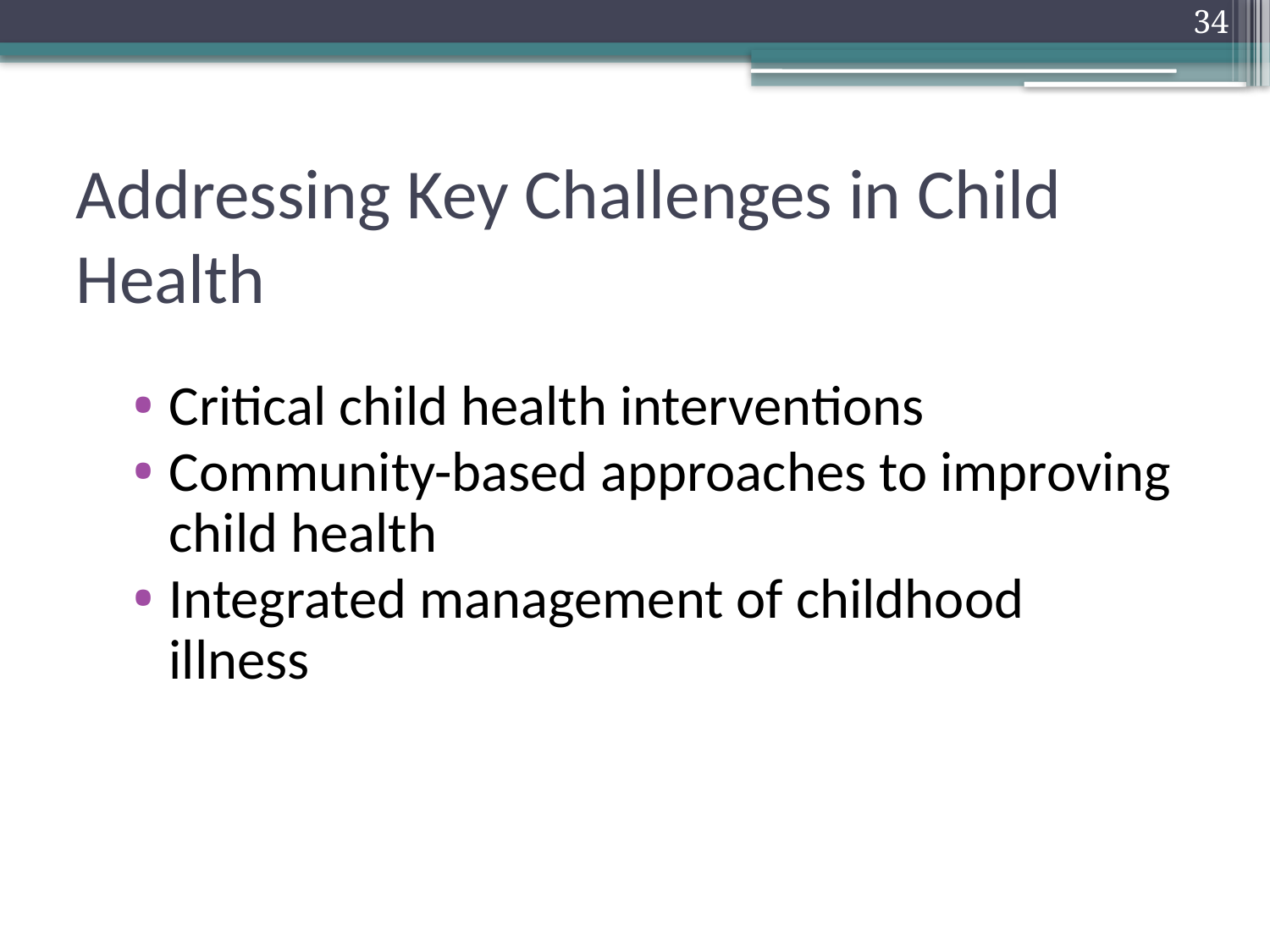

34
# Addressing Key Challenges in Child Health
Critical child health interventions
Community-based approaches to improving child health
Integrated management of childhood illness

## Slide 35
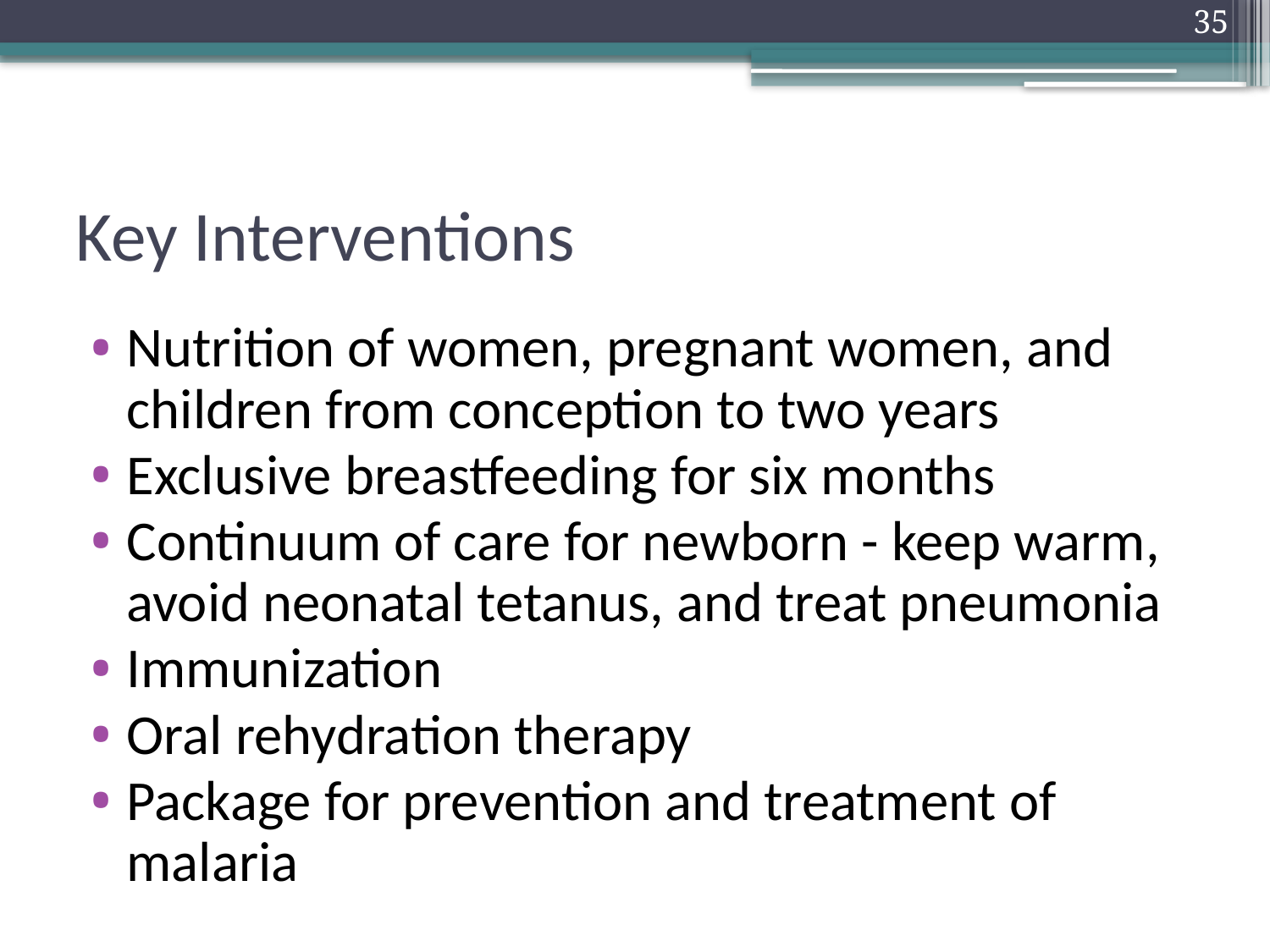

35
# Key Interventions
Nutrition of women, pregnant women, and children from conception to two years
Exclusive breastfeeding for six months
Continuum of care for newborn - keep warm, avoid neonatal tetanus, and treat pneumonia
Immunization
Oral rehydration therapy
Package for prevention and treatment of malaria

## Slide 36
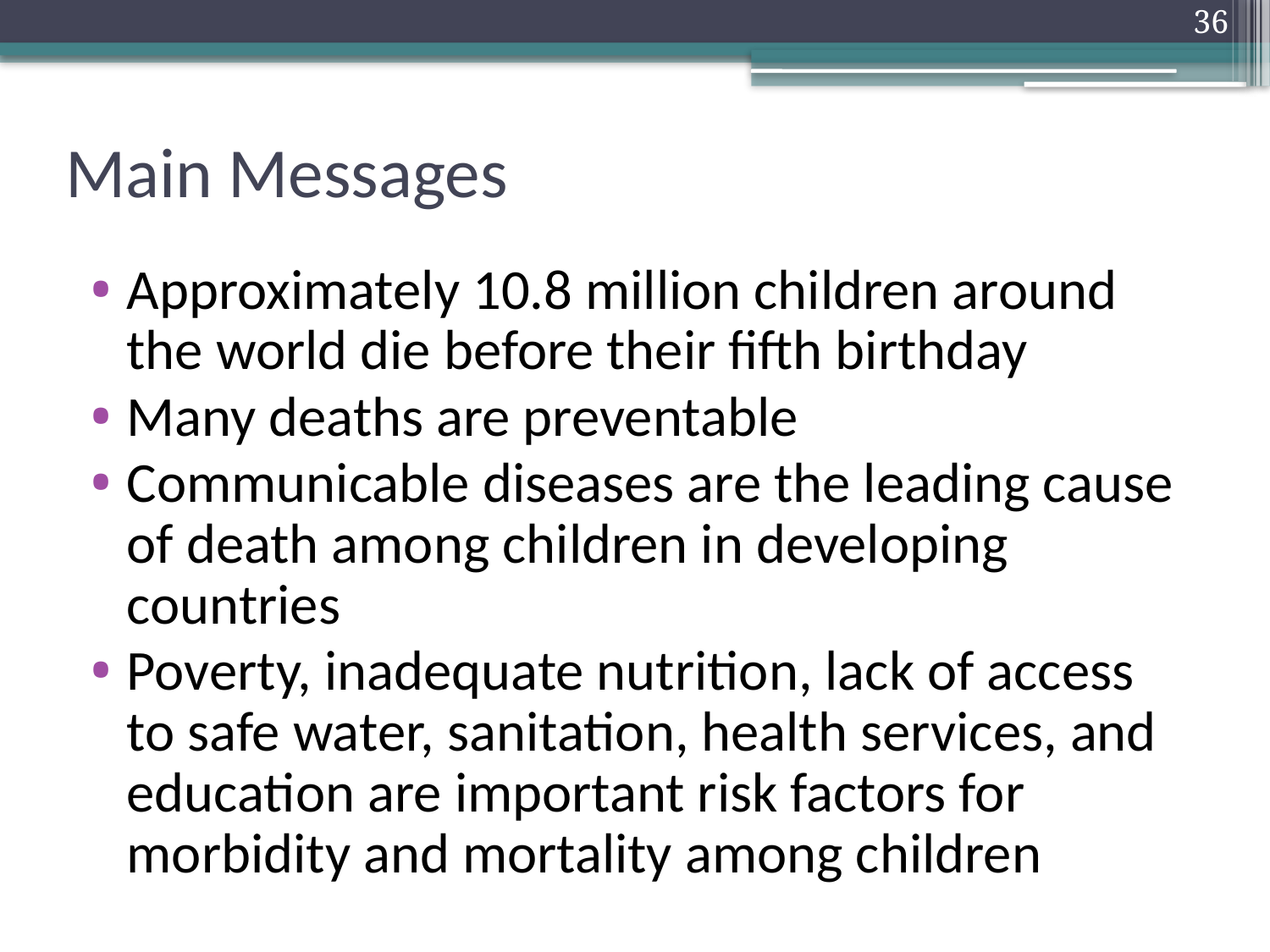

36
# Main Messages
Approximately 10.8 million children around the world die before their fifth birthday
Many deaths are preventable
Communicable diseases are the leading cause of death among children in developing countries
Poverty, inadequate nutrition, lack of access to safe water, sanitation, health services, and education are important risk factors for morbidity and mortality among children

## Slide 37
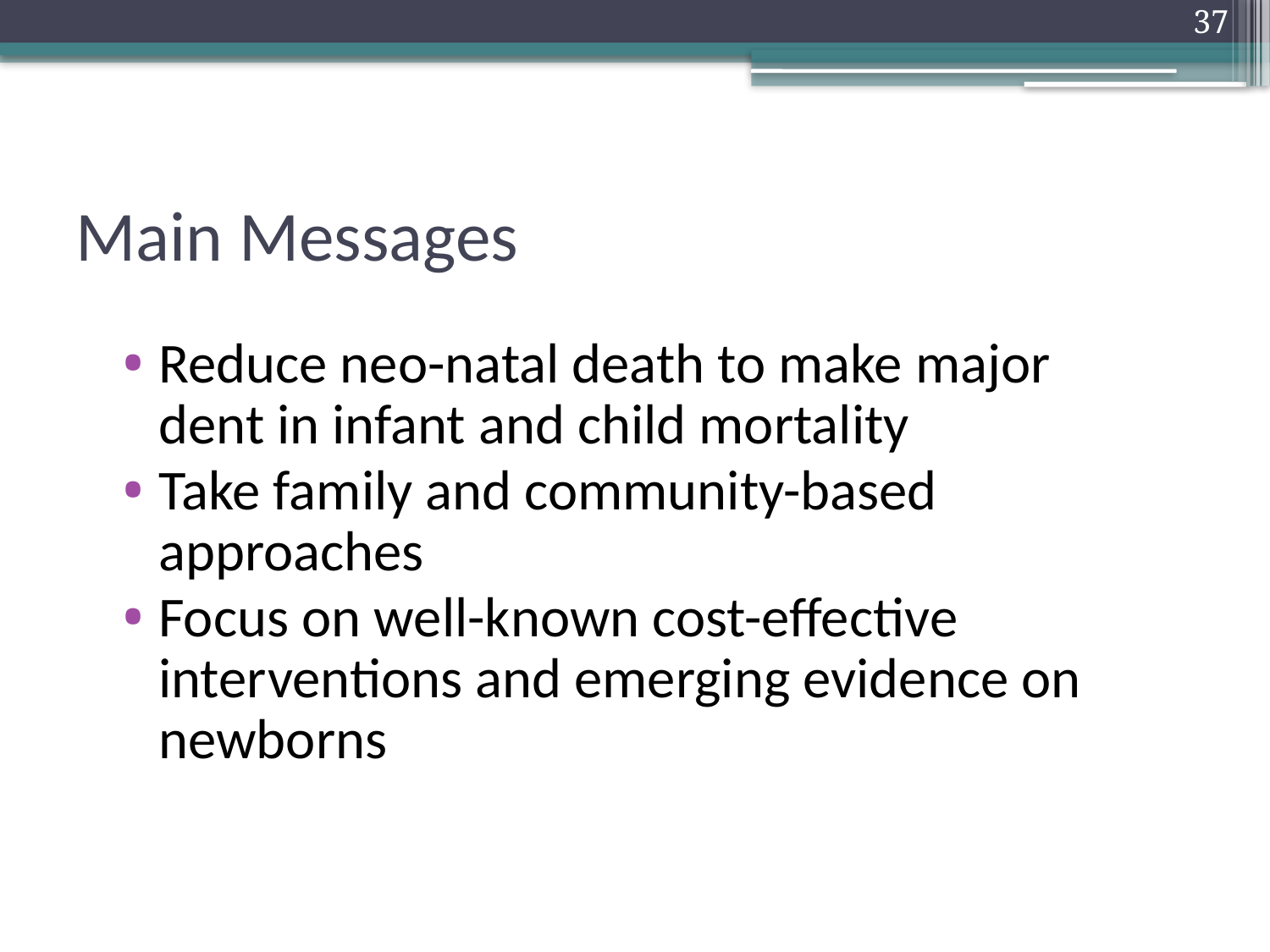

37
# Main Messages
Reduce neo-natal death to make major dent in infant and child mortality
Take family and community-based approaches
Focus on well-known cost-effective interventions and emerging evidence on newborns
