# Supplementary material for: The JeffSTARS Advocacy and Community Partnership Elective: A Closer Look at Child Health Advocacy in Action
Source: MedEdPORTAL. 2016 Dec 31;12:10526. doi: 10.15766/mep_2374-8265.10526 (PMC6365684; doi:10.15766/mep_2374-8265.10526)
Supplement: Supplementary file 1 — A. CM1. Course Implementation at New Institution Checklist.docx B. CM2. Elective Checklist.docx C. CM3. Sample Schedule.docx D. CM4. Seminar Topic List With Learning Objectives.docx E. CM5. Syllabus Bibliography.docx F. CM6. List of Community Partners.docx G. CM7. Orientation for New Community Partner.docx H. CM8. Selected Past Projects.docx I. CM9. Sample Fact Sheets for Legislative Visits.docx J. Seminar Materials folder K. ET1. Advocacy Elective Assessment 1.pdf L. ET2. Advocacy Elective Assessment 2.pdf M. ET3. Trainee Evaluation by Community or Faculty Mentor.docx N. ET4. Trainee Evaluation of Seminar.docx O. ET5. Trainee Evaluation of Community Partner.docx P. ET6. Final Report Template.docx Q. Selected Trainee Abstracts and Presented Results folder [file mep-12-10526-s001.zip › J._Seminar_Materials_folder/7._Media_Advocacy.pptx]

## Slide 1
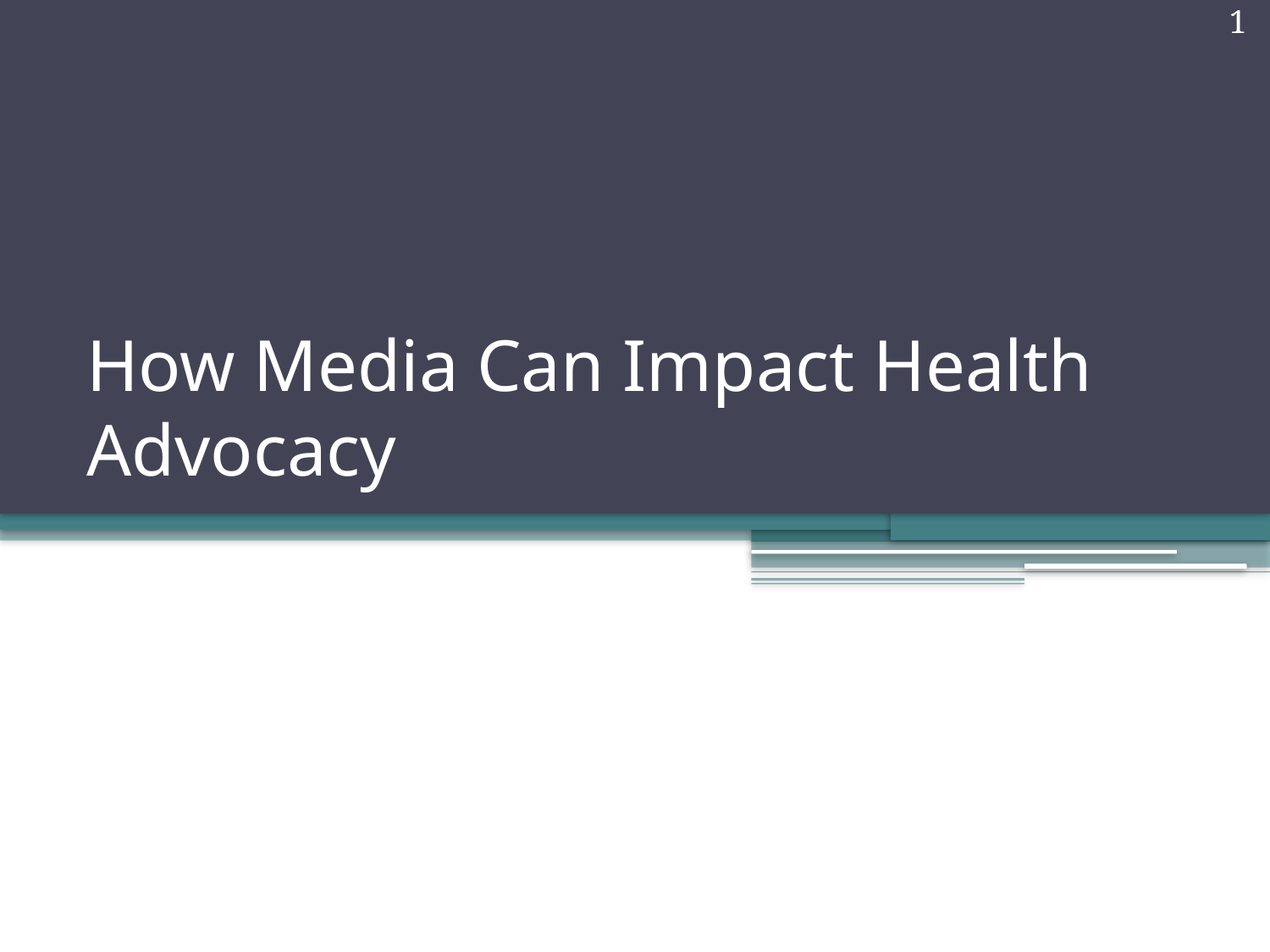

1
# How Media Can Impact Health Advocacy

## Slide 2
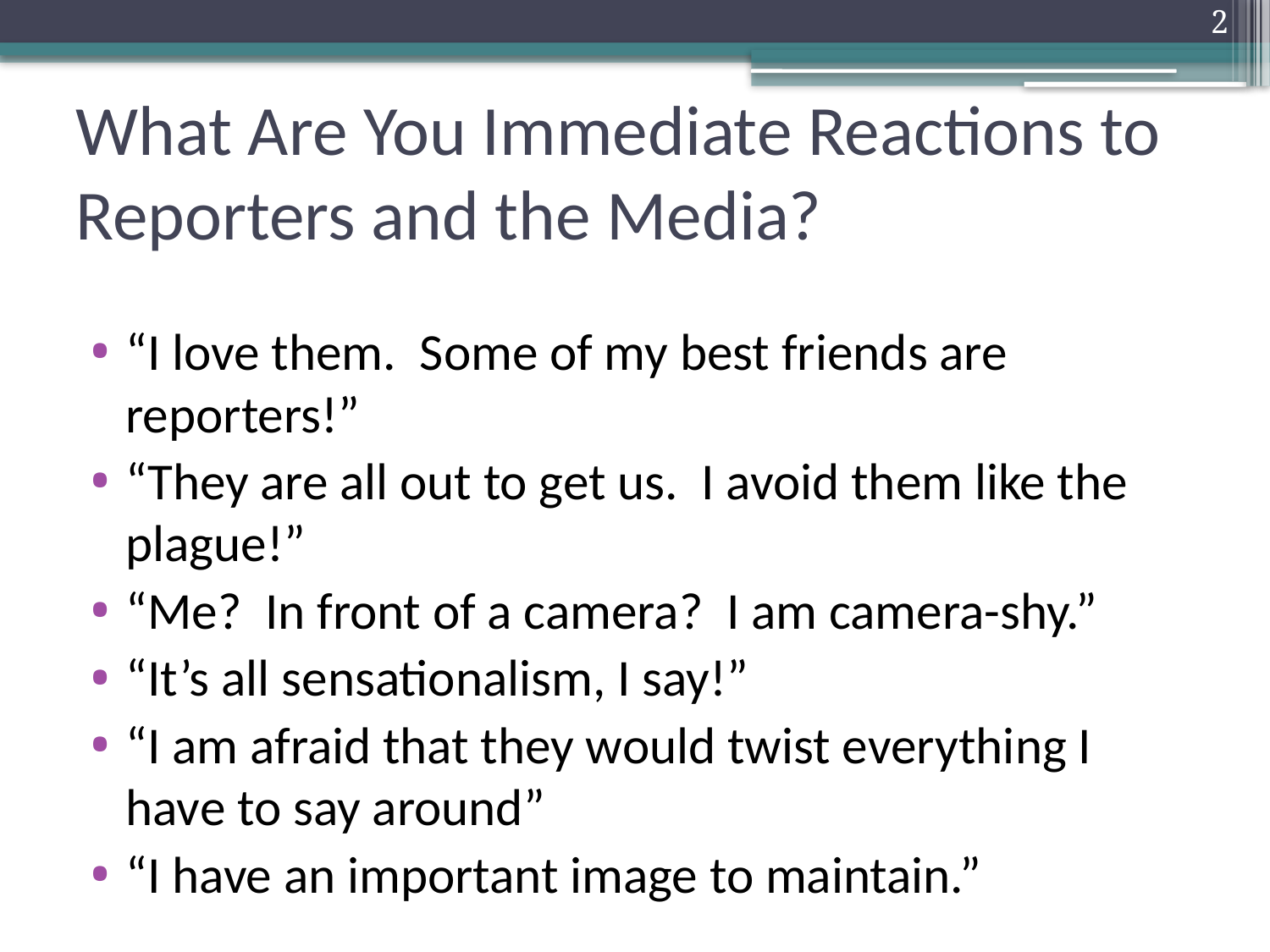

2
# What Are You Immediate Reactions to Reporters and the Media?
“I love them. Some of my best friends are reporters!”
“They are all out to get us. I avoid them like the plague!”
“Me? In front of a camera? I am camera-shy.”
“It’s all sensationalism, I say!”
“I am afraid that they would twist everything I have to say around”
“I have an important image to maintain.”

## Slide 3
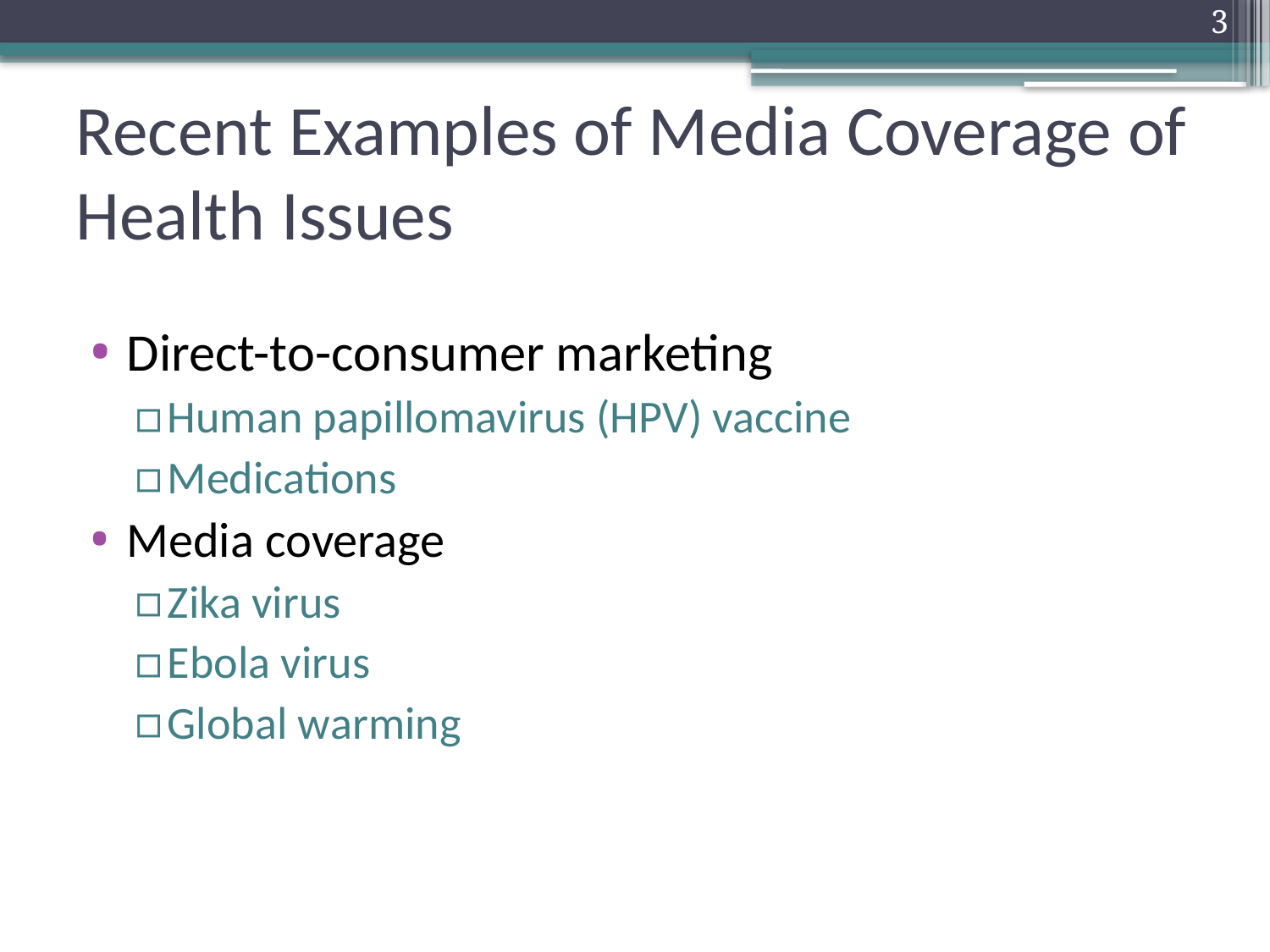

3
# Recent Examples of Media Coverage of Health Issues
Direct-to-consumer marketing
Human papillomavirus (HPV) vaccine
Medications
Media coverage
Zika virus
Ebola virus
Global warming

## Slide 4
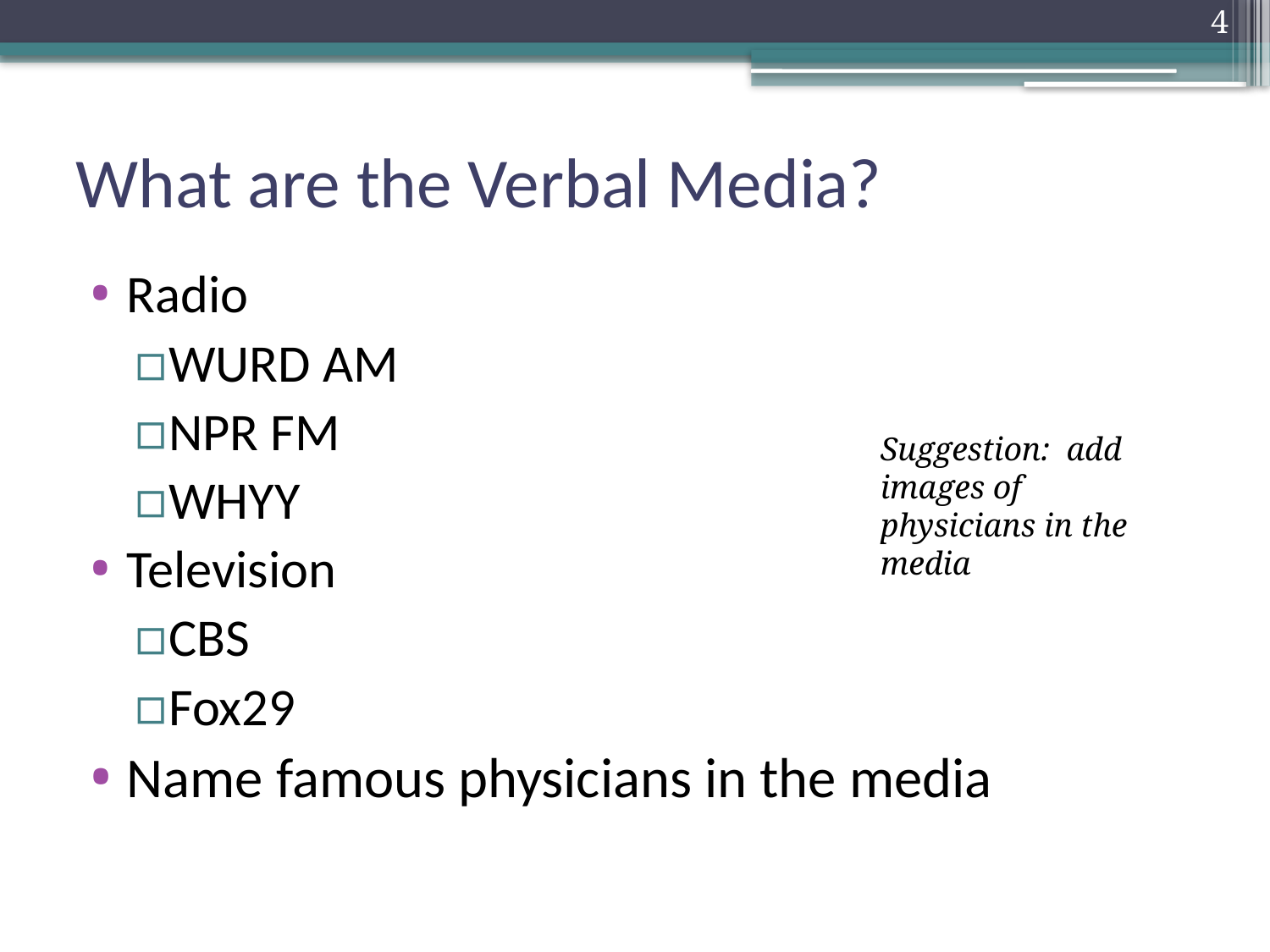

4
# What are the Verbal Media?
Radio
WURD AM
NPR FM
WHYY
Television
CBS
Fox29
Name famous physicians in the media
Suggestion: add images of physicians in the media

## Slide 5
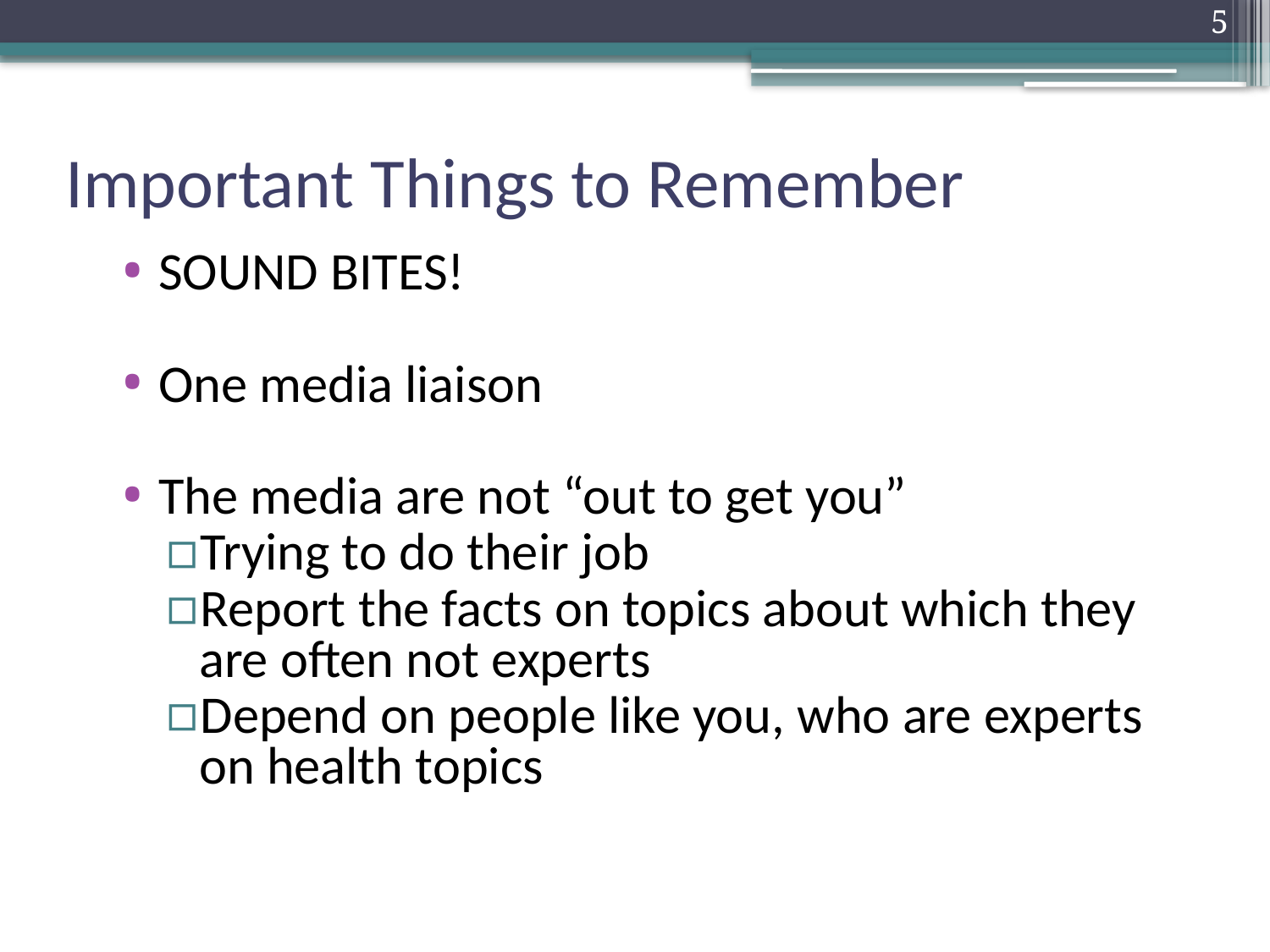

5
# Important Things to Remember
SOUND BITES!
One media liaison
The media are not “out to get you”
Trying to do their job
Report the facts on topics about which they are often not experts
Depend on people like you, who are experts on health topics

## Slide 6
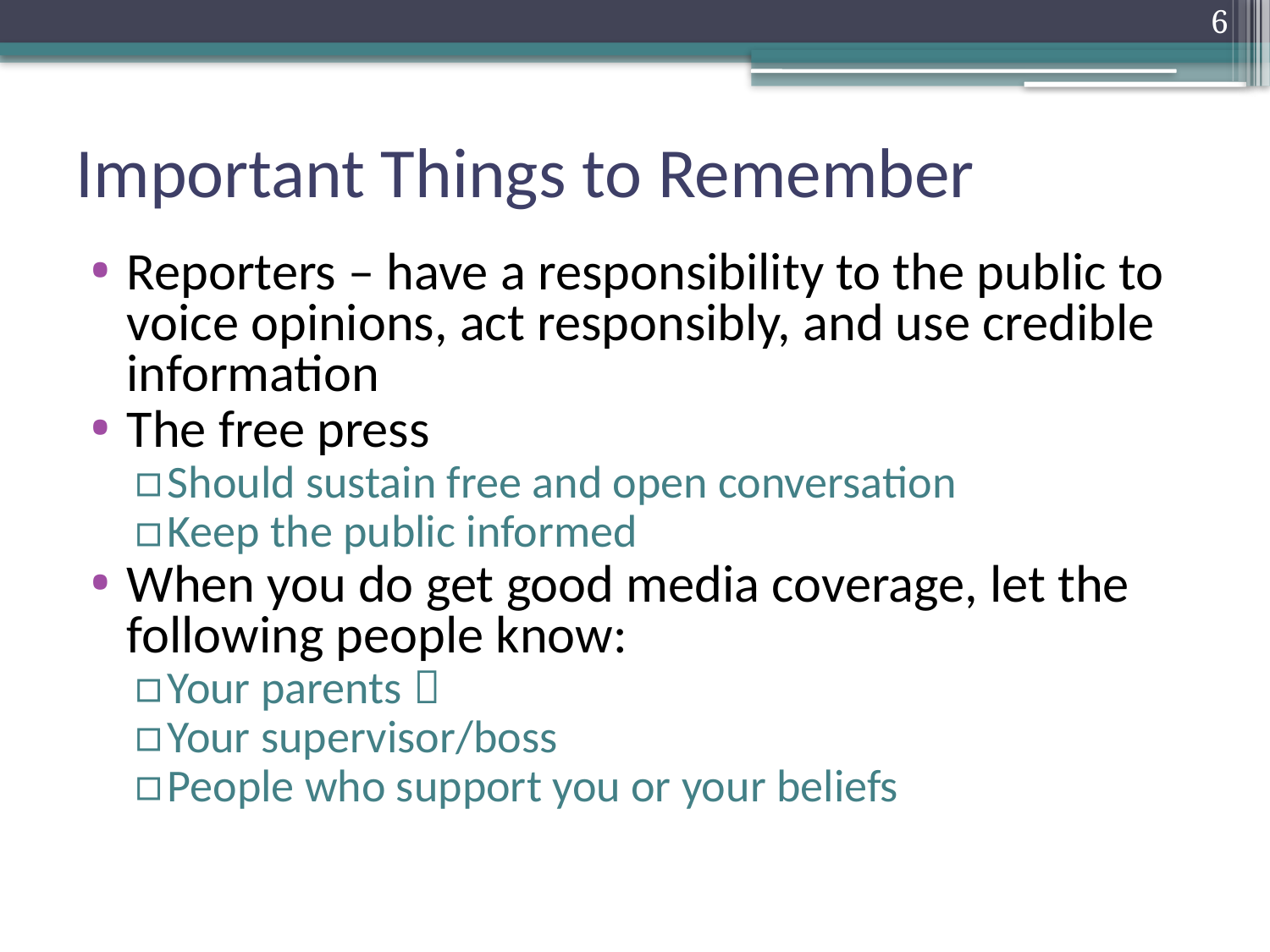

6
# Important Things to Remember
Reporters – have a responsibility to the public to voice opinions, act responsibly, and use credible information
The free press
Should sustain free and open conversation
Keep the public informed
When you do get good media coverage, let the following people know:
Your parents 
Your supervisor/boss
People who support you or your beliefs

## Slide 7
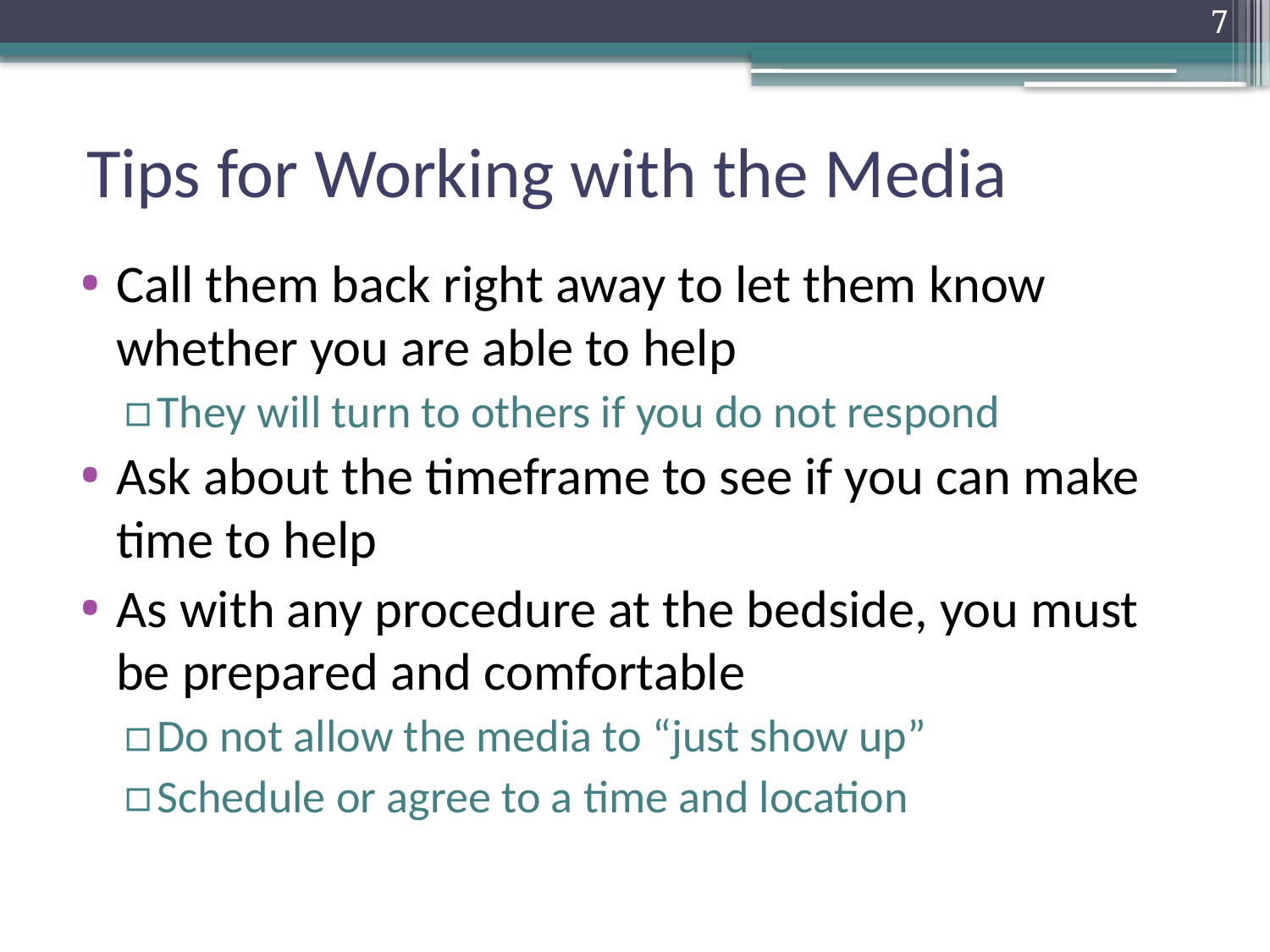

7
# Tips for Working with the Media
Call them back right away to let them know whether you are able to help
They will turn to others if you do not respond
Ask about the timeframe to see if you can make time to help
As with any procedure at the bedside, you must be prepared and comfortable
Do not allow the media to “just show up”
Schedule or agree to a time and location

## Slide 8
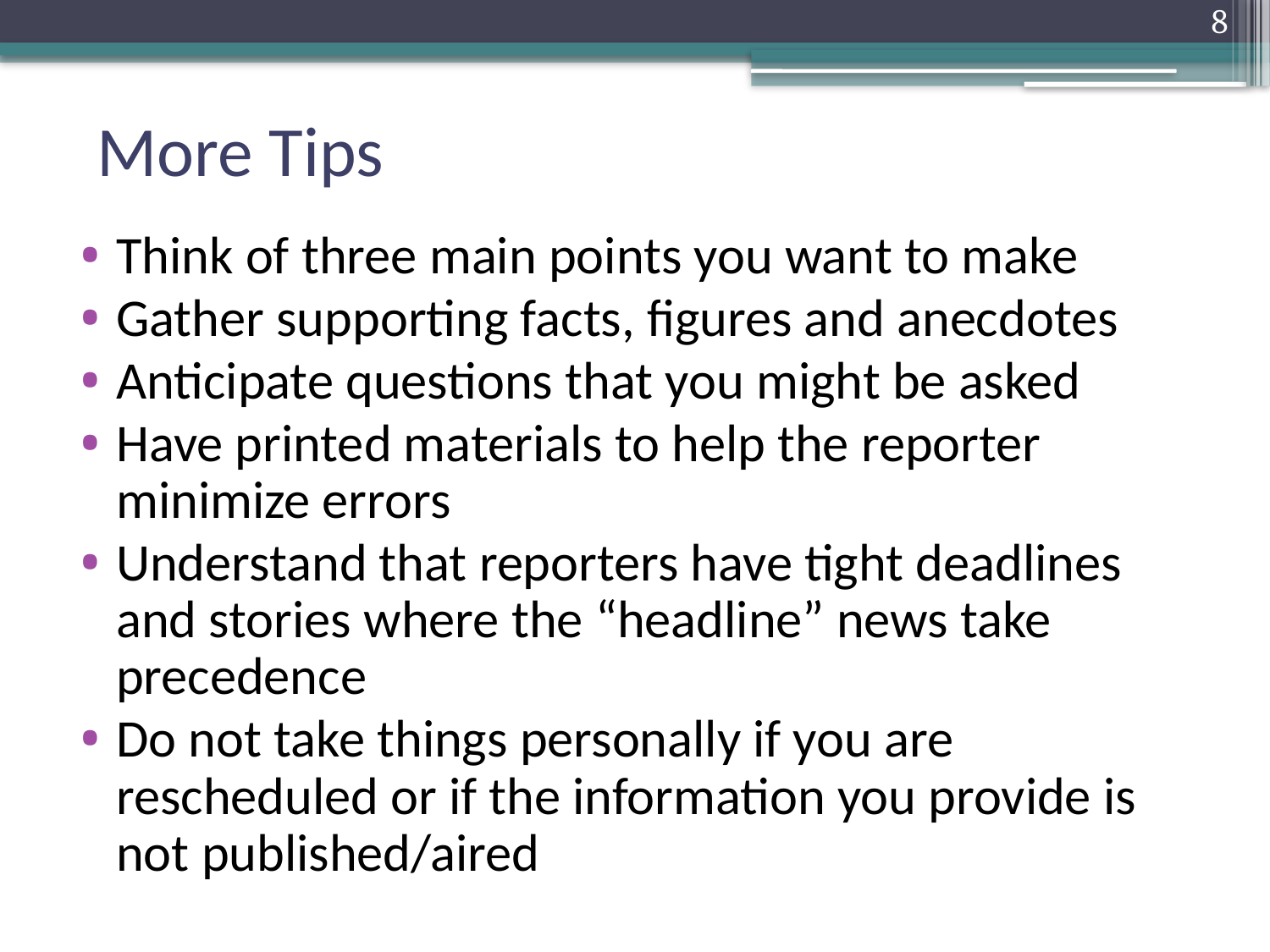

8
# More Tips
Think of three main points you want to make
Gather supporting facts, figures and anecdotes
Anticipate questions that you might be asked
Have printed materials to help the reporter minimize errors
Understand that reporters have tight deadlines and stories where the “headline” news take precedence
Do not take things personally if you are rescheduled or if the information you provide is not published/aired

## Slide 9
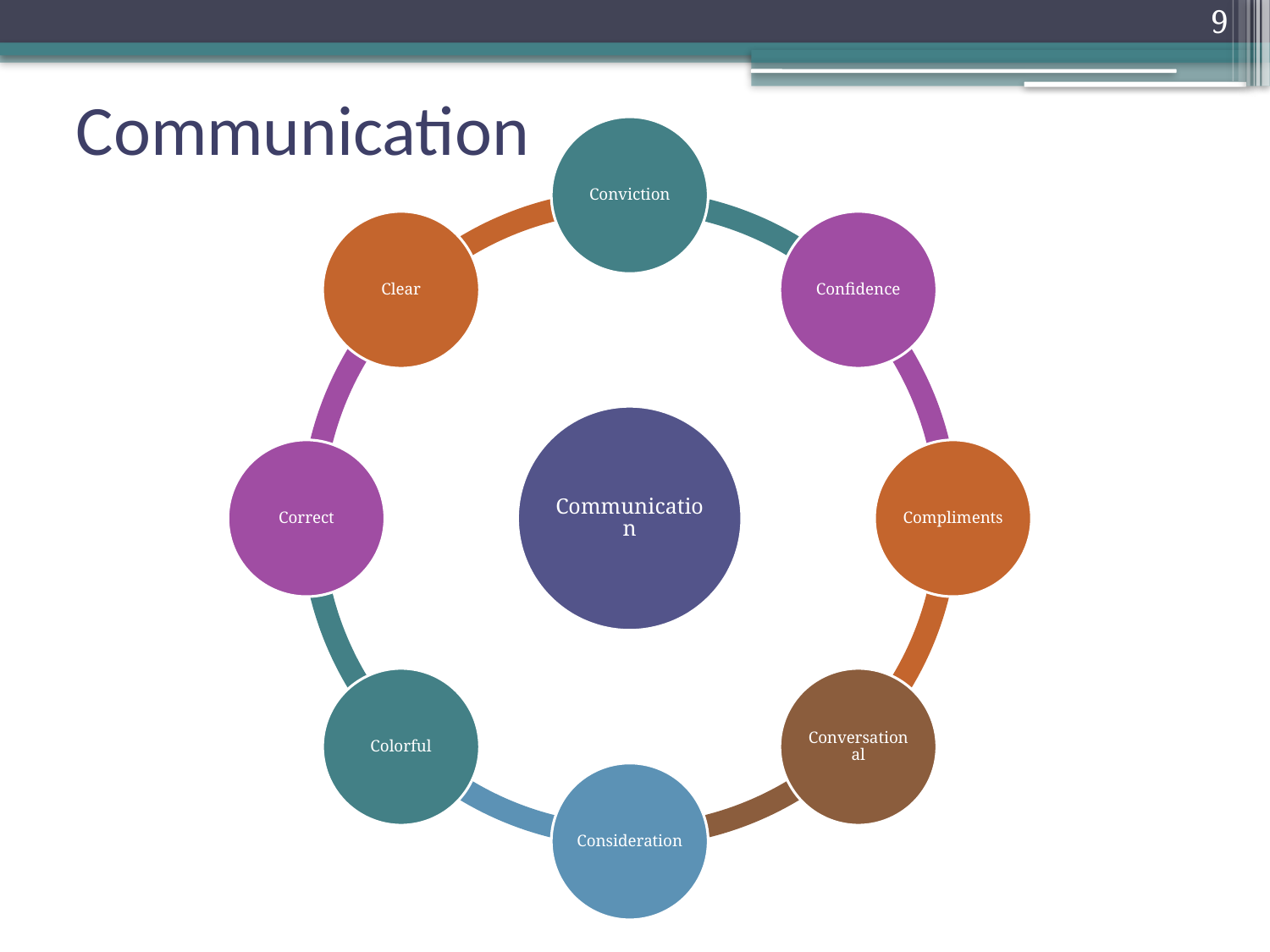

9
# Communication

## Slide 10
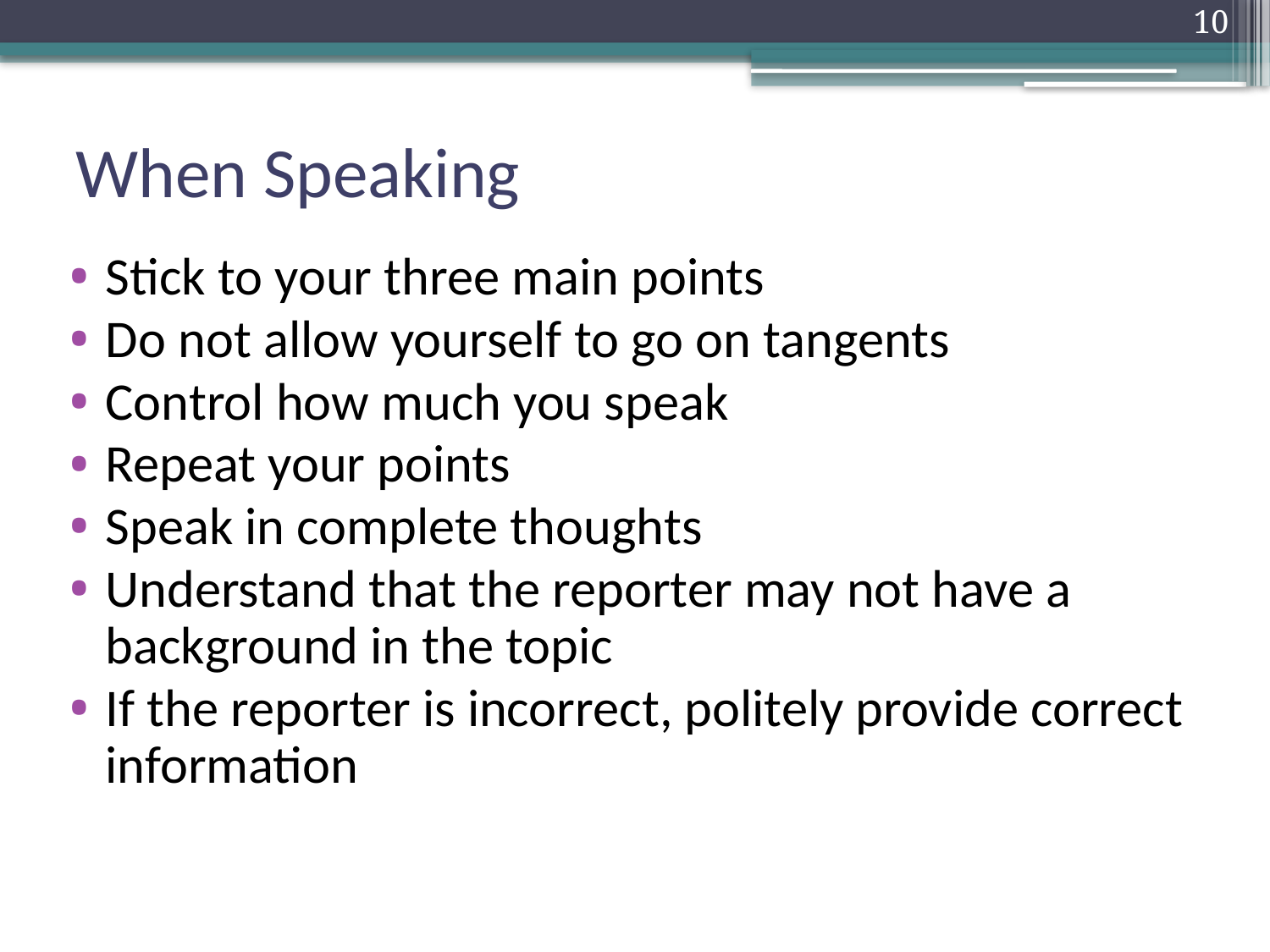

10
# When Speaking
Stick to your three main points
Do not allow yourself to go on tangents
Control how much you speak
Repeat your points
Speak in complete thoughts
Understand that the reporter may not have a background in the topic
If the reporter is incorrect, politely provide correct information

## Slide 11
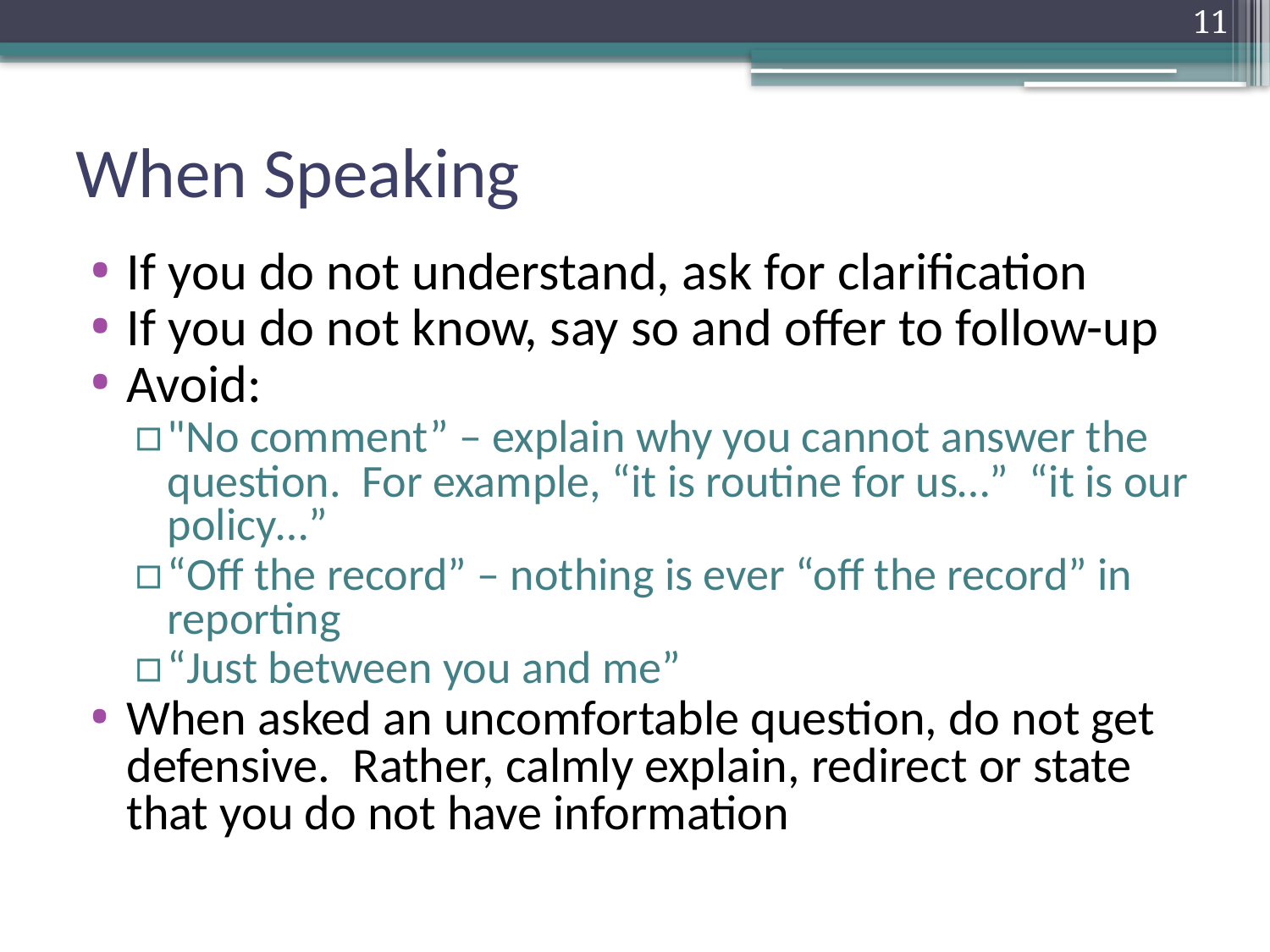

11
# When Speaking
If you do not understand, ask for clarification
If you do not know, say so and offer to follow-up
Avoid:
"No comment” – explain why you cannot answer the question. For example, “it is routine for us…” “it is our policy…”
“Off the record” – nothing is ever “off the record” in reporting
“Just between you and me”
When asked an uncomfortable question, do not get defensive. Rather, calmly explain, redirect or state that you do not have information

## Slide 12
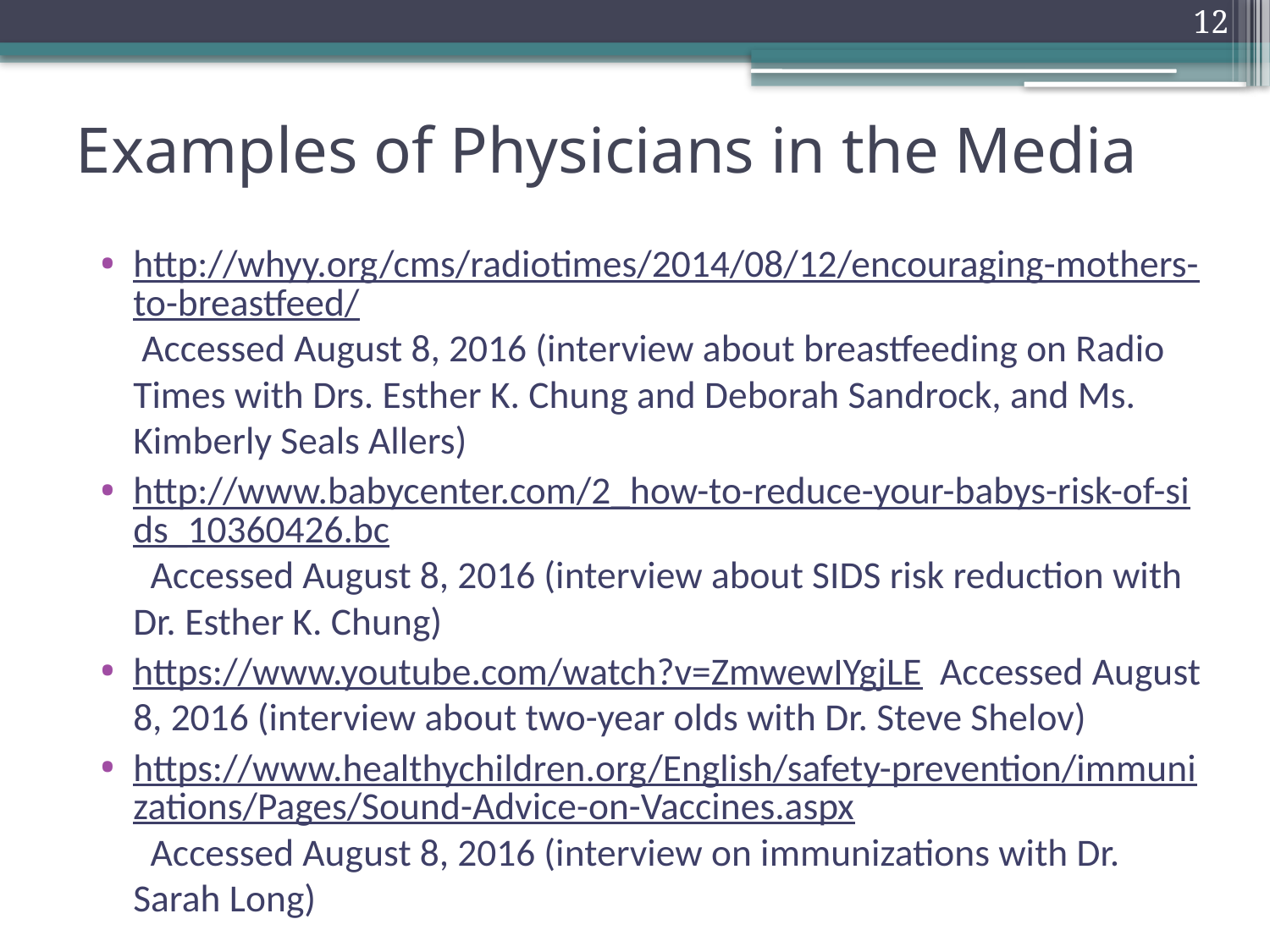

12
# Examples of Physicians in the Media
http://whyy.org/cms/radiotimes/2014/08/12/encouraging-mothers-to-breastfeed/ Accessed August 8, 2016 (interview about breastfeeding on Radio Times with Drs. Esther K. Chung and Deborah Sandrock, and Ms. Kimberly Seals Allers)
http://www.babycenter.com/2_how-to-reduce-your-babys-risk-of-sids_10360426.bc Accessed August 8, 2016 (interview about SIDS risk reduction with Dr. Esther K. Chung)
https://www.youtube.com/watch?v=ZmwewIYgjLE Accessed August 8, 2016 (interview about two-year olds with Dr. Steve Shelov)
https://www.healthychildren.org/English/safety-prevention/immunizations/Pages/Sound-Advice-on-Vaccines.aspx Accessed August 8, 2016 (interview on immunizations with Dr. Sarah Long)

## Slide 13
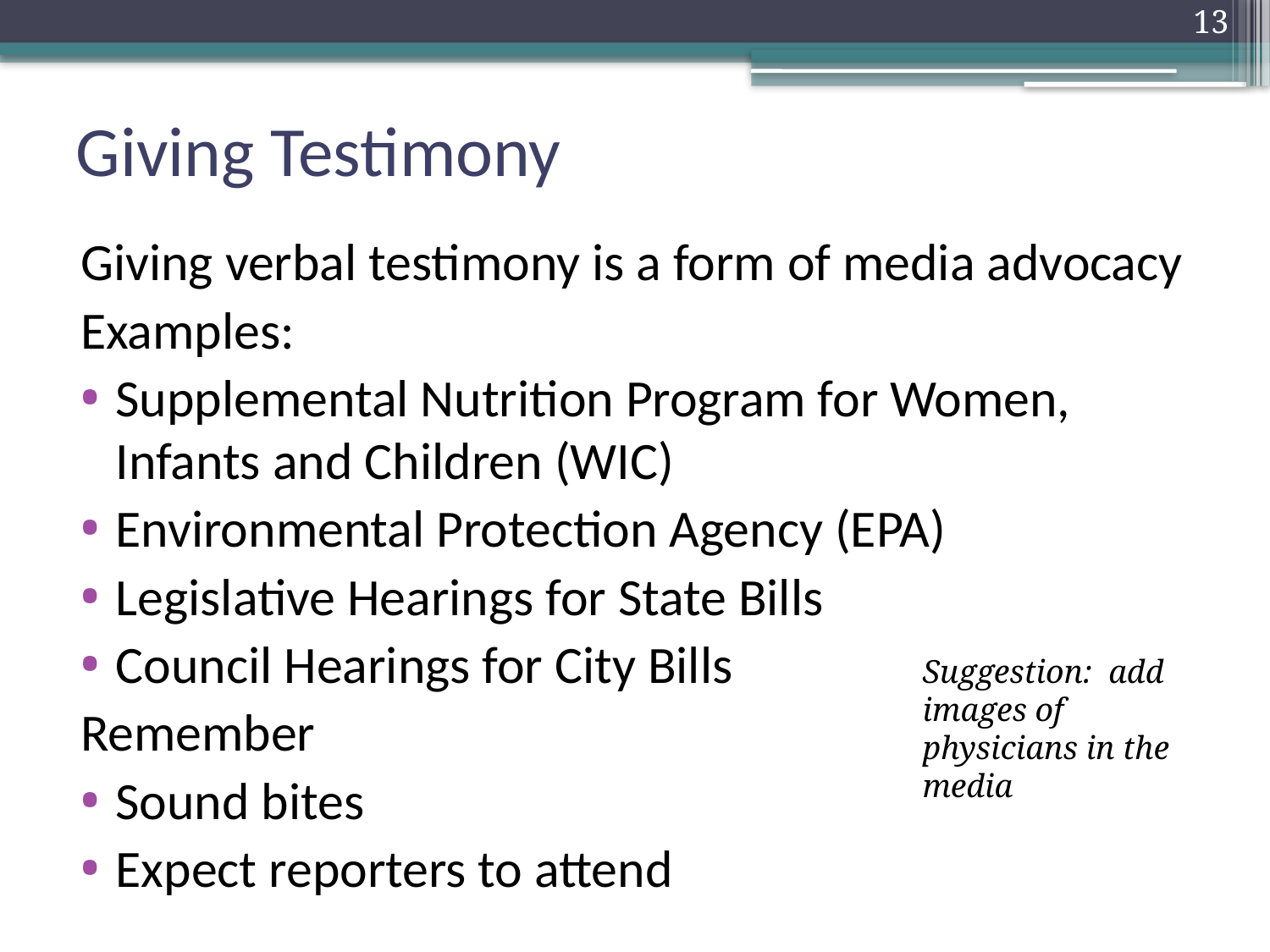

13
# Giving Testimony
Giving verbal testimony is a form of media advocacy
Examples:
Supplemental Nutrition Program for Women, Infants and Children (WIC)
Environmental Protection Agency (EPA)
Legislative Hearings for State Bills
Council Hearings for City Bills
Remember
Sound bites
Expect reporters to attend
Suggestion: add images of physicians in the media

## Slide 14
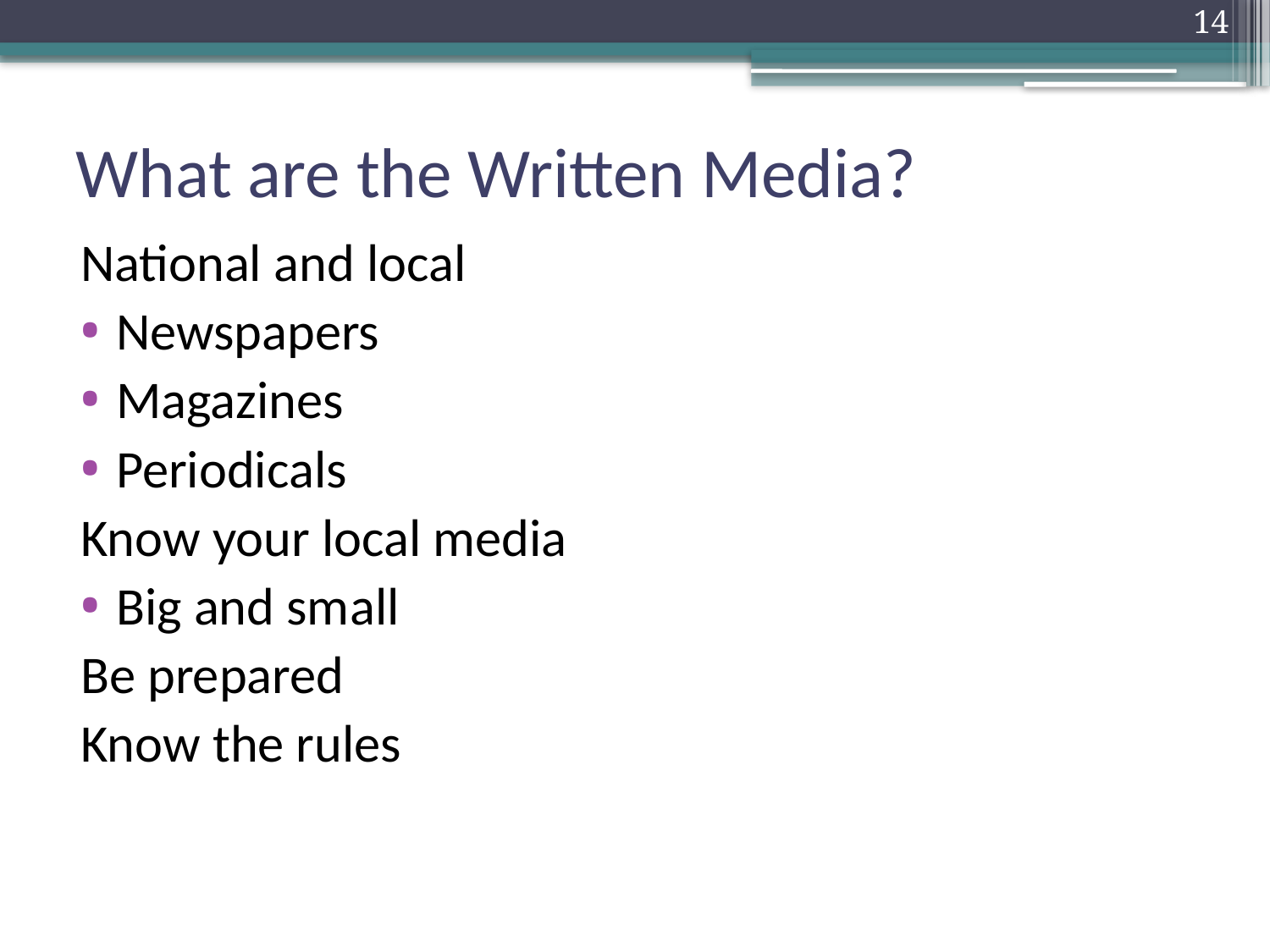

14
# What are the Written Media?
National and local
Newspapers
Magazines
Periodicals
Know your local media
Big and small
Be prepared
Know the rules

## Slide 15
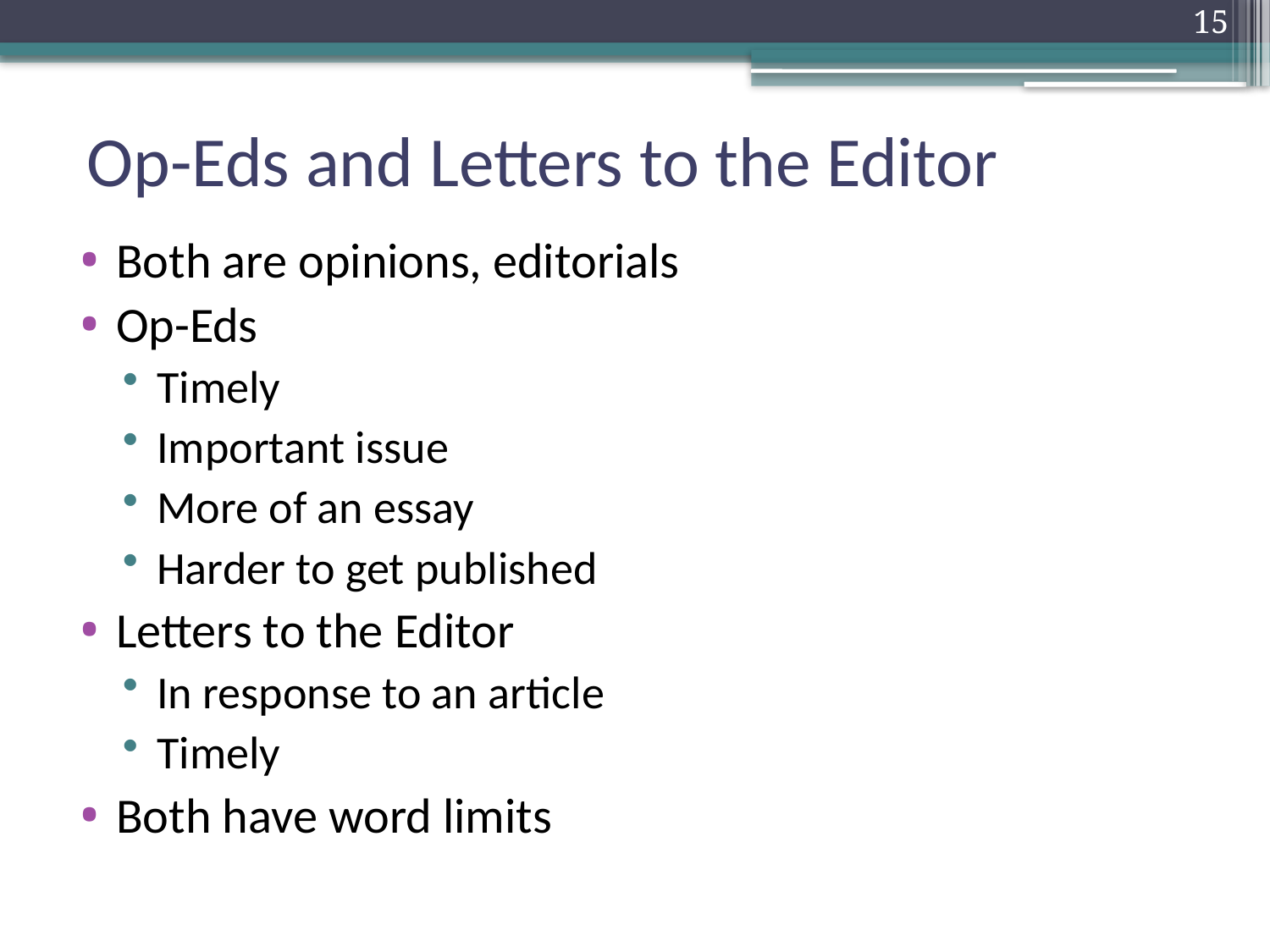

15
# Op-Eds and Letters to the Editor
Both are opinions, editorials
Op-Eds
Timely
Important issue
More of an essay
Harder to get published
Letters to the Editor
In response to an article
Timely
Both have word limits

## Slide 16
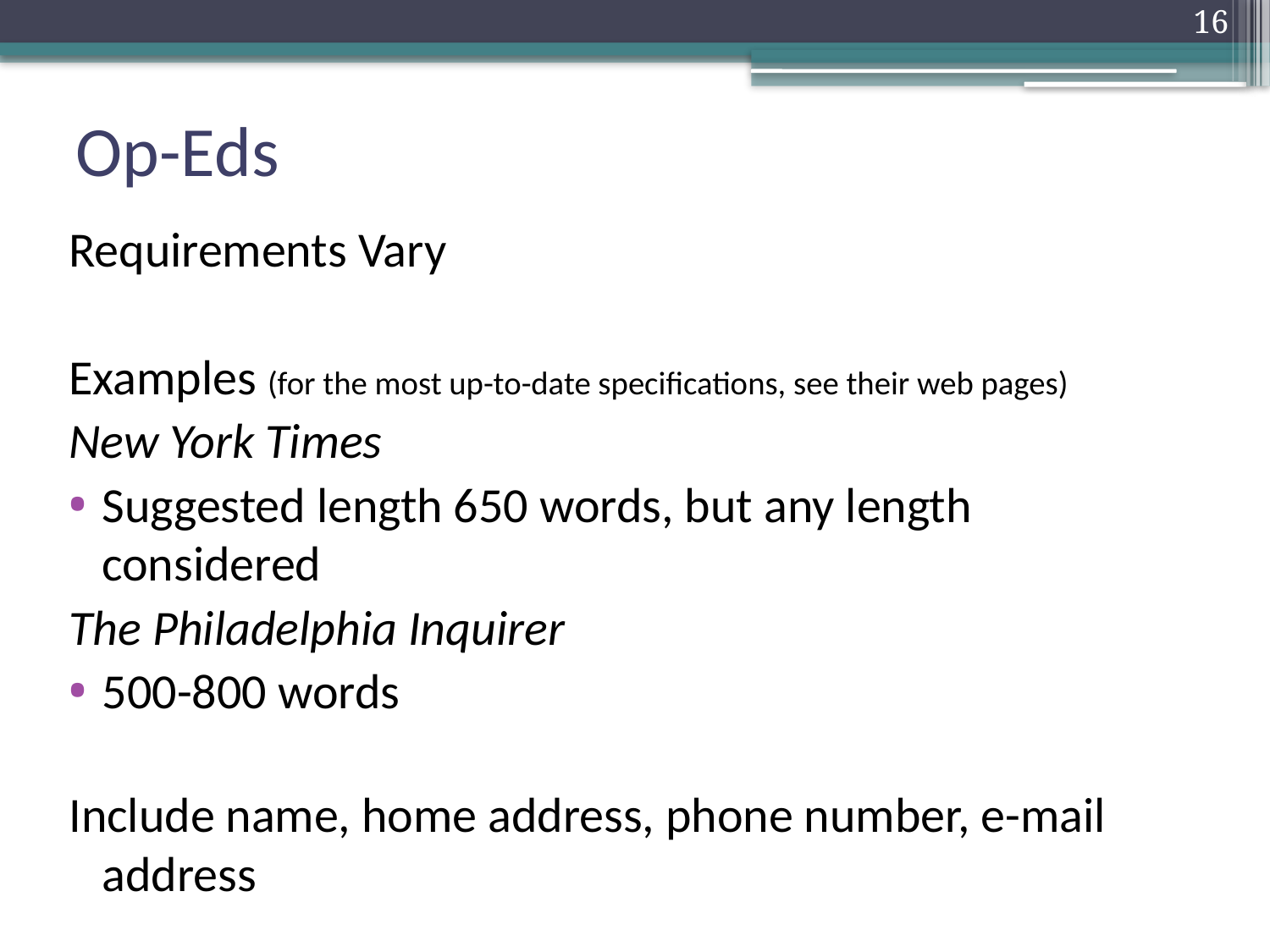

16
# Op-Eds
Requirements Vary
Examples (for the most up-to-date specifications, see their web pages)
New York Times
Suggested length 650 words, but any length considered
The Philadelphia Inquirer
500-800 words
Include name, home address, phone number, e-mail address

## Slide 17
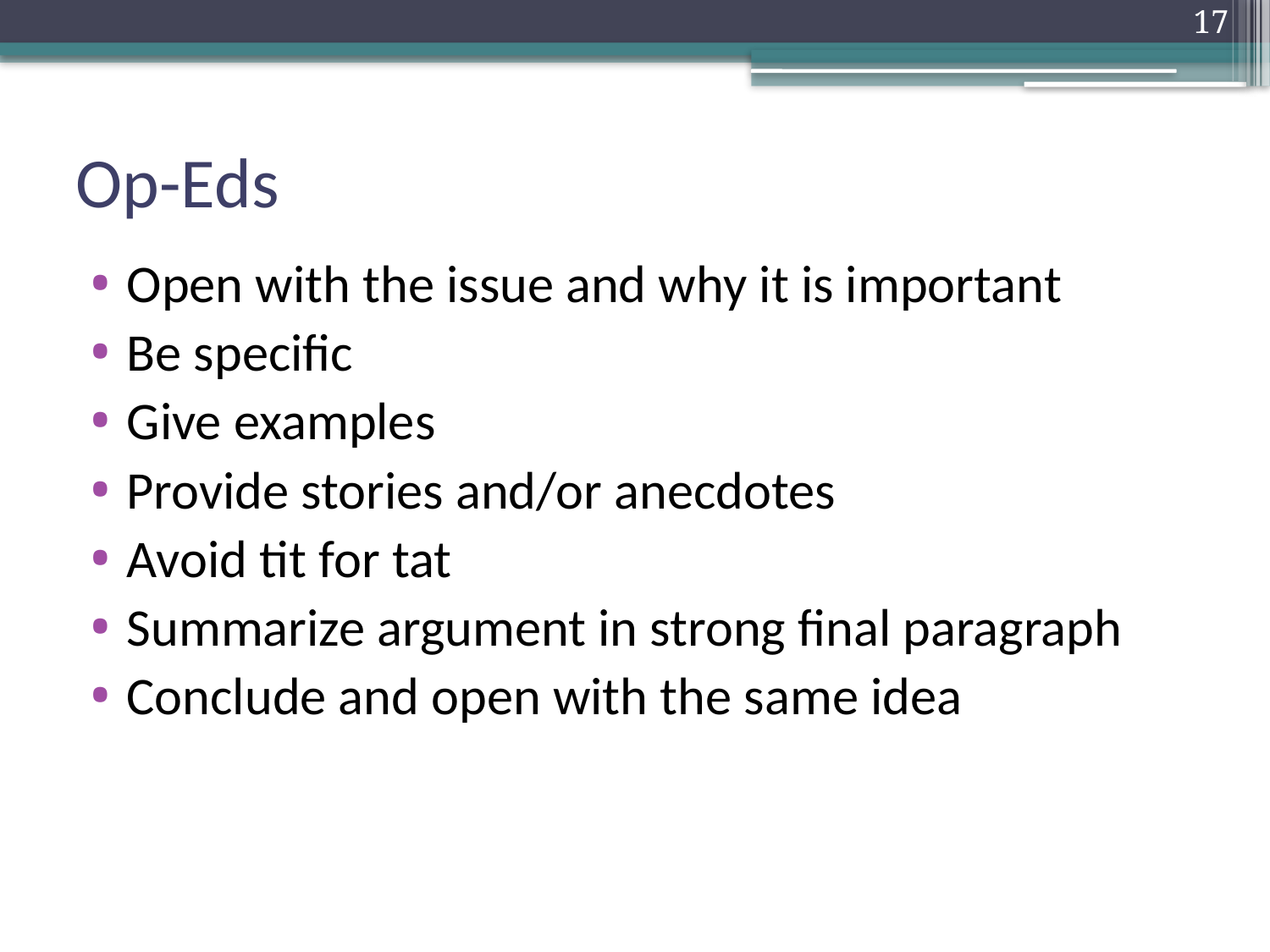

17
# Op-Eds
Open with the issue and why it is important
Be specific
Give examples
Provide stories and/or anecdotes
Avoid tit for tat
Summarize argument in strong final paragraph
Conclude and open with the same idea

## Slide 18
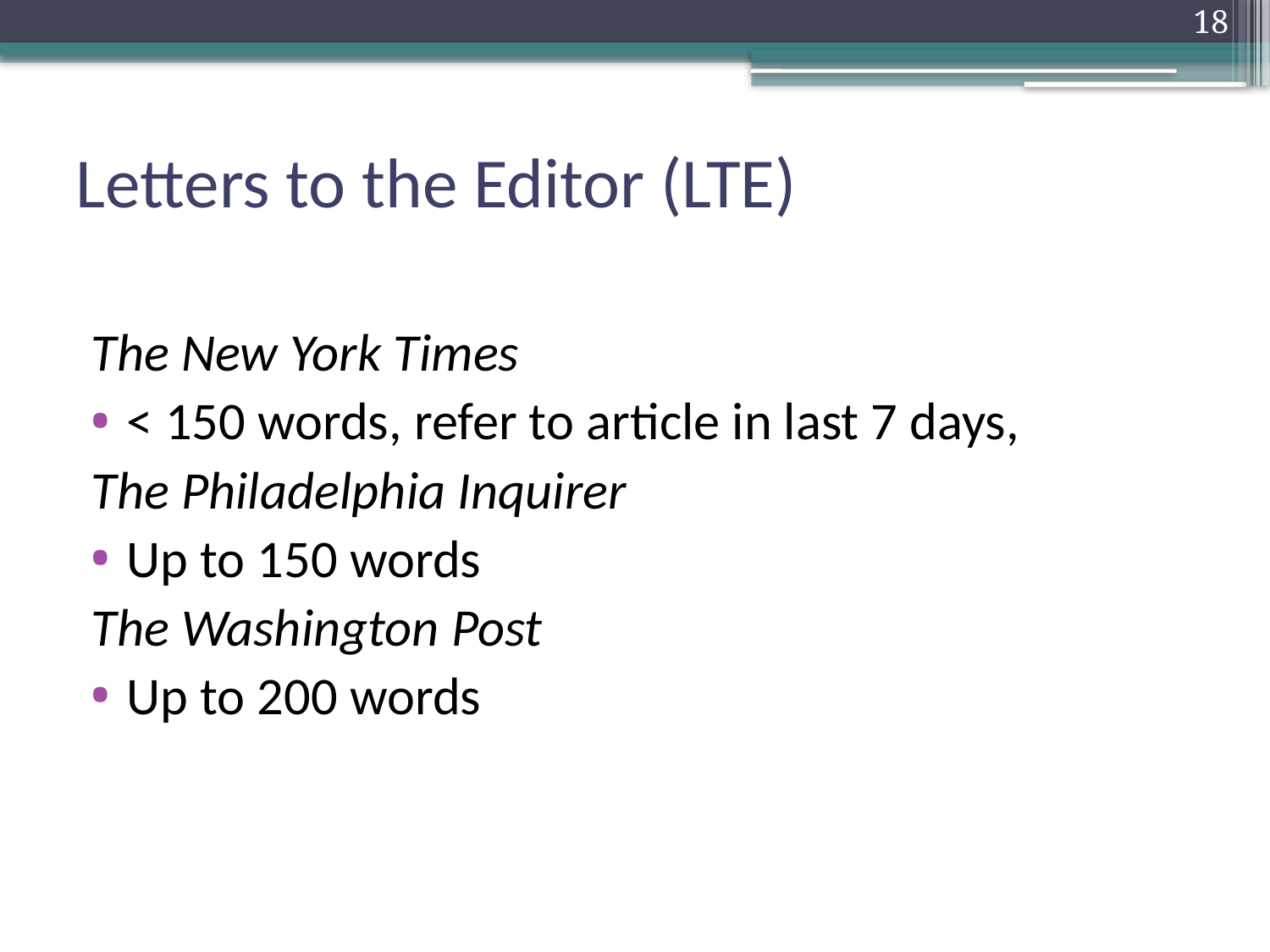

18
# Letters to the Editor (LTE)
The New York Times
< 150 words, refer to article in last 7 days,
The Philadelphia Inquirer
Up to 150 words
The Washington Post
Up to 200 words

## Slide 19
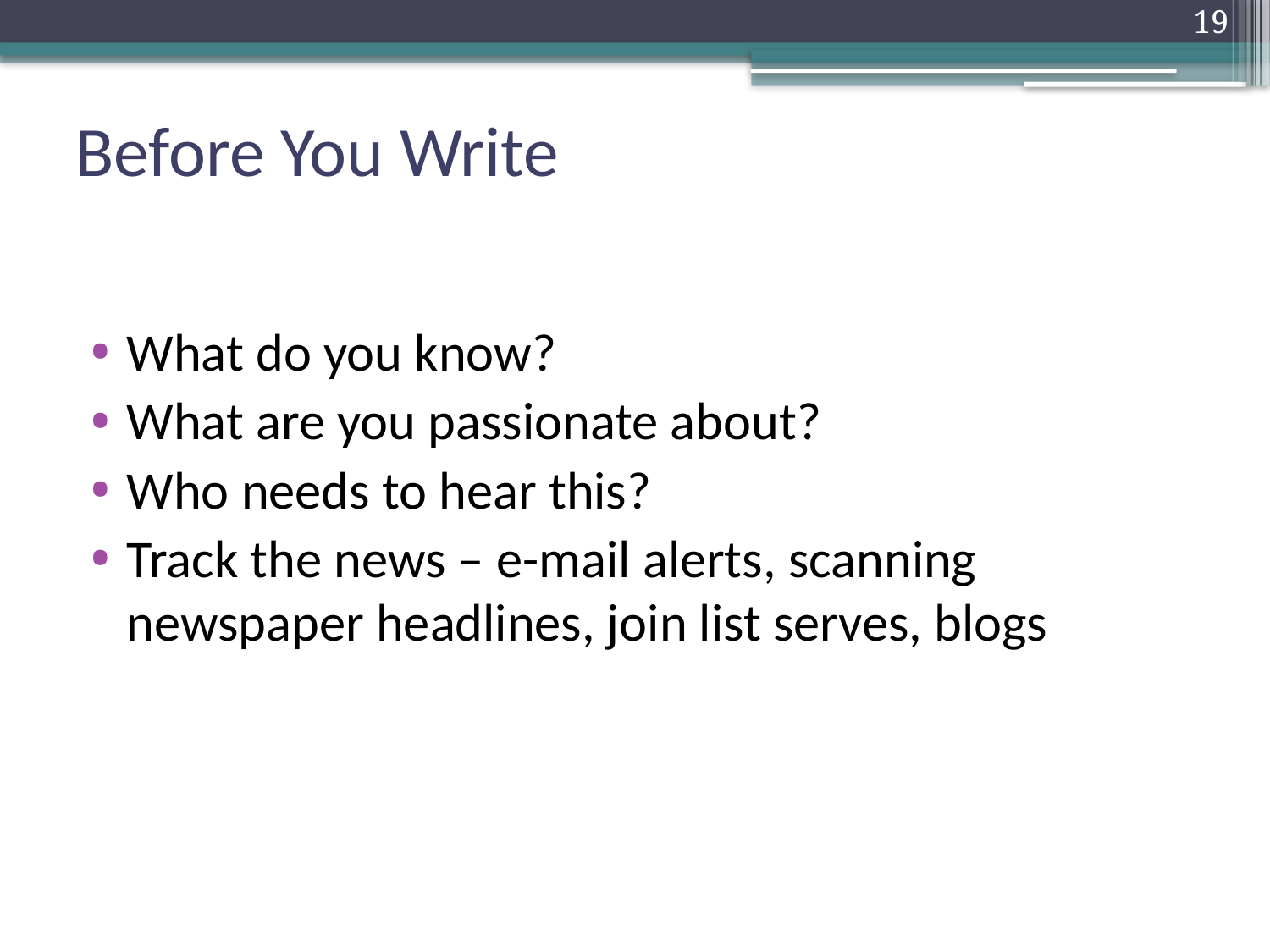

19
# Before You Write
What do you know?
What are you passionate about?
Who needs to hear this?
Track the news – e-mail alerts, scanning newspaper headlines, join list serves, blogs

## Slide 20
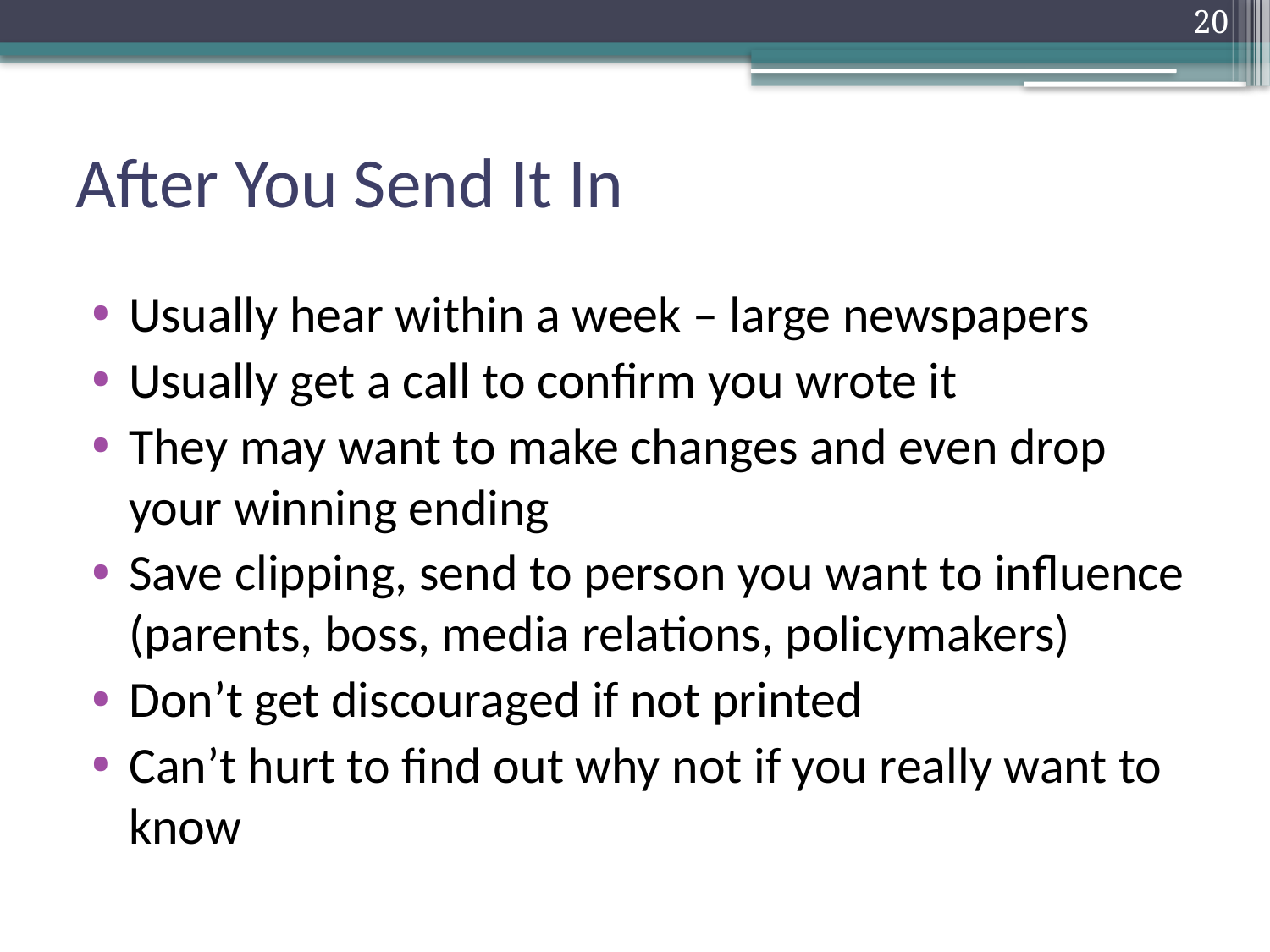

20
# After You Send It In
Usually hear within a week – large newspapers
Usually get a call to confirm you wrote it
They may want to make changes and even drop your winning ending
Save clipping, send to person you want to influence (parents, boss, media relations, policymakers)
Don’t get discouraged if not printed
Can’t hurt to find out why not if you really want to know

## Slide 21
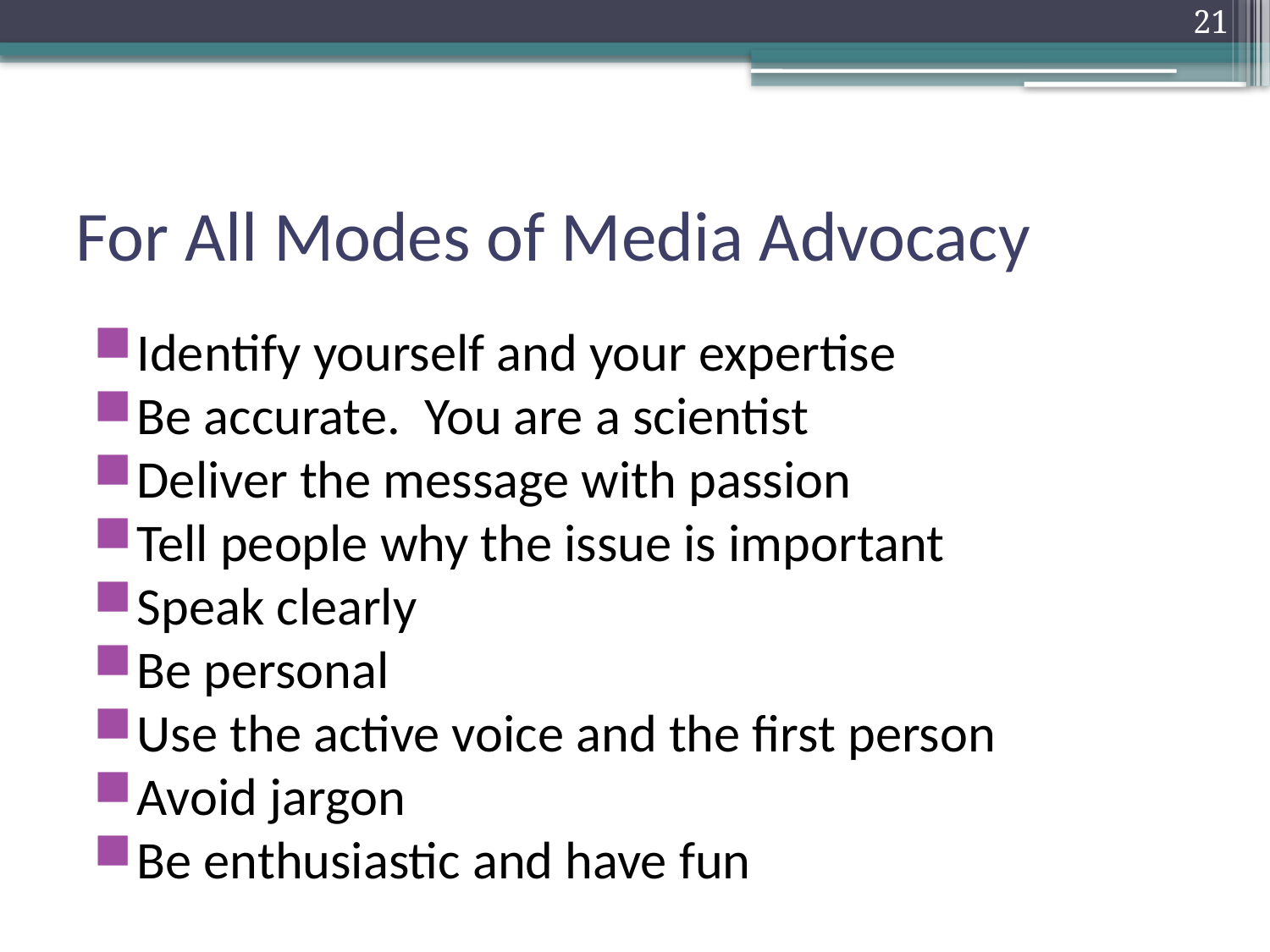

21
# For All Modes of Media Advocacy
Identify yourself and your expertise
Be accurate. You are a scientist
Deliver the message with passion
Tell people why the issue is important
Speak clearly
Be personal
Use the active voice and the first person
Avoid jargon
Be enthusiastic and have fun

## Slide 22
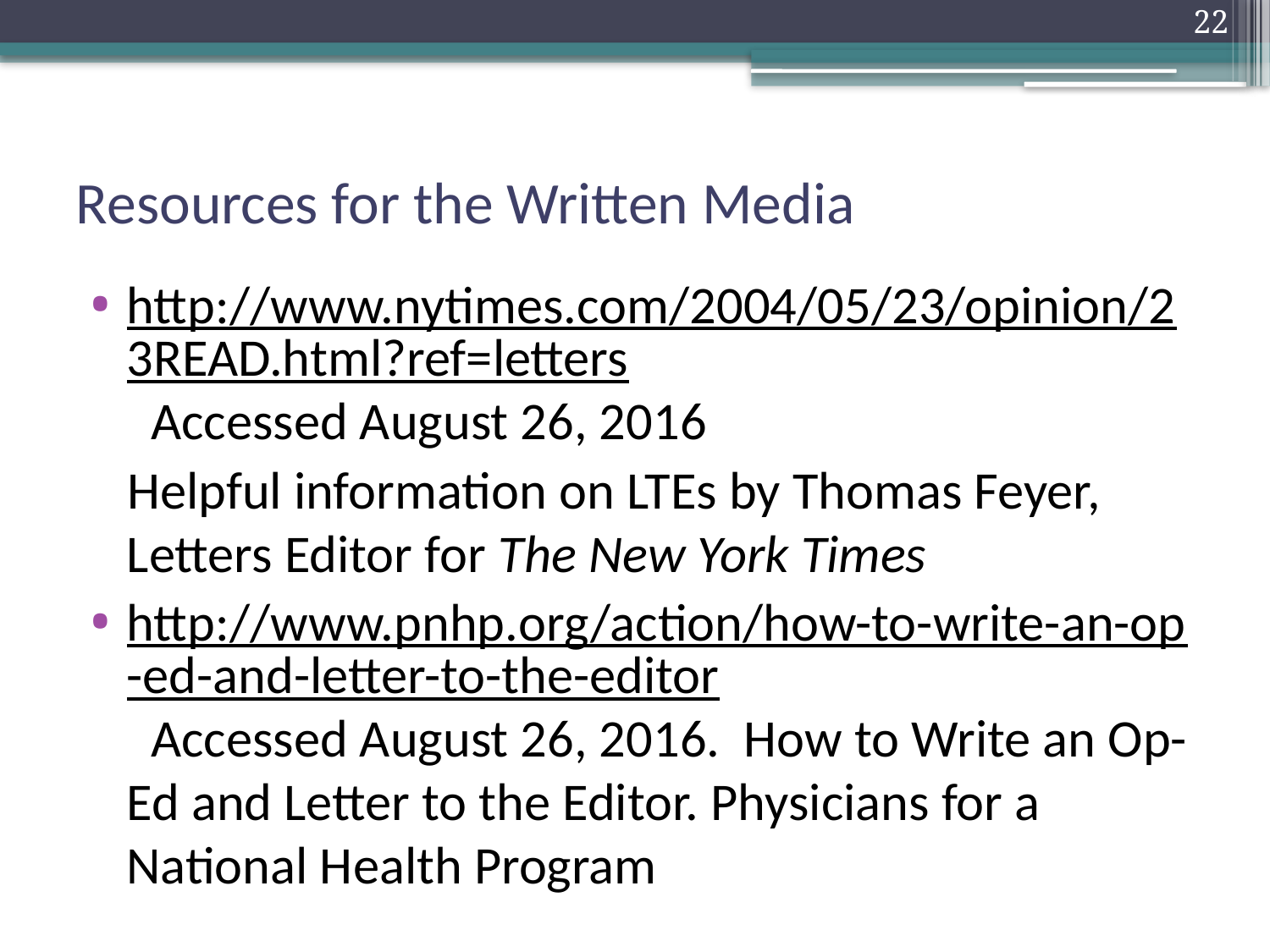

22
# Resources for the Written Media
http://www.nytimes.com/2004/05/23/opinion/23READ.html?ref=letters Accessed August 26, 2016
 Helpful information on LTEs by Thomas Feyer, Letters Editor for The New York Times
http://www.pnhp.org/action/how-to-write-an-op-ed-and-letter-to-the-editor Accessed August 26, 2016. How to Write an Op-Ed and Letter to the Editor. Physicians for a National Health Program
