# Supplementary material for: The JeffSTARS Advocacy and Community Partnership Elective: A Closer Look at Child Health Advocacy in Action
Source: MedEdPORTAL. 2016 Dec 31;12:10526. doi: 10.15766/mep_2374-8265.10526 (PMC6365684; doi:10.15766/mep_2374-8265.10526)
Supplement: Supplementary file 1 — A. CM1. Course Implementation at New Institution Checklist.docx B. CM2. Elective Checklist.docx C. CM3. Sample Schedule.docx D. CM4. Seminar Topic List With Learning Objectives.docx E. CM5. Syllabus Bibliography.docx F. CM6. List of Community Partners.docx G. CM7. Orientation for New Community Partner.docx H. CM8. Selected Past Projects.docx I. CM9. Sample Fact Sheets for Legislative Visits.docx J. Seminar Materials folder K. ET1. Advocacy Elective Assessment 1.pdf L. ET2. Advocacy Elective Assessment 2.pdf M. ET3. Trainee Evaluation by Community or Faculty Mentor.docx N. ET4. Trainee Evaluation of Seminar.docx O. ET5. Trainee Evaluation of Community Partner.docx P. ET6. Final Report Template.docx Q. Selected Trainee Abstracts and Presented Results folder [file mep-12-10526-s001.zip › L._ET2._Advocacy_Elective_Assessment_2.pdf]

New Advocacy Assessment Two

1. Advocacy Knowledge & Skills

***1. As a unique identifier, please enter the initals of your first and last name followed by the 2-digit month and 2-digit day of your date of birth. For example, Jane Doe born on October 3rd would enter: JD1003.**

***2. What is your current level of medical training?**

- (a) 3rd-year Medical Student
- (b) 4th-year Medical Student
- (c) PL-1 Pediatrics Resident
- (d) PL-2 Family and Community Medicine Resident
- (e) PL-2 Pediatrics Resident
- (f) PL-3 Pediatrics Resident

***3. Please rate your knowledge/understanding of each of the following AT THE PRESENT TIME**

	No knowledge	Minimal knowledge	Some knowledge	Substantial knowledge	Expert knowledge
Social-welfare system	☐	☐	☐	☐	☐
Foster (substitute) care system	☐	☐	☐	☐	☐
Public education system	☐	☐	☐	☐	☐
Juvenile justice system	☐	☐	☐	☐	☐
Mental health system	☐	☐	☐	☐	☐
Health care and insurance system	☐	☐	☐	☐	☐

New Advocacy Assessment Two

***4. Please rate your knowledge/understanding of the following advocacy topics AT THE PRESENT TIME**

	No knowledge	Minimal knowledge	Some knowledge	Substantial knowledege	Expert knowledge
Health care financing	☐	☐	☐	☐	☐
Developmental disabilities & chronic illness	☐	☐	☐	☐	☐
Adolescent sexuality & pregnancy	☐	☐	☐	☐	☐
Cultural dynamics	☐	☐	☐	☐	☐
Poverty	☐	☐	☐	☐	☐
Housing & homelessness	☐	☐	☐	☐	☐
Child abuse	☐	☐	☐	☐	☐
Breastfeeding	☐	☐	☐	☐	☐
Childcare & early childhood education	☐	☐	☐	☐	☐
Violence prevention	☐	☐	☐	☐	☐
Unintentional injury	☐	☐	☐	☐	☐
Environmental health	☐	☐	☐	☐	☐
Environmental tobacco smoke	☐	☐	☐	☐	☐

New Advocacy Assessment Two

*5. Please rate your comfort/ability to do the following AT THE PRESENT TIME

	Not at all comfortable	Minimally comfortable	Moderately comfortable	Very comfortable
Communicate with a family regardless of culture	☐	☐	☐	☐
Testify in court about one of your patients	☐	☐	☐	☐
Lobby in support of legislation to benefit children's health	☐	☐	☐	☐
Be media spokesperson for a specific child health issue	☐	☐	☐	☐
Work with community-based organizations to serve children	☐	☐	☐	☐
Advocate with an insurance company Medical Director for the needs of a patient	☐	☐	☐	☐
Develop a working knowledge of a specific public health issue affecting children in your practice or community	☐	☐	☐	☐
Design a program to address a specific child health issue	☐	☐	☐	☐
Network with key stakeholders to improve children's health	☐	☐	☐	☐
Help government develop policy for children	☐	☐	☐	☐

*6. Write a definition of health advocacy (2-3 sentences long)

*7. The American Academy of Pediatrics describes seven components of the medical home. List those of which you are aware.

*8. List 2 ways that Medicaid and Medicare differ.

*9. List up to 4 reasons why physicians are ideal health advocates.

*10. Describe the socio-ecological model as it pertains to a health problem or behavior

*11. List several different ways that physicians can advocate for health.

*12. What role does the media (e.g. newspapers, television, radio) play in health advocacy?

*13. What are the crucial legislative steps for a bill to become a law?

*14. Provide an example of a health problem and list the relevant stakeholder groups in the community

*15. For the problem and stakeholders described in #14, provide examples of differing viewpoints held by each stakeholder that makes policy development a challenge.

*16. When building community collaborations, it is essential to hear others' viewpoints. List up to 4 reasons why physicians may find it difficult to listen to others' perspectives particularly on issues related to health.

***17. List up to 5 barriers that low income families may encounter when managing a family member's health problem.**

***18. An emergency medicine physician is concerned about the number of cases of child physical abuse seen in her community. List a variety of ways that she might advocate to reduce the rate of child abuse in her community.**

***19. Breastfeeding rates at your local hospital are only 8%. In your capacity as a respected physician, list several ways you might advocate for hospital policy changes to increase breastfeeding rates.**

***20. A pulmonologist is seeing poorly controlled asthma in his urban practice. List several ways in which he might advocate for social and physical environmental changes that could impact asthma rates.**

***21. Name the two US senators from the state of Pennsylvania**

***22. Who is the mayor of Philadelphia?**

2. Advocacy Inventory

***1. Do you see yourself participating in advocacy work in your future professional role or roles?**

- ☐ Yes
- ☐ No

***2. What is your current level of interest in advocacy?**

☐ No interest ☐ Minimal interest ☐ Moderate interest ☐ Very interested

***3. What impact can you make by incorporating advocacy into your medical career?**

- ○ Not really any impact
- ○ Minor impact
- ○ Moderate impact
- ○ Major impact

Please explain your answer

[ ]

***4. The following issues might be factors for a physician in the decision to be an advocate.**
Rate how important you anticipate each factor will be for you.

	Not important	Minimally important	Moderately important	Very important
Time	☐	☐	☐	☐
Financial considerations	☐	☐	☐	☐
Knowledge	☐	☐	☐	☐
Skills	☐	☐	☐	☐
Personal inclination	☐	☐	☐	☐
Importance of issue	☐	☐	☐	☐
Not part of my job	☐	☐	☐	☐
Someone else is doing it	☐	☐	☐	☐
Likelihood that it will make a difference	☐	☐	☐	☐

Other factor - Please write in

[ ]

***5. In your future professional role/s, do you see yourself advocating for the following?**

	Never	Rarely	Sometimes	Frequently	Always
Individual patients	☐	☐	☐	☐	☐
Changes in policy in my workplace or hospital that will improve the care of my patients	☐	☐	☐	☐	☐
Changes in policy in my workplace or hospital that will improve the health of individuals in the community	☐	☐	☐	☐	☐
Changes in policy in my township or city that will improve the health of individuals in the community	☐	☐	☐	☐	☐
Changes in policy in my state that will improve the health of individuals in the community	☐	☐	☐	☐	☐
Changes in policy nationally that will improve the health of individuals in the community	☐	☐	☐	☐	☐

***6. How often do you read a national (i.e., covers national news) newspaper?**

○ Never ○ Rarely ○ Sometimes ○ Frequently ○ Daily

***7. How often do you read a local (i.e. covers local news) newspaper?**

○ Never ○ Rarely ○ Sometimes ○ Frequently ○ Daily

***8. How often do you watch TV news that covers national events?**

○ Never ○ Rarely ○ Sometimes ○ Frequently ○ Daily

***9. How often do you watch TV news that covers local events?**

○ Never ○ Rarely ○ Sometimes ○ Frequently ○ Daily

***10. How sure are you that you can identify advocacy issues when you see patients?**

☐ Not sure ☐ Somewhat sure ☐ Very sure

***11. How sure are you that you can identify advocacy issues in the community?**

☐ Not sure ☐ Somewhat sure ☐ Very sure

***12. How important is it for a physician to do the following in their professional lives?**

	Not important at all	Not very important	Important	Very important
Engage in work on behalf of children/adults/families in community settings sometime in career	◌	◌	◌	◌
Make a substantial salary	◌	◌	◌	◌
Work in settings of great need	◌	◌	◌	◌
Live a lifestyle with some luxuries	◌	◌	◌	◌
Have training about advocacy	◌	◌	◌	◌
Quickly pay back student loans	◌	◌	◌	◌
Be considered a "pillar of the community"	◌	◌	◌	◌
