# Supplementary material for: The JeffSTARS Advocacy and Community Partnership Elective: A Closer Look at Child Health Advocacy in Action
Source: MedEdPORTAL. 2016 Dec 31;12:10526. doi: 10.15766/mep_2374-8265.10526 (PMC6365684; doi:10.15766/mep_2374-8265.10526)
Supplement: Supplementary file 1 — A. CM1. Course Implementation at New Institution Checklist.docx B. CM2. Elective Checklist.docx C. CM3. Sample Schedule.docx D. CM4. Seminar Topic List With Learning Objectives.docx E. CM5. Syllabus Bibliography.docx F. CM6. List of Community Partners.docx G. CM7. Orientation for New Community Partner.docx H. CM8. Selected Past Projects.docx I. CM9. Sample Fact Sheets for Legislative Visits.docx J. Seminar Materials folder K. ET1. Advocacy Elective Assessment 1.pdf L. ET2. Advocacy Elective Assessment 2.pdf M. ET3. Trainee Evaluation by Community or Faculty Mentor.docx N. ET4. Trainee Evaluation of Seminar.docx O. ET5. Trainee Evaluation of Community Partner.docx P. ET6. Final Report Template.docx Q. Selected Trainee Abstracts and Presented Results folder [file mep-12-10526-s001.zip › M._ET3._Trainee_Evaluation_by_Community_or_Faculty_Mentor.docx]

**The JeffSTARS Curriculum – Advocacy Elective**

**ET3**

**Evaluation of Advocacy Elective Trainee by Community or Faculty Mentor**

***Please comment about the trainee’s ability in the following 3 domains.***

***Particularly note change in the trainee’s competency/skills over the course of the elective.***

**Trainee Name:____**

**Mentor:____**

**____________________________________________________________**

**Written Project Report**

***Overall Rating of Project Report***

**1 2 3 4 5**

**Low High**

**Knowledge**

***Overall Rating of Knowledge***

**1 2 3 4 5**

**Low High**

**Attitudes/Professionalism**

***Overall Rating of Attitudes/Professionalism***

**1 2 3 4 5**

**Low High**

**Skills**

***Overall Rating of Skills***

**1 2 3 4 5**

**Low High**

**Please comment on, giving examples whenever possible and rate from 1 (low) to 5 (High) or NO (No Opportunity to observe) the following performance areas:**

**Ability to serve as a Consultant/Collaborative Leader/Partner**

**Comments:**

**1 2 3 4 5 NO**

**Low High**

**Ability to practice from a population–based perspective and to understand relationships between individual, family, and community-level health determinants that affect children/patients and families in the communities they serve**

**Comments:**

**1 2 3 4 5 NO**

**Low High**

**Competency in the care of children/patients in special populations, including (but not limited to) children and youth in substitute care, homeless children and youth, children and youth with chronic conditions, immigrants and refugees, and children and youth who are adopted**

**Comments:**

**1 2 3 4 5 NO**

**Low High**

**Advocacy skills to address relevant individual, community, and population health issues**

**Comments:**

**1 2 3 4 5 NO**

**Low High**

**Skills that result in effective care of children/patients and families from all cultural backgrounds and from diverse communities**

**Comments:**

**1 2 3 4 5 NO**

**Low High**

**Awareness of and responsiveness to the larger context and system of health care and the ability to effectively call on system resources to provide care that is of optimal value**

**Comments:**

**1 2 3 4 5 NO**

**Low High**

**Interpersonal communication skills**

**Comments:**

**1 2 3 4 5 NO**

**Low High**

**Professionalism, with a commitment to carrying out professional responsibilities, adherence to ethical principles, and sensitivity to a diverse patient population**

**Comments:**

**1 2 3 4 5 NO**

**Low High**

**Please add any additional comments, giving examples whenever possible, about the trainee’s ability in the domains of knowledge, attitudes and skills.**

**Particularly note change in the trainee’s competency/skills over the course of the elective.**
