# Supplementary material for: The JeffSTARS Advocacy and Community Partnership Elective: A Closer Look at Child Health Advocacy in Action
Source: MedEdPORTAL. 2016 Dec 31;12:10526. doi: 10.15766/mep_2374-8265.10526 (PMC6365684; doi:10.15766/mep_2374-8265.10526)
Supplement: Supplementary file 1 — A. CM1. Course Implementation at New Institution Checklist.docx B. CM2. Elective Checklist.docx C. CM3. Sample Schedule.docx D. CM4. Seminar Topic List With Learning Objectives.docx E. CM5. Syllabus Bibliography.docx F. CM6. List of Community Partners.docx G. CM7. Orientation for New Community Partner.docx H. CM8. Selected Past Projects.docx I. CM9. Sample Fact Sheets for Legislative Visits.docx J. Seminar Materials folder K. ET1. Advocacy Elective Assessment 1.pdf L. ET2. Advocacy Elective Assessment 2.pdf M. ET3. Trainee Evaluation by Community or Faculty Mentor.docx N. ET4. Trainee Evaluation of Seminar.docx O. ET5. Trainee Evaluation of Community Partner.docx P. ET6. Final Report Template.docx Q. Selected Trainee Abstracts and Presented Results folder [file mep-12-10526-s001.zip › N._ET4._Trainee_Evaluation_of_Seminar.docx]

**The JeffSTARS Curriculum – Advocacy Elective**

**ET4**

**Evaluation by Advocacy Elective Trainee of Seminar**

**Title:**

Speaker:

**1) *Which aspects of this session were especially, useful, valuable, interesting or new?***

**2) *If this session were to be repeated, what recommendations do you make for change?***

**3) Overall Rating**

**1 2 3 4 5**

**Low High**

**4) Advocacy Areas addressed:**

- Culturally effective care
- Child advocacy
- Medical home
- Special populations
- Pediatrician as Consultant/Collaborative Leader/Partner
- Educational and Child Care settings
- Public Health and Prevention
- Inquiry and Application

**5) ACGME Competencies addressed:**

- Patient care
- Medical knowledge
- Practice-based Learning and Improvement
- Interpersonal and Communication skills
- Professionalism
- Systems-Based Practice
