# Supplementary material for: The JeffSTARS Advocacy and Community Partnership Elective: A Closer Look at Child Health Advocacy in Action
Source: MedEdPORTAL. 2016 Dec 31;12:10526. doi: 10.15766/mep_2374-8265.10526 (PMC6365684; doi:10.15766/mep_2374-8265.10526)
Supplement: Supplementary file 1 — A. CM1. Course Implementation at New Institution Checklist.docx B. CM2. Elective Checklist.docx C. CM3. Sample Schedule.docx D. CM4. Seminar Topic List With Learning Objectives.docx E. CM5. Syllabus Bibliography.docx F. CM6. List of Community Partners.docx G. CM7. Orientation for New Community Partner.docx H. CM8. Selected Past Projects.docx I. CM9. Sample Fact Sheets for Legislative Visits.docx J. Seminar Materials folder K. ET1. Advocacy Elective Assessment 1.pdf L. ET2. Advocacy Elective Assessment 2.pdf M. ET3. Trainee Evaluation by Community or Faculty Mentor.docx N. ET4. Trainee Evaluation of Seminar.docx O. ET5. Trainee Evaluation of Community Partner.docx P. ET6. Final Report Template.docx Q. Selected Trainee Abstracts and Presented Results folder [file mep-12-10526-s001.zip › O._ET5._Trainee_Evaluation_of_Community_Partner.docx]

**The JeffSTARS Curriculum – Advocacy Elective**

**ET5**

**Evaluation by Advocacy Elective Trainee of Community Partner**

**Community Partner Site (name of organization and supervisor):**

Please rate your community site on the following factors:

**Welcome and Orientation**

1 2 3 4 5

Low High

*Comments*

**The quality of the mentoring and supervision you received**

1 2 3 4 5

Low High

*Comments*

**The availability of your mentor or supervisor**

1 2 3 4 5

Low High

*Comments*

**Facilitation of your work**

1 2 3 4 5

Low High

*Comments*

**Your integration into the community partner’s work setting**

1 2 3 4 5

Low High

*Comments*

**Learning opportunities**

1 2 3 4 5

Low High

*Comments*

**Overall Rating of Community Partner Experience**

1 2 3 4 5

Low High

*Comments*
