# Supplementary material for: The JeffSTARS Advocacy and Community Partnership Elective: A Closer Look at Child Health Advocacy in Action
Source: MedEdPORTAL. 2016 Dec 31;12:10526. doi: 10.15766/mep_2374-8265.10526 (PMC6365684; doi:10.15766/mep_2374-8265.10526)
Supplement: Supplementary file 1 — A. CM1. Course Implementation at New Institution Checklist.docx B. CM2. Elective Checklist.docx C. CM3. Sample Schedule.docx D. CM4. Seminar Topic List With Learning Objectives.docx E. CM5. Syllabus Bibliography.docx F. CM6. List of Community Partners.docx G. CM7. Orientation for New Community Partner.docx H. CM8. Selected Past Projects.docx I. CM9. Sample Fact Sheets for Legislative Visits.docx J. Seminar Materials folder K. ET1. Advocacy Elective Assessment 1.pdf L. ET2. Advocacy Elective Assessment 2.pdf M. ET3. Trainee Evaluation by Community or Faculty Mentor.docx N. ET4. Trainee Evaluation of Seminar.docx O. ET5. Trainee Evaluation of Community Partner.docx P. ET6. Final Report Template.docx Q. Selected Trainee Abstracts and Presented Results folder [file mep-12-10526-s001.zip › P._ET6._Final_Report_Template.docx]

**The JeffSTARS Curriculum – Advocacy Elective**

**ET6**

**Advocacy Elective Final Report Template**

Thank you for participating in this elective. We hope that this has helped you in your journey to being a health advocate. Please answer the following questions electronically, and submit no later than the last day of your rotation. Please leave these headers in place, do not alter the margins or font, and write your responses in prose. Your final report should be approximately 5-10 pages of double-spaced text.

**A. Describe your expectations and goals for this elective related to 1) advocacy in general, and 2) your project. Were you able to reach these goals? Please explain.**

**B. Describe your advocacy project**

1. **What were successes related to the project?**
2. **What were challenges related to the project?**
3. **What are major lessons learned from working on your project?**

**C. Describe your community partner.**

1. **What were advantages of working with the community partner?**
2. **What were limitations and challenges in working with the community partner?**
3. **What are major lessons learned from working with the community partner?**

**D. Describe the extent to which the seminars and mentor meetings impacted your elective, project, and overall advocacy knowledge.**

**E. Please note any unexpected opportunities.**
