# Supplementary material for: The JeffSTARS Advocacy and Community Partnership Elective: A Closer Look at Child Health Advocacy in Action
Source: MedEdPORTAL. 2016 Dec 31;12:10526. doi: 10.15766/mep_2374-8265.10526 (PMC6365684; doi:10.15766/mep_2374-8265.10526)
Supplement: Supplementary file 1 — A. CM1. Course Implementation at New Institution Checklist.docx B. CM2. Elective Checklist.docx C. CM3. Sample Schedule.docx D. CM4. Seminar Topic List With Learning Objectives.docx E. CM5. Syllabus Bibliography.docx F. CM6. List of Community Partners.docx G. CM7. Orientation for New Community Partner.docx H. CM8. Selected Past Projects.docx I. CM9. Sample Fact Sheets for Legislative Visits.docx J. Seminar Materials folder K. ET1. Advocacy Elective Assessment 1.pdf L. ET2. Advocacy Elective Assessment 2.pdf M. ET3. Trainee Evaluation by Community or Faculty Mentor.docx N. ET4. Trainee Evaluation of Seminar.docx O. ET5. Trainee Evaluation of Community Partner.docx P. ET6. Final Report Template.docx Q. Selected Trainee Abstracts and Presented Results folder [file mep-12-10526-s001.zip › Q._Selected_Trainee_Abstracts_and_Presented_Results_folder/Lead_Poisoning.pdf]

Title: The Role of Future Pediatricians in Advocating for Lead Prevention in Schools

Alissa S Werzen, BA[1], Maura McInerney, JD[2], Esther K Chung, MD, MPH[3] and James D Plumb, MD, MPH[4]. [1]Sidney Kimmel Medical College of Thomas Jefferson U., Philadelphia, PA, United States; [2]Education Law Center, Philadelphia, PA, United States; [3]Pediatrics, Nemours and Thomas Jefferson U., Philadelphia, PA, United States and [4]Family and Community Medicine, Sidney Kimmel Medical College of Thomas Jefferson U., Philadelphia, PA, United States.

Background: Although the health effects of lead have been well-described, an emerging body of research asserts its adverse association with scores on tests of aptitude, educational attainment and behavior. In Philadelphia, almost 95% of the housing stock was built prior to federal legislation banning lead paint. As many of the city's schoolchildren remain at risk for continued lead exposure, physician advocacy is needed to raise awareness among educators and lawmakers as to its untoward effects to develop a comprehensive, multidisciplinary approach to lead mitigation.

Objective: 1) In conjunction with a legal advocacy organization, explore the ways in which chronic lead exposure impacts child growth and development 2) Share results of a literature review with schoolteachers and education advocates to galvanize them to intervene on this issue 3) Develop recommendations as to how to mitigate sources of ongoing lead exposure

Design/Methods: This was a collaborative project between a 4th-year medical student via a one-month elective with JeffSTARS, an advocacy curriculum, and the Education Law Center, an organization that advocates for a quality education for all Pennsylvania schoolchildren. We developed four targeted policy recommendations and prepared an issue statement detailing lead's detrimental association with learning ability, educational attainment, and behavior, with a focus on the need for special education services.

Results: The following policy recommendations were developed:1) Schools can play a role in ensuring that all children are screened for elevated blood lead levels 2) As lead in school drinking fountains remains a common source of exposure, schools should test their drinking water and move to a bottle-based system 3) Schools can connect parents and families with community resources for lead abatement 4) Immigrant and refugee children are at particular risk for the development of elevated blood lead levels and should be more closely monitored upon their arrival to the US.

Conclusions: As a result of this partnership, an issue statement and policy recommendations were developed to reduce lead exposure among children. Working with the Education Law Center allowed a future physician to understand how lawyers and physicians can work together to impact education policy as it relates to health. This elective reinforced a career interest in advocacy, as it allowed for the perfect amalgamation of public policy, population health, and patient care.
