# Supplementary material for: The JeffSTARS Advocacy and Community Partnership Elective: A Closer Look at Child Health Advocacy in Action
Source: MedEdPORTAL. 2016 Dec 31;12:10526. doi: 10.15766/mep_2374-8265.10526 (PMC6365684; doi:10.15766/mep_2374-8265.10526)
Supplement: Supplementary file 1 — A. CM1. Course Implementation at New Institution Checklist.docx B. CM2. Elective Checklist.docx C. CM3. Sample Schedule.docx D. CM4. Seminar Topic List With Learning Objectives.docx E. CM5. Syllabus Bibliography.docx F. CM6. List of Community Partners.docx G. CM7. Orientation for New Community Partner.docx H. CM8. Selected Past Projects.docx I. CM9. Sample Fact Sheets for Legislative Visits.docx J. Seminar Materials folder K. ET1. Advocacy Elective Assessment 1.pdf L. ET2. Advocacy Elective Assessment 2.pdf M. ET3. Trainee Evaluation by Community or Faculty Mentor.docx N. ET4. Trainee Evaluation of Seminar.docx O. ET5. Trainee Evaluation of Community Partner.docx P. ET6. Final Report Template.docx Q. Selected Trainee Abstracts and Presented Results folder [file mep-12-10526-s001.zip › Q._Selected_Trainee_Abstracts_and_Presented_Results_folder/Youth_Sentencing.pdf]

Title: Life, Interrupted: Advocating for Youth Sentenced to Life Without Parole

Laura V Livaditis, BA[1], Lauren Fine, Esq[2], Joanna Visser Adjoian, Esq[2] and Esther K Chung, MD, MPH[1].
[1]Nemours/Thomas Jefferson U., Philadelphia, PA, United States and [2]Youth Sentencing & Reentry Project, Philadelphia, PA, United States.

Background: The U.S. is the only nation that sentences youth as adults to life in prison without parole. Opportunities exist at juvenile hearings to argue for appropriate sentencing recommendations. However, many legal representatives remain unaware of how youth differ from adults psychosocially and developmentally. To our knowledge, no source has approached youth sentencing from a health outcomes perspective, connecting psychosocial and developmental risk factors for illicit behavior with health consequences of long-term incarceration.

Objective: 1) To detail neurobiological differences in decision-making between youth and adults, and 2) to understand childhood traumatic exposures as risk factors for criminal behavior.

Design/Methods: Through a collaboration between JeffSTARS, an advocacy program, and the Youth Sentencing & Reentry Project, a non-profit legal organization advocating for youth in the adult criminal justice system, a 4th year medical student explored ways in which neurodevelopment and childhood traumatic exposures impact juvenile criminal behavior.

Results: As a result of this partnership, two reports were created: *Guide to Adolescent Brain Development* and *Effects of Childhood Traumatic Events on Adolescent Development and Behavior*. These reports distinguish juvenile from adult offenders by detailing various risk factors that uniquely predispose youth to engage in illicit behavior. Adolescents tend to be more impulsive, less likely to consider future consequences, and more likely than adults to employ decision-making patterns in which rewards are favored over consequences. Also, adolescents exposed to forms of violence are more likely to commit violent acts. As trauma is processed via externalized, aggressive behavior, victims become trapped in cycles of exposure and victimization that lead to poor social, legal and medical outcomes. By applying knowledge of development and trauma outcomes to show increased propensity for risk-taking and violence perpetuation, physicians can empower legal representatives to present the most effective arguments for fair sentencing of youth.

Conclusions: Opportunities exist at juvenile hearings to argue against juvenile life without parole in favor of thoughtful, developmentally appropriate sentencing recommendations. By applying knowledge of neurobiological immaturity and sequelae of childhood traumatic exposures, pediatric providers can partner with legal representatives to advocate for fair sentencing of youth.
